# A Highly Potent Class of Halogenated Phenazine Antibacterial and Biofilm-Eradicating Agents Accessed Through a Modular Wohl-Aue Synthesis

**DOI:** 10.1038/s41598-017-01045-3

**Published:** 2017-05-17

**Authors:** Hongfen Yang, Yasmeen Abouelhassan, Gena M. Burch, Dimitris Kallifidas, Guangtao Huang, Hussain Yousaf, Shouguang Jin, Hendrik Luesch, Robert W. Huigens

**Affiliations:** 1 Department of Medicinal Chemistry, Center for Natural Products Drug Discovery and Development (CNPD3), College of Pharmacy, University of Florida, 1345 Center Dr., Gainesville, FL 32610 USA; 2 Department of Molecular Genetics and Microbiology, College of Medicine, University of Florida, 1200 Newell Drive, Gainesville, FL 32610 USA

## Abstract

Unlike individual, free-floating planktonic bacteria, biofilms are surface-attached communities of slow- or non-replicating bacteria encased within a protective extracellular polymeric matrix enabling persistent bacterial populations to tolerate high concentrations of antimicrobials. Our current antibacterial arsenal is composed of growth-inhibiting agents that target rapidly-dividing planktonic bacteria but not metabolically dormant biofilm cells. We report the first modular synthesis of a library of 20 halogenated phenazines (HP), utilizing the Wohl-Aue reaction, that targets both planktonic and biofilm cells. New HPs, including 6-substituted analogues, demonstrate potent antibacterial activities against MRSA, MRSE and VRE (MIC = 0.003–0.78 µM). HPs bind metal(II) cations and demonstrate interesting activity profiles when co-treated in a panel of metal(II) cations in MIC assays. HP **1** inhibited RNA and protein biosynthesis while not inhibiting DNA biosynthesis using ^3^H-radiolabeled precursors in macromolecular synthesis inhibition assays against MRSA. New HPs reported here demonstrate potent eradication activities (MBEC = 0.59–9.38 µM) against MRSA, MRSE and VRE biofilms while showing minimal red blood cell lysis or cytotoxicity against HeLa cells. PEG-carbonate HPs **24** and **25** were found to have potent antibacterial activities with significantly improved water solubility. HP small molecules could have a dramatic impact on persistent, biofilm-associated bacterial infection treatments.

## Introduction

**Figure 1:**
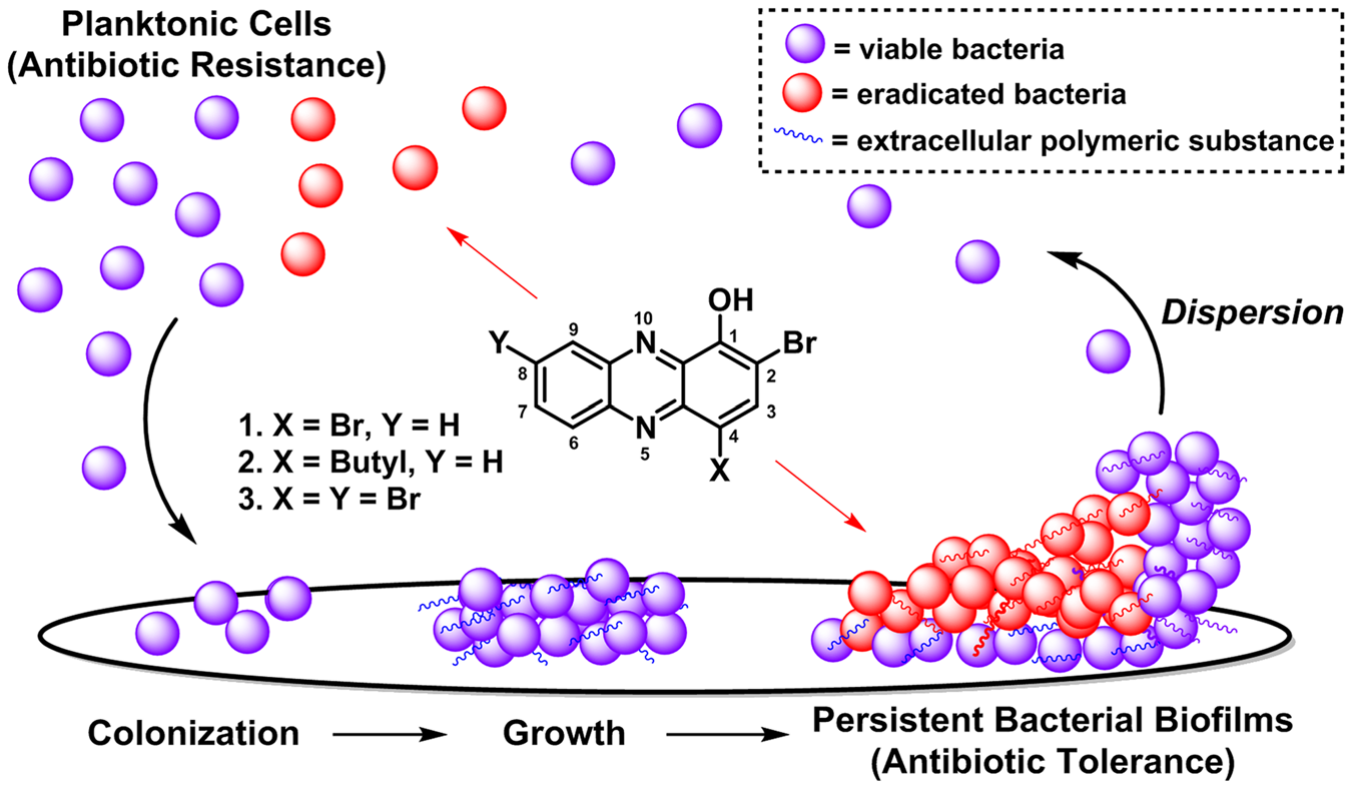
﻿Illustration of free-swimming planktonic bacteria, persistent surface-attached biofilms and halogenated phenazine small molecules that effectively eradicate both bacterial lifestyles.

Pathogenic bacteria present two distinct biomedical challenges, which include (1) antibiotic resistance^1–3^ and (2) antibiotic tolerance^3–6^. Rapidly-dividing, free-floating planktonic bacteria have multiple well-defined mechanisms by which they gain, or develop, resistance to antibiotics^2, 3^. We have learned that planktonic bacteria use a signaling process known as quorum sensing to communicate and coordinate the simultaneous attachment to a surface for initial colonization and biofilm development (Fig. 1)^7, 8^. Bacterial biofilms are surface-attached communities of metabolically dormant (slow- or non-replicating) bacteria encased within a protective extracellular polymeric matrix of biomolecules that enable them to thrive in hostile environments^8–12^. Antibiotic tolerance results from persistent, non-replicating bacteria (i.e., biofilms) and are the underlying cause of chronic infections, which affect 17 million individuals and lead to >500,000 deaths each year^13, 14^.

Our entire antibiotic arsenal is composed of growth-inhibiting agents that hit bacterial targets critical to rapidly-dividing bacteria (e.g., cell wall machinery, bacterial ribosomes)^3^. The large majority of our antibiotic classes were discovered before 1970, which is ~25 years before bacterial biofilms were identified as a biomedical problem^15^. Despite the historical significance of natural products and phenotype screening in antibiotic discovery, the paradigm shift in drug discovery to high throughput screening of synthetic libraries in target-based screens has generated no new chemical entities that have entered the market to date^16^. To make matters worse, few pharmaceutical companies continue antibiotic discovery programs^17^.

Innovative discovery strategies must be implemented to identify compounds that effectively eradicate persistent, antibiotic-tolerant biofilms that operate via unconventional, growth-independent mechanisms. Considering the history of antibiotic discovery, which relies heavily on microbial warfare agents (i.e., penicillin, streptomycin, vancomycin), it stands to reason that alternative microbial warfare agents exist that include biofilm-eradicating agents we have yet to harness for therapeutic purposes^18^. Clinically effective biofilm-eradicating agents would significantly change bacterial treatments and enable the control of persistent biofilm infections.

To develop a new anti-biofilm strategy, our group has been inspired by the microbial interaction between *Pseudomonas aeruginosa* and *Staphylococcus aureus* in the lungs of young Cystic Fibrosis (CF) patients^19^. When CF patients are young, they often experience *S*. *aureus* lung infections; however, as these CF patients age, *P*. *aeruginosa* co-infects the lung and secretes a series of phenazine antibiotics which help eradicate *S*. *aureus* to become the primary pathogen in the CF patient’s lung. With this microbial interaction in mind, we felt there was a strong possibility that the eradication of established *S*. *aureus* biofilms enabled *P*. *aeruginosa* to successfully compete for the CF patient’s lung. We reasoned that phenazine antibiotic metabolites^20, 21^ may be an ideal starting point to explore unique antibacterial agents that eradicate bacterial biofilms. During our initial studies, we explored a diverse panel of phenazine small molecules, including several phenazine antibiotics^22^. From these investigations, we identified the marine phenazine antibiotic 2-bromo-1-hydroxyphenazine and its brominated analogue, 2,4-dibromo-1-hydroxyphenazine **1**, as potent antibacterial agents against *S*. *aureus* (MIC = 6.25 and 1.56 µM, respectively). These halogenated phenazines demonstrated significantly higher antibacterial activity compared to other phenazine antibiotics, incliding pyocyanin.

We have identified a focused series of HP analogues (i.e., **1–3**, Fig. 1) that potently eradicates planktonic and biofilms of multiple drug-resistant pathogens, including: *S*. *aureus*, *S*. *epidermidis* and *Enterococcus faecium* ^23–25^. Based on our preliminary findings, HPs bind copper(II) and iron(II) and elicit their potent antibacterial activities through a metal(II)-dependent mechanism^25^. Our pursuits have been met with challenges regarding the chemical synthesis of the HP scaffold. Our previous work has enabled the exploration of 7,8-disubstituted HPs along with 2,4-mixed halogenations HPs with the use of *o*-phenylenediamines building blocks^24, 25^; however, the availability of these building blocks does not enable the level of diversity necessary for our campaign. Our goal for this work is to develop a rapid and convergent synthesis of HP small molecules that enable us to further explore the 6-, 7-, 8- and 9-positions of the HP scaffold through the use of diverse aniline building blocks.

## Results and Discussion

### Chemical Synthesis of New HPs Using the Wohl-Aue Reaction

**Figure 2:**
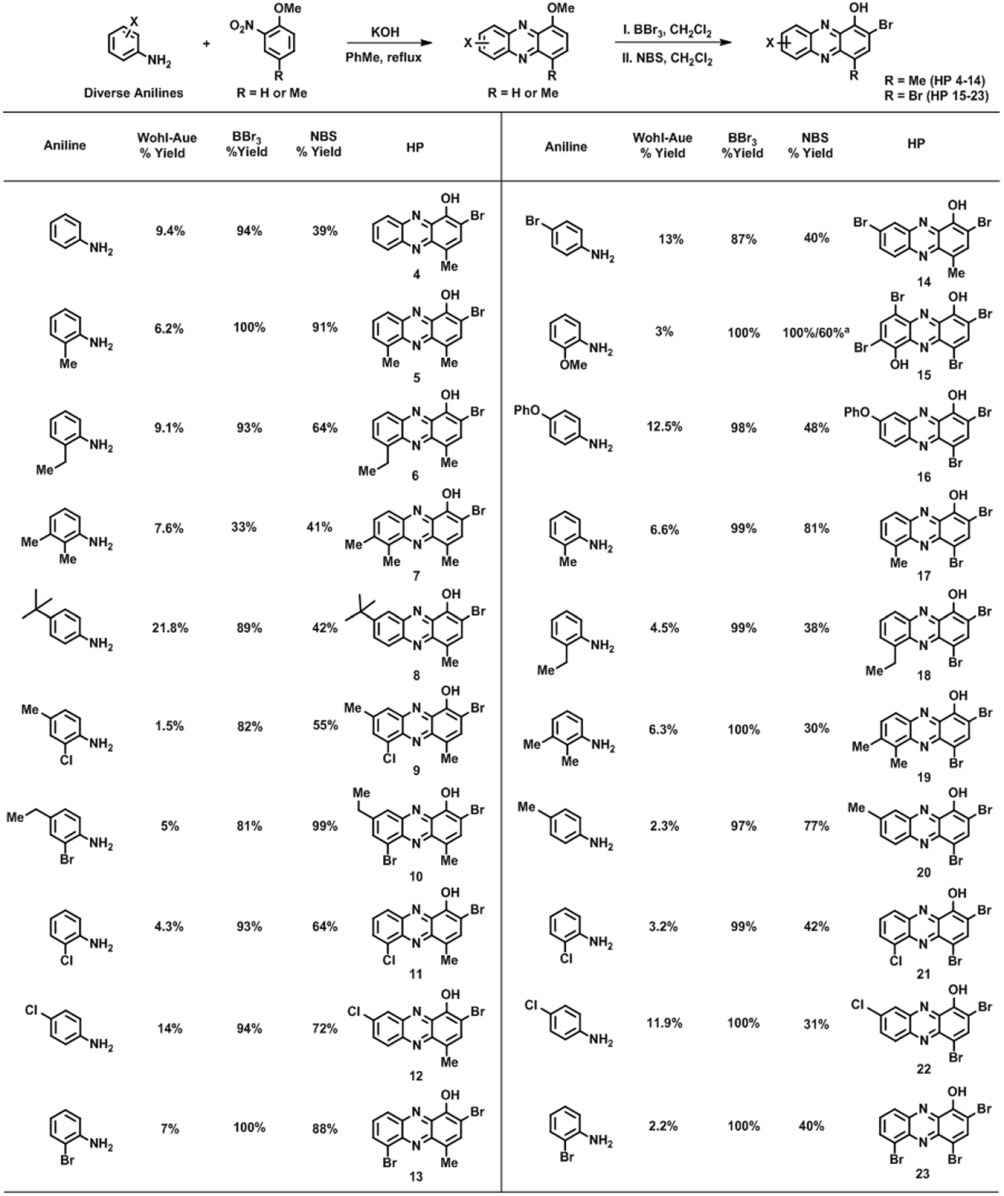
Chemical synthesis of a diverse panel of HPs using a convergent Wohl-Aue route﻿. Note: 1,6-Dimethoxylphenazine (Wohl-Aue product) was dibrominated (NBS), then demethylated (BBr3) before final dibromination (NBS) to give HP 15. See Supporting Information for full synthesis details.

The Wohl-Aue reaction^26, 27^ involves the base-promoted condensation between a nitroarene and an aniline to yield a phenazine. With the potential to diversify the HP scaffold at the 6-, 7-, 8- and 9-positions with an array of substituted aniline building blocks, we utilized 2-nitroanisole and 4-methyl-2-nitroanisole in Wohl-Aue condensation reactions with a panel of 14 diverse anilines to produce a total of 20 new HPs in three steps enabling rapid and extensive biological evaluations (Fig. 2).

We found the Wohl-Aue reaction to be useful in the convergent synthesis of new 1-methoxyphenazine scaffolds; however, we found this reaction to be very low yielding (2–22%, 20 examples; Fig. 2). In addition, it is known that the Wohl-Aue reaction suffers from unproductive side reactions, including bond formation between aniline’s nitrogen and the 4-position of 2-nitroanisole^28^. We attempted to mitigate this undesired reaction by using 4-methyl-2-nitroanisole; however, reaction yields remained generally low regardless of which 2-nitroanisole material was used. The 4-methyl-2-nitroanisole condensation reactions with anilines gave a 9% average yield in the Wohl-Aue reaction (11 examples), while 2-nitroanisole was condensed with anilines to give phenazine products in an average of 6% yield (9 examples). Despite these low yields, these reactions were carried out on scales that produced 40–650 milligrams of diverse 1-methoxyphenazines, which was sufficient for the final two steps of the synthetic sequence. After the Wohl-Aue reaction, 1-methoxyphenazines were subjected to boron tribromide demethylation to afford 1-hydroxyphenazines (33–100% yield, 88% average yield; 20 examples). Final bromination with *N*-bromosuccinimide (NBS) produced 2-bromo-4-methyl-HP analogues **4–14** (39–99% yield, 63% average yield, 11 examples) or 2,4-dibromo-HP analogues **15–23** (30–81% yield, 50% average yield, 9 examples). During our investigations, we were able to prepare 12–220 milligrams of each HP, which enabled sufficient material for biological studies.

### Biological Evaluation of HPs

#### Antibacterial Studies

Following the synthesis of **4–23**, these new HPs were evaluated for their antibacterial activity against methicillin-resistant *S*. *aureus* (MRSA), methicillin-resistant *S*. *epidermidis* (MRSE), vancomycin-resistant *E*. *faecium* (VRE) and *Mycobacterium tuberculosis* (MtB). HPs **1–3** (Fig. 1) are from our previous studies and were used as positive-controls in our MIC assays. In addition, a panel of conventional antibiotics (vancomycin^29^), metal-binding agents (EDTA^30^, TPEN^31, 32^) and membrane-active biofilm eradicator (QAC-10^33^) were used as comparators (Table 1).

**Table 1:** Summary of antibacterial activities and cytotoxicity against HeLa cells for HP analogues. All values are reported in micromolar (µM) concentrations.

Compound	MRSA BAA-1707	MRSA BAA-44	MRSA-1	MRSA-2	MRSE 35984	*S*. *epi* 12228	VRE 700221	MtB H37Ra	HeLa Cytotox. IC_50_
1	1.17^a^	1.56	0.78	2.35^a^	2.35^a^	2.35^a^	4.69^a^	25	~100
2	0.78	2.35^a^	0.78	3.13	0.59^a^	1.56	0.78	—	—
3	0.20	0.78	2.35^a^	0.78	3.13	3.13	0.78	—	—
4	3.13	50	50	3.13	2.35^a^	3.13	3.13	—	>100
5	9.38^a^	—	37.5^a^	—	1.17^a^	—	>100	—	—
6	4.69^a^	—	0.78	—	0.30^a^	—	0.78	—	—
7	4.69^a^	—	50	—	18.8^a^	—	18.8^a^	—	—
8	>100	—	>100	—	>100	—	>100	—	—
9	1.17^a^	1.56	1.56	2.35^a^	0.39	2.35^a^	2.35^a^	50	>100
10	18.8^a^	—	50	—	37.5^a^	—	50	—	—
11	2.35^a^	3.13	0.39	3.13	1.56	1.56	3.13	—	—
12	18.8^a^	—	75^a^	—	9.38^a^	—	12.5	—	—
13	1.17^a^	1.17^a^	0.39	2.35^a^	0.78	1.17^a^	1.17^a^	—	—
14	9.38^a^	—	75^a^	—	75^a^	—	18.8^a^	—	—
15	>50	—	>50	>50	>50	—	>50	—	—
16	9.38^a^	18.8^a^	25	18.8^a^	9.38^a^	18.8^a^	2.35^a^	—	—
17	0.30^a^	0.39	0.10^b^	0.30^a^	0.30^a^	0.39	0.59^a^	25	>100
18	0.10^b^	0.59^a^	0.15^a^	0.39	0.10^b^	0.30^a^	0.15^a^	25	>100
19	0.15^a^	9.38^a^	4.69^a^	4.69^a^	0.30^a^	6.25	0.15^a^	—	>100
20	2.35^a^	3.13	18.8^a^	2.35^a^	6.25	9.38^a^	2.35^a^	—	—
21	0.08^a^	0.59^a^	0.10^b^	0.39	0.30^a^	0.39	1.56	50	>100
22	0.15^a^	0.30^a^	0.15^a^	0.59^a^	18.8^a^	12.5	1.56	6.25	>100
23	0.05	0.59^a^	0.10^b^	0.30^a^	0.78	0.30^a^	1.17^a^	25	>100
24	0.59^a^	1.56	1.17^a^	2.35^a^	1.56	0.78	6.25	25	>100
25	0.003	0.013	0.10^b^	0.10^b^	0.15^a^	0.10^b^	0.59^a^	12.5	>100
EDTA	25	—	12.5	—	—	—	—	—	—
TPEN	46.9^a^	—	75^a^	50	12.5	—	—	—	—
QAC-10	4.69^a^	—	4.69^a^	3.13	2.35^a^	—	2.35^a^	—	—
BAC-16	1.56	—	—	—	1.56	—	3.13	—	—
vancomycin	0.39	0.39	0.39	0.59^a^	0.78	1.17^a^	>100	—	—
daptomycin	3.13	18.8^a^	—	4.69^a^	12.5	6.25	125	—	—
linezolid	12.5	1.56	4.69^a^	3.13	3.13	1.56	3.13	—	—
streptomycin	—	—	—	—	—	—	—	1.32	—

Note: ^a^Midpoint for a 2-fold range in observed values. ^b^Lowest concentration tested. All biological results were generated from three independent experiments.

**Figure 3:**
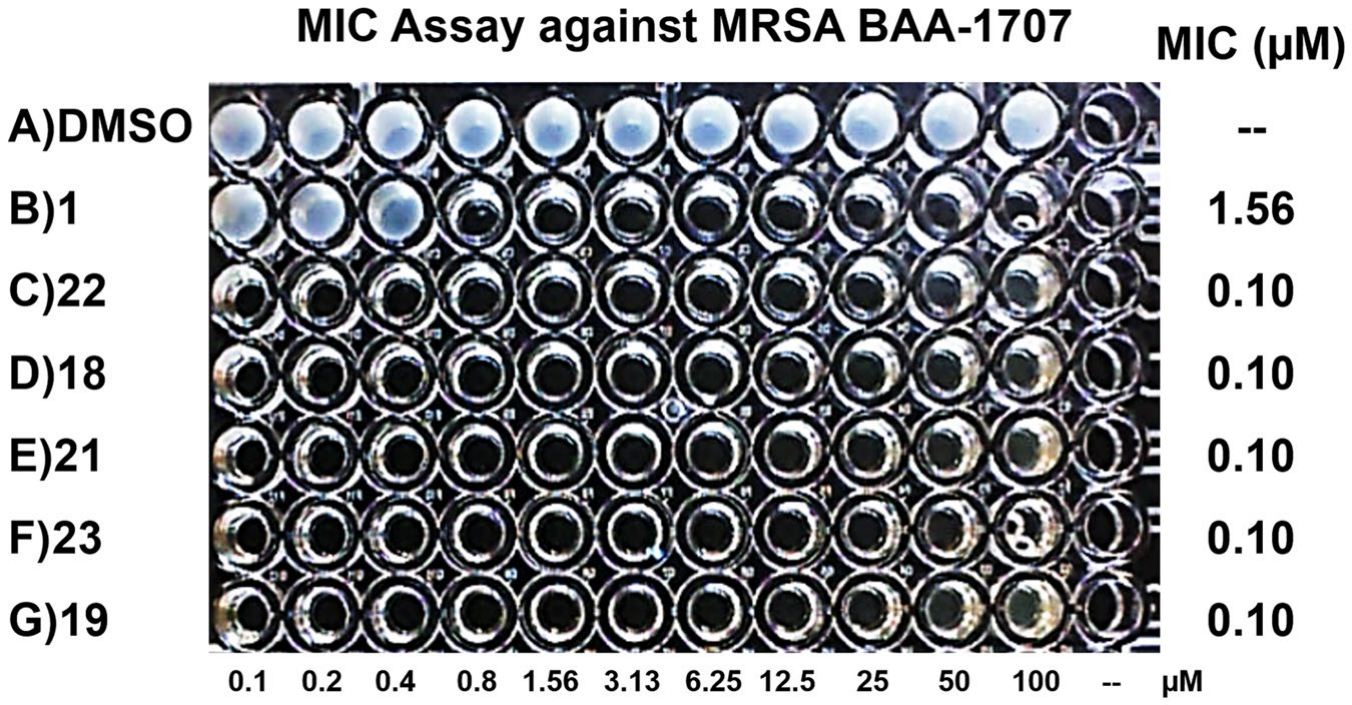
MIC assay results of potent new HPs (MIC = 0.10 µM) against MRSA BAA-1707﻿.

Our Wohl-Aue derived HPs were tested in MIC assays against MRSA BAA-1707. We found that the 4-methyl-HP analogues **4–14** (MIC = 1.17–18.8 µM; **8** was inactive, MIC > 100 µM; Table 1) generally displayed significantly reduced antibacterial activities compared to the 2,4-dibromo-HP series **15–23** (MIC = 0.05–3.13 µM; **16** had an MIC = 9.38 µM; **15** was inactive, MIC > 50 µM). To our surprise, 2,4-dibromo-HP analogues **17**, **18**, **21**, **23** are substituted at the 6-position with either a methyl-, ethyl-, chloro- and bromo- groups and demonstrated potent antibacterial activities against MRSA BAA-1707 (MIC = 0.05–0.30 µM), which is 4- to 16-fold more potent than parent HP **1** (Fig. 3). We have been unable to explore HP analogues containing 6-position substitutions in previous studies; however, by using the Wohl-Aue reaction described herein, diverse 2-alkylated anilines yield 6-substituted HPs. In addition, HPs **17**, **18**, **21**, **23** along with 8-chloro HP **22** proved to be highly potent (MIC = 0.05–0.59 µM) against a panel of four MRSA isolates (Table 1).

In addition to MRSA isolates, several Wohl-Aue derived HPs demonstrated potent antibacterial activities against MRSE and VRE strains (Table 1). 2-Bromo-4-methyl HP analogues **6**, **9** and **13** displayed impressive antibacterial activities against MRSE 35984 (MIC = 0.30–0.78 µM) and VRE 700221 (MIC = 0.78–2.35 µM) while 2,4-dibrominated HP analogues **17**, **18**, **19**, **21** and **23** demonstrated more potent antibacterial activities against MRSE 35984 (MIC = 0.10–0.78 µM) and VRE 700221 (MIC = 0.15–1.56 µM). HPs **18** (6-ethyl HP) demonstrated the most potent antibacterial activities against *S*. *epidermidis* (MIC = 0.10–0.30 µM) and *E*. *faecium* (MIC = 0.15 µM) while **17** (6-methyl HP) gave a similar profile with a slight reduction in antibacterial potency (*S*. *epidermidis* MIC = 0.30–0.39 µM; *E*. *faecium* MIC = 0.59 µM). Each of the HPs discussed here, including the 4-methyl HP analogues, demonstrated improved antibacterial activities against MRSE and VRE compared to parent HP **1** (MRSE MIC = 2.35 µM; VRE MIC = 4.69 µM).

*M*. *tuberculosis* (MtB) is the largest bacterial threat to humans worldwide killing over 1.5 million humans around the globe each year^34, 35^. MtB is a slow-growing or non-replicating pathogen that requires prolonged antibiotic treatments (i.e., ≥6 months), often with multiple drug combinations due to problems with drug-resistance^36–38^. To further compound these problems, currently there is an inadequate antibiotic pipeline for new MtB drugs^37^. With active HPs targeting both rapidly-dividing planktonic and persistent biofilm cells of Gram-positive pathogens, we felt that HPs could demonstrate antibacterial activities against the persistent pathogen, MtB. We previously reported HPs with good anti-TB activity^24, 25^ and, during the course of these studies, we evaluated Wohl-Aue derived HPs for antibacterial activity against MtB H37Ra. We tested a smaller panel of HPs (**9**, **17**, **18**, **21–23**; Table 1) that demonstrated potent antibacterial activities against MRSA, MRSE, and VRE. From these studies, most HPs reported moderate antibacterial activities against MtB H37Ra (MIC = 12.5–50 µM); however, 8-chloroHP analogue **22** (MIC = 6.25 µM, 2.4 µg/mL) demonstrated the most potent antibacterial activity from this series against MtB H37Ra. Unlike our previous investigations that reported lead anti-TB HPs not containing a bromine atom in the 2-position, HP **22** is the most potent 2,4-dibromoHP analogue we have reported to date.

#### Macromolecular Synthesis Inhibition of HP 1 against MRSA BAA-1707 Cultures

**Figure 4:**
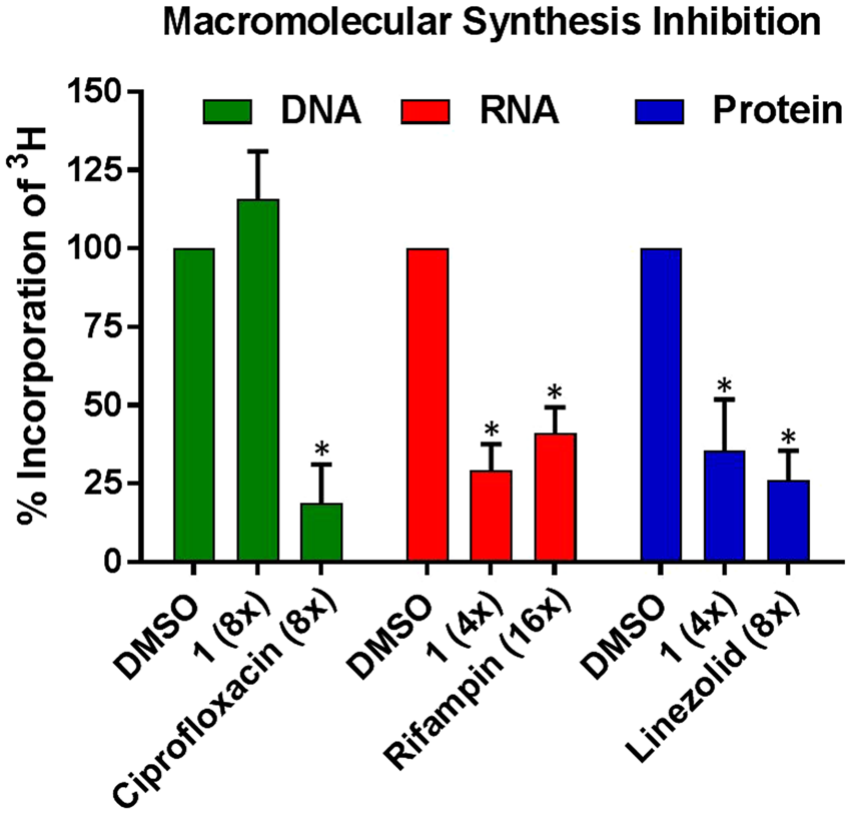
Results of HP 1 against MRSA BAA-1707 in macromolecular syn﻿thesis inhibition experiments with [﻿^3﻿^H]-labeled precursors (generated from three, or more, independent experiments). HP 1 inhibits RNA and protein biosynthesis while not inhibiting DNA biosynthesis (p-values ≤ 0.005*; pairwise student t-test comparing relative treated samples to DMSO vehicle only samples).

To characterize the antibacterial mechanism of action for HP small molecules, we investigated the effects HP **1** has against MRSA BAA-1707 on global biosynthetic pathways in rapidly-dividing (exponentially-growing) planktonic cells through quantifying the incorporation of various [^3^H]-labeled precursors into their corresponding macromolecules^39^. For these experiments, MRSA-1707 cultures were treated with [^3^H]-thymidine (DNA biosynthesis), [^3^H]-uridine (RNA biosynthesis) and [^3^H]-leucine (protein biosynthesis) in the presence of HP **1** and antibiotic controls (4–16 x MIC). From these initial experiments, we found that HP **1** does not inhibit DNA biosynthesis at 8 x MIC; however, at 4 x MIC, HP **1** inhibits both RNA and protein biosynthesis in MRSA-1707 (Fig. 4). Currently, more elaborate mode of action studies are underway in our labs and aim to understand these results in the context of metal(II)-binding and biofilm eradication.

#### Mammalian Cytotoxicity Studies

Several new HPs (**4**, **9**, **17–19**, **21–23**) that displayed potent antibacterial activities against MRSA, MRSE and VRE were evaluated against HeLa cells to determine mammalian cell cytotoxicity in lactate dehydrogenase (LDH) release assays^40^ at 25, 50 and 100 µM. During these investigations, we found parent HP **1** to have an IC_50_ value ~100 µM while the remaining HP analogues recorded IC_50_ values >100 µM (Table 1). When comparing the HeLa cytotoxicity (IC_50_) to the antibacterial activities (MIC) of MRSA BAA-1707, several HPs demonstrated selectivity indexes of >330- to >2,000-fold against MRSA BAA-1707 cells. For example, HP **23** reported an IC_50_ > 100 µM against HeLa cells while reporting potent antibacterial activities with an MIC = 0.05 µM against MRSA BAA-1707, resulting in a selective index of >2,000-fold towards inhibiting MRSA planktonic cells. This promising bacterial selectivity profile is crucial for translating HP antibacterial agents into viable therapeutic options.

#### PEG Carbonate-HP Synthesis

In an effort to enhance the drug-likeness of our active HP small molecules, we synthesized two polyethylene glycol (PEG) carbonates as potential prodrugs, which enables: (1) improvement of water solubility through the installation of a PEG group, (2) enhanced bacterial penetration and release of HP through possible bacterial esterase processing resulting in active HP, carbon dioxide and non-toxic PEG, (3) mitigating the metal-binding moiety required for the antibacterial activities of HPs, which would be important in the development of HPs in more advanced preclinical studies. We selected parent HP **1** (CLogP: 4.68) and active HP **17** (CLogP: 5.18) to design PEG carbonate-HPs that would have improved water solubility (reduced CLogP values; calculated using ChemBioDraw Ultra, version 13).

**Figure 5:**
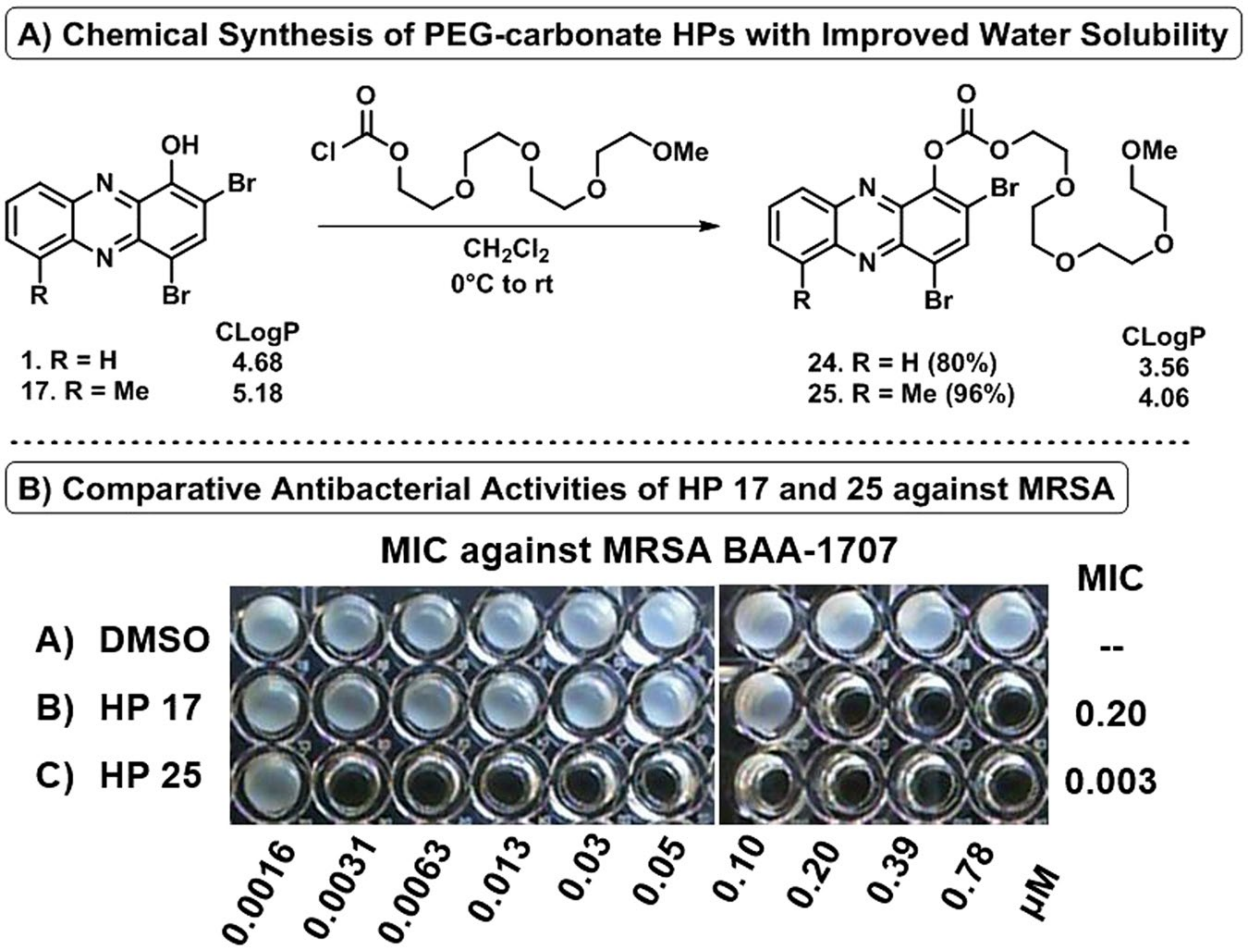
(**A**) Chemical synthesis of PEG-carbonate HPs 24 and 25. (**B**) Antibacterial assay of PEG-carbonate HP 25 alongside non-carbonate version HP 17 demonstrating enhanced antibacterial activities against MRSA.

We synthesized PEG carbonate-HPs **24** and **25** through the initial condensation of triphosgene with tetraethyleneglycol monomethyl ether to generate a PEGylated chloroformate intermediate (not shown), which was immediately condensed with **1** to give **24** in 80% yield and **17** to give **25** in 96% yield (Fig. 5A). Both PEG carbonate-HPs have reduced CLogP values with **24** having a CLogP of 3.56 while **25** has a CLogP of 4.06. These carbonates do not directly bind metal(II) cations in UV-Vis experiments (see next section) and are chemically stable in aqueous formulations for >1 month at room temperature. PEG carbonate-HPs **24** and **25** have maintained or enhanced antibacterial and biofilm eradication activities compared to their phenolic precursors **1** and **17** suggesting improved bacterial cell entry and possible involvement of bacterial esterase enzymes for cleavage of the carbonate group to deliver the active HP once inside bacteria. To support this model, **25** reported MICs of 0.003 and 0.013 µM against MRSA-1707 and MRSA-44 while corresponding HP **17** reported MICs of 0.30 and 0.39 against the same strains correlating to an impressive 100- and 30-fold enhancement of antibacterial activities for HP carbonate **25** compared to (non-carbonate) **17** (Fig. 5B). A further discussion can be found in the structure-activity relationships section. Similar to non-carbonate HPs, **24** and **25** showed no HeLa cell cytotoxicity at 100 µM.

#### Metal(II) Binding (UV-Vis) and Co-Treatment in Antibacterial Assays with MRSA-1707

**Figure 6:**
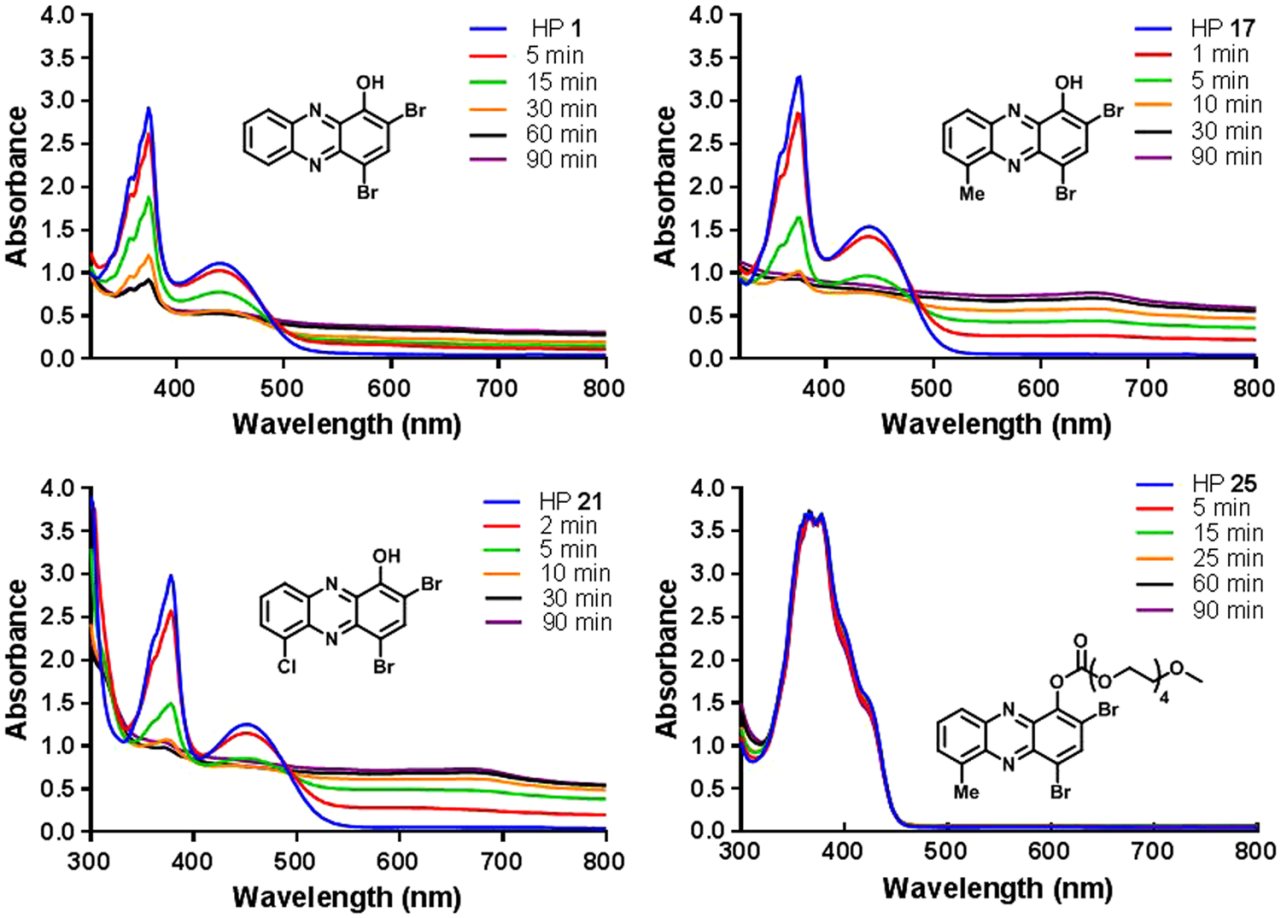
UV-Vis analysis of copper(II) binding various halogenated phenazines. The HP:copper(II) complex is insoluble and precipitates out of solution, thus the disappearance of HP peaks is clear while there is not a strong appearance of HP:copper(II) complex in the UV-Vis spectrum﻿.

We previously reported that HPs directly binds copper(II) and iron(II) cations in UV-Vis experiments and loses antibacterial activities when co-treated with these metal(II) cations in antibacterial assays against MRSA-2 (clinical isolate; Shands Hospital; Gainesville, FL)^25^. HPs bind metal(II) cations through a chelation event involving the oxygen atom of the 1-hydroxyl group and the adjacent nitrogen at the 10-position of the HP scaffold to form a stable 2:1 HP:metal(II) complex. During these investigations, we evaluated the ability of HPs **1**, **17** and **21** to directly bind copper(II), iron(II), zinc(II) and magnesium(II) in UV-Vis experiments and found these HPs to bind copper(II), iron(II) and zinc(II), but not magnesium(II) cations. Based on the kinetics of UV-Vis experiments, HP **17** (6-methyl HP) and **21** (6-chloro HP) chelated copper(II) at a faster rate than HP **1** (Fig. 6), which could explain the enhanced antibacterial activities of **17** and **21** compared to HP **1**. Interestingly, HP **25** does not bind any of the metal(II) cations directly as the carbonate functionality blocks direct metal-chelation.

We found that co-treatment of HPs with metal(II) cations at 200 µM in MIC assays against MRSA BAA-1707 led to reduced antibacterial activities (i.e., 4- to >20,000-fold elevated MIC values with copper(II) cation co-treatment; Table 2) for **1**, **17** and **25** with copper(II) and iron(II); however, it is interesting to note that co-treatment with zinc(II) increased antibacterial activities (reduced MICs; Table 2) of HPs and no changes were observed in the antibacterial activities when HPs were co-treated with magnesium(II). HP **21** showed reduced antibacterial activities against MRSA BAA-1707 in the presence of copper(II) and iron(II); however, no changes in antibacterial activities were observed when **21** was co-treated with zinc(II) or magnesium(II). PEG-carbonate HP **25** gives the most dramatic antibacterial response to co-treatment with metal(II) cations (>20,000-fold elevated MIC against MRSA with copper(II) and 67-fold reduction in MIC against MRSA with zinc(II), yet does not directly bind either metal(II) cation directly) further supporting that PEG-carbonate **25** enters MRSA at a high efficiency, then is converted to the active, metal-chelating HP **17** which elicits a potent antibacterial response. Interestingly, structurally related non-halogenated comparators, 1-hydroxyphenazine (1-OHP) and 8-hydroxyquinoline (8-OHQ), demonstrated drastically different metal(II) cation profiles compared to HPs **1**, **17**, **21** and **25**.

**Table 2:** UV-Vis and metal(II)-cation co-treatment MIC assays summary for HPs against MRSA BAA-1707.

MRSA BAA-1707 (concentration in µM)									
Test Cpd.	MIC	Copper(II)		Iron(II)		Zinc(II)		Magnesium(II)	
		Binding Y/N	MIC w/Cu^2+^	Binding Y/N	MIC w/Fe^2+^	Binding Y/N	MIC w/Zn^2+^	Binding Y/N	MIC w/Mg^2+^
1	1.17^a^	Y	4.69^a^	Y	2.35^a^	Y	0.39	N	1.17^a^
17	0.30^a^	Y	75^a^	Y	12.5	Y	0.05	N	0.30^a^
21	0.10^b^	Y	6.25	Y	0.59^a^	Y	0.15^a^	N	0.15^a^
25	0.0047^a^	N	>100	N	25	N	0.00007^a^	N	0.0047^a^
8-OHQ	9.38	Y	4.69^a^	Y	12.5	Y	12.5	N	6.25
1-OHP	250	—	75	—	250	—	>500	—	250
TPEN	46.9^a^	—	500	—	125	—	250	—	62.5
EDTA	25	—	50	—	50	—	50	—	50
Doxy.	0.78	—	0.78	—	3.13	—	0.39	—	0.78

Note: ^a^Midpoint for a 2-fold range in observed values. ^b^MIC for **21** in these experiments was 0.1 µM. UV-Vis results are reported as “Binding Y/N” for HPs. Each metal(II) cation was tested at 200 µM in co-treatment MIC assays. 8-OHQ (8-hydroxyquinoline) and 1-OHP (1-hydroxyphenazine) were used as positive controls and have metal-binding moieties related to HPs.

The metal-chelating agents EDTA and TPEN, a membrane-permeable metal-chelating agent, were used as comparators in metal(II) cation co-treatment assays. EDTA showed only slight reductions in antibacterial assays (MIC values elevated 2-fold) when co-treated with each metal(II) cation. TPEN showed more dramatic reductions in antibacterial activities (MIC values elevated 3- to 11-fold) with copper(II), iron(II) and zinc(II); however, there was not a significant change in MIC values with TPEN was co-treated with magnesium(II). Doxycycline, a tetracycline antibiotic that chelates a magnesium(II) ion in the bacterial ribosome during protein synthesis inhibition^41^, was also tested in these metal(II) cation co-treatment assays against MRSA BAA-1707 and only iron(II) and zinc(II) modulated antibacterial activities with 4-fold elevated MIC values and 2-fold reduced MIC values, respectively. These studies demonstrate unique antibacterial profiles for HP small molecules with the enhancement of zinc(II) cations being of particular interest. Future studies are aimed to further understand these results in the context of biofilm viability.

#### Biofilm Eradication Studies

From our previous studies, we have found that biofilm eradication activities correlate well to antibacterial activities for HP small molecules^24, 25^. With this in mind, we advanced a focused set of Wohl-Aue derived HP analogues to biofilm eradication assays using Calgary Biofilm Devices (CBD)^42, 43^ containing specialized 96-well plates with a lid containing 96 pegs anchored to the lid to provide a surface for biofilms to form and be treated (one peg per microtiter well). Unlike MIC assays which are used to determine the inhibition of rapidly-dividing planktonic bacteria, CDB assays have three phases to test for biofilm eradication, including: (1) biofilm-attachment/establishment to the CBD peg surface (24 hours, media and bacteria only), (2) treating established biofilms on CBD pegs with test compounds (24 hours, media and test compounds only) and (3) recovery, growth, dispersion and planktonic proliferation of viable biofilms (24 hours, media only). At the end of biofilm eradication assays, turbid microtiter wells in 96-well plates result from pegs that have viable biofilms whereas microtiter wells that results in non-turbid microtiter wells result from pegs containing eradicated biofilms. Using the CBD assay is operationally simple as lid pegs that have attached biofilms are rapidly washed and transferred to new 96-well plates (containing media, with or without test compound) as one moves through each of the three assay phases. Upon completion of biofilm eradication assays, minimum biofilm eradication concentration (MBEC) values are determined as the lowest concentration at which biofilms are completely eradicated. Using the CBD, planktonic-killing activities can also be determined and is useful in our investigations as minimum bactericidal concentrations (MBC) enable us to assess planktonic-biofilm killing dynamics in the same assay using a single culture. We have found that CBD assays provide superior insights into biofilm-eradicating agents compared to obtaining MIC values and MBEC values using different assays.

**Figure 7:**
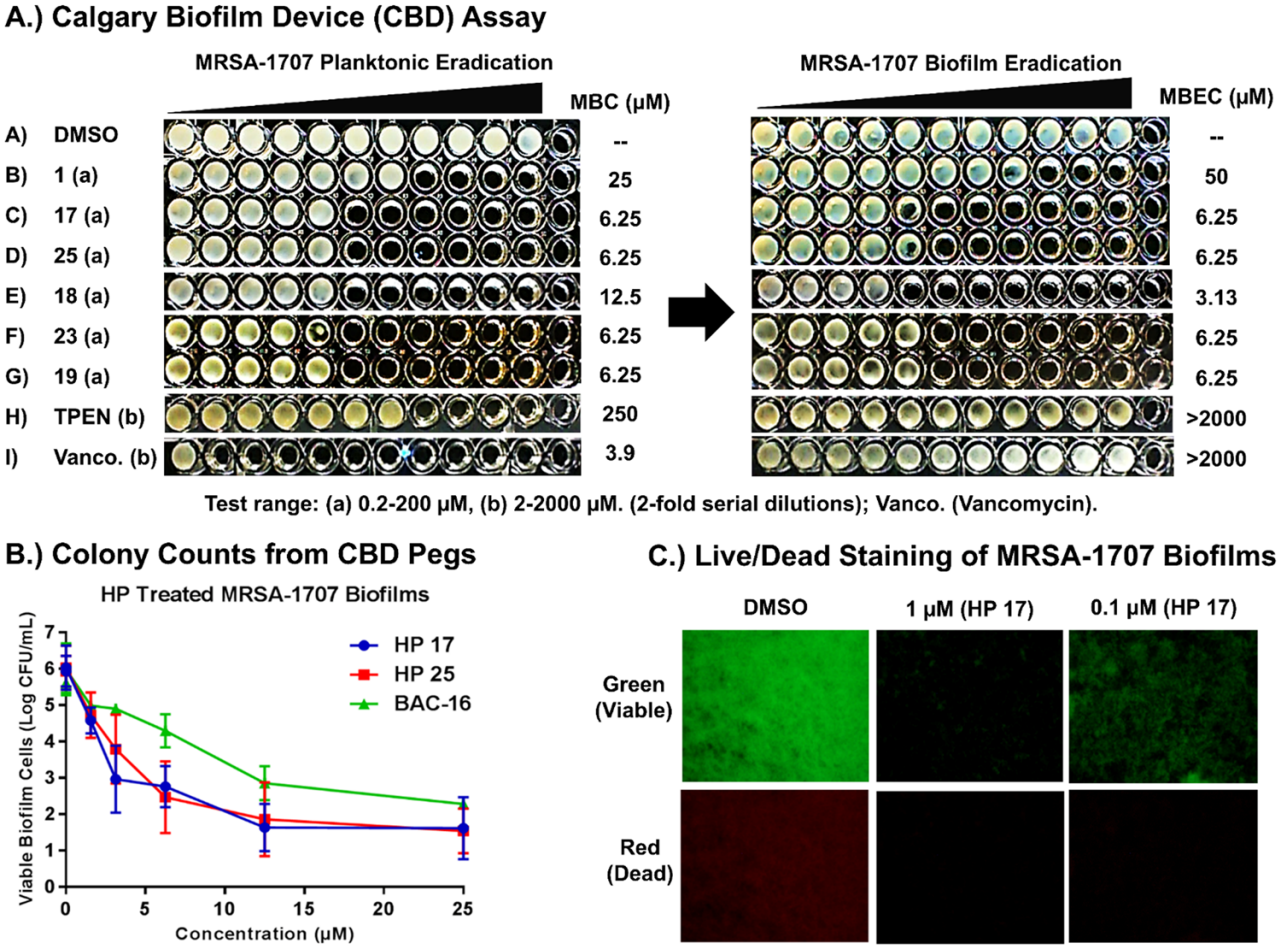
(**A**) Calgary biofilm device (CBD) assay of a panel of HPs, TPEN and vancomycin against MRSA-1707 demonstrating the potent and unique biofilm eradication activities of HP small molecules. (**B**) Dose-response curve of biofilm eradication for HP 17 (MBEC = 4.69 µM), HP 25 (MBEC = 9.38 µM) and BAC-16 (MBEC = 25 µM) against MRSA-1707 (pulse treatment). (**C**) Live/Dead staining (fluorescence images) of established MRSA-1707 biofilms treated with HP 17 after 24 hours﻿.

During these investigations, we evaluated 10 new HP analogues (**4**, **9**, **17–19**, **21–25**) in biofilm eradication assays against MRSA BAA-1707 and found five HPs (**17–19**, **22**, **23**, **25**) to demonstrate potent minimum biofilm eradication concentrations (MBEC) between 4.69 and 37.5 µM (Table 3). Halogenated Phenazines **18** (MBEC = 4.69 µM), **17** (MBEC = 6.25 µM), **19** (MBEC = 9.38 µM), **23** (MBEC = 9.38 µM) and PEG-carbonate **25** (MBEC = 9.38 µM) proved to be the most potent MRSA biofilm-eradicating HPs from this series (Fig. 7A). Similar to our antibacterial findings from MIC analysis, HP analogues **17–19** and **23** possess either a methyl group, ethyl group or bromine atom at the 6-position of the HP scaffold and performed with high potency in biofilm eradication assays.

**Table 3:** Summary of biofilm eradication and hemolysis studies with Halogenated Phenazines.

Compound	MRSA BAA-1707 MIC	MRSA BAA-1707 MBC/MBEC	MRSE 35984 MIC	MRSE 35984 MBC/MBEC	VRE 700221 MIC	VRE 700221 MBC/MBEC	Hemolysis at 200 µM (%)
1	1.17^a^	50^b^/75^a^	2.35^a^	23.5^a^/250	4.69^a^	18.8^a^/9.38^a^	≤1
4	3.13	46.9^a^/93.8^a^	2.35^a^	150^a^/>200	3.13	12.5^b^/4.69^a^	≤1
9	1.17^a^	>200/>200	0.39	12.5^b^/>200	2.35^a^	12.5/9.38^a^	≤1
17	0.30^a^	6.25/6.25	0.30^a^	9.38^a^/4.69^a^	0.59^a^	1.56^b^/0.59^a^	6.7
18	0.10^c^	6.25/4.69^a^	0.10^c^	9.38^a^/2.35^a^	0.15^a^	3.13^b^/0.59^a^	1.7
19	0.15^a^	4.69^a^/9.38^a^	0.30^a^	6.25/4.69^a^	0.15^a^	1.56^b^/0.59^b^	5.3
21	0.08^a^	6.25/75^a^	0.30^a^	18.8^a^/75^a^	1.56	25/25	≤1
22	0.15^a^	18.8^a^/37.5^a^	18.8^a^	50/37.5^a^	1.56	9.38^a^/2.35^a^	≤1
23	0.05	4.69^a^/9.38^a^	0.78	4.69^a^/12.5	1.17^a^	9.38^a^/9.38^a^	5.1
24	0.59^a^	75^a^/100	1.56	75^b^/100	6.25	9.38^a^/4.69^a^	2.4
25	0.003	9.38^a^/9.38^a^	0.15^a^	9.38^a^/4.69^a^	0.59^a^	1.56^b^/0.59^a^	≤1
QAC-10	4.69^a^	93.8^a^/93.8^a^	2.35^a^	31.3/31.3	2.35^a^	3.0^a^/3.0^a^	>99
BAC-16	1.56	31.3^b^/15.6	1.56	—	3.13	5.9^a^/3.0^a^	—
TPEN	46.9^a^	375^a^/>2000	12.5	250/>2000	—	—	≤1
EDTA	25	>2000/>2000	—	1000/>2000	—	>2000/93.8^a^	≤1
Vancomycin	0.39	3.9/>2000	0.78	3.0^b^/>2000	>100	>200/150^a^	—
Daptomycin	3.13	125/>2000	12.5	—	125	375/93.8^a^	—
Linezolid	12.5	31.3/>2000	3.13	—	3.13	4.69^b^/1.56	—

Notes: ^a^Midpoint for a 2-fold range in observed values. ^b^Midpoint value of a 4-fold range in values. ^c^Lowest concentration tested. All biological results summarized in this table were generated from three independent experiments.

In addition to HPs, we assayed various comparators in biofilm eradication assays against MRSA BAA-1707 (Table 3), including membrane-lysing agents (quaternary ammonium cation-10, QAC-10, a reported biofilm eradicating agent^33^; BAC-16), metal-chelating agents (EDTA^30^, TPEN^31, 32^) and conventional antibiotics used in MRSA treatments (i.e., vancomycin, linezolid, daptomycin)^44, 45^. During these studies, our lead HPs **17–19** were found to be 10- to 21-fold more potent than QAC-10 (MBEC = 93.8 µM) against MRSA BAA-1707 biofilms. Interestingly, the metal-chelating agents EDTA and TPEN were both found to be inactive (MBEC > 2,000 µM; Fig. 6A) in biofilm eradication assays against MRSA BAA-1707 when tested alongside HP small molecules. We found this result particularly interesting and we feel the lack of biofilm eradication activity by EDTA and TPEN speaks to the unique mechanism that leads to HP-induced biofilm killing as HP **18** proves to be >425-fold more potent than EDTA or TPEN in CBD assays. Vancomycin, daptomycin and linezolid are front-running MRSA therapies^44, 45^ and are unable to eradicate MRSA BAA-1707 biofilms (MBEC > 2,000 µM) despite being active against planktonic cells in CBD assays (i.e., vancomycin, MBC = 3.9 µM). These findings are illustrative of the abilities of MRSA biofilms to tolerate and thrive in the presence of high concentrations of current antibiotic therapies.

We previously demonstrated one HP analogue slowly kills MRSA persister cells in kinetic kill experiments of stationary cultures^24^, which are known to contain higher populations of non-replicating persister cells^46, 47^. We were curious to see if the biofilm eradication activities of HPs would show a more potent effect if MRSA biofilms were pulse treated with active HPs (i.e., two subsequent 24 hour compound treatment phases). We tested HPs **17** and **25** alongside BAC-16, a membrane-lysing comparator, in pulse experiments where after the 24-hour compound treatment phase, CBD lids were transferred to a second 96-well plate (extending phase 2 of the biofilm eradication assay) with the same test compound concentrations to treat MRSA biofilms for an additional 24 hours before transferring to the recovery plate (phase 3). The results from the pulse biofilm eradication experiments showed only slight improvements in biofilm eradication activities for HP **17** (MBEC = 4.69 µM) while HP **25** reported the same potency as in standard CBD assay conditions (MBEC = 9.38 µM) against MRSA-1707. BAC-16 reported an MBEC of 25 µM in pulse experiments against MRSA-1707. Following the completion of the pulse experiments, we removed treated and untreated pegs from the CBD to determine viable biofilm cell counts following sonication and plating to generate the dose-response curve in Fig. 7B. At the 6.25 µM, we found that **17** and **25** reduced viable MRSA-1707 biofilm cells by 3-logs (99.9% biofilm eradication) whereas at 25 µM these HPs reduced viable MRSA-1707 biofilm cells by >4-logs (>99.99% biofilm eradication).

In addition to the CBD assays, we tested HP **17** against MRSA-1707 biofilms in live/dead staining experiments. Following a 24-hour MRSA-1707 biofilm establishment phase, HP **17** was added at 0.1, 1 and 5 µM and then allowed to incubate at 37 °C for 24 hours. After this time, images of the treated and untreated MRSA-1707 biofilms were obtained using fluorescence microscopy (Fig. 7C). As expected, HP **17** demonstrated a potent biofilm eradication and clearance response towards MRSA-1707 biofilms, even at the lowest concentration tested of 0.1 µM.

Our panel of 10 new HP small molecules also demonstrated impressive biofilm eradication activities against methicillin-resistant *S*. *epidermidis* (MRSE ATCC 35984) and vancomycin-resistant *Enterococcus faecium* (VRE ATCC 700221) biofilms in CBD assays. HP **18** the most potent biofilm eradication activities against MRSE biofilms (MBEC = 2.35 µM) and VRE biofilms (MBEC = 0.59 µM). Similar biofilm eradication profiles were observed for HPs **17** and **19** as both HPs reported MBECs = 4.69 µM against MRSE and MBECs = 0.59 µM against VRE (Table 3). In addition, we found HPs to be more active against VRE biofilms (MBEC = 0.59–9.38 µM) than MRSE biofilms (MBEC = 2.35–75 µM), and similar to our findings against MRSA-1707 biofilms, HPs out-performed all conventional antibiotics (e.g. vancomycin), metal-chelating agents (e.g., EDTA) and membrane-lysing agents (e.g., QAC-10, BAC-16). The remarkable biofilm eradication activity possessed by these new HPs could lead to ground-breaking advances in treating persistent, biofilm-associated infections.

#### Red Blood Cell Hemolysis

Following biofilm eradication studies, we assayed our HPs for hemolysis activity against red blood cells (RBCs) at a single high concentration (200 µM). Antimicrobial peptides and mimics thereof (i.e., quaternary ammonium cationic compounds) eradicate biofilm cells through membrane lysis; however, identifying potent membrane-lysing agents that target bacterial membranes over mammalian membranes has proven challenging^33, 48, 49^. Our Wohl-Aue derived HPs demonstrated little, if any, hemolytic activity against RBCs at 200 µM (≤1 to ≤7% hemolysis, Table 3). HP **18**, the most potent biofilm-eradicating agent from this collection, demonstrated 1.7% hemolysis at 200 µM while completely eradicating biofilms at 85- to 339-fold lower concentrations (MBEC = 0.59–2.35 µM) compared to membrane-lysing QAC-10 (MBEC = 93.8 µM) that demonstrated >99% lysis of RBCs at 200 µM when tested alongside HPs. The potent biofilm eradication activities of HPs together with the lack of RBC lysis and HeLa cytotoxicity demonstrates a unique targeting ability of these HP small molecules which could lead to significant breakthroughs in the treatment of persistent, biofilm-associated bacterial infections in the clinic.

### Structure-Activity Relationships

**Figure 8:**
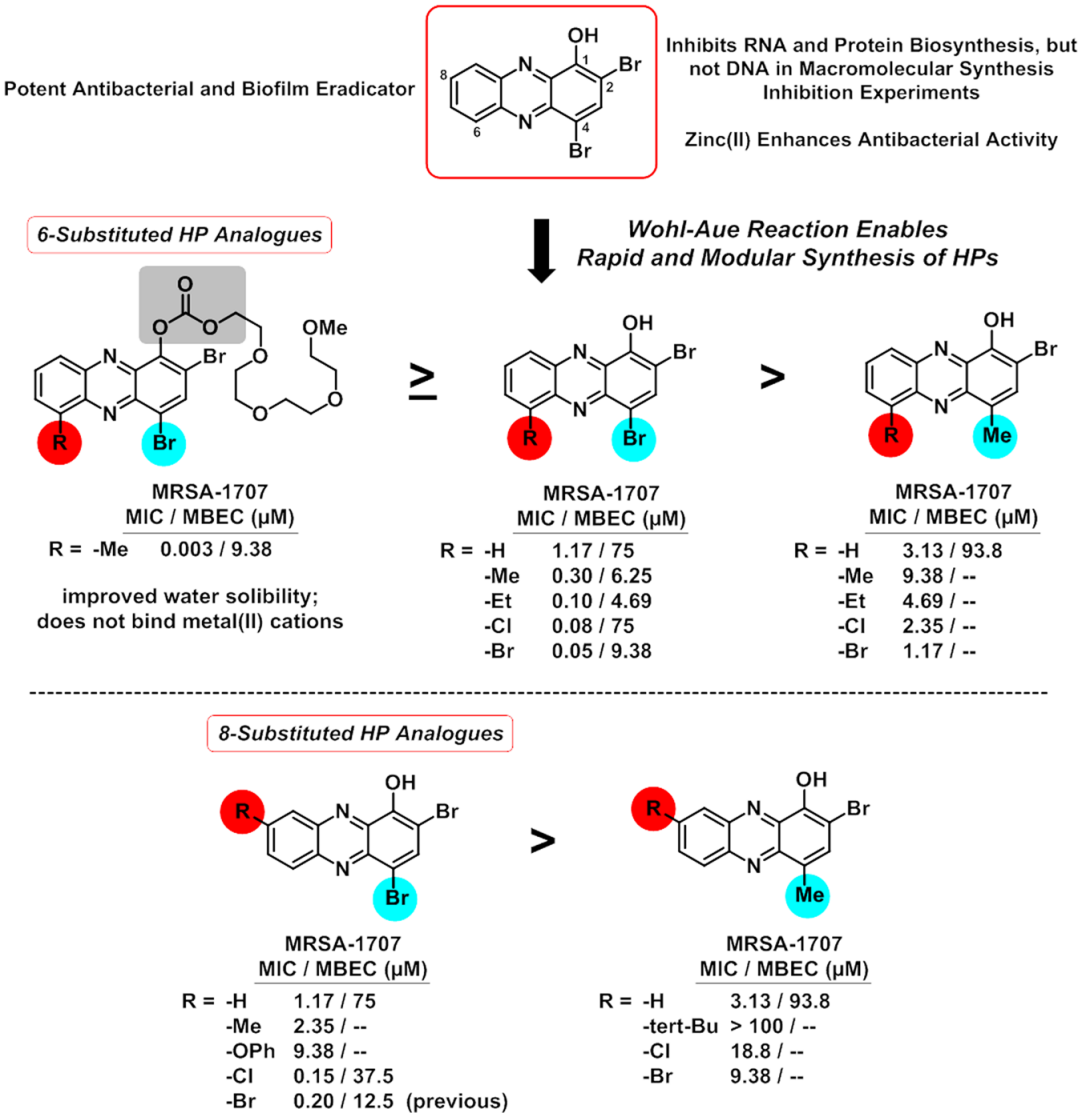
New activity profiles focused heavily on 4-, 6- and 8-substituted HPs during these studies﻿.

Based on our previous studies, we have found that the HP scaffold **1** requires the 1-hydroxyl group and 2-bromine atom to demonstrate antibacterial activities^22, 25^. In addition, the 4-position is highly active when containing a bromine atom (i.e., HP **1**), but can also tolerate a butyl group (i.e., HP **2**)^25^ which motivated these investigations as Wohl-Aue reactions could provide synthetic avenues to both 4-brominated and 4-methylated HPs. During these studies, we found the 4-bromo HPs to be significantly more active than 4-methyl HP analogues, which could result from enhanced bacterial membrane permeability or phenolic acidity, which may be critical for metal-chelation. This SAR profile is observed against both planktonic and biofilm cells. To illustrate, the 4-bromo HP analogue, 4-bromo-6,7-dimethyl HP **19** demonstrates 12-fold more potent antibacterial activities and >21-fold more potent biofilm eradication activities against MRSA-1707 compared to it’s 4-methyl HP analogue, 4,6,7-trimethyl HP **7** (Fig. 8).

This study is the first to report HP analogues with substitutions at the 6-position, which proved to significantly enhance antibacterial activities of the HP scaffold as 6-methyl (**17**), 6,7-dimethyl (**19**), 6-ethyl (**18**), 6-chloro (**21**) and 6-bromo (**23**) all demonstrated potent antibacterial and biofilm eradication activities compared to HP **1** (Fig. 7). In addition, we previously reported potent antibacterial activities of 7,8-disubstituted HP analogues^24, 25^ and here, we report our investigations of three new 8-monosubstutited HP analogues, including: 8-phenoxyether HP (**16**), 8-methyl HP (**20**) and 8-chloro HP (**22**). Both 8-phenoxyether HP (**16**) and 8-methyl HP demonstrated a 2- to 24-fold loss in antibacterial activities against staphylococcal pathogens (MRSA, MRSE) compared to HP **1**; however, both **16** and **18** reported a 2-fold increase in antibacterial activities against VRE. Interestingly, 8-chloro HP **22** demonstrated a 5- to 8-fold increase in antibacterial activities against four MRSA isolates (Table 1) while losing 5- to 8-fold in antibacterial activities against both *S*. *epidermidis* strains compared to HP **1**. In addition, HP **22** demonstrated improved biofilm eradication activities against MRSA, MRSE and VRE biofilms compared to HP **1** and equipotent activities compared to the 7,8-dichloro HP analogue (not shown), which we previously described; however, 8-chloro HP **22** was less potent than the 6-substituted series of HP analogues. Similar to the antibacterial profiles of HP **16** and **18**, HP **22** showed an increased potency against VRE (4-fold) compared to HP **1**.

Understanding the significance of each of the functional group substitutions and combinations of multiple substitutions on positions 6–9 of the HP scaffold will require additional investigations, which are underway in our labs. However, substitutions in these positions clearly control antibacterial activities and one can propose that these compounds are targeting one or more bacterial metalloproteins that are critical to biofilm viability. We believe that metalloprotein targeting is a viable notion as our HP small molecules generate different activity profiles when treated with our panel of metal(II) cations compared to EDTA and TPEN, which are known to be involved in general metal(II) sequestration. Following this line of thought, the 6-position of the HP scaffold would likely not interfere with a HP-metal binding event in a metalloprotein due to it’s distal location in relation to the metal-binding moiety of the HP scaffold. The observed improvement in antibacterial potency of 6-position HP analogues could be due to more rapid metal-binding kinetics, which we demonstrated during these investigations (Fig. 6). Similar activities may be explained by substitution at the 7-position and 8-halogenated HPs; however, 8-methyl HP **23** and 8-phenoxy HP **15** lose activity compared to unsubstituted HP **1**, potentially due to disfavored interactions in their respective target(s) or decreased bacterial membrane penetration. It is also possible that the halogens in the 6–8 positions of the HP scaffold enhance bacterial membrane permeability compared to alkyl or ether groups, leading to more efficient bacteria entry and metalloprotein targeting. The only 9-substituted HP analogue synthesized during these studies was HP **15**, which proved to be inactive against all strains tested against (MIC > 50 µM). Additional 9-position analogues are needed to confirm this initial result; however, this position may impede HP-target interactions at a metal(II) center in a metalloprotein. This collective series of HPs enable us to begin outlining proposals regarding potential bacterial targets and key interactions, which will guide future developments as we advance our mechanistic studies (probe design) and pre-clinical evaluations (therapeutic leads). Ultimately, these analogues have enabled us to ask interesting questions about biofilm killing that we are set to answer in future studies.

The synthetic route developed here does not enable for an ideal synthesis of 7- or 9-monosubstituted HP analogues due to regioisomers that would result from condensing 3-substituted anilines with 2-nitroanisole in the Wohl-Aue reaction. Currently, we are developing synthetic routes to explore such analogues and will report our findings accordingly.

In addition to our significant findings regarding the 6–8 positions of the HP scaffold, we report that the phenolic hydroxyl group can tolerate a PEG-carbonate and show enhanced antibacterial activities towards multiple MRSA isolates and maintain biofilm eradication activities against MRSA-1707 compared to their non-carbonate analogues. In previous studies, we found that some ester groups are well-tolerated while ether- or amine-group substitution of the phenolic hydroxyl group leads to a complete loss of antibacterial activities^22^. The structural requirement for a 1-hydroxyl group (or masked ester/carbonate) on the HP scaffold, coupled with the results from UV-Vis experiments and metal(II) co-treatment MIC profiles, leads us to conclude that non-metal(II) binding carbonates **24** and **25** are prodrugs that release metal(II)-chelators HP **1** and **17**, which bind metal(II)-cations and eradicate planktonic and biofilm cells against susceptible, Gram-positive human pathogens. Continued efforts regarding prodrug investigations are currently underway in our labs and could lead to significant advances in the treatment of bacterial infections.

**Figure 9:**
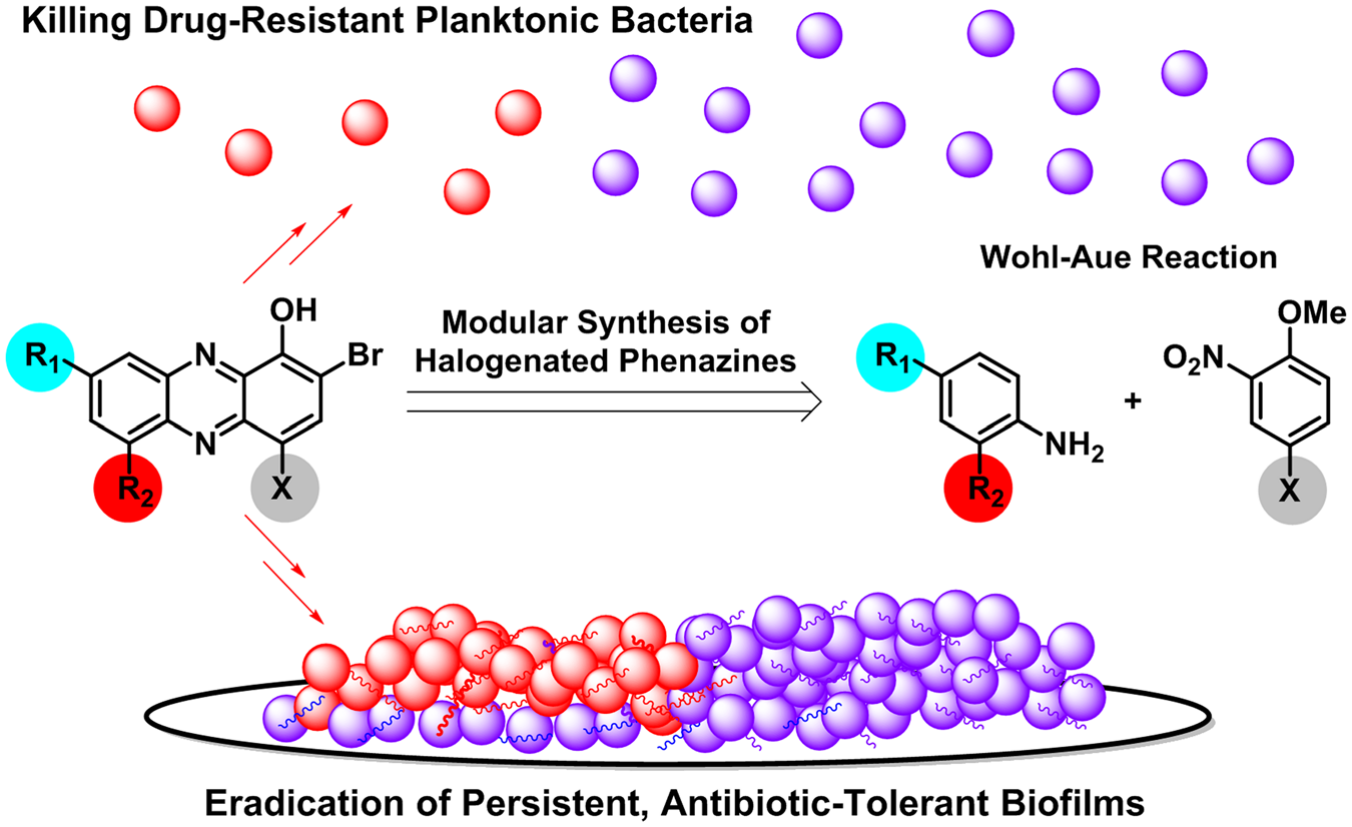
Development of a modular synthesis to a diverse array of halogenated phenazines that target both planktonic and biofilms cells﻿.

In conclusion, we have utilized a convergent Wohl-Aue reaction to synthesize a diverse class of halogenated phenazine small molecules that have enabled exploration of the 4-, 6- and 8-positions of the HP scaffold using readily available building blocks (Fig. 9). From these studies, we have discovered that 6-substituted HPs demonstrate enhanced antibacterial and biofilm eradication activities against MRSA, MRSE and VRE compared to parent HP **1**. We also found that the antibacterial activities of HPs are enhanced with zinc(II) co-treatment, yet are inhibited with copper(II) and iron(II). This metal(II) co-treatment profile differs from known metal-chelating agents EDTA and TPEN, which were unable to eradicate MRSA and MRSE biofilms during these investigations. In macromolecular synthesis inhibition experiments, we found HP **1** to inhibit RNA and protein biosynthesis while not inhibiting DNA synthesis in planktonic cultures of MRSA-1707. This is also our first report of a 2,4-dibrominated HP (8-chloro HP, **18**) demonstrating good antibacterial activity against MtB. In addition, we synthesized a PEG-carbonate HP with enhanced water solubility that demonstrated 30- to 100-fold enhancement of antibacterial activities against MRSA strains, likely through a prodrug mechanism, which could prove essential for translational aspects of HP small molecules. These findings, taken together with the low cytotoxicity and lack of hemolysis activity, give promise that HP small molecules could lead to significant breakthroughs in the treatment of persistent, biofilm-associated bacterial infections.

## Methods

### General Information

All reagents for chemical synthesis were purchased from commercial sources and used without further purification. Reagents were purchased at ≥95% purity and commercially available controls were used in our biological investigations without further purification. All microwave reactions were carried out in sealed tubes in an Anton Paar Monowave 300 Microwave Synthesis Reactor. A constant power was applied to ensure reproducibility. Temperature control was automated via IR sensor and all indicated temperatures correspond to the maximal temperature reached during each experiment. Analytical thin layer chromatography (TLC) was performed using 250 μm Silica Gel 60 F254 pre-coated plates (EMD Chemicals Inc.). Flash column chromatography was performed using 230–400 Mesh 60 Å Silica Gel from Sorbent Technologies. All melting points were obtained, uncorrected, using a Mel-Temp capillary melting point apparatus from Laboratory Services, Inc.

Bacterial strains used during these investigations include: methicillin-resistant *Staphylococcus aureus* (Clinical Isolate from Shands Hospital in Gainesville, FL: MRSA-2; ATCC strains: BAA-1707, BAA-44), methicillin-resistant *Staphylococcus epidermidis* (MRSE, ATCC 35984; ATCC 12228), Vancomycin-resistant *Enterococcus* (VRE, ATCC 700221) and *M*. *tuberculosis* H37Ra (ATCC 25177). Compounds were stored as DMSO stocks at room temperature in the absence of light when they are stable in DMSO stock without observing any loss in biological activity for several months at a time. To ensure compound integrity of our DMSO stock solutions, we did not subject these DMSO stocks of our test compounds to multiple freeze-thaw cycles.

### General Chemical Synthesis Procedures

#### Wohl-Aue reaction

To a 100 mL round-bottom flask was added 4-*tert*-butylaniline (1.60 mL, 10.0 mmol), 4-methyl-2-nitroanisole (1.53 mL, 11.0 mmol), and potassium hydroxide (2.80 g, 50.0 mmol) in toluene (16 mL). The reaction was then allowed to reflux for 10 hours. After the reaction was complete, the resulting mixture was then transferred to a separatory funnel with brine and extracted with dichloromethane (20 mL × 3). The organic layers were combined, filtered and concentrated *in vacuo*. The resulting crude solid was purified via column chromatography using 99:1 to 85:15 hexanes:ethyl acetate to afford yellow solid 8-*tert*-butyl-4-methyl-1-methoxyphenazine (612 mg, 22%; compound **30** in supporting information, precursor to HP **8**).

#### Demethylation of 1-methoxyphenazines

To a round bottom flask, 6-methyl-1-methoxyphenazine (376 mg, 1.68 mmol) was dissolved in anhydrous dichloromethane (50 mL) and cooled to −78 °C before dropwise addition of 1 M boron tribromide solution in dichloromethane (10.0 mL, 10.0 mmol). The reaction was left to stir at −78 °C for 1 hour, and allowed to reach ambient temperature overnight. The reaction was then heated to reflux for 8 hours until complete (monitored by TLC). Upon completion of the reaction, brine (50 mL) was added to quench the reaction. The contents of the resulting biphasic mixture were then transferred to a separatory funnel and dichloromethane was used to extract the product. The resulting organic layers were dried with sodium sulfate, filtered through cotton, and removed *in vacuo*. The resulting solid was purified via column chromatography using dichloromethane to elute 6-methyl-1-hydroxyphenazine as a yellow solid (100%, 350 mg). Note: Some 1-hydroxyphenazines were purified with the addition of 1% acetic acid to 99% dichloromethane via column chromatography.

#### Bromination of 1-hydroxyphenazines

6-Methyl-1-hydroxyphenazine (156 mg, 0.742 mmol) and *N*-bromosuccinimide (277 mg, 1.56 mmol) were dissolved in dichloromethane (60 mL) and allowed to stir at room temperature for 4 hours. The reaction contents were washed with brine (60 mL) and extracted with dichloromethane. The extracts were dried with sodium sulfate, filtered, and concentrated *in vacuo*. The resulting solid was purified via column chromatography using 99:1 dichloromethane:acetic acid to elute 6-methyl-2,4-dibromo-1-hydroxyphenazine **17** as a yellow solid. Note: 1.0 Equivalent of *N*-bromosuccinimide was used to synthesize 4-methyl HP analogues.

#### Synthesis of PEG-carbonate HPs

Tetraethyleneglycol monomethyl ether (69 µL 0.33 mmol) was placed in an oven-dried round-bottomed flask and dissolved in anhydrous dichloromethane (1 mL). The solution was then cooled to 0 °C. Pyridine (37 µL, 0.47 mmol) and triethylamine (11 µL 0.73 mmol) was then added via syringe, followed by triphosgene (48.2 mg, 0.16 mmol) dissolved in dichloromethane (1 mL). The resulting mixture was stirred from 0 °C to room temperature and continued to stir at room temperature for 5 hours. After that, then the reaction was cooled to 0 °C before the addition of solution of **17** (86 mg, 0.23 mmol) and triethylamine (49 µL 0.35 mmol) in anhydrous dichloromethane was added to the reaction in dropwise. The reaction solution was stirred for 5 min at 0 °C and then reach ambient temperature and stirred at room temperature overnight. After the reaction was complete, the reaction mixture was poured into a separatory funnel containing 1 M ammonium chloride (20 mL), and the biphasic mixture was shaken vigorously. Upon separation of layers, the aqueous layer was re-extracted with dichloromethane (2 × 30 mL). Organic extracts were collected, dried over Sodium Sulfate, filtered, and concentrated under vacuum. The resulting crude material was purified using flash column chromatography with 3:1 hexanes:ethyl acetate to 100% ethyl acetate as eluent yield **25** as a yellow oil (135 mg, 96%).

### Biology and UV-Vis Experimental Procedures

#### Minimum Inhibitory Concentration (MIC) Susceptibility Assays

The minimum inhibitory concentration (MIC) for each test compound was determined by the broth microdilution method as recommended by the Clinical and Laboratory Standards Institute (CLSI). In a 96-well plate, eleven two-fold serial dilutions of each compound were made in a final volume of 100 μL Luria Broth. Each well was inoculated with ~10^5^ bacterial cells at the initial time of incubation, prepared from a fresh log phase culture (OD_600_ of 0.5 to 1.0 depending on bacterial strain). The MIC was defined as the lowest concentration of compound that prevented bacterial growth after incubating 16 to 18 hours at 37 °C (MIC values were supported by spectrophotometric readings at OD_600_). The concentration range tested for each test compound during this study was 0.10 to 100 μM. DMSO served as our vehicle and negative control in each microdilution MIC assay. DMSO was serially diluted with a top concentration of 1% v/v. All compounds were tested in a minimum of three independent experiments. NOTE: Metal(II) cation studies were performed in a similar setup to the standard MIC assay, with the addition of 200 µM of the metal(II) cation (i.e., copper(II) sulfate) to the media. All data were obtained from three independent experiments.

#### MIC Assay for Mycobacterium tuberculosis

*M*. *tuberculosis* H37Ra (ATCC 25177) was inoculated in 10 mL Middlebrook 7H9 medium and allowed to grow for two weeks. The culture was then diluted with fresh medium until an OD_600_ of 0.01 was reached. Aliquots of 200 µL were then added to each well of a 96-well plate starting from the second column. Test compounds were dissolved in DMSO at final concentration of 10 mM. 7.5 µL of each compound along with DMSO (negative control) and streptomycin (positive control-40 mg/ml stock solution) were added to 1.5 mL of the Mycobacterium diluted cultures, resulting in 50 µM final concentration of each halogenated phenazine analogues and 340 µM for streptomycin. The final DMSO concentration was maintained at 0.5%. Aliquots of 400 µl were added to wells of the first column of the 96-well plate and serially diluted two-fold (200 µl) per well across the plate to obtain final concentrations that ranges from 0.024 to 50 µM for the test compounds and 0.16 to 340 µM for streptomycin. Three rows were reserved for each compound. The plates were then incubated at 37 °C for seven days. Minimum inhibitory concentrations are reported as the lowest concentration at which no bacterial growth was observed. OD_600_ absorbance was recorded using SpectraMax M5 (Molecular Devices). Data obtained from three independent experiments were analyzed using Excel.

#### Macromolecular Synthesis Inhibition Assays

Macromolecular syntheses experiments were carried out in methicillin-resistant *Staphylococcus aureus* BAA-1707. An overnight culture (100 µL) of *S*. *aureus* BAA-1707 was sub-cultured into 10 mL of fresh TSBG media which was allowed to grow to exponential phase (OD_600_ = 0.2–0.3) before transferring 500 µL to each well in a 24 well-plate. The test compounds and vehicle control (DMSO) were added to achieve the desired concentrations relative to their MIC values against *S*. *aureus* BAA-1707. Treated cultures were then incubated at 37 °C for 30 minutes before radioactive precursors for DNA ([^3^H] thymidine (0.5 µCi)), RNA ([^3^H] uridine (0.5 µCi)) and protein ([^3^H] leucine (1 µCi)) were added. Antibiotics with known modes of action were used as positive controls in these experiments, these included: ciprofloxacin (DNA inhibition), rifampicin (RNA inhibition) and linezolid (protein inhibition). DMSO served as our negative control. DNA and RNA radiolabeled cultures were then incubated in 37 °C for 15 minutes before being stopped by adding 60 µL of cold 5% trichloroacetic acid (TCA). The protein synthesis experiment was stopped after 40 minutes by adding 60 µL cold TCA. These mixtures were then incubated at 2 °C for at least 30 minutes before the contents of the plates were transferred onto glass microfiber filters (24 mm) and washed 5 times with 1 mL of 5% TCA. The filters are allowed to dry overnight before 3.5 mL of the scintillation fluid was added to the scintillation vials containing the filters and the radiation counts were measured using liquid scintillation LS 6500.

#### UV-Vis Experiments

The rates of halogenated phenazine-copper(II) complex formation were independently evaluated via UV-Vis spectrometry following addition of 0.5 equivalents copper(II) sulfate to stirring solutions of HP (10 mM, 4 mL) in dimethyl sulfoxide. Spectral scanning was performed from 200 to 800 nm in 2 nm increments. The disappearance of HPs **1**, **17** and **22** was observed over the indicated time points. The halogenated phenazine-copper(II) complex formation yielded a loss in absorbance due to precipitation. No change of the UV-Vis spectra was observed for **25** as a result of no metal(II) binding.

#### Calgary Biofilm Device (CBD) Experiments to Determine Minimum Bactericidal Concentrations (MBC) and Minimum Biofilm Eradication Concentrations (MBEC)

Biofilm eradication experiments were performed using the Calgary Biofilm Device to determine MBC/MBEC values for various compounds of interest (Innovotech, product code: 19111). The Calgary device (96-well plate with lid containing pegs to establish biofilms on) was inoculated with 125 µL of a mid-log phase culture diluted 1,000-fold in tryptic soy broth with 0.5% glucose (TSBG) to establish bacterial biofilms after incubation at 37 °C for 24 hours. The lid of the Calgary device was then removed, washed and transferred to another 96-well plate containing 2-fold serial dilutions of the test compounds (the “challenge plate”). The total volume of media with compound in each well in the challenge plate is 150 µL. The Calgary device was then incubated at 37 °C for 24 hours. The lid was then removed from the challenge plate and MBC/MBEC values were determined using different experimental pathways. To determine MBC values, 20 µL of the challenge plate was transferred into a fresh 96-well plate containing 180 µL TSBG and incubated overnight at 37 °C. The MBC values were determined as the concentration giving a lack of visible bacterial growth (i.e., turbidity). For determination of MBEC values, the Calgary device lid (with attached pegs/treated biofilms) was transferred to a new 96-well plate containing 150 µL of fresh TSBG media in each well and incubated for 24 hours at 37 °C to allow viable biofilms to grow and disperse resulting in turbidity after the incubation period. MBEC values were determined as the lowest test concentration that resulted in eradicated biofilm (i.e., wells that had no turbidity after final incubation period). All data were obtained from a minimum of three independent experiments. Note: Pulse experiments followed a normal CBD assay protocol; however, the compound treatment phase (the “challenge plate”) consisted of two sequential 24 hour compound treatment plates before the final recovery plate. Following this, CBD pegs were removed from the lid, sonicated for 30 minutes in PBS and plated out to determine biofilm cell killing in colony forming units per milliliter (CFU/mL).

#### Live/Dead staining (Fluorescence Microscopy) of MRSA BAA-1707 Biofilms

A mid-log culture of MRSA BAA-1707 was diluted 1:1,000-fold and 500 µL was transferred to each compartment of a 4 compartment CELLview dish (Greiner Bio-One 627871). The dish was then incubated for 24 hours at 37 °C. After this time, the cultures were removed and the plate was washed with 0.9% saline. The dish was then treated with the compounds in fresh media at various concentrations. DMSO was used as our negative control in this assay. The dish was incubated with the compound for 24 hours at 37 °C. After this time, the cultures were removed and the dish was washed with 0.9% saline for 2 minutes. Saline was then removed and 500 µL of the stain (Live/Dead BacLight Viability Kit, Invitrogen) were added for 15 minutes and left in the dark. After this time, the stain was removed and the dish was washed twice with 0.9% saline. Then the dish was fixed with 500 µL 4% paraformaldehyde in PBS for 30 minutes. Images of remaining MRSA biofilms were then taken with a fluorescence microscope. All data were analyzed using ImageJ software from three independent experiments.

#### LDH Release Assay for HeLa Cytotoxicity Assessment

HeLa cytotoxicity was assessed using the LDH release assay described by CytoTox96 (Promega G1780). HeLa cells were grown in Dulbecco’s Modified Eagle Medium (DMEM; Gibco) supplemented with 10% Fetal Bovine Serum (FBS) at 37 °C with 5% CO_2_. When the HeLa cultures exhibited 70–80% confluence, halogenated phenazines were then diluted by DMEM (10% FBS) at concentrations of 25, 50 and 100 µM and added to HeLa cells. Triton X-100 (at 2% v/v) was used as the positive control for maximum lactate dehydrogenate (LDH) activity in this assay (i.e., complete cell death) while “medium only” lanes served as negative control lanes (i.e., no cell death). DMSO was used as our vehicle control. HeLa cells were treated with compounds for 24 hours and then 50 µL of the supernatant was transferred into a fresh 96-well plate where 50 µL of the reaction mixture was added to the 96-well plate and incubated at room temperature for 30 minutes. Finally, Stop Solution (50 µL) was added to the incubating plates and the absorbance was measured at 490 nm. Results are on the next page and are from three independent experiments.

#### Hemolysis Assay with Red Blood Cells

As previously described, freshly drawn human red blood cells (hRBC with ethylenediaminetetraacetic acid (EDTA) as an anticoagulant) were washed with Tris-buffered saline (0.01 M Tris-base, 0.155 M sodium chloride (NaCl), pH 7.2) and centrifuged for 5 minutes at 3,500 rpm. The washing was repeated three times with the buffer. In 96-well plate, test compounds were added to the buffer from DMSO stocks. Then 2% hRBCs (50 µL) in buffer were added to test compounds to give a final concentration of 200 µM. The plate was then incubated for 1 hour at 37 °C. After incubation, the plate was centrifuged for 5 minutes at 3,500 rpm. Then 80 µL of the supernatant was transferred to another 96-well plate and the optical density (OD) was read at 405 nm. DMSO served as our negative control (0% hemolysis) while Triton X served as our positive control (100% hemolysis). The percent of hemolysis was calculated as (OD_405_ of the compound- OD_405_ DMSO)/(OD_405_ Triton X- OD_405_ buffer) from three independent experiments.

### Associated Content

General synthesis procedures; antibacterial, HeLa cell cytotoxicity, hemolysis assay protocols; full characterization data reported for all new compounds, including: H/ C NMR spectra, HRMS and melting points (for solids).

## Electronic supplementary material

Supplementary Info
