# Supplementary material for: A Highly Potent Class of Halogenated Phenazine Antibacterial and Biofilm-Eradicating Agents Accessed Through a Modular Wohl-Aue Synthesis
Source: Sci Rep. 2017 May 17;7:2003. doi: 10.1038/s41598-017-01045-3 (PMC5435703; doi:10.1038/s41598-017-01045-3)
Supplement: Supplementary file 1 — Supplementary Info [file 41598_2017_1045_MOESM1_ESM.pdf]

A Highly Potent Class of Halogenated Phenazine Antibacterial and Biofilm-Eradicating Agents Accessed Through a Modular Wohl-Aue Synthesis

Hongfen Yang†, Yasmeen Abouelhassan†, Gena M. Burch†, Dimitris Kallifidas†, Guangtao Huang‡, Hussain Yousaf†, Shouguang Jin‡, Hendrik Luesch†, Robert W. Huigens III†*

†Department of Medicinal Chemistry, Center for Natural Products, Drug Discovery and Development (CNPD3), College of Pharmacy, University of Florida, Gainesville, Florida 32610; ‡Department of Molecular Genetics & Microbiology, College of Medicine, University of Florida

*Corresponding Author: rhuigens@cop.ufl.edu

Supporting Information

1.) General Information **S2**

2.) Synthetic Procedures and Characterization Data **S3**

3.) Biological Methods and UV-Vis Experiments **S19**

4.) Literature References **S26**

5.) Supporting Images of Biological Experiments **S27**

6.) ^{1}H NMR & ^{13}C NMR Spectra for New Compounds **NMR-1**

1.) General Information:

All reagents for chemical synthesis were purchased from commercial sources and used without further purification. Reagents were purchased at ≥ 95% purity and commercially available controls were used in our biological investigations without further purification. All microwave reactions were carried out in sealed tubes in an Anton Paar Monowave 300 Microwave Synthesis Reactor. A constant power was applied to ensure reproducibility. Temperature control was automated via IR sensor and all indicated temperatures correspond to the maximal temperature reached during each experiment. Analytical thin layer chromatography (TLC) was performed using 250 µm Silica Gel 60 F254 pre-coated plates (EMD Chemicals Inc.). Flash column chromatography was performed using 230-400 Mesh 60Å Silica Gel from Sorbent Technologies. All melting points were obtained, uncorrected, using a Mel-Temp capillary melting point apparatus from Laboratory Services, Inc.

NMR experiments were recorded using broadband probes on a Varian Mercury-Plus-400 spectrometer via VNMR-J software (400 MHz for ^{1}H and 100 MHz for ^{13}C). All spectra are presented using MestReNova 11.0 (Mnova) software and are displayed without the use of the signal suppression function. Spectra were obtained in the following solvents (reference peaks also included for ^{1}H and ^{13}C NMRs): $CDCl_3$ (^{1}H NMR: 7.26 ppm; ^{13}C NMR: 77.23 ppm) and d_6-DMSO (^{1}H NMR: 2.50 ppm; ^{13}C NMR: 39.52 ppm). All NMR experiments were performed at room temperature. Chemical shift values (δ) are reported in parts per million (ppm) for all ^{1}H NMR and ^{13}C NMR spectra. ^{1}H NMR multiplicities are reported as: br = broad, s = singlet, d = doublet, t = triplet, q = quartet, m = multiplet. High-Resolution Mass Spectrometry (HRMS) were obtained for all new compounds from the Chemistry Department at the University of Florida.

Bacterial strains used during these investigations include: methicillin-resistant *Staphylococcus aureus* (Clinical Isolate from Shands Hospital in Gainesville, FL: MRSA-2; ATCC strains: BAA-1707, BAA-44), methicillin-resistant *Staphylococcus epidermidis* (MRSE, ATCC 35984), *S. epidermidis* (ATCC 12228), Vancomycin-resistant *Enterococcus faecium* (VRE, ATCC 700221) and *Mycobacterium tuberculosis* H37Ra (ATCC 25177). Compounds were stored as DMSO stocks at room temperature in the absence of light when they are stable in DMSO stock without observing any loss in biological activity for several months at a time. To ensure compound integrity of our DMSO stock solutions, we did not subject these DMSO stocks of our test compounds to multiple freeze-thaw cycles.

2.) Synthetic Procedures and Characterization Data:

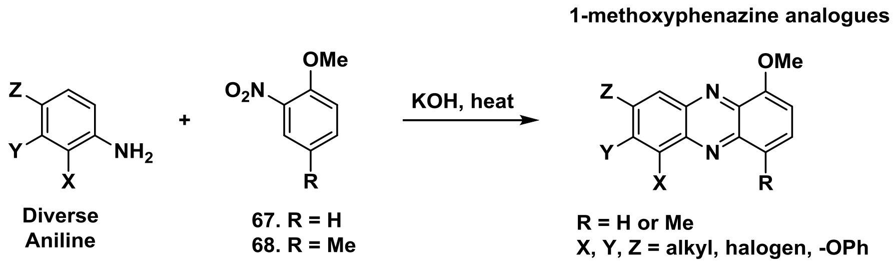

General procedure of the Wohl-Aue reaction for the synthesis of 26-45: To a 100 mL round-bottom flask was added 4-*tert*-butylaniline (1.60 mL, 10.0 mmol), **68** (1.53 mL, 11.0 mmol), and potassium hydroxide (2.80 g, 50.0 mmol) in toluene (16 mL). The reaction was then allowed to reflux for 10 hours. After the reaction was complete, the resulting mixture was then transferred to a separatory funnel with brine and extracted with dichloromethane (20 mL × 3). The organic layers were combined, filtered and concentrated *in vacuo*. The resulting crude solid was purified via column chromatography using 99:1 to 85:15 hexanes:ethyl acetate to afford yellow solid **30** (612 mg, 22%).

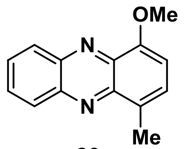

26

Yield: 9% yield; 315 mg of **26** was isolated as a yellow solid.

^{1}H NMR (400 MHz, CDCl$_3$): δ 8.39 (m, 1H), 8.28 (m, 1H), 7.87 – 7.79 (m, 2H), 7.56 (dq, *J* = 7.7, 1.1 Hz, 1H), 6.98 (d, *J* = 7.7 Hz, 1H), 4.15 (s, 3H), 2.83 (d, *J* = 1.1 Hz, 3H).

^{13}C NMR (100 MHz, CDCl$_3$): δ 153.5, 143.7, 142.9, 141.9, 137.2, 130.5, 130.2, 130.2, 129.9, 129.2, 129.2, 106.4, 56.5, 17.4.

HRMS (ESI): calc. for $C_{14}H_{13}N_2O$ [M+H]$^+$: 225.1022, found: 225.1017.

MP: 148 - 150 °C.

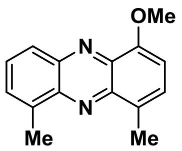

27

Yield: 6% yield; 296 mg of **27** was isolated as a yellow solid.

^{1}H NMR (400 MHz, CDCl$_3$): δ 8.22 (ddd, *J* = 8.6, 1.6, 0.8 Hz, 1H), 7.70 (dd, *J* = 8.6, 6.8 Hz, 1H), 7.65 (m, 1H), 7.53 (dq, *J* = 7.7, 1.2 Hz, 1H), 6.96 (d, *J* = 7.7 Hz, 1H), 4.14 (s, 3H), 2.92 (s, 3H), 2.82 (d, *J* = 1.2 Hz, 3H).

^{13}C NMR (100 MHz, CDCl$_3$): δ 153.3, 142.7, 142.3, 142.3, 142.0, 138.2, 136.7, 130.1, 129.6, 128.6, 128.0, 106.3, 56.4, 17.6, 17.2.

HRMS (ESI): calc. for $C_{15}H_{15}N_2O$ [M+H]$^+$: 239.1179, found: 239.1171.

MP: 193 - 195 °C.

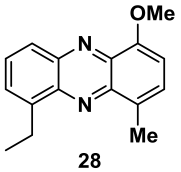

28

Yield: 9% yield; 460 mg of **28** was isolated as a yellow solid.

^{1}H NMR (400 MHz, CDCl$_3$): δ 8.21 (dd, *J* = 8.7, 1.2 Hz, 1H), 7.72 (dd, *J* = 8.7, 6.8 Hz, 1H), 7.62 (dq, *J* = 6.8, 1.2 Hz, 1H), 7.49 (dq, *J* = 7.7, 1.2 Hz, 1H), 6.92 (d, *J* = 7.7 Hz, 1H), 4.12 (s, 3H), 3.42 (q, *J* = 7.5 Hz, 2H), 2.80 (d, *J* = 1.2 Hz, 3H), 1.46 (t, *J* = 7.5 Hz, 3H).

^{13}C NMR (100 MHz, CDCl$_3$): δ 153.4, 143.9, 142.6, 142.1, 141.7, 136.7, 130.2, 129.7, 128.6, 128.1, 128.0, 106.3, 56.4, 24.6, 17.2, 15.0.

HRMS (ESI): calc. for $C_{16}H_{17}N_2O$ [M+H]$^+$: 253.1335, found: 253.1335.

MP: 113 - 115 °C.

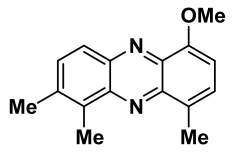

29

Yield: 8% yield; 386 mg of **29** was isolated as a yellow solid.

^{1}H NMR (400 MHz, $CDCl_3$): δ 8.12 (d, *J* = 8.9 Hz, 1H), 7.61 (d, *J* = 8.9 Hz, 1H), 7.48 (dq, *J* = 7.7, 1.1 Hz, 1H), 6.90 (d, *J* = 7.7 Hz, 1H), 4.12 (s, 3H), 2.85 (s, 3H), 2.81 (d, *J* = 1.1 Hz, 3H), 2.55 (s, 3H).

^{13}C NMR (100 MHz, $CDCl_3$): δ 153.4, 142.6, 142.1, 140.8, 137.7, 136.0, 134.4, 134.0, 129.6, 128.5, 126.9, 105.9, 56.4, 20.8, 17.2, 13.2.

HRMS (ESI): calc. for $C_{16}H_{17}N_2O$ $[M+H]^+$: 253.1335, found: 253.1346.

MP: 154 - 156 °C.

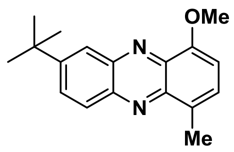

30

Yield: 22% yield; 613 mg of **30** was isolated as a yellow solid.

^{1}H NMR (400 MHz, $CDCl_3$): δ 8.30 (dd, *J* = 2.2, 0.6 Hz, 1H), 8.19 (dd, *J* = 9.2, 0.6 Hz, 1H), 7.93 (dd, *J* = 9.2, 2.2 Hz, 1H), 7.51 (dq, *J* = 7.7, 1.1 Hz, 1H), 6.95 (d, *J* = 7.7 Hz, 1H), 4.14 (s, 3H), 2.81 (d, *J* = 1.1 Hz, 3H), 1.47 (s, 9H).

^{13}C NMR (100 MHz, $CDCl_3$): δ 153.1, 153.1, 143.1, 141.6, 141.4, 136.8, 129.8, 128.9, 128.8, 128.3, 124.6, 105.9, 56.0, 35.4, 30.8, 17.2.

HRMS (ESI): calc. for $C_{18}H_{21}N_2O$ $[M+H]^+$: 281.1648, found: 281.1659.

MP: 85 - 87 °C.

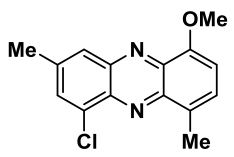

31

Yield: 2% yield; 40 mg of **31** was isolated as a yellow solid.

^{1}H NMR (400 MHz, $CDCl_3$): δ 8.08 (m, 1H), 7.81 (d, *J* = 1.8 Hz, 1H), 7.56 (dq, *J* = 7.7, 1.1 Hz, 1H), 6.99 (d, *J* = 7.7 Hz, 1H), 4.14 (s, 3H), 2.86 (s, 3H), 2.62 (s, 3H).

^{13}C NMR (100 MHz, $CDCl_3$): δ 153.1, 142.9, 142.3, 140.3, 138.1, 137.3, 132.9, 132.6, 129.7, 129.2, 127.6, 107.1, 56.4, 22.3, 17.1.

HRMS (ESI): calc. for $C_{15}H_{14}ClN_2O$ $[M+H]^+$: 273.0789, found: 273.0797.

MP: 198 - 200 °C.

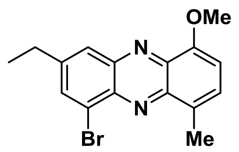

32

Yield: 5% yield; 168 mg of **32** was isolated as a yellow solid.

^{1}H NMR (400 MHz, $CDCl_3$): δ 8.44 (d, *J* = 2.0 Hz, 1H), 7.71 (m, 1H), 7.56 (dq, *J* = 7.8, 1.1 Hz, 1H), 6.98 (d, *J* = 7.8 Hz, 1H), 4.14 (s, 3H), 3.42 (q, *J* = 7.5 Hz, 2H), 2.81 (d, *J* = 1.1 Hz, 3H), 1.47 (t, *J* = 7.5 Hz, 3H).

^{13}C NMR (100 MHz, $CDCl_3$): δ 153.3, 145.6, 142.6, 142.4, 140.3, 137.0, 131.7, 129.9, 129.7, 129.1, 124.8, 107.0, 56.5, 24.4, 17.1, 14.7.

HRMS (ESI): calc. for $C_{16}H_{16}BrN_2O$ $[M+H]^+$: 331.0441, found: 331.0448.

MP: 151 - 153 °C.

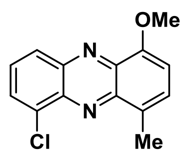

33

Yield: 4% yield; 224 mg of **33 w**as isolated as a yellow solid.

^{1}H NMR (400 MHz, $CDCl_3$): δ 8.33 (dd, *J* = 8.8, 1.3 Hz, 1H), 7.95 (dd, *J* = 7.3, 1.3 Hz, 1H), 7.73 (dd, *J* = 8.8, 7.3 Hz, 1H), 7.60 (dq, *J* = 7.8, 1.1 Hz, 1H), 7.02 (d, *J* = 7.8 Hz, 1H), 4.15 (s, 3H), 2.87 (d, *J* = 1.1 Hz, 3H).

^{13}C NMR (100 MHz, $CDCl_3$): δ 153.3, 143.5, 142.4, 139.5, 137.4, 133.7, 130.0, 129.9, 129.9, 129.5, 129.3, 107.3, 56.6, 17.1.

HRMS (ESI): calc. for $C_{14}H_{12}ClN_2O$ $[M+H]^+$: 259.0633, found: 259.0639.

MP: 182 - 184 °C.

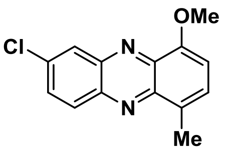

34

Yield: 14% yield; 366 mg of **34** was isolated as a yellow solid.

^{1}H NMR (400 MHz, $CDCl_3$): δ 8.38 (dd, *J* = 2.4, 0.5 Hz, 1H), 8.19 (dd, *J* = 9.3, 0.5 Hz, 1H), 7.74 (dd, *J* = 9.3, 2.4 Hz, 1H), 7.54 (dq, *J* = 7.7, 1.1 Hz, 1H), 6.97 (d, *J* = 7.7 Hz, 1H), 4.13 (s, 3H), 2.78 (d, *J* = 1.1 Hz, 3H).

^{13}C NMR (100 MHz, $CDCl_3$): δ 153.4, 143.6, 141.8, 141.3, 137.5, 136.1, 131.9, 131.2, 129.5, 129.4, 128.6, 107.1, 56.5, 17.3.

HRMS (ESI): calc. for $C_{14}H_{12}ClN_2O$ $[M+H]^+$: 259.0633, found: 259.0629.

MP: 169 - 171 °C.

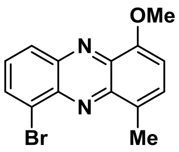

35

Yield: 7% yield; 625 mg of **35** was isolated as a yellow solid.

^{1}H NMR (400 MHz, $CDCl_3$): δ 8.33 (dd, *J* = 8.8, 1.2 Hz, 1H), 8.14 (dd, *J* = 7.3, 1.2 Hz, 1H), 7.63 (dd, *J* = 8.8, 7.3 Hz, 1H), 7.53 (dq, *J* = 7.7, 1.1 Hz, 1H), 6.95 (d, *J* = 7.7 Hz, 1H), 4.12 (s, 3H), 2.83 (d, *J* = 1.1 Hz, 3H).

^{13}C NMR (100 MHz, $CDCl_3$): δ 153.1, 143.6, 142.2, 140.0, 137.4, 133.6, 130.1, 130.0, 129.8, 129.7, 124.9, 107.2, 56.5, 17.0.

HRMS (ESI): calc. for $C_{14}H_{12}BrN_2O$ $[M+H]^+$: 303.0128, found: 303.0115.

MP: 209 - 211 °C.

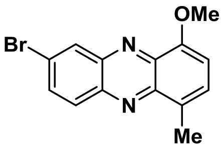

36

Yield: 13% yield; 528 mg of **36** was isolated as a yellow solid.

^{1}H NMR (400 MHz, $CDCl_3$): δ 8.59 (dd, *J* = 2.2, 0.5 Hz, 1H), 8.13 (dd, *J* = 9.2, 0.5 Hz, 1H), 7.88 (dd, *J* = 9.2, 2.2 Hz, 1H), 7.57 (dq, *J* = 7.7, 1.1 Hz, 1H), 6.98 (d, *J* = 7.7 Hz, 1H), 4.14 (s, 3H), 2.80 (d, *J* = 1.1 Hz, 3H).

^{13}C NMR (100 MHz, $CDCl_3$): δ 153.4, 143.7, 142.0, 141.4, 137.4, 134.2, 132.1, 131.2, 129.6, 129.3, 124.5, 107.1, 56.5, 17.3.

HRMS (ESI): calc. for $C_{14}H_{12}BrN_2O$ $[M+H]^+$: 303.0128, found: 303.0115.

MP: 167 - 169 °C.

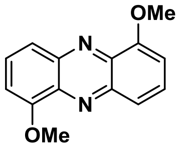

37

Yield: 3% yield; 650 mg of **37** was isolated as a yellow solid.

^{1}H NMR (400 MHz, $CDCl_3$): δ 7.98 (dd, *J* = 8.9, 1.1 Hz, 2H), 7.73 (dd, *J* = 8.9, 7.6 Hz, 2H), 7.08 (dd, *J* = 7.6, 1.1 Hz, 2H), 4.16 (s, 6H).

^{13}C NMR (100 MHz, $CDCl_3$): δ 155.1, 143.2, 137.1, 130.3, 122.2, 107.0, 56.7.

Note: NMR spectra matches those previously reported.[2]

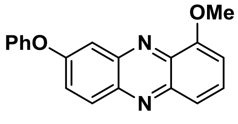

38

Yield: 13% yield; 566 mg of **38** was isolated as a yellow solid.

^{1}H NMR (400 MHz, $CDCl_3$): δ 8.19 (d, *J* = 9.4 Hz, 1H), 7.79 (dd, *J* = 8.9, 1.1 Hz, 1H), 7.73 (dd, *J* = 9.4, 2.7 Hz, 1H), 7.67 (dd, *J* = 8.8, 7.6 Hz, 1H), 7.51 (d, *J* = 2.7 Hz, 1H), 7.49 – 7.42 (m, 2H), 7.28 (m, 1H), 7.24 – 7.17 (m, 2H), 7.04 (d, *J* = 7.6 Hz, 1H), 4.12 (s, 3H). **Note:** TMS was used as a reference (0.00 ppm) due to the $CHCl_3$ signal being buried in a concentrated NMR sample.

^{13}C NMR (100 MHz, CDCl$_3$): δ 160.5, 154.9, 154.9, 143.4, 143.2, 141.0, 136.9, 131.0, 130.5, 129.5, 126.3, 125.6, 121.6, 121.4, 110.9, 106.9, 56.5.

HRMS (ESI): calc. for $C_{19}H_{15}N_2O_2$ $[M+H]^+$: 303.1128, found: 303.1132.

MP: 187 - 189 °C.

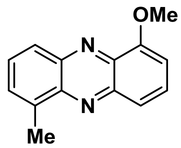

39

Yield: 7% yield; 444 mg of **39** was isolated as a yellow solid.

^{1}H NMR (400 MHz, d_6-DMSO): δ 8.10 (dd, J = 8.2, 1.9 Hz, 1H), 7.88 – 7.75 (m, 4H), 7.27 (d, J = 7.4 Hz, 1H), 4.07 (s, 3H), 2.83 (s, 3H).

^{13}C NMR (100 MHz, d_6-DMSO): δ 154.9, 142.7, 142.2, 141.8, 136.9, 136.0, 131.0, 130.4, 130.3, 127.6, 120.9, 107.2, 56.0, 17.3.

HRMS (ESI): calc. for $C_{14}H_{13}N_2O$ $[M+H]^+$: 225.1022, found: 225.1021.

MP: 189 - 191 °C.

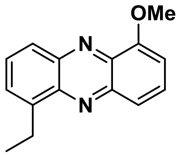

40

Yield: 5% yield; 212 mg of **40** was isolated as an orange solid.

^{1}H NMR (400 MHz, CDCl$_3$): δ 8.23 (m, 1H), 7.86 (dd, *J* = 8.7, 1.1 Hz, 1H), 7.79 – 7.68 (m, 2H), 7.66 (dq, *J* = 6.8, 1.1 Hz, 1H), 7.06 (dd, *J* = 7.5, 1.1 Hz, 1H), 4.18 (s, 3H), 3.43 (q, *J* = 7.5 Hz, 2H), 1.45 (t, *J* = 7.5 Hz, 3H).

^{13}C NMR (100 MHz, CDCl$_3$): δ 155.2, 143.7, 143.5, 142.8, 142.6, 136.6, 130.4, 130.0, 128.3, 128.2, 122.2, 106.4, 56.6, 24.3, 14.9.

HRMS (ESI): calc. for $C_{15}H_{15}N_2O$ $[M+H]^+$: 239.1179, found: 239.1177.

MP: 132 - 134 °C.

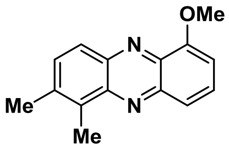

41

Yield: 6% yield; 300 mg of **41** was isolated as a red solid.

^{1}H NMR (400 MHz, CDCl$_3$): δ 8.14 (m, 1H), 7.85 (dd, *J* = 8.9, 1.1 Hz, 1H), 7.70 (dd, *J* = 8.9, 7.6 Hz, 1H), 7.64 (d, *J* = 8.9 Hz, 1H), 7.03 (d, *J* = 7.6 Hz, 1H), 4.17 (s, 3H), 2.86 (s, 3H), 2.57 (s, 3H).

^{13}C NMR (100 MHz, CDCl$_3$): δ 155.1, 143.6, 143.2, 141.3, 138.1, 135.8, 134.2, 134.0, 129.8, 127.1, 122.0, 106.0, 56.5, 20.9, 13.3.

HRMS (ESI): calc. for $C_{15}H_{15}N_2O$ $[M+H]^+$: 239.1179, found: 239.1172.

MP: 156 - 158 °C.

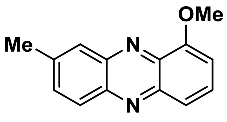

42

Yield: 2% yield; 158 mg of **42** was isolated as a yellow solid.

^{1}H NMR (400 MHz, CDCl$_3$): δ 8.14 (dq, *J* = 1.8, 1.1 Hz, 1H), 8.09 (d, *J* = 8.9 Hz, 1H), 7.79 (dd, *J* = 8.9, 1.1 Hz, 1H), 7.72 – 7.64 (m, 2H), 7.03 (dd, *J* = 7.6, 1.1 Hz, 1H), 4.15 (s, 3H), 2.62 (d, *J* = 1.1 Hz, 3H).

^{13}C NMR (100 MHz, CDCl$_3$): δ 155.2, 143.9, 142.5, 142.5, 141.0, 136.9, 134.0, 130.1, 128.9, 128.5, 121.6, 106.5, 56.6, 22.4.

HRMS (ESI): calc. for $C_{14}H_{13}N_2O$ $[M+H]^+$: 225.1022, found: 225.1027.

MP: 181 - 183 °C.

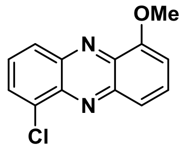

43

Yield: 3% yield; 155 mg of **43** was isolated as a yellow solid.

^{1}H NMR (400 MHz, CDCl$_3$): δ 8.33 (dd, *J* = 8.8, 1.4 Hz, 1H), 7.97 (m, 1H), 7.95 (d, *J* = 1.3 Hz, 1H), 7.80 (dd, *J* = 8.9, 7.7 Hz, 1H), 7.73 (dd, *J* = 8.8, 7.3 Hz, 1H), 7.11 (dd, *J* = 7.7, 1.1 Hz, 1H), 4.18 (s, 3H).

^{13}C NMR (100 MHz, CDCl$_3$): δ 155.1, 144.3, 142.8, 140.4, 137.3, 133.0, 131.4, 130.4, 129.6, 129.6, 122.0, 107.4, 56.8.

HRMS (ESI): calc. for $C_{13}H_{10}ClN_2O$ [M+H]$^+$: 245.0476, found: 245.0451.

MP: 221 - 223 °C.

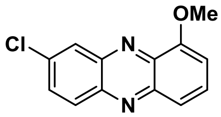

44

Yield: 12% yield; 292 mg of **44** was isolated as a yellow solid.

^{1}H NMR (400 MHz, CDCl$_3$): δ 8.41 (d, *J* = 2.3 Hz, 1H), 8.17 (d, *J* = 9.2 Hz, 1H), 7.87 – 7.71 (m, 3H), 7.10 (dd, *J* = 7.3, 1.4 Hz, 1H), 4.18 (s, 3H).

^{13}C NMR (100 MHz, CDCl$_3$): 155.2, 144.3, 142.2, 142.1, 137.3, 136.4, 132.3, 131.0, 130.8, 128.7, 121.6, 107.2, 56.7.

HRMS (ESI): calc. for $C_{13}H_{10}ClN_2O$ [M+H]$^+$: 245.0476, found: 245.0473.

MP: 206 - 208 °C.

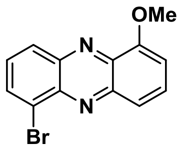

45

Yield: 1% yield; 106 mg of **45** was isolated as an orange solid.

^{1}H NMR (400 MHz, CDCl$_3$): δ 8.38 (dd, J = 8.7, 1.2 Hz, 1H), 8.20 (dd, J = 7.3, 1.2 Hz, 1H), 7.98 (dd, J = 8.9, 1.1 Hz, 1H), 7.81 (dd, J = 8.9, 7.6 Hz, 1H), 7.68 (dd, J = 8.8, 7.3 Hz, 1H), 7.12 (dd, J = 7.6, 1.1 Hz, 1H), 4.19 (s, 3H).

^{13}C NMR (100 MHz, CDCl$_3$): δ 155.1, 144.6, 142.8, 141.1, 137.4, 134.2, 131.4, 130.4, 130.3, 124.2, 122.1, 107.4, 56.8.

HRMS (ESI): calc. for $C_{13}H_9BrN_2ONa$ [M+Na]$^+$ 310.9805, found: 310.9805.

MP: 224 - 226 °C.

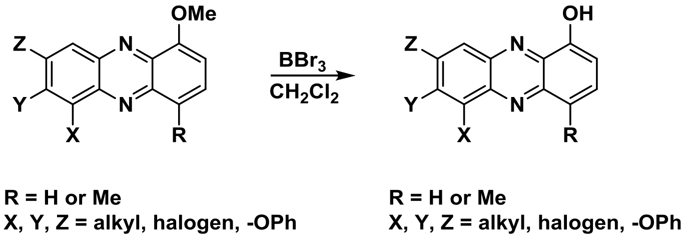

General procedure for demethylation of 1-methoxyphenazines: To a round bottom flask, **39** (376 mg, 1.68 mmol) was dissolved in anhydrous dichloromethane (50 mL) and cooled to -78 °C before dropwise addition of 1M boron tribromide solution in dichloromethane (10.0 mL, 10.0 mmol). The reaction was left to stir at -78 °C for 1 hour, and allowed to reach ambient temperature overnight. The reaction was then heated to reflux for 8 hours until complete (monitored by TLC). Upon completion of the reaction, brine (50 mL) was added to quench the reaction. The contents of the resulting biphasic mixture were then transferred to a separatory funnel and dichloromethane was used to extract the product. The resulting organic layers were dried with sodium sulfate, filtered through cotton, and removed *in vacuo*. The resulting solid was purified via column chromatography using dichloromethane to elute compound **60** as a yellow solid (100%, 350 mg). **Note:** Some

1-hydroxyphenazines were purified with the addition of 1% acetic acid to 99% dichloromethane via column chromatography.

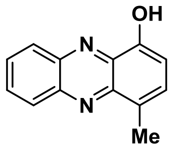

46

Yield: 100% yield; 94 mg of **46** was isolated as a yellow solid.

^{1}H NMR (400 MHz, CDCl$_3$): δ 8.31 (m, 1H), 8.21 (m, 1H), 8.07 (s, 1H), 7.91 – 7.79 (m, 2H), 7.56 (dq, *J* = 7.6, 1.2 Hz, 1H), 7.13 (d, *J* = 7.6 Hz, 1H), 2.82 (d, *J* = 1.2 Hz, 3H).

^{13}C NMR (100 MHz, CDCl$_3$): δ 149.9, 143.5, 143.4, 140.8, 135.0, 130.4, 130.4, 130.4, 130.3, 129.1, 127.9, 108.7, 17.2.

HRMS (ESI): calc. for $C_{13}H_{11}N_2O$ [M+H]$^+$: 211.0866, found: 211.0871.

MP: 197 -199 °C.

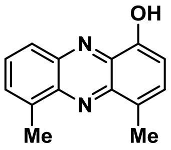

47

Yield: 100% yield; 89 mg of **47** was isolated as a yellow solid.

^{1}H NMR (400 MHz, CDCl$_3$): δ 8.02 (s, 1H), 7.97 (ddd, *J* = 8.6, 1.6, 0.8 Hz, 1H), 7.66 (dd, *J* = 8.6, 6.8 Hz, 1H), 7.60 (m, 1H), 7.48 (dq, *J* = 7.6, 1.2 Hz, 1H), 7.08 (d, *J* = 7.6 Hz, 1H), 2.88 (s, 3H), 2.77 (d, *J* = 1.2 Hz, 3H).

^{13}C NMR (100 MHz, CDCl$_3$): δ 149.7, 142.8, 142.2, 140.9, 138.5, 134.5, 130.3, 129.8, 129.5, 128.2, 126.9, 108.5, 17.7, 16.8.

HRMS (ESI): calc. for $C_{14}H_{13}N_2O$ [M+H]$^+$: 225.1022, found: 225.1024.

MP: 174 - 176 °C.

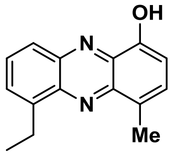

48

Yield: 93% yield; 88 mg of **48** was isolated as a yellow solid.

^{1}H NMR (400 MHz, CDCl$_3$): δ 8.03 (s, 1H), 7.96 (dd, *J* = 8.7, 1.4 Hz, 1H), 7.68 (dd, *J* = 8.7, 6.8 Hz, 1H), 7.59 (dd, *J* = 6.8, 1.4 Hz, 1H), 7.47 (dq, *J* = 7.5, 1.1 Hz, 1H), 7.07 (d, *J* = 7.5 Hz, 1H), 3.39 (q, *J* = 7.5 Hz, 2H), 2.77 (d, *J* = 1.1 Hz, 3H), 1.45 (t, *J* = 7.5 Hz, 3H).

^{13}C NMR (100 MHz, CDCl$_3$): δ 149.7, 144.2, 142.1, 142.1, 140.9, 134.4, 130.4, 129.7, 128.2, 127.8, 126.8, 108.5, 24.7, 16.8, 14.9.

HRMS (ESI): calc. for $C_{15}H_{15}N_2O$ [M+H]$^+$: 239.1179, found: 239.1182.

MP: 111 - 113 °C.

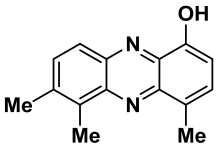

49

Yield: 33% yield; 31 mg of **49** was isolated as an orange solid.

^{1}H NMR (400 MHz, CDCl$_3$): δ 7.97 (br. s, 1H), 7.92 (d, *J* = 8.9 Hz, 1H), 7.61 (d, *J* = 8.9 Hz, 1H), 7.50 (dq, *J* = 7.5, 1.1 Hz, 1H), 7.07 (d, *J* = 7.5 Hz, 1H), 2.86 (s, 3H), 2.81 (d, *J* = 1.1 Hz, 3H), 2.57 (s, 3H).

^{13}C NMR (100 MHz, CDCl$_3$): δ 149.9, 142.9, 142.4, 139.9, 137.6, 135.0, 134.3, 134.0, 129.7, 128.2, 125.9, 108.1, 20.8, 16.8, 13.3.

HRMS (ESI): calc. for $C_{15}H_{15}N_2O$ [M+H]$^+$: 239.1179, found: 239.1182.

MP: 156 - 158 °C.

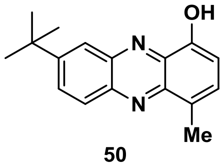

50

Yield: 89% yield; 109 mg of **50** was isolated as a red solid.

^{1}H NMR (400 MHz, $CDCl_3$): δ 8.21 (dd, *J* = 9.2, 0.6 Hz, 1H), 8.10 (br. s, 1H), 8.08 (dd, *J* = 2.2, 0.6 Hz, 1H), 7.93 (dd, *J* = 9.2, 2.2 Hz, 1H), 7.50 (dq, *J* = 7.5, 1.1 Hz, 1H), 7.10 (d, *J* = 7.5 Hz, 1H), 2.80 (d, *J* = 1.1, Hz, 3H), 1.49 (s, 9 H).

^{13}C NMR (100 MHz, $CDCl_3$): δ 153.8, 149.8, 143.0, 142.3, 140.9, 135.0, 130.2, 129.8, 129.5, 127.8, 123.5, 108.5, 35.6, 31.0, 17.2.

HRMS (ESI): calc. for $C_{17}H_{19}N_2O$ $[M+H]^+$: 267.1492, found: 267.1495.

MP: 97 - 99 °C.

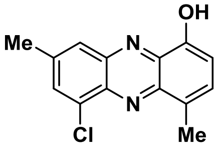

51

Yield: 82% yield; 23 mg of **51** was isolated as a yellow solid.

^{1}H NMR (400 MHz, $CDCl_3$): δ 7.92 (s, 1H), 7.89 (m, 1H), 7.81 (d, *J* = 1.8 Hz, 1H), 7.57 (dq, *J* = 7.6, 1.1 Hz, 1H), 7.15 (d, *J* = 7.6 Hz, 1H), 2.85 (d, *J* = 1.1 Hz, 3H), 2.63 (d, *J* = 1.2 Hz, 3H).

^{13}C NMR (100 MHz, $CDCl_3$): δ 149.5, 142.4, 141.2, 140.6, 138.6, 135.0, 133.3, 132.5, 130.4, 128.5, 126.5, 109.5, 22.2, 16.8.

HRMS (ESI): calc. for $C_{14}H_{12}ClN_2O$ $[M+H]^+$: 259.0633, found: 259.0644.

MP: 163 - 165 °C.

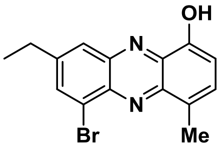

52

Yield: 81% yield; 70 mg of **52** was isolated as a yellow solid.

^{1}H NMR (400 MHz, $CDCl_3$): δ 8.19 (d, *J* = 2.2 Hz, 1H), 7.88 (s, 1H), 7.68 (m, 1H), 7.52 (dq, *J* = 7.6, 1.1 Hz, 1H), 7.11 (d, *J* = 7.6 Hz, 1H), 3.38 (q, *J* = 7.5 Hz, 2H), 2.77 (d, *J* = 1.1 Hz, 3H), 1.45 (t, *J* = 7.5 Hz, 3H).

^{13}C NMR (100 MHz, $CDCl_3$): δ 149.5, 145.8, 141.9, 141.0, 140.6, 134.6, 131.4, 130.2, 128.7, 128.3, 125.1, 109.3, 24.4, 16.8, 14.5.

HRMS (ESI): calc. for $C_{15}H_{14}BrN_2O$ $[M+H]^+$: 317.0284, found: 317.0296.

MP: 134 - 136 °C.

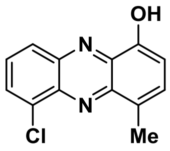

53

Yield: 93% yield; 88 mg of **52** was isolated as an orange solid.

^{1}H NMR (400 MHz, $CDCl_3$): δ 8.12 (dd, *J* = 8.7, 1.3 Hz, 1H), 7.94 (dd, *J* = 7.3, 1.3 Hz, 1H), 7.92 (s, 1H), 7.72 (dd, *J* = 8.7, 7.3 Hz, 1H), 7.59 (dq, *J* = 7.6, 1.1 Hz, 1H), 7.16 (d, *J* = 7.6 Hz, 1H), 2.84 (d, *J* = 1.1 Hz, 3H).

^{13}C NMR (100 MHz, $CDCl_3$): δ 149.6, 143.0, 141.3, 140.0, 135.2, 134.1, 131.1, 129.9, 129.8, 128.6, 128.3, 109.7, 16.8.

HRMS (ESI): calc. for $C_{13}H_{10}ClN_2O$ $[M+H]^+$: 245.0476, found: 245.0467.

MP: 186 - 188 °C.

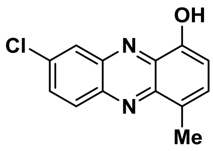

54

Yield: 94% yield; 89 mg of **54** was isolated as a yellow solid.

^{1}H NMR (400 MHz, $CDCl_3$): δ 8.23 (dd, *J* = 9.3, 0.6 Hz, 1H), 8.19 (dd, *J* = 2.3, 0.6 Hz, 1H), 7.94 (s, 1H), 7.76 (dd, *J* = 9.3, 2.3 Hz, 1H), 7.56 (dd, *J* = 7.6, 1.1 Hz, 1H), 7.15 (d, *J* = 7.6 Hz, 1H), 2.79 (d, *J* = 1.1 Hz, 3H).

^{13}C NMR (100 MHz, $CDCl_3$): δ 149.8, 143.3, 141.9, 140.7, 136.5, 135.3, 131.8, 131.6, 130.8, 128.1, 127.6, 109.5, 17.1.

HRMS (ESI): calc. for $C_{13}H_{10}ClN_2O$ [M+H]$^+$: 245.0476, found: 245.0464.

MP: 166 - 168 °C.

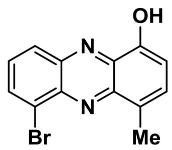

55

Yield: 100% yield; 114 mg of **55** was isolated as a yellow solid.

^{1}H NMR (400 MHz, CDCl$_3$): δ 8.23 – 8.13 (m, 2H), 7.90 (s, 1H), 7.66 (m, 1H), 7.59 (d, *J* = 7.6 Hz, 1H), 7.16 (d, *J* = 7.6 Hz, 1H), 2.85 (s, 3H).

^{13}C NMR (100 MHz, CDCl$_3$): δ 149.5, 143.2, 141.2, 140.6, 135.2, 133.6, 131.1, 130.3, 129.0, 128.6, 125.3, 109.7, 16.8.

HRMS (ESI): calc. for $C_{13}H_{10}BrN_2O$ [M+H]$^+$: 288.9971, found: 288.9978.

MP: 198 - 200 °C.

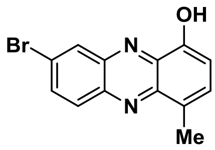

56

Yield: 87% yield; 100 mg of **56** was isolated as a yellow solid.

^{1}H NMR (400 MHz, CDCl$_3$): δ 8.39 (d, *J* = 2.1 Hz, 1H), 8.15 (d, *J* = 9.2 Hz, 1H), 7.93 (s, 1H), 7.87 (dd, *J* = 9.2, 2.1 Hz, 1H), 7.56 (dq, *J* = 7.6, 1.2 Hz, 1H), 7.14 (d, *J* = 7.6 Hz, 1H), 2.79 (d, *J* = 1.2 Hz, 3H).

^{13}C NMR (100 MHz, CDCl$_3$): δ 149.7, 143.2, 141.9, 140.9, 135.1, 134.1, 131.4, 131.0, 130.8, 128.1, 124.9, 109.5, 17.1.

HRMS (ESI): calc. for $C_{13}H_{10}BrN_2O$ [M+H]$^+$: 288.9971, found: 288.9978.

MP: 168 - 170 °C.

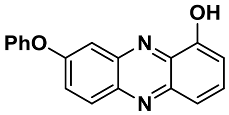

59

Yield: 98% yield; 117 mg of **59** was isolated as a yellow solid.

^{1}H NMR (400 MHz, CDCl$_3$): δ 8.24 (dd, *J* = 9.5, 0.5 Hz, 1H), 8.04 (br. s, 1H), 7.75 (dd, *J* = 8.9, 1.4 Hz, 1H), 7.74 – 7.69 (m, 2H), 7.53 – 7.46 (m, 2H), 7.38 (dd, *J* = 2.7, 0.5 Hz, 1H), 7.31 (m, 1H), 7.25 – 7.18 (m, 3H).

^{13}C NMR (100 MHz, CDCl$_3$): δ 160.2, 155.1, 151.5, 142.8, 142.5, 141.7, 134.8, 131.5, 130.9, 130.5, 126.2, 125.5, 121.0, 120.1, 110.4, 109.4.

HRMS (ESI): calc. for $C_{18}H_{13}N_2O_2$ [M+H]$^+$: 289.0972, found: 289.0966.

MP: 143 -145 °C.

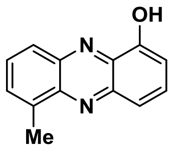

60

Yield: 99% yield; 350 mg of **60** was isolated as a yellow solid.

^{1}H NMR (400 MHz, CDCl$_3$): δ 8.21 (s, 1H), 8.04 (d, *J* = 8.5 Hz, 1H), 7.81 (dd, *J* = 8.9, 1.1 Hz, 1H), 7.78 – 7.63 (m, 3H), 7.22 (dd, *J* = 7.3, 1.2 Hz, 1H), 2.92 (s, 3H).

^{13}C NMR (100 MHz, CDCl$_3$): δ 151.7, 144.0, 143.2, 141.5, 138.2, 134.3, 131.4, 130.5, 130.0, 127.2, 120.5, 108.8, 18.0.

HRMS (ESI): calc. for $C_{13}H_{11}N_2O$ [M+H]$^+$: 211.0866, found: 211.0873.

MP: 178 - 180 °C.

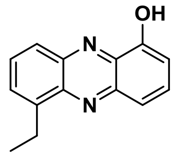

Yield: 99% yield; 84 mg of **61** was isolated as a yellow solid.

^{1}H NMR (400 MHz, CDCl$_3$): δ 8.21 (s, 1H), 8.03 (d, *J* = 8.7 Hz, 1H), 7.80 (dd, *J* = 9.0, 1.2 Hz, 1H), 7.77 – 7.70 (m, 2H), 7.65 (d, *J* = 6.8 Hz, 1H), 7.21 (dd, *J* = 7.3, 1.2 Hz, 1H), 3.43 (q, *J* = 7.5 Hz, 2H), 1.45 (t, *J* = 7.5 Hz, 3H).

^{13}C NMR (100 MHz, CDCl$_3$): δ 151.7, 144.0, 143.4, 143.2, 141.5, 134.3, 131.3, 130.7, 128.2, 127.1, 120.6, 108.8, 24.5, 15.0.

HRMS (ESI): calc. for $C_{14}H_{13}N_2O$ [M+H]$^+$: 225.1022, found: 225.1024.

MP: 118 - 120 °C.

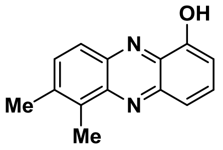

Yield: 100% yield; 139 mg of **62** was isolated as a yellow solid.

^{1}H NMR (400 MHz, *d$_6$*-DMSO): δ 10.49 (s, 1H), 8.03 (d, *J* = 8.9 Hz, 1H), 7.79 – 7.71 (m, 2H), 7.69 (dd, *J* = 8.7, 1.4 Hz, 1H), 7.15 (dt, *J* = 7.3, 1.4 Hz, 1H), 2.78 (s, 3H), 2.56 (s, 3H).

^{1}H NMR (400 MHz, CDCl$_3$): δ 8.17 (s, 1H), 7.93 (d, *J* = 8.9 Hz, 1H), 7.78 (dd, *J* = 8.8, 1.0 Hz, 1H), 7.71 (dd, J = 8.9, 7.3 Hz, 1H), 7.61 (d, *J* = 8.8 Hz, 1H), 7.18 (dd, *J* = 7.3, 1.0 Hz, 1H), 2.85 (s, 3H), 2.57 (s, 3H).

^{13}C NMR (100 MHz, *d$_6$*-DMSO): δ 153.4, 142.9, 142.2, 140.2, 138.0, 134.7, 134.1, 133.3, 131.4, 126.2, 119.3, 110.0, 20.3, 13.0.

^{13}C NMR (100 MHz, CDCl$_3$): δ 151.7, 143.9, 143.2, 140.3, 138.1, 134.5, 133.7, 131.1, 126.0, 120.5, 108.4, 21.0, 13.5. **Note:** One ^{13}C signal missing, likely due to overlap.

HRMS (ESI): calc. for $C_{14}H_{13}N_2O$ [M+H]$^+$: 225.1022, found: 225.1029.

MP: 144 - 146 °C.

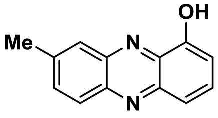

Yield: 97% yield; 75 mg of **63** was isolated as a yellow solid.

^{1}H NMR (400 MHz, CDCl$_3$): δ 8.21 (s, 1H), 8.13 (d, *J* = 8.9 Hz, 1H), 7.94 (m, 1H), 7.77 – 7.69 (m, 2H), 7.67 (dd, *J* = 8.9, 2.0 Hz, 1H), 7.21 (dd, *J* = 6.9, 1.6 Hz, 1H), 2.64 (d, *J* = 1.1 Hz, 3H).

^{13}C NMR (100 MHz, CDCl$_3$): δ 151.8, 143.3, 143.0, 141.3, 141.3, 134.6, 133.8, 131.2, 129.1, 127.3, 120.0, 109.0, 22.3.

HRMS (ESI): calc. for $C_{13}H_{11}N_2O$ [M+H]$^+$: 211.0866, found: 211.0873.

MP: 147 - 149 °C.

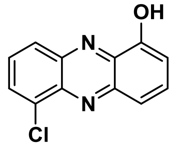

Yield: > 99% yield; 92 mg of **64** was isolated as a yellow solid.

^{1}H NMR (400 MHz, CDCl$_3$): δ 8.15 (dd, *J* = 8.8, 1.3 Hz, 1H), 8.10 (s, 1H), 7.97 (dd, *J* = 7.3, 1.3 Hz, 1H), 7.91 (dd, *J* = 8.9, 1.1 Hz, 1H), 7.81 (dd, *J* = 8.9, 7.4 Hz, 1H), 7.74 (dd, *J* = 8.8, 7.3 Hz, 1H), 7.27 (dd, *J* = 7.4, 1.1 Hz, 1H).

^{13}C NMR (100 MHz, CDCl$_3$): δ 151.6, 143.8, 141.8, 140.9, 135.0, 133.5, 132.7, 130.4, 129.9, 128.5, 120.6, 109.9.

HRMS (ESI): calc. for $C_{12}H_8ClN_2O$ [M+H]$^+$: 231.0320, found: 231.0331.

MP: 185 - 187 °C.

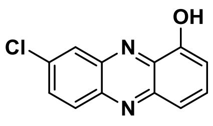

65

Yield: 100% yield; 85 mg of **65** was isolated as an orange solid.

^{1}H NMR (400 MHz, CDCl$_3$): δ 8.23 (d, *J* = 2.3 Hz, 1H), 8.21 (d, *J* = 9.3, Hz, 1H), 8.10 (s, 1H), 7.81 – 7.75 (m, 3H), 7.27 (dd, *J* = 6.2, 2.6 Hz, 1H).

^{13}C NMR (100 MHz, CDCl$_3$): δ 151.8, 144.0, 142.8, 141.2, 136.8, 135.1, 132.4, 132.3, 131.2, 127.7, 120.2, 109.9.

HRMS (ESI): calc. for $C_{12}H_8ClN_2O$ [M+H]$^+$: 231.0320, found: 231.0330.

MP: 190 - 192 °C.

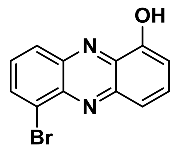

66

Yield: 100% yield; 72 mg of **66** was isolated as a yellow solid.

^{1}H NMR (400 MHz, CDCl$_3$): δ 8.22 (m, 1H), 8.20 (s, 1H), 8.09 (s, 1H), 7.93 (dd, *J* = 8.9, 1.1 Hz, 1H), 7.82 (dd, *J* = 8.9, 7.4 Hz, 1H), 7.69 (m, 1H), 7.28 (dd, *J* = 7.4, 1.1 Hz, 1H).

^{13}C NMR (100 MHz, CDCl$_3$): 151.5, 144.0, 141.6, 141.5, 135.0, 134.0, 132.6, 130.4, 129.2, 124.6, 120.5, 109.9.

HRMS (ESI): calc. for $C_{12}H_8BrN_2O$ [M+H]$^+$: 274.9815, found: 274.9824.

MP: 171 - 173 °C.

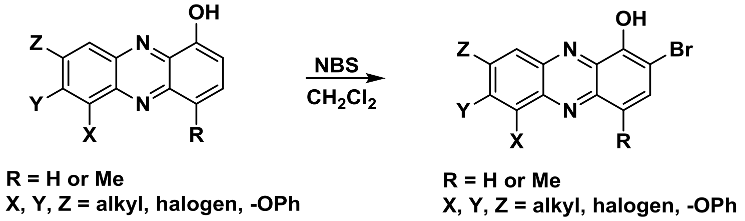

General procedure for bromination of 1-hydroxyphenazines: 60 (156 mg, 0.742 mmol) and *N*-bromosuccinimide (277 mg, 1.56 mmol) were dissolved in dichloromethane (60.0 mL) and allowed to stir at room temperature for 4 hours. The reaction contents were washed with brine (60.0 mL) and extracted with dichloromethane. The extracts were dried with sodium sulfate, filtered, and concentrated *in vacuo*. The resulting solid was purified via column chromatography using 99:1 dichloromethane:acetic acid to elute **17** as a yellow solid. **Note**: When R = Me, 1.0 equivalent of *N*-bromosuccinimide was used for bromination.

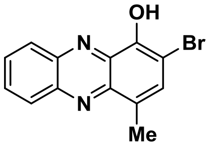

4

Yield: 39% yield; 52 mg of **4** was isolated as a yellow solid.

^{1}H NMR (400 MHz, CDCl$_3$): δ 8.31 (br. s, 1H), 8.23 (m, 1H), 8.15 (m, 1H), 7.86 – 7.79 (m, 2H), 7.64 (q, *J* = 1.2 Hz, 1H), 2.76 (d, *J* = 1.2 Hz, 3H).

^{13}C NMR (100 MHz, CDCl$_3$): δ 147.2, 143.2, 142.3, 140.9, 134.5, 133.6, 131.1, 130.7, 130.3, 129.2, 129.0, 103.3, 16.8.

HRMS (ESI): calc. for $C_{13}H_{10}BrN_2O$ [M+H]$^+$: 288.9971, found: 288.9978.

MP: 178 - 180 °C.

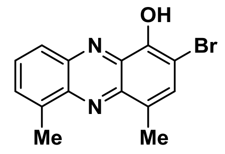

5

Yield: 91% yield; 87 mg of **5** was isolated as a yellow solid.

^{1}H NMR (400 MHz, *d_6*-DMSO): δ 10.88 (br. s, 1H), 8.13 (ddd, *J* = 8.6, 1.6, 0.8 Hz, 1H), 7.87 (dd, *J* = 8.6, 6.8 Hz, 1H), 7.84 (q, *J* = 1.1 Hz, 1H), 7.82 (ddd, *J* = 6.8, 1.6, 1.0 Hz, 1H), 2.85 (s, 3H), 2.73 (d, *J* = 1.1 Hz, 3H).

^{13}C NMR (100 MHz, *d_6*-DMSO): δ 148.4, 141.2, 140.9, 140.7, 137.4, 135.2, 133.2, 131.1, 130.1, 128.0, 126.7, 104.6, 17.0, 16.0.

HRMS (ESI): calc. for $C_{14}H_{12}BrN_2O$ [M+H]$^+$: 303.0128, found: 303.0139.

MP: 204 - 206 °C.

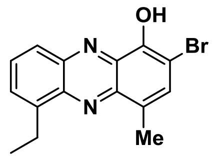

6

Yield: 64% yield; 48 mg of **6** was isolated as a yellow solid.

^{1}H NMR (400 MHz, $CDCl_3$): δ 8.33 (br. s, 1H), 8.05 (dd, *J* = 8.7, 1.3 Hz, 1H), 7.78 (dd, *J* = 8.7, 6.8 Hz, 1H), 7.71 – 7.66 (m, 2H), 3.43 (q, *J* = 7.5 Hz, 2H), 2.80 (d, *J* = 1.2 Hz, 3H), 1.46 (t, *J* = 7.5 Hz, 3H).

^{13}C NMR (100 MHz, $CDCl_3$): δ 147.1, 144.3, 142.0, 141.2, 141.1, 134.0, 133.0, 131.2, 129.6, 128.3, 126.7, 103.0, 24.7, 16.6, 14.9.

HRMS (ESI): calc. for $C_{15}H_{14}BrN_2O$ [M+H]$^+$: 317.0284, found: 317.0279.

MP: 132 - 134 °C.

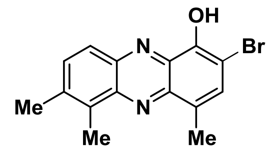

7

Yield: 41% yield; 12 mg of **7** was isolated as a yellow solid.

^{1}H NMR (400 MHz, *d_6*-DMSO): δ 10.79 (s, 1H), 8.05 (d, *J* = 9.0 Hz, 1H), 7.81 (d, *J* = 9.0 Hz, 1H), 7.81 (m, 1H), 2.79 (s, 3H), 2.73 (d, *J* = 0.8 Hz, 3H), 2.56 (s, 3H).

^{13}C NMR (100 MHz, *d_6*-DMSO): δ 148.4, 141.0, 140.7, 139.7, 138.3, 134.8, 134.4, 133.8, 132.9, 128.0, 125.6, 104.0, 20.3, 16.0, 12.8.

HRMS (ESI): calc. for $C_{15}H_{14}BrN_2O$ [M+H]$^+$: 317.0284, found: 317.0292.

MP: 207 - 209 °C.

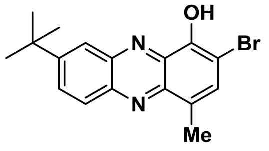

8

Yield: 42% yield; 40 mg of **8** was isolated as a yellow-green solid.

^{1}H NMR (400 MHz, $CDCl_3$): δ 8.36 (br. s, 1H), 8.15 (d, *J* = 9.2 Hz, 1H), 8.05 (d, *J* = 2.1 Hz, 1H), 7.94 (dd, *J* = 9.2, 2.1 Hz, 1H), 7.56 (m, 1H), 2.73 (d, *J* = 1.2 Hz, 3H), 1.49 (s, 9H).

^{13}C NMR (100 MHz, $CDCl_3$): δ 154.6, 147.1, 142.1, 141.8, 141.1, 134.5, 132.9, 130.5, 129.5, 129.1, 123.4, 103.0, 35.7, 30.9, 16.8.

HRMS (ESI): calc. for $C_{17}H_{18}BrN_2O$ [M+H]$^+$: 345.0579, found: 345.0613.

MP: 206 - 208 °C.

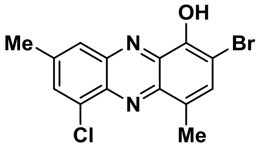

9

Yield: 55% yield; 12 mg of **9** was isolated as a yellow solid.

^{1}H NMR (400 MHz, d_6-DMSO): δ 10.96 (s, 1H), 8.09 (d, *J* = 1.5 Hz, 1H), 8.02 (m, 1H), 7.90 (m, 1H), 2.74 (s, 3H), 2.64 (s, 3H).

^{1}H NMR (400 MHz, $CDCl_3$): δ 8.21 (br. s, 1H), 7.90 (dq, *J* = 2.0, 1.1 Hz, 1H), 7.83 (d, *J* = 2.0 Hz, 1H), 7.70 (q, *J* = 1.2 Hz, 1H), 2.83 (d, *J* = 1.2 Hz, 3H), 2.64 (d, *J* = 1.1 Hz, 3H).

^{13}C NMR (100 MHz, d_6-DMSO): δ 148.4, 141.6, 141.4, 141.0, 137.0, 135.8, 133.7, 133.1, 131.7, 128.1, 126.5, 105.7, 21.6, 16.0.

^{13}C NMR (100 MHz, $CDCl_3$): δ 147.0, 141.7, 141.6, 138.6, 134.8, 133.8, 133.6, 133.0, 129.9, 126.5, 104.3, 22.4, 16.6. **Note:** One ^{13}C signal missing, likely due to overlap.

HRMS (DART): calc. for $C_{14}H_{11}BrClN_2O$ [M+H]$^+$: 336.9738, found: 336.9736.

MP: 234 - 236 °C.

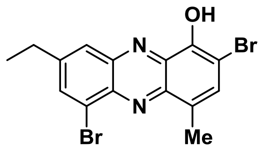

10

Yield: 99% yield; 72 mg of **10** was isolated as a yellow solid.

^{1}H NMR (400 MHz, $CDCl_3$): δ 8.25 (dt, *J* = 2.1, 0.6 Hz, 1H), 8.20 (s, 1H), 7.73 (dt, *J* = 2.1, 1.0 Hz, 1H), 7.69 (q, *J* = 1.1 Hz, 1H), 3.40 (m, 2H), 2.78 (d, *J* = 1.1 Hz, 3H), 1.45 (t, *J* = 7.5 Hz, 3H).

^{13}C NMR (100 MHz, $CDCl_3$): δ 147.0, 146.1, 141.5, 141.1, 140.7, 134.4, 133.6, 132.0, 129.7, 128.7, 126.1, 104.1, 24.5, 16.5, 14.7.

HRMS (ESI): calc. for $C_{15}H_{13}Br_2N_2O$ [M+H]$^+$: 394.9389, found: 394.9400.

MP: 189 - 191 °C.

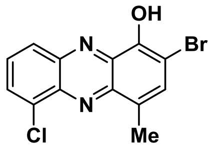

11

Yield: 64% yield; 64 mg of **11** was isolated as a yellow solid.

^{1}H NMR (400 MHz, d_6-DMSO): δ 11.07 (s, 1H), 8.28 (dd, *J* = 8.7, 1.2 Hz, 1H), 8.19 (dd, *J* = 7.4, 1.2 Hz, 1H), 7.94 (dd, *J* = 8.7, 7.4 Hz, 1H), 7.93 (m, 1H), 2.75 (d, *J* = 1.1 Hz, 3H).

^{13}C NMR (100 MHz, d_6-DMSO): δ 148.5, 141.5, 141.4, 138.2, 135.9, 134.3, 132.2, 130.8, 130.6, 128.4, 128.1, 105.7, 16.0.

HRMS (DART): calc. for $C_{13}H_9BrClN_2O$ [M+H]$^+$: 322.9581, found: 322.9584.

MP: 219 - 221 °C.

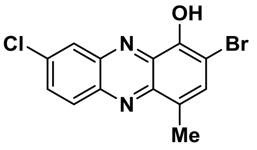

12

Yield: 72% yield; 85 mg of **12** was isolated as a yellow solid.

^{1}H NMR (400 MHz, d_6-DMSO): δ 10.98 (s, 1H), 8.27 (d, *J* = 2.4 Hz, 1H), 8.26 (d, *J* = 9.3 Hz, 1H), 7.96 (dd, *J* = 9.3, 2.4 Hz, 1H), 7.87 (q, *J* = 1.1 Hz, 1H), 2.69 (d, *J* = 1.1 Hz, 3H).

^{13}C NMR (100 MHz, d_6-DMSO): δ 148.5, 141.9, 140.7, 140.5, 135.9, 135.7, 134.0, 132.0, 131.6, 128.0, 127.2, 105.6, 16.2.

HRMS (ESI): calc. for $C_{13}H_9BrClN_2O$ [M+H]$^+$: 322.9581, found: 322.9596.

MP: Decom. 225 °C.

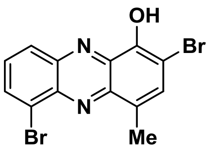

13

Yield: 88% yield; 88 mg of **13** was isolated as a yellow solid.

^{1}H NMR (400 MHz, d_6-DMSO): δ 11.06 (s, 1H), 8.38 (dd, *J* = 7.3, 1.2 Hz, 1H), 8.31 (dd, *J* = 8.7, 1.2 Hz, 1H), 7.93 (q, *J* = 1.1 Hz, 1H), 7.87 (dd, *J* = 8.7, 7.3 Hz, 1H), 2.75 (d, *J* = 1.1 Hz, 3H).

^{13}C NMR (100 MHz, d_6-DMSO): δ 148.5, 141.7, 141.4, 138.9, 135.9, 134.3, 134.2, 131.4, 129.1, 128.0, 123.9, 105.8, 15.9.

HRMS (DART): calc. for $C_{13}H_9Br_2N_2O$ [M+H]$^+$: 366.9076, found: 366.9080.

MP: 209 - 211 °C.

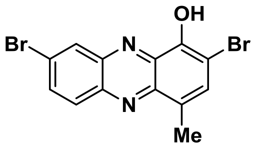

14

Yield: 40% yield; 23 mg of **14** was isolated as a yellow solid.

^{1}H NMR (400 MHz, *d*$_6$-DMSO): δ 8.44 (m, 1H), 8.19 (d, *J* = 9.2 Hz, 1H), 8.06 (ddd, *J* = 9.3, 2.2, 0.6 Hz, 1 H), 7.88 (m, 1H), 2.70 (s, 3H).

^{13}C NMR (100 MHz, *d*$_6$-DMSO): δ 148.5, 141.9, 141.0, 140.5, 135.8, 134.3, 134.0, 131.4, 130.6, 128.0, 124.6, 105.6, 16.2.

HRMS (ESI): calc. for $C_{13}H_9Br_2N_2O$ [M+H]$^+$: 366.9076, found: 366.9061.

MP: Decom. 215 °C.

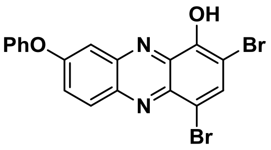

16

Yield: 48% yield; 78 mg of **16** was isolated as a yellow solid.

^{1}H NMR (400 MHz, *d*$_6$-DMSO): δ 11.26 (s, 1H), 8.40 – 8.29 (m, 2H), 7.96 (dd, *J* = 9.5, 2.8 Hz, 1H), 7.60 (dd, *J* = 8.0, 7.7 Hz, 2H), 7.43 – 7.34 (m, 3H), 7.26 (d, *J* = 2.8 Hz, 1H).

^{13}C NMR (100 MHz, *d*$_6$-DMSO): δ 161.0, 154.0, 150.4, 142.4, 140.2, 138.3, 135.7, 135.3, 131.5, 130.7, 126.9, 126.0, 121.3, 111.5, 108.6, 104.6.

HRMS (ESI): calc. for $C_{18}H_{11}Br_2N_2O_2$ [M+H]$^+$: 446.9162, found: 446.9161.

MP: Decom. 203 °C.

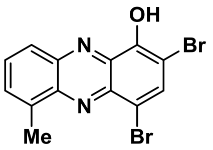

17

Yield: 81% yield; 220 mg of **17** was isolated as a yellow solid.

^{1}H NMR (400 MHz, *d*$_6$-DMSO): δ 11.51 (s, 1H), 8.40 (s, 1H), 8.18 (m, 1H), 7.93 (dd, *J* = 8.0, 6.8 Hz, 1H), 7.89 (m, 1H), 2.88 (s, 3H).

^{13}C NMR (100 MHz, *d*$_6$-DMSO): δ 150.8, 142.1, 141.5, 138.5, 137.4, 136.5, 135.1, 132.0, 131.1, 126.7, 111.9, 104.4, 16.9.

HRMS (DART): calc. for $C_{13}H_9Br_2N_2O$ [M+H]$^+$: 366.9076, found: 366.9087.

MP: 214 - 216 °C.

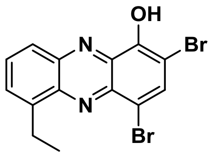

18

Yield: 38% yield; 51 mg of **18** was isolated as a yellow solid.

^{1}H NMR (400 MHz, *d*$_6$-DMSO): δ 11.52 (br. s, 1H), 8.41 (s, 1H), 8.20 (dd, *J* = 8.7, 1.5 Hz, 1H), 7.97 (dd, *J* = 8.7, 6.9 Hz, 1H), 7.89 (m, 1H), 3.39 (q, *J* = 7.5 Hz, 2H), 1.43 (t, *J* = 7.5 Hz, 3H).

^{13}C NMR (100 MHz, *d*$_6$-DMSO): δ 150.8, 143.1, 141.5, 141.5, 138.5, 136.4, 135.1, 132.2, 129.6, 126.7, 111.9, 104.4, 23.8, 14.7.

HRMS (ESI): calc. for $C_{14}H_{11}Br_2N_2O$ [M+H]$^+$: 382.9213, found: 382.9220.

MP: 145 - 147 °C.

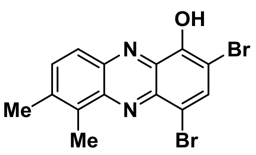

19

Yield: 30% yield; 71 mg of **19** was isolated as a yellow solid.

^{1}H NMR (400 MHz, *d*$_6$-DMSO): δ 11.43 (s, 1H), 8.35 (s, 1H), 8.06 (d, *J* = 8.9 Hz, 1H), 7.83 (d, *J* = 8.9 Hz, 1H), 2.80 (s, 3H), 2.55 (s, 3H).

^{13}C NMR (100 MHz, *d*$_6$-DMSO): δ 150.7, 141.8, 140.2, 139.4, 138.4, 136.2, 135.7, 134.3, 133.8, 125.5, 111.9, 103.8, 20.3, 12.8.

HRMS (ESI): calc. for $C_{14}H_{10}Br_2N_2ONa$ [M+Na]$^+$: 404.9032, found: 404.9026.

MP: 212 - 214 °C.

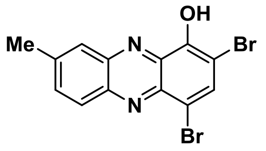

20

Yield: 77% yield; 77 mg of **20** was isolated as a yellow solid.

^{1}H NMR (400 MHz, d_6-DMSO): 11.46 (s, 1H), 8.38 (s, 1H), 8.22 (dt, J = 8.9, 0.5 Hz, 1H), 8.09 (ddd, J = 1.9, 1.2, 0.7 Hz, 1H), 7.91 (ddd, J = 8.9, 1.9, 0.5 Hz, 1H), 2.67 (d, J = 1.1 Hz, 3H).

^{13}C NMR (100 MHz, d_6-DMSO): δ 150.8, 142.9, 141.7, 141.5, 139.0, 136.2, 135.4, 135.1, 128.9, 126.8, 111.5, 104.3, 21.8.

HRMS (ESI): calc. for $C_{13}H_9Br_2N_2O$ $[M+H]^+$: 368.9056, found: 368.9055.

MP: 213 - 215 °C.

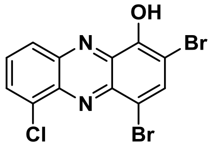

21

Yield: 42% yield; 55 mg of **21** was isolated as a yellow solid.

^{1}H NMR (400 MHz, d_6-DMSO): δ 11.69 (s, 1H), 8.50 (s, 1H), 8.33 (dd, J = 8.8, 1.2 Hz, 1H), 8.26 (dd, J = 7.4, 1.2 Hz, 1H), 8.00 (dd, J = 8.8, 7.4 Hz, 1H).

^{13}C NMR (100 MHz, d_6-DMSO): δ 150.9, 142.0, 139.4, 139.3, 137.6, 135.9, 132.1, 131.7, 131.5, 128.4, 111.7, 105.4.

HRMS (DART): calc. for $C_{12}H_6Br_2ClN_2O$ $[M+H]^+$: 386.8530, found: 386.8521.

MP: 230 - 232 °C.

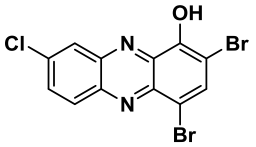

22

Yield: 31% yield; 37 mg of **22** was isolated as a yellow solid.

^{1}H NMR (400 MHz, d_6-DMSO): δ 11.62 (s, 1H), 8.45 (s, 1H), 8.42 – 8.30 (m, 2H), 8.05 (dd, J = 9.2, 2.4 Hz, 1H).

^{13}C NMR (100 MHz, d_6-DMSO): δ 150.9, 141.4, 141.2, 139.6, 137.3, 136.7, 135.9, 132.9, 131.4, 127.1, 111.5, 105.3.

HRMS (DART): calc. for $C_{12}H_6Br_2ClN_2O$ $[M+H]^+$: 386.8530, found: 386.8524.

MP: 245 - 247 °C.

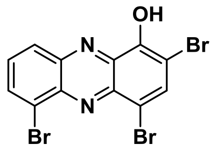

23

Yield: 40% yield; 41 mg of **23** was isolated as a yellow solid.

^{1}H NMR (400 MHz, d_6-DMSO): δ 11.69 (s, 1H), 8.50 (s, 1H), 8.45 (dd, J = 7.3, 1.2 Hz, 1H), 8.36 (dd, J = 8.8, 1.2 Hz, 1H), 7.94 (dd, J = 8.8, 7.3 Hz, 1H).

^{13}C NMR (100 MHz, d_6-DMSO): δ 150.8, 141.9, 140.0, 139.6, 137.6, 135.9, 135.1, 132.3, 129.0, 123.6, 111.6, 105.4.

HRMS (DART): calc. for $C_{12}H_6Br_3N_2O$ $[M+H]^+$: 432.8005, found: 432.8004.

MP: 205 - 207 °C.

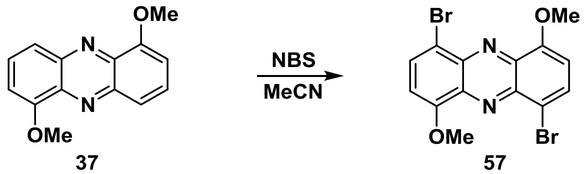

Synthesis of 57: To an 8 mL sealed microwave vial was added **37** (121 mg, 0.50 mmol) in acetonitrile (4 mL). The resulting mixture was then heated at 80 °C in the microwave reactor for 12 minutes. The solvent was

removed *in vacuo* and the resulting solid was purified via column chromatography using dichloromethane to elute, affording **57**, which was isolated as a yellow solid (99%, 198 mg).

^{1}H NMR (400 MHz, $CDCl_3$): δ 8.12 (d, *J* = 8.3 Hz, 2H), 7.02 (d, *J* = 8.3 Hz, 2H), 4.17 (s, 6H).

^{13}C NMR (100 MHz, $CDCl_3$): δ 154.4, 139.2, 136.5, 133.1, 113.3, 107.3, 56.1.

HRMS (ESI): calc. for $C_{14}H_{11}Br_2N_2O_2$ $[M+H]^+$: 398.9162, found: 398.9153.

MP: > 260 °C.

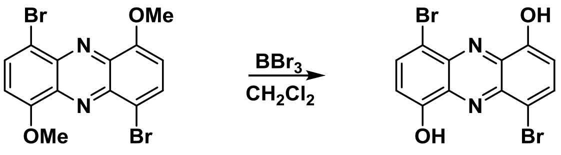

Procedure for the synthesis of 58: To a round bottom flask was added **57** (160 mg, 0.40 mmol) dissolved in anhydrous dichloromethane (20 mL). The mixture was brought to -78 °C in a dry ice bath before dropwise addition of 1 M boron tribromide solution in dichloromethane (4.02 mL, 4.02 mmol). The reaction was left to stir at -78 °C for 1 hour, and then allowed to reach ambient temperature for reaction overnight. The reaction was heated to reflux for 8 hours until complete (monitored by TLC). Brine (20 mL) was then added to the mixture to quench the reaction. The mixture was then transferred to a separatory funnel, and then extracted with dichloromethane. Organic extracts were dried with sodium sulfate, filtered through cotton, and removed *in vacuo*. The resulting crude product was purified via column chromatography using dichloromethane to elute **58** as a red solid (100%, 149 mg).

^{1}H NMR (400 MHz, *d*$_6$-DMSO): δ 10.78 (br. s, 2H), 8.16 (d, *J* = 8.2 Hz, 2H), 7.21 (d, *J* = 8.2 Hz, 2H).

^{13}C NMR (100 MHz, *d*$_6$-DMSO): δ 153.6, 139.2, 136.3, 134.7, 111.8, 110.7.

HRMS (DART): calc. for $C_{12}H_7N_2O_2Br_2$ $[M+H]^+$: 368.8869, found: 368.8860.

MP: > 260 °C.

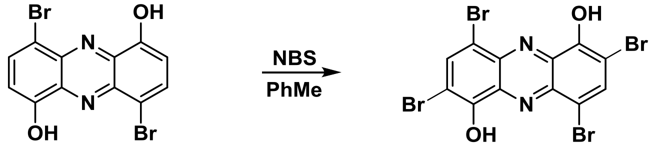

Procedure for the synthesis of 15: 58 (30.0 mg, 0.08 mmol) and *N*-bromosuccinimide (43.7 mg, 0.18 mmol) were suspended in 1 mL toluene and allowed to stir at room temperature for 1 hour. The reaction contents were filtered. The filtrate was washed with dichloromethane (6 mL), resulting product **15** as a dark red solid (60%, 26 mg).

^{1}H NMR (400 MHz, *d*$_6$-DMSO): δ 11.07 (s, 2H), 8.51 (s, 2H).

^{13}C NMR (100 MHz, *d*$_6$-DMSO): δ 150.6, 138.5, 137.8, 135.8, 111.6, 106.6.

HRMS (DART): calc. for $C_{12}H_5Br_4N_2O_2$ $[M+H]^+$: 524.7079, found: 524.7058.

MP: > 260 °C.

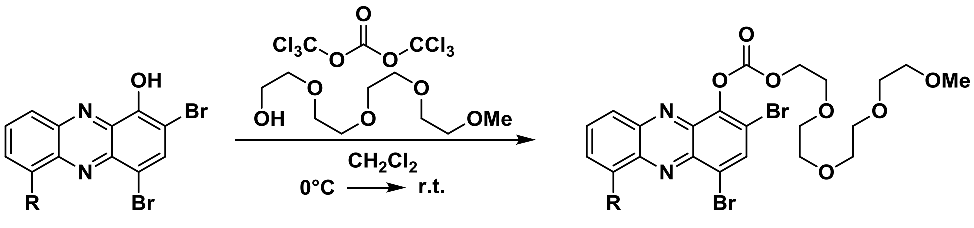

General Procedure for the Synthesis of HP-Carbonate (24 and 25) Tetraethyleneglycol monomethyl ether (69 µL 0.33 mmol) was placed in an oven-dried round-bottomed flask and dissolved in anhydrous dichloromethane (1 mL). The solution was then cooled to 0 °C. Pyridine (37 µL, 0.47 mmol) and triethylamine (11 µL 0.73 mmol) was then added via syringe, followed by triphosgene (48.2 mg, 0.16 mmol) dissolved in dichloromethane (1mL). The resulting mixture was stirred from 0 °C to room temperature and continued to stir at room temperature for 5 hours. After that, then the reaction was cooled to 0 °C before the addition of solution of **17** (86 mg, 0.23 mmol) and triethylamine (49 µL 0.35 mmol) in anhydrous dichloromethane was added to the reaction in dropwise. The reaction solution was stirred for 5 min at 0 °C and then reach ambient temperature and stirred at room temperature overnight. After the reaction was complete, the reaction mixture was poured into a separatory funnel containing 1 M ammonium chloride (20 mL), and the biphasic mixture was shaken vigorously. Upon separation of layers, the aqueous layer was re-extracted with dichloromethane (2 × 30 mL). Organic extracts were collected, dried over Sodium Sulfate, filtered, and concentrated under vacuum. The resulting crude material was purified using flash column chromatography with 3:1 hexanes:ethyl acetate to 100% ethyl acetate as eluent yield **25** as a yellow oil (135 mg, 96%).

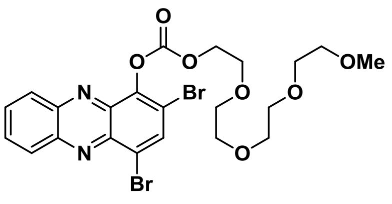

24

Yield: 80% yield; 92 mg of **24** was isolated as a yellow oil.

^{1}H NMR (400 MHz, CDCl$_3$): δ 8.35 (m, 1H), 8.34 (s, 1H), 8.28 (m, 1H), 7.96 – 7.88 (m, 2H), 4.53 (m, 2H), 3.88 (m, 2H), 3.73 (m, 2H), 3.71 – 3.62 (m, 8H), 3.54 (m, 2H), 3.37 (s, 3H).

^{13}C NMR (100 MHz, CDCl$_3$): δ 152.3, 144.6, 143.7, 143.5, 140.2, 137.6, 135.8, 132.5, 132.1, 130.2, 130.0, 122.6, 116.8, 72.1, 71.0, 70.9, 70.8, 70.8, 70.7, 69.0, 68.9, 59.2.

HRMS (ESI): calc. for $C_{22}H_{25}Br_2N_2O_7$ [M+H]$^+$: 589.0004, found: 588.9998.

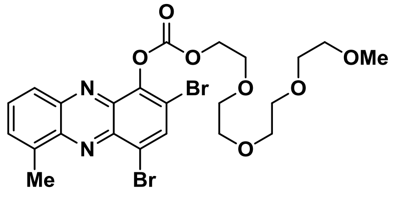

25

Yield: 96% yield; 135 mg of **25** was isolated as a yellow oil.

^{1}H NMR (400 MHz, CDCl$_3$): δ 8.33 (s, 1H), 8.11 (ddd, *J* = 8.7, 1.5, 0.8 Hz, 1H), 7.80 (dd, *J* = 8.7, 6.8 Hz, 1H), 7.74 (m, 1H), 4.53 (m, 2H), 3.88 (m, 2H), 3.73 (m, 2H), 3.71 – 3.61 (m, 8H), 3.55 (m, 2H), 3.37 (s, 3H), 2.97 (s, 3H).

^{13}C NMR (100 MHz, CDCl$_3$): δ 152.3, 144.3, 143.6, 143.2, 139.2, 138.7, 137.1, 135.2, 132.4, 131.1, 127.6, 123.2, 116.5, 72.1, 71.0, 70.8, 70.8, 70.8, 70.7, 68.9, 68.9 (determined by HSQC), 59.2, 17.5. **Note:** See spectra section for HSQC of **25**.

HRMS (ESI): calc. for $C_{23}H_{27}Br_2N_2O_7$ [M+H]$^+$: 603.0161, found: 603.0164.

3.) Biological Methods:

A.) Minimum Inhibitory Concentration (MIC) Susceptibility Assay (in 96-well plate):

The minimum inhibitory concentration (MIC) for each test compound was determined by the broth microdilution method as recommended by the Clinical and Laboratory Standards Institute (CLSI). In a 96-well plate, eleven two-fold serial dilutions of each compound were made in a final volume of 100 µL Luria Broth. Each well was inoculated with ~10^5 bacterial cells at the initial time of incubation, prepared from a fresh log phase culture (OD_{600} of 0.5 to 1.0 depending on bacterial strain). The MIC was defined as the lowest concentration of compound that prevented bacterial growth after incubating 16 to 18 hours at 37 °C (MIC values were supported by spectrophotometric readings at OD_{600}). The concentration range tested for each test compound during this study was 0.10 to 100 µM. DMSO served as our vehicle and negative control in each microdilution MIC assay. DMSO was serially diluted with a top concentration of 1% v/v. All compounds were tested in a minimum of three independent experiments.

B.) MIC Assay with Metal(II) Cation Co-Treatment:

Metal(II) cation studies were performed in a similar setup to the standard MIC assay, with the addition of 200 µM of the metal(II) cation (i.e., copper(II) sulfate) to the media.[3] All data were obtained from three independent experiments.

C.) MIC Assay for *Mycobacterium tuberculosis*:

M. tuberculosis H37Ra (ATCC 25177) was inoculated in 10 mL Middlebrook 7H9 medium and allowed to grow for two weeks. The culture was then diluted with fresh medium until an OD_{600} of 0.01 was reached. Aliquots of 200 µL were then added to each well of a 96-well plate starting from the second column. Test compounds were dissolved in DMSO at final concentration of 10 mM. 7.5 µL of each compound along with DMSO (negative control) and streptomycin (positive control-40mg/ml stock solution) were added to 1.5 mL of the Mycobacterium diluted cultures, resulting in 50 µM final concentration of each halogenated phenazine analogues and 340 µM for streptomycin. The final DMSO concentration was maintained at 0.5%. Aliquots of 400 µl were added to wells of the first column of the 96-well plate and serially diluted two-fold (200 µl) per well across the plate to obtain final concentrations that ranges from 0.024 to 50 µM for the test compounds and 0.16 to 340 µM for streptomycin. Three rows were reserved for each compound. The plates were then incubated at 37°C for seven days. Minimum inhibitory concentrations are reported as the lowest concentration at which no bacterial growth was observed. OD_{600} absorbance was recorded using SpectraMax M5 (Molecular Devices). Data obtained from three independent experiments were analyzed using Excel.

D.) Calgary Biofilm Device (CBD) Experiments

Minimum Bactericidal Concentrations (MBC) and Minimum Biofilm Eradication Concentrations (MBEC) Determination

Biofilm eradication experiments were performed using the Calgary Biofilm Device to determine MBC/MBEC values for various compounds of interest (Innovotech, product code: 19111). The Calgary device (96-well plate with lid containing pegs to establish biofilms on) was inoculated with 125 µL of a mid-log phase culture diluted 1,000-fold in tryptic soy broth with 0.5% glucose (TSBG) to establish bacterial biofilms after incubation at 37 °C for 24 hours. The lid of the Calgary device was then removed, washed and transferred to another 96-well plate containing 2-fold serial dilutions of the test compounds (the "challenge plate"). The total volume of media with compound in each well in the challenge plate is 150 µL. The Calgary device was then incubated at 37 °C

for 24 hours. The lid was then removed from the challenge plate and MBC/MBEC values were determined using different experimental pathways. To determine **MBC values**, 20 µL of the challenge plate was transferred into a fresh 96-well plate containing 180 µL TSBG and incubated overnight at 37 °C. The MBC values were determined as the concentration giving a lack of visible bacterial growth (i.e., turbidity). For determination of **MBEC values**, the Calgary device lid (with attached pegs/treated biofilms) was transferred to a new 96-well plate containing 150 µL of fresh TSBG media in each well and incubated for 24 hours at 37 °C to allow viable biofilms to grow and disperse resulting in turbidity after the incubation period. MBEC values were determined as the lowest test concentration that resulted in eradicated biofilm (i.e., wells that had no turbidity after final incubation period). All data were obtained from a minimum of three independent experiments.

Pulse experiments followed a normal CBD assay protocol; however, the compound treatment phase (the “challenge plate”) consisted of two sequential 24 hour compound treatment plates before the final recovery plate. Following this, CBD pegs were removed from the lid, sonicated for 30 minutes in PBS and plated out to determine biofilm cell killing in colony forming units per milliliter (CFU/mL).

Note: MRSA strain BAA-1707 and *S. epidermidis* (MRSE; ATCC 35984) and *E. faecium* (VRE; ATCC 700221) were tested using these parameters.

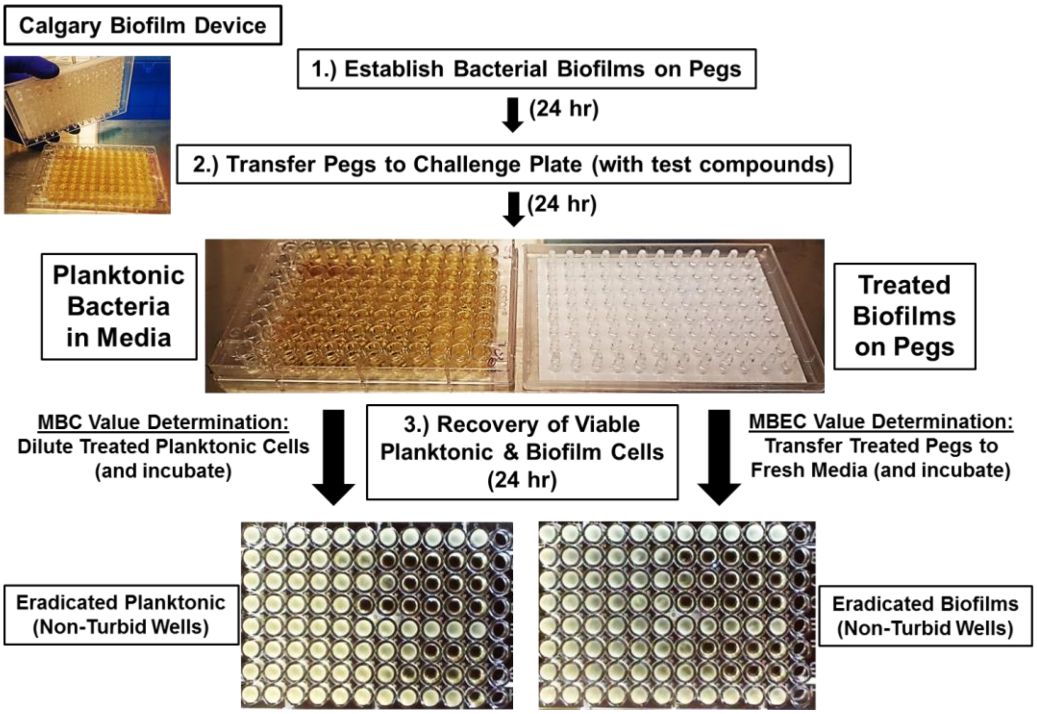

Work flow for the determination of MBC and MBEC values using the Calgary Biofilm Device.

E.) Live / Dead staining (Fluorescence Microscopy) of MRSA BAA-1707 Biofilms:

A mid-log culture of MRSA BAA-1707 was diluted 1:1,000-fold and 500 µL was transferred to each compartment of a 4 compartment CELLview dish (Greiner Bio-One 627871). The dish was then incubated for 24 hours at 37 °C. After this time, the cultures were removed and the plate was washed with 0.9% saline. The dish was then treated with the compounds in fresh media at various concentrations. DMSO was used as our negative control in this assay. The dish was incubated with the compound for 24 hours at 37 °C. After this time,

the cultures were removed and the dish was washed with 0.9% saline for 2 minutes. Saline was then removed and 500 µL of the stain (Live/Dead BacLight Viability Kit, Invitrogen) were added for 15 minutes and left in the dark. After this time, the stain was removed and the dish was washed twice with 0.9% saline. Then the dish was fixed with 500 µL 4% paraformaldehyde in PBS for 30 minutes. Images of remaining MRSA biofilms were then taken with a fluorescence microscope. All data were analyzed using Image J software from three independent experiments.

F.) Hemolysis Assay with Red Blood Cells:

As previously described, freshly drawn human red blood cells (hRBC with ethylenediaminetetraacetic acid (EDTA) as an anticoagulant) were washed with Tris-buffered saline (0.01M Tris-base, 0.155 M sodium chloride (NaCl), pH 7.2) and centrifuged for 5 minutes at 3,500 rpm. The washing was repeated three times with the buffer. In 96-well plate, test compounds were added to the buffer from DMSO stocks. Then 2% hRBCs (50 µL) in buffer were added to test compounds to give a final concentration of 200 µM. The plate was then incubated for 1 hour at 37 °C. After incubation, the plate was centrifuged for 5 minutes at 3,500 rpm. Then 80 µL of the supernatant was transferred to another 96-well plate and the optical density (OD) was read at 405 nm. DMSO served as our negative control (0% hemolysis) while Triton X served as our positive control (100% hemolysis). The percent of hemolysis was calculated as (OD_{405} of the compound- OD_{405} DMSO) / (OD_{405} Triton X- OD_{405} buffer) from three independent experiments.

G.) LDH Release Assay for HeLa Cytotoxicity Assessment:

HeLa cytotoxicity was assessed using the LDH release assay described by CytoTox96 (Promega G1780). HeLa cells were grown in Dulbecco's Modified Eagle Medium (DMEM; Gibco) supplemented with 10% Fetal Bovine Serum (FBS) at 37°C with 5% CO_2. When the HeLa cultures exhibited 70-80% confluence, halogenated phenazines were then diluted by DMEM (10% FBS) at concentrations of 25, 50 and 100 µM and added to HeLa cells. Triton X-100 (at 2% v/v) was used as the positive control for maximum lactate dehydrogenate (LDH) activity in this assay (i.e., complete cell death) while "medium only" lanes served as negative control lanes (i.e., no cell death). DMSO was used as our vehicle control. HeLa cells were treated with compounds for 24 hours and then 50 µL of the supernatant was transferred into a fresh 96-well plate where 50 µL of the reaction mixture was added to the 96-well plate and incubated at room temperature for 30 minutes. Finally, Stop Solution (50 µL) was added to the incubating plates and the absorbance was measured at 490 nm. Results are on the next page and are from three independent experiments.

Halogenated Phenazine cytotoxicity results (Triton-X = 100% cell death; Medium Only: 0% cell death):

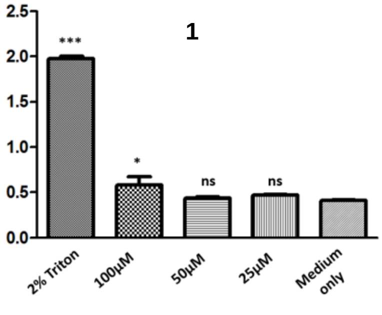

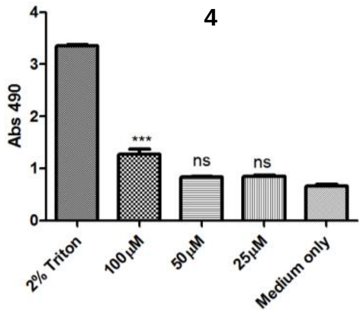

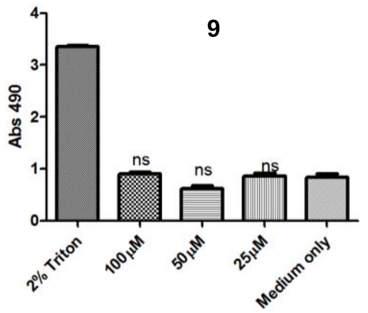

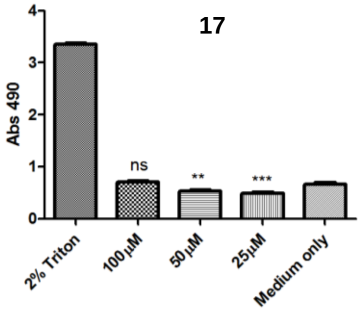

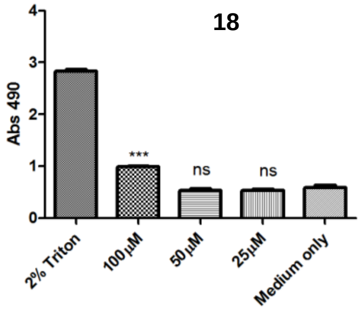

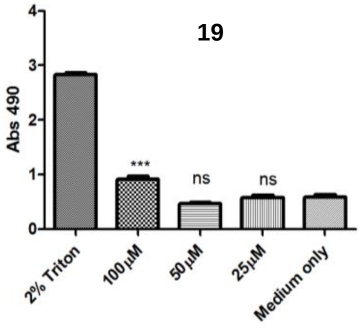

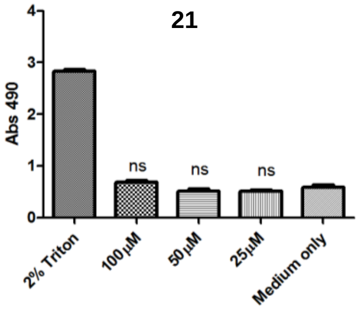

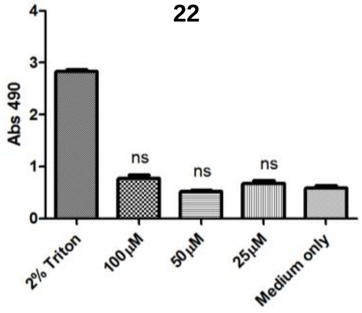

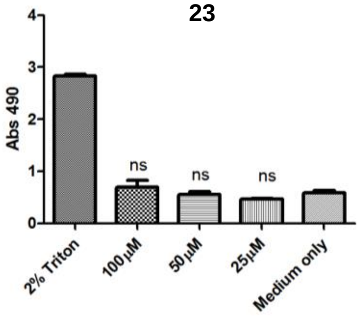

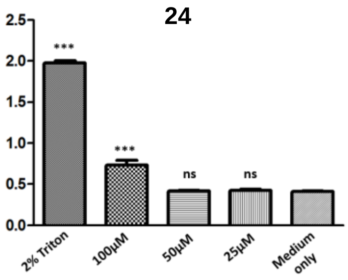

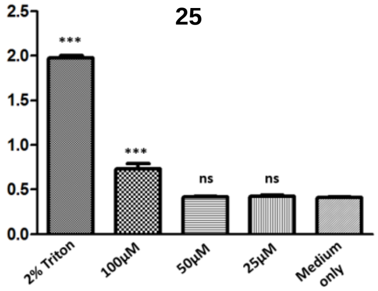

H.) Macromolecular Synthesis Inhibition Assay:

Macromolecular syntheses experiments were carried out in methicillin-resistant *Staphylococcus aureus* BAA-1707. An overnight culture (100 µL) of *S. aureus* BAA-1707 was sub-cultured into 10 mL of fresh TSBG media which was allowed to grow to exponential phase (OD_{600} = 0.2-0.3) before transferring 500 µL to each well in a 24 well-plate. The test compounds and vehicle control (DMSO) were added to achieve the desired concentrations relative to their MIC values against *S. aureus* BAA-1707. Treated cultures were then incubated at 37°C for 30 minutes before radioactive precursors for DNA ([^{3}H] thymidine (0.5 µCi)), RNA ([^{3}H] uridine (0.5 µCi)) and protein ([^{3}H] leucine (1 µCi)) were added. Antibiotics with known modes of action were used as positive controls in these experiments, these included: ciprofloxacin (DNA inhibition), rifampicin (RNA inhibition) and linezolid (protein inhibition). DMSO served as our negative control. DNA and RNA radiolabeled cultures were then incubated in 37°C for 15 minutes before being stopped by adding 60 µL of cold 5% trichloroacetic acid (TCA). The protein synthesis experiment was stopped after 40 minutes by adding 60 µL cold TCA. These mixtures were then incubated at 2°C for at least 30 minutes before the contents of the plates were transferred onto glass microfiber filters (24 mm) and washed 5 times with 1 mL of 5% TCA. The filters

are allowed to dry overnight before 3.5 mL of the scintillation fluid was added to the scintillation vials containing the filters and the radiation counts were measured using liquid scintillation LS 6500.

I.) UV-Vis Experiments to Demonstrate Direct Metal(II) Binding:

The rates of halogenated phenazine-copper(II) complex formation were independently evaluated via UV-Vis spectrometry following addition of 0.5 equivalents copper(II) sulfate to stirring solutions of **HP** (10 mM, 4 mL) in dimethyl sulfoxide. Spectral scanning was performed from 200 to 800 nm in 2 nm increments. The disappearance of HPs **1, 17** and **22** was observed over the indicated time points. The halogenated phenazine-copper(II) complex formation yielded a loss in absorbance due to precipitation. No change of the UV-Vis spectra was observed for **25** as a result of no metal(II) binding. 8-Hydroxyquinoline was also tested as a positive control.

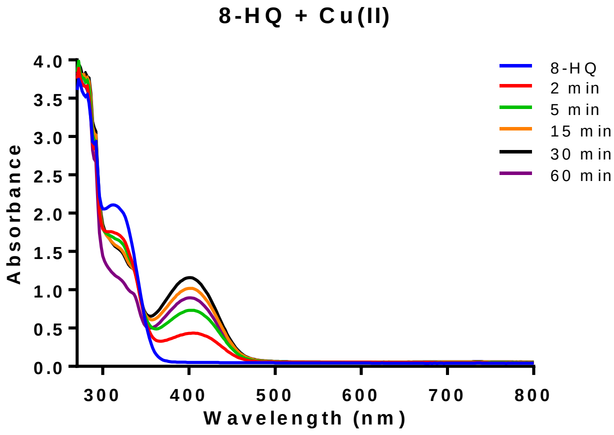

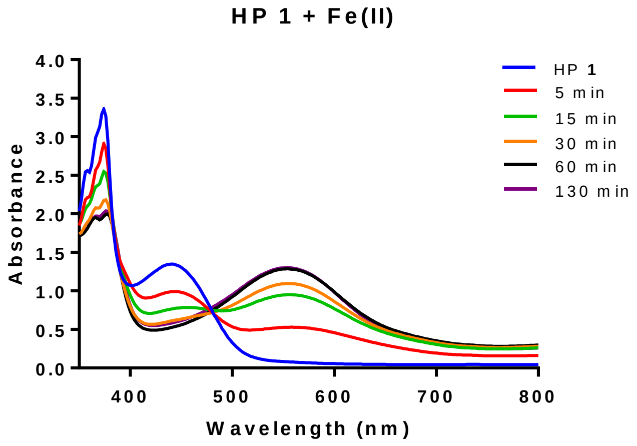

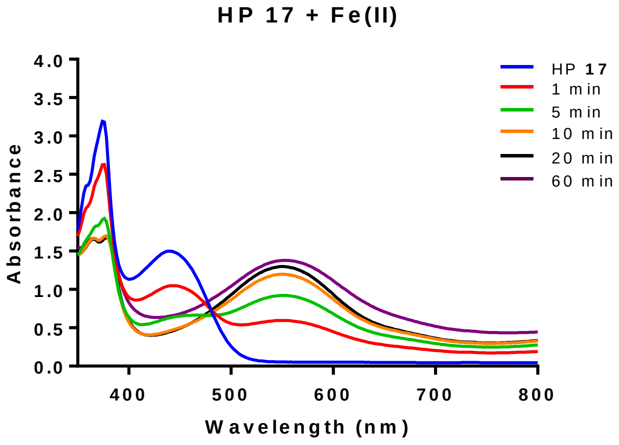

HP 21 + Fe(II)

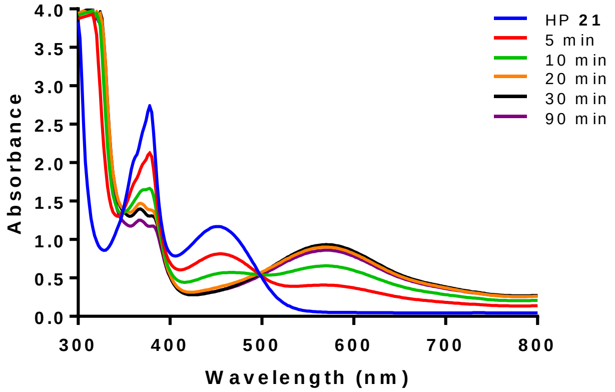

HP 25 + Fe(II)

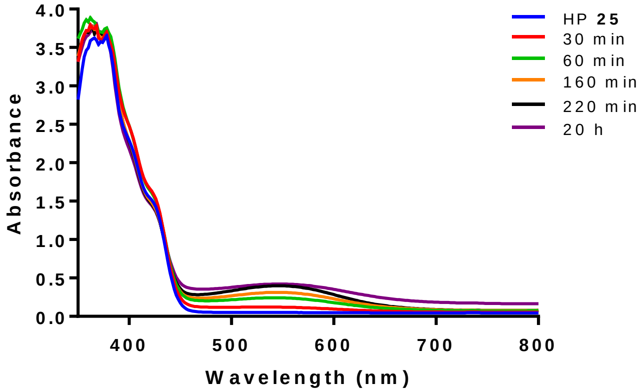

8-HQ + Fe(II)

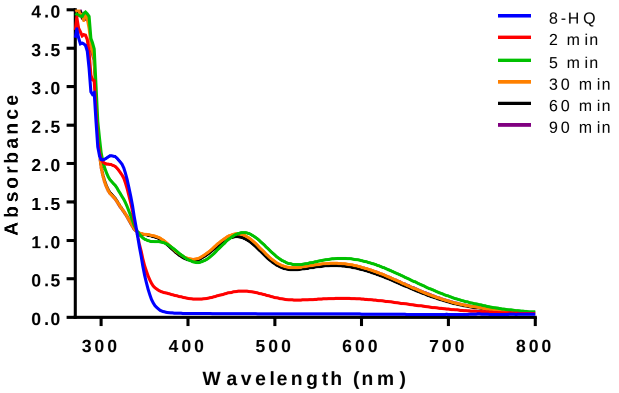

HP 1 + Zn(II)

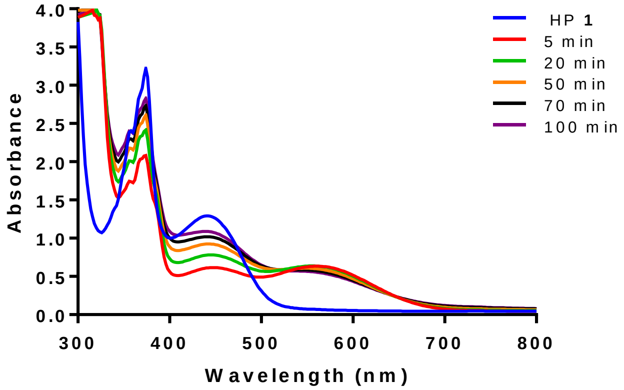

HP 17 + Zn(II)

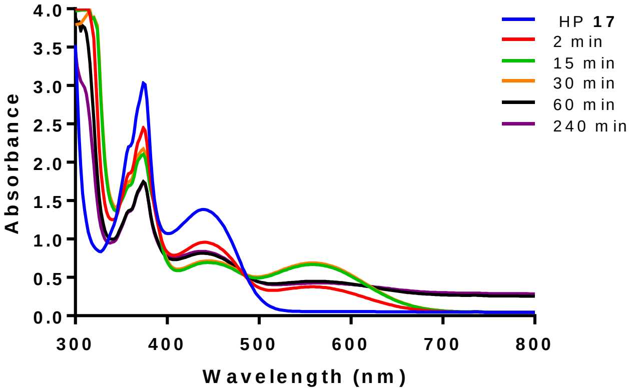

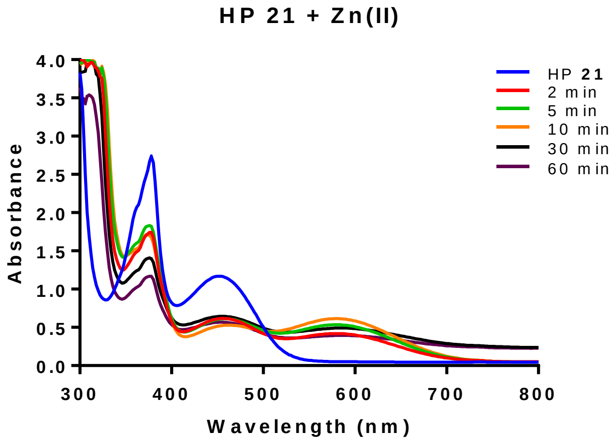
HP 21 + Zn(II)
Absorbance
Wavelength (nm)
HP 21
2 min
5 min
10 min
30 min
60 min

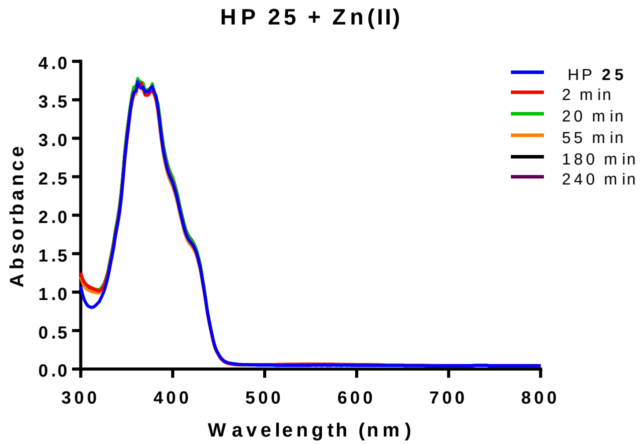
HP 25 + Zn(II)
Absorbance
Wavelength (nm)
HP 25
2 min
20 min
55 min
180 min
240 min

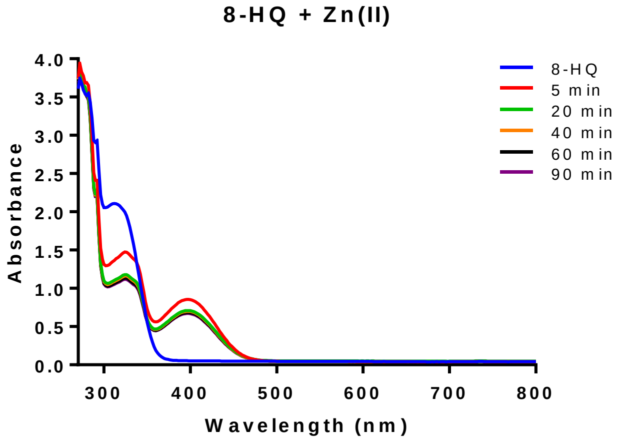
8-HQ + Zn(II)
Absorbance
Wavelength (nm)
8-HQ
5 min
20 min
40 min
60 min
90 min

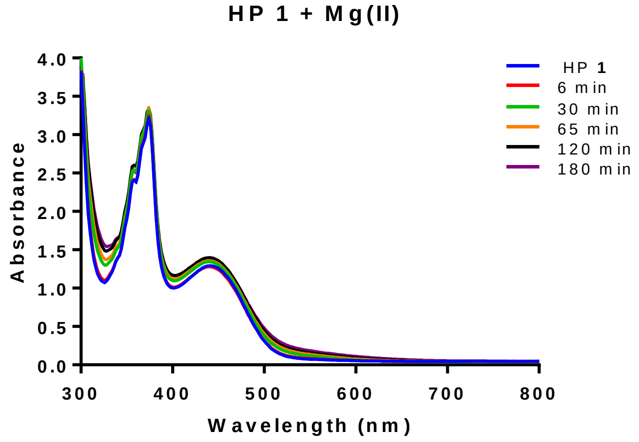
HP 1 + Mg(II)
Absorbance
Wavelength (nm)
HP 1
6 min
30 min
65 min
120 min
180 min

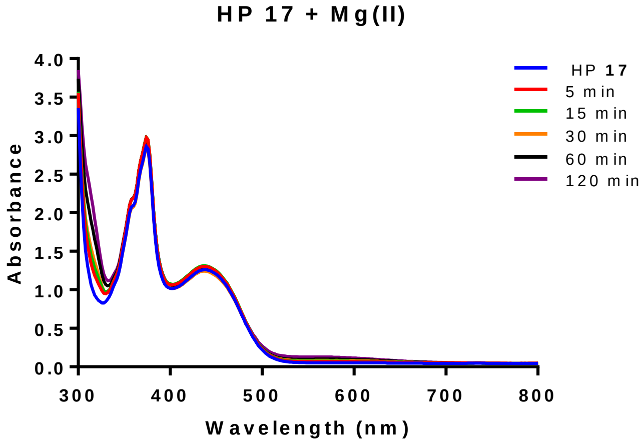
HP 17 + Mg(II)
Absorbance
Wavelength (nm)
HP 17
5 min
15 min
30 min
60 min
120 min

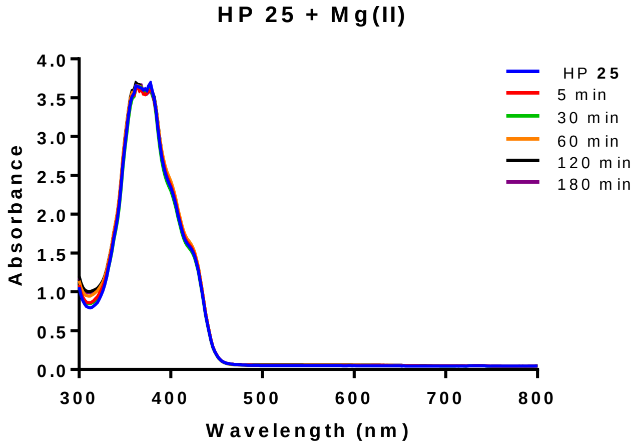
HP 25 + Mg(II)
Absorbance
Wavelength (nm)
HP 25
5 min
30 min
60 min
120 min
180 min

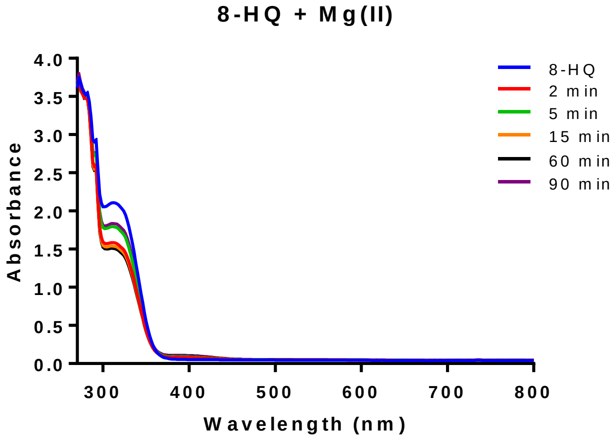

4.) Literature References:

1. Garrison, A. T.; Abouelhassan, Y.; Norwood IV, V. M.; Kallifidas, D.; Bai, F.; Nguyen, T. M.; Rolfe. M. A.; Burch. G. M.; Jin, S.; Luesch, H.; Huigens III, R. W. *J. Med. Chem.* **2016**, *59*, 3808 –3825.
2. Chowdhury, G.; Sarkar, U.; Pullen, S.; Wilson, W.; Rajapakse, A.; Fuchs-Knotts, T.; and Gates, K. S. *Chem. Res. Toxicol*. **2012**, *25*, 197 – 206.
3. Abouelhassan, Y.; Garrison, A. T.; Bai, F.; Norwood IV, V. M.; Nguyen, M. T.; Jin, S.; Huigens III, R. W. *ChemMedChem* **2015**, *10*, 1157-1162.

CBD Biofilm Eradication Assays against MRSA BAA-1707

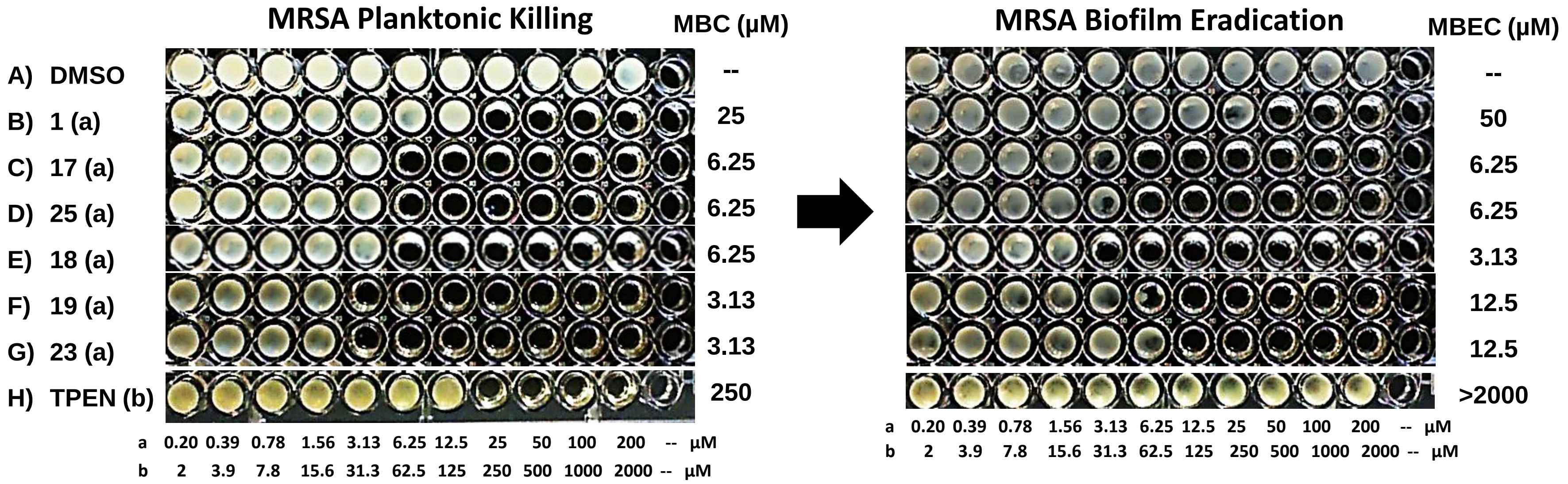

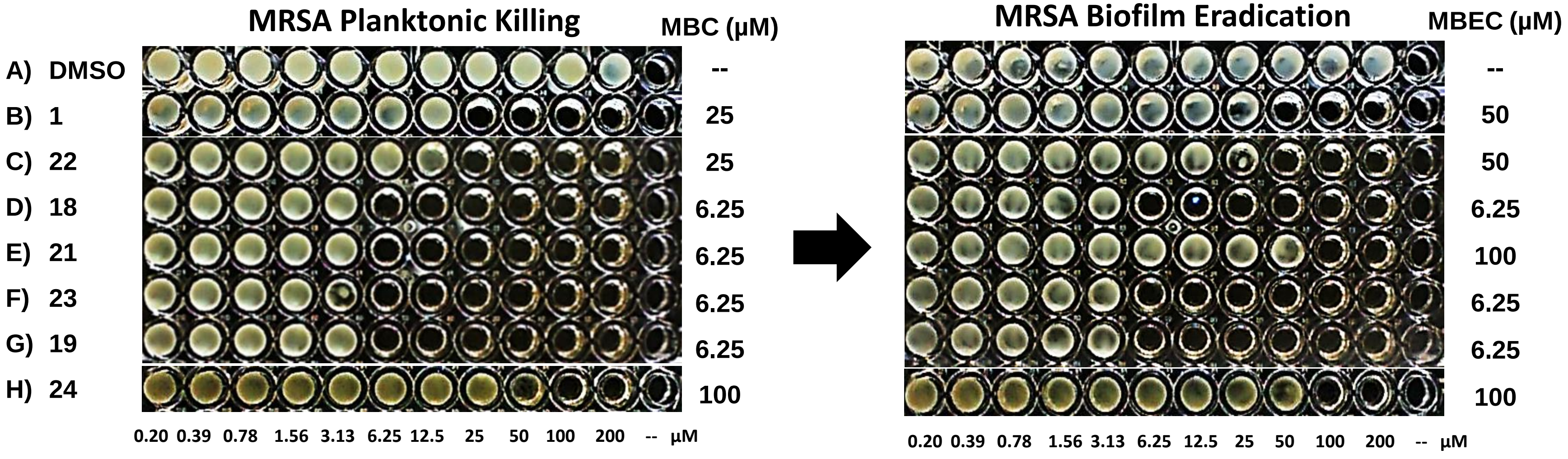

CBD Biofilm Eradication Assay against MRSE 35984

MRSE Planktonic Killing

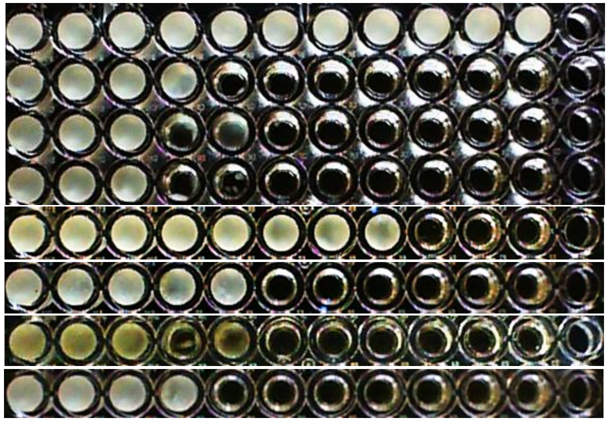

		MBC (µM)
A)	DMSO	--
B)	1(b)	15.6
C)	19(a)	6.25
D)	23(a)	6.25
E)	24(a)	50
F)	17(a)	6.25
G)	18(a)	6.25
H)	25(a)	3.13

a 0.20 0.39 0.78 1.56 3.13 6.25 12.5 25 50 100 200 -- µM

b 1 2 3.9 7.8 15.6 31.3 62.5 125 250 500 1000 -- µM

MRSE Biofilm Eradication

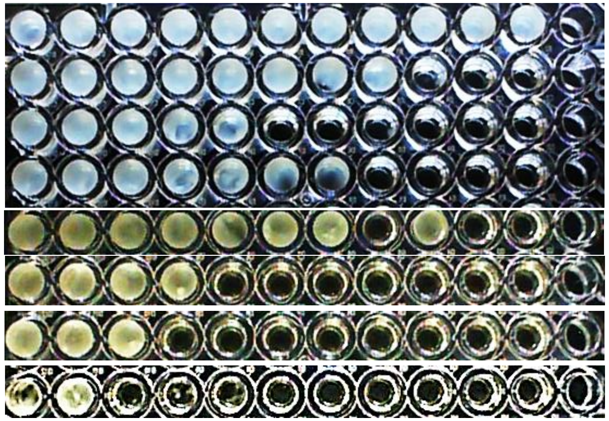

		MBEC (µM)
A)	DMSO	--
B)	1(b)	250
C)	19(a)	6.25
D)	23(a)	12.5
E)	24(a)	100
F)	17(a)	3.13
G)	18(a)	1.56
H)	25(a)	6.25

a 0.20 0.39 0.78 1.56 3.13 6.25 12.5 25 50 100 200 -- µM

b 1 2 3.9 7.8 15.6 31.3 62.5 125 250 500 1000 -- µM

CBD Biofilm Eradication Assay against VRE 700221

VRE Planktonic Killing

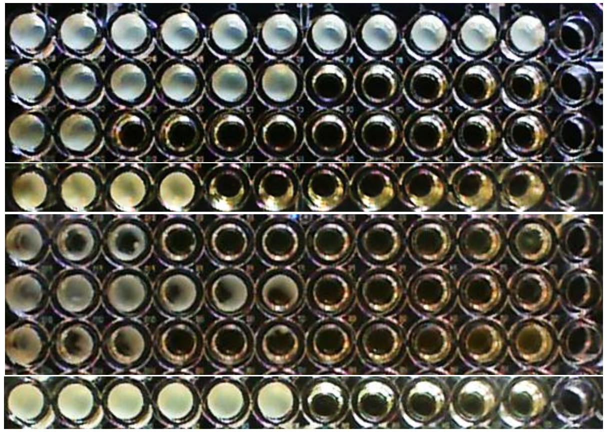

		MBC (µM)
A)	DMSO	--
B)	1	12.5
C)	17	0.78
D)	18	3.13
E)	19	1.56
F)	24	12.5
G)	25	1.56
H)	22	12.5

0.20 0.39 0.78 1.56 3.13 6.25 12.5 25 50 100 200 -- µM

VRE Biofilm Eradication

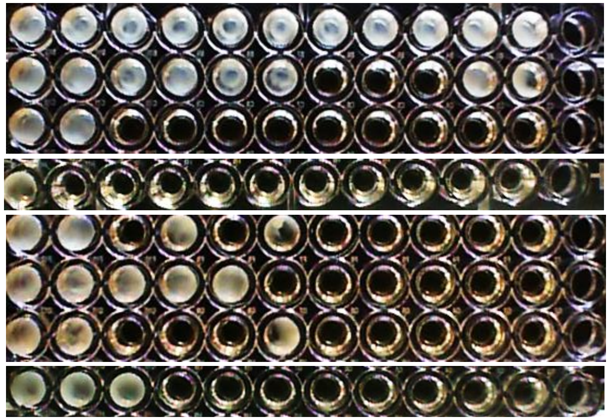

		MBEC (µM)
A)	DMSO	--
B)	1	12.5
C)	17	0.78
D)	18	0.39
E)	19	0.78
F)	24	6.25
G)	25	0.78
H)	22	1.56

0.20 0.39 0.78 1.56 3.13 6.25 12.5 25 50 100 200 -- µM

MIC Assays against MRSA Isolates

MIC Assay Against MRSA BAA-1707

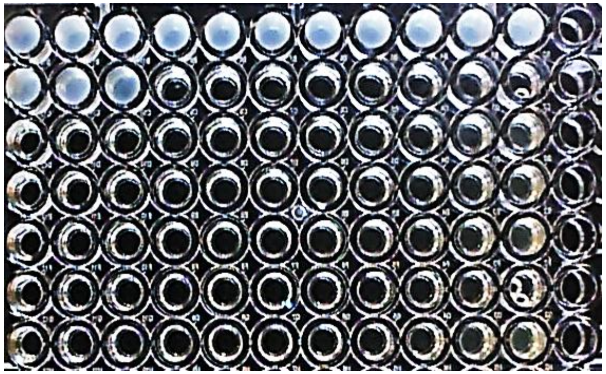

Row	Compound	MIC (µM)
A)	DMSO	--
B)	1	1.56
C)	22	0.10
D)	18	0.10
E)	21	0.10
F)	23	0.10
G)	19	0.10

0.1 0.2 0.39 0.78 1.56 3.13 6.25 12.5 25 50 100 -- µM

MIC Assay Against MRSA BAA-1707

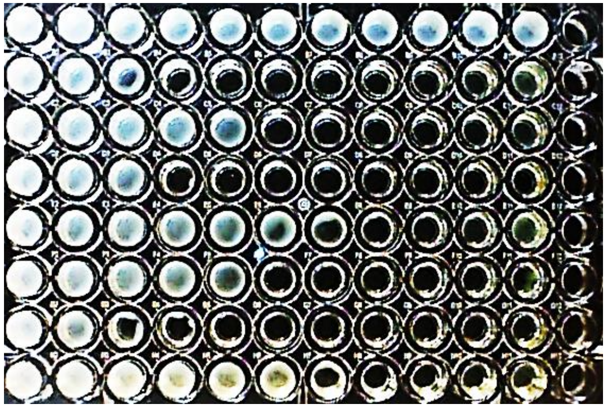

Row	Compound	MIC (µM)
A)	DMSO	--
B)	2	0.78
C)	4	3.13
D)	13	0.78
E)	10	12.5
F)	11	3.13
G)	9	0.78
H)	5	12.5

0.1 0.2 0.39 0.78 1.56 3.13 6.25 12.5 25 50 100 -- µM

MIC Assay Against MRSA BAA-44

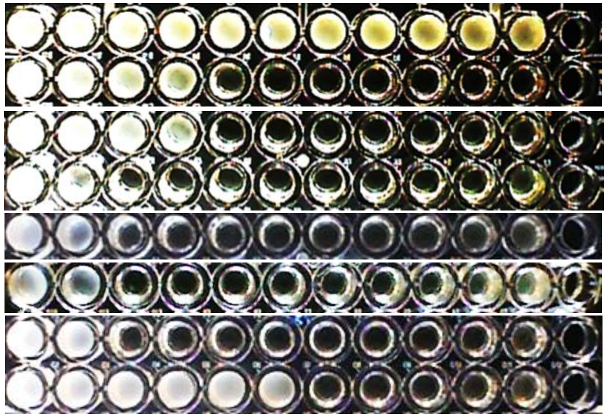

Row	Compound	MIC(µM)
A)	DMSO	--
B)	1	1.56
C)	24	1.56
D)	17	0.39
E)	18	0.78
F)	22	0.39
G)	23	0.39
H)	19	6.25

0.1 0.2 0.39 0.78 1.56 3.13 6.25 12.5 25 50 100 -- µM

MIC Assay Against MRSA-1

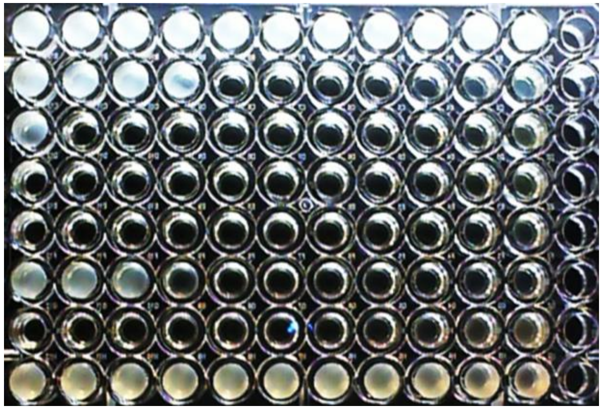

Row	Compound	MIC (µM)
A)	DMSO	--
B)	3 (b)	1.56
C)	18 (b)	0.20
D)	21 (b)	0.10
E)	23 (b)	0.10
F)	24 (b)	1.56
G)	25 (b)	0.10
H)	15 (a)	>50

(a) 0.05 0.1 0.2 0.39 0.78 1.56 3.13 6.25 12.5 25 50 -- µM

(b) 0.1 0.2 0.39 0.78 1.56 3.13 6.25 12.5 25 50 100 -- µM

MIC Assays Against MRSE and VRE

MIC Assay Against MRSA-2

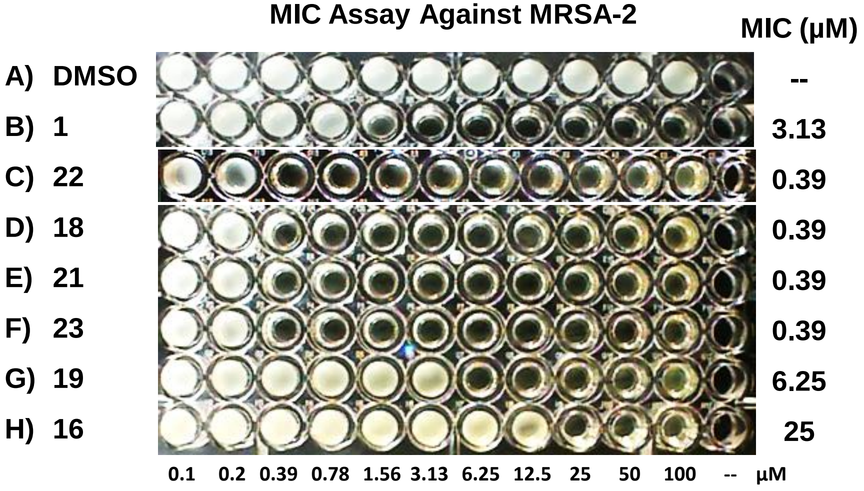

MIC Assay Against MRSE 35984

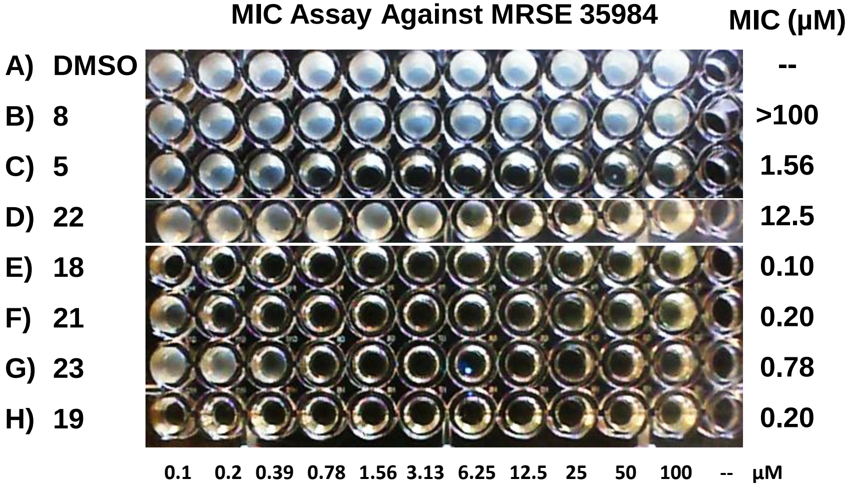

MIC Assay Against *S. epidermidis* 12228

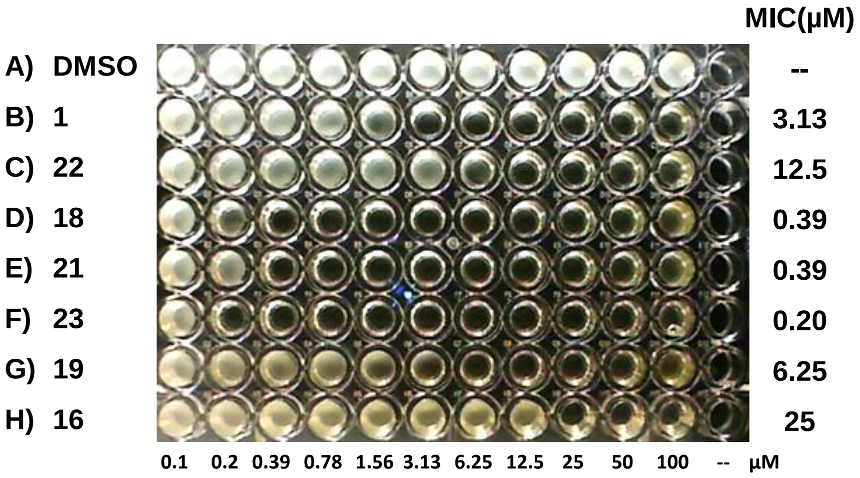

MIC Assay Against VRE 700221

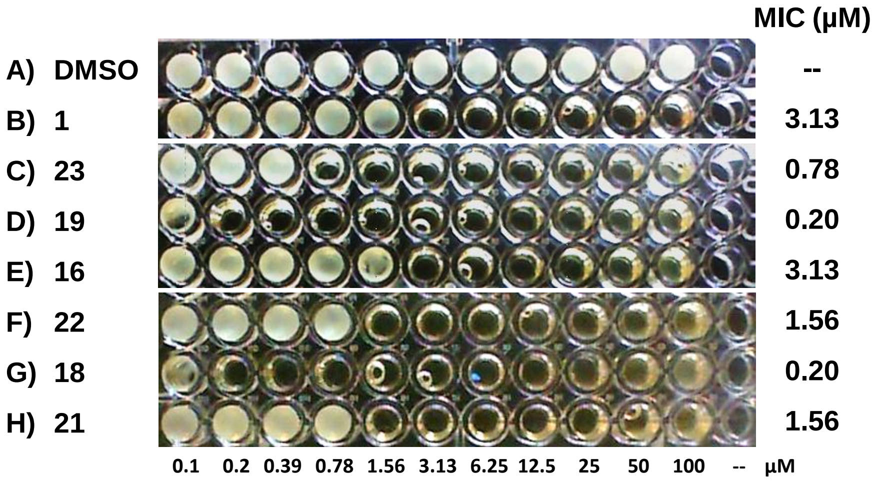

Co-Treatment of MRSA BAA-1707 with Metal(II) Cations

Co-Treatment of MRSA BAA 1707 with 200 µM Cu(II)

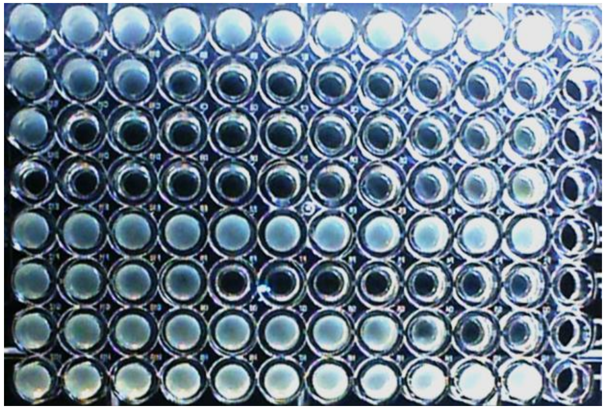

0.1 0.2 0.39 0.78 1.56 3.13 6.25 12.5 25 50 100 -- µM

Row	Treatment	MIC (µM)
A)	DMSO	--
B)	1	0.78
C)	17	**0.20**
D)	25	0.10
E)	DMSO + Cu(II)	--
F)	1 + Cu(II)	3.13
G)	17 + Cu(II)	**50**
H)	25 + Cu(II)	>100

The antibacterial activity of HP 17 is reduced 250-fold in the presence of copper (II)

Co-Treatment of MRSA BAA 1707 with 200 µM Fe(II)

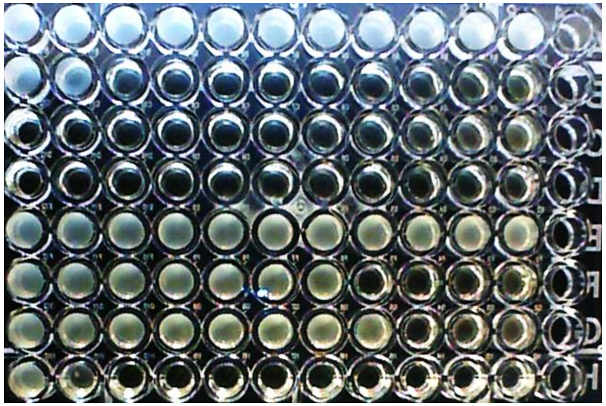

Row	Treatment	MIC (µM)
A)	DMSO	--
B)	17	0.39
C)	25	0.10
D)	21	0.10
E)	DMSO + Fe(II)	--
F)	17 + Fe(II)	12.5
G)	25 + Fe(II)	25
H)	21 + Fe(II)	0.78

Co-Treatment of MRSA BAA 1707 with Metal(II) Cations

Co-Treatment of MRSA BAA 1707 with 200 μM Zn(II)
Extended Plate Assay

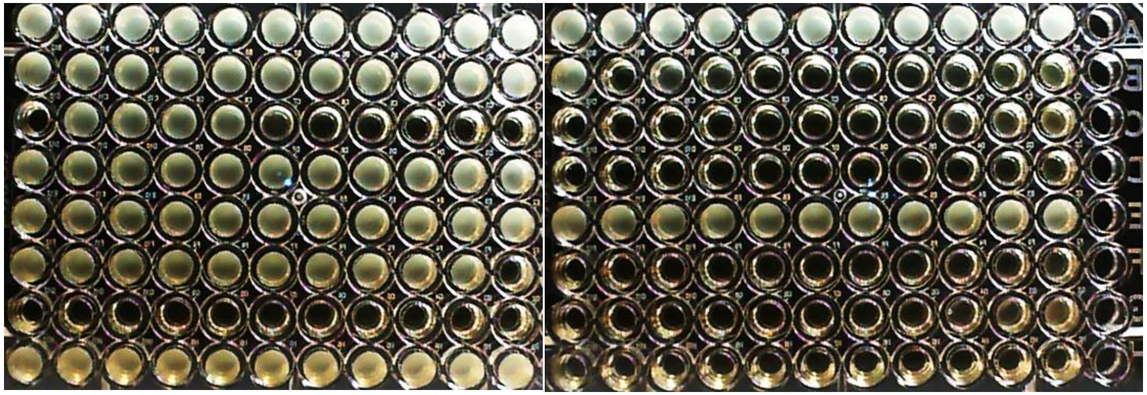

	MIC (μM)
A) DMSO	--
B) 17	0.20
C) 25	0.003
D) 21	0.10
E) DMSO + Zn(II)	--
F) 17 + Zn(II)	0.05
G) 25 + Zn(II)	0.00005
H) 21 + Zn(II)	0.10

Columns: 0.00005, 0.0001, 0.0002, 0.00039, 0.00078, 0.00156, 0.00313, 0.00625, 0.0125, 0.025, 0.05, 0.1, 0.2, 0.39, 0.78, 1.56, 3.13, 6.25, 12.5, 25, 50, 100 μM

Co-Treatment of MRSA BAA 1707 with 200 μM Mg(II)

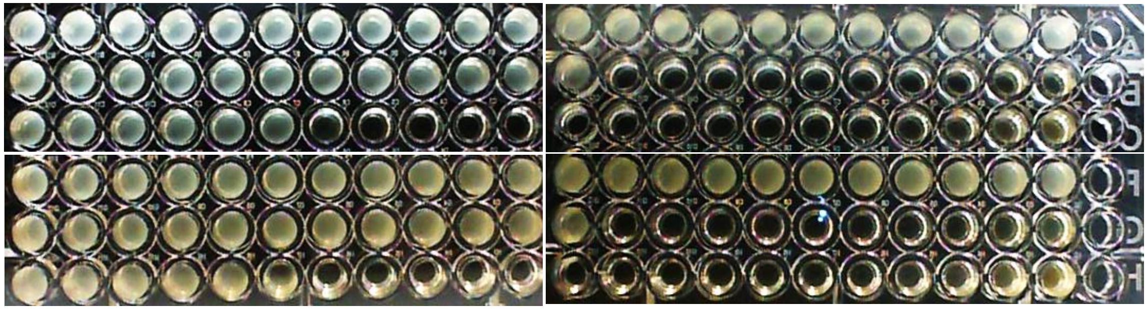

	MIC (μM)
A) DMSO	--
B) 17	0.20
C) 25	0.003
D) DMSO + Mg(II)	--
E) 17 + Mg(II)	0.20
F) 25 + Mg (II)	0.003

Columns: 0.00005, 0.0001, 0.0002, 0.00039, 0.00078, 0.00156, 0.00313, 0.00625, 0.0125, 0.025, 0.05, 0.1, 0.2, 0.39, 0.78, 1.56, 3.13, 6.25, 12.5, 25, 50, 100 μM

Note: Co-treatment MIC assays performed over two 96-well plates for highly potent compounds.

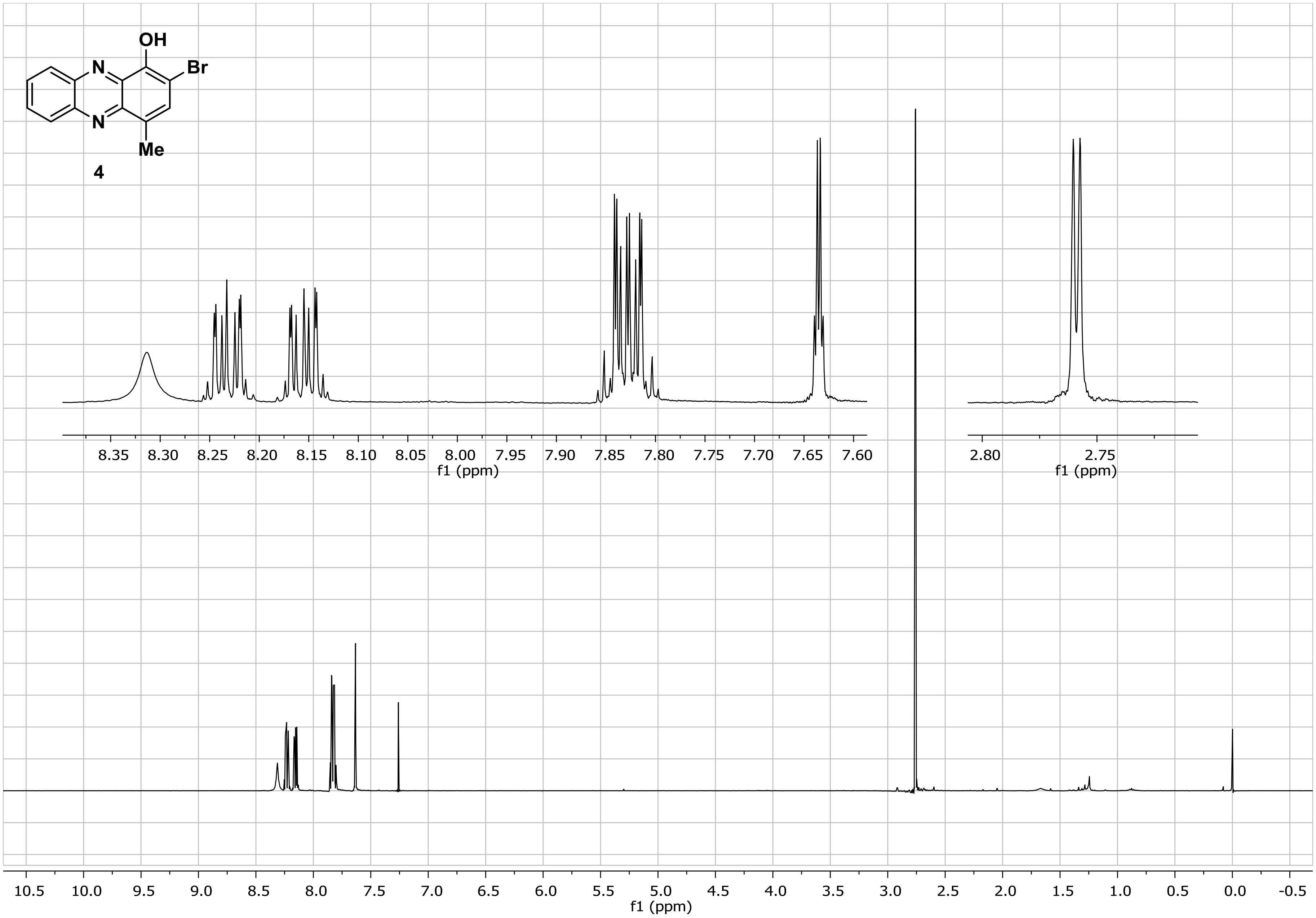

OH
Br
N
N
Me
4
8.35
8.30
8.25
8.20
8.15
8.10
8.05
8.00
7.95
7.90
7.85
7.80
7.75
7.70
7.65
7.60
f1 (ppm)
2.80
2.75
f1 (ppm)
10.5
10.0
9.5
9.0
8.5
8.0
7.5
7.0
6.5
6.0
5.5
5.0
4.5
4.0
3.5
3.0
2.5
2.0
1.5
1.0
0.5
0.0
-0.5
f1 (ppm)

NMR-1

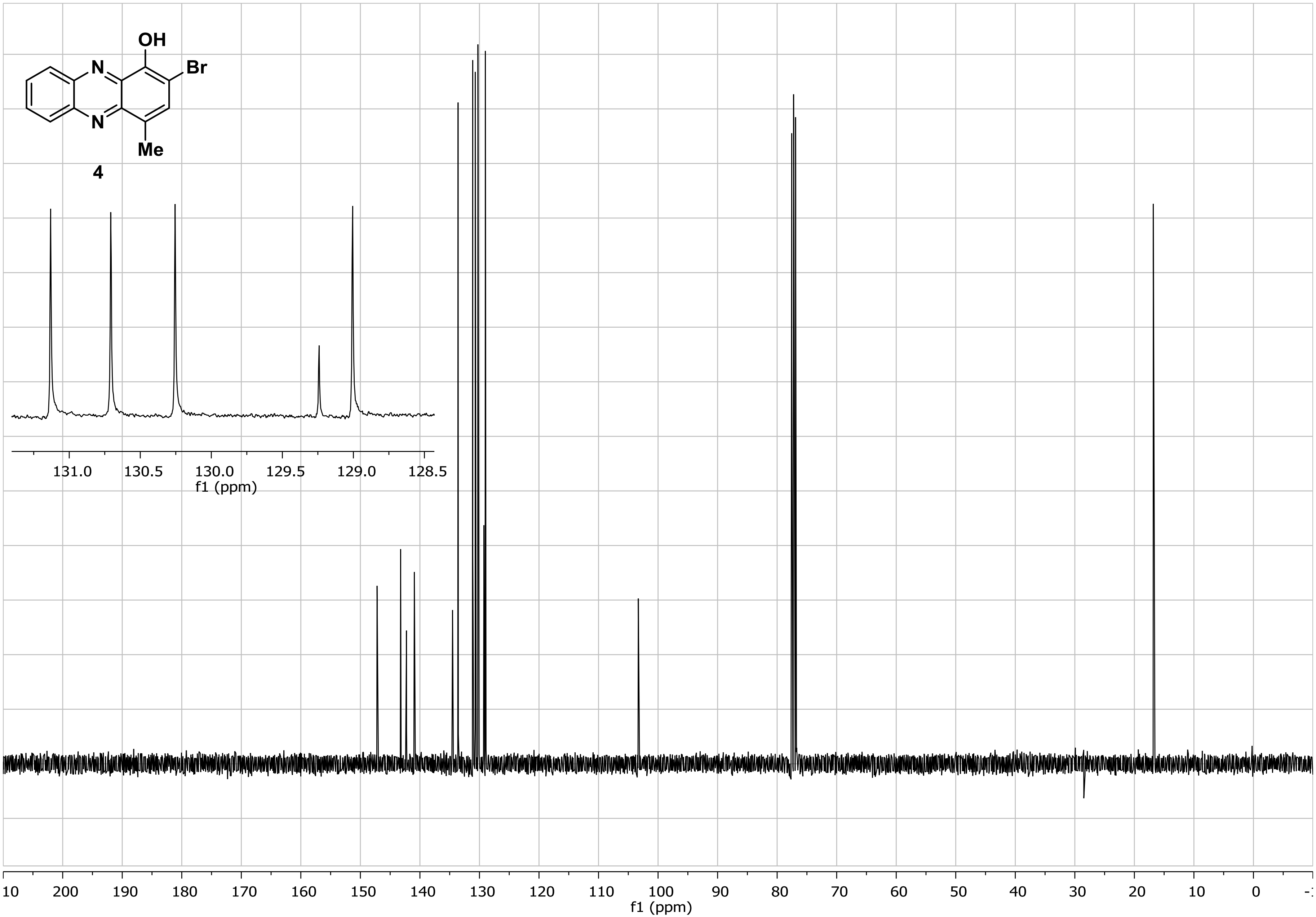
OH
Br
N
N
Me
4
131.0
130.5
130.0
129.5
129.0
128.5
f1 (ppm)
200
190
180
170
160
150
140
130
120
110
100
90
80
70
60
50
40
30
20
10
0
f1 (ppm)

NMR-2

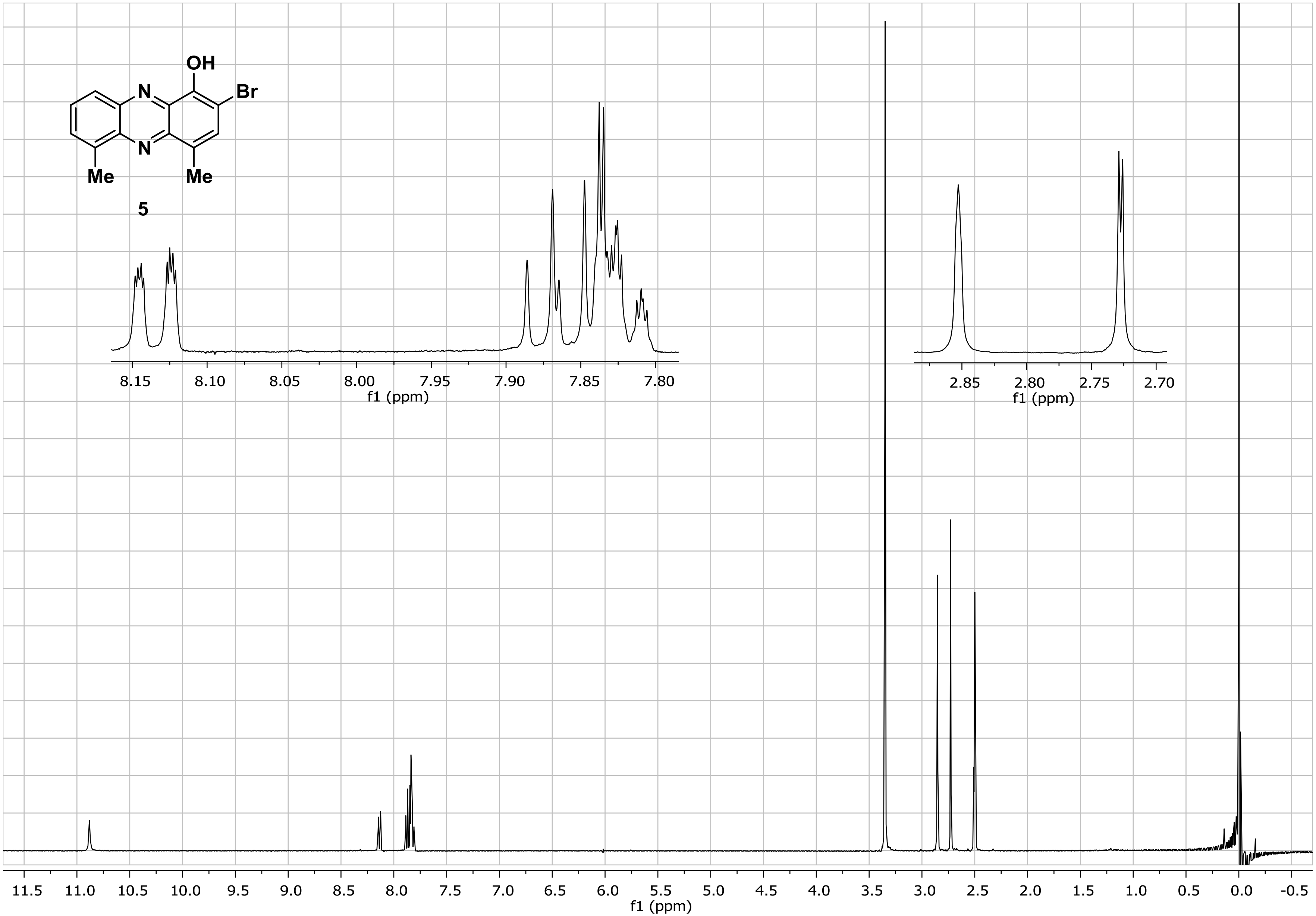

NMR-3

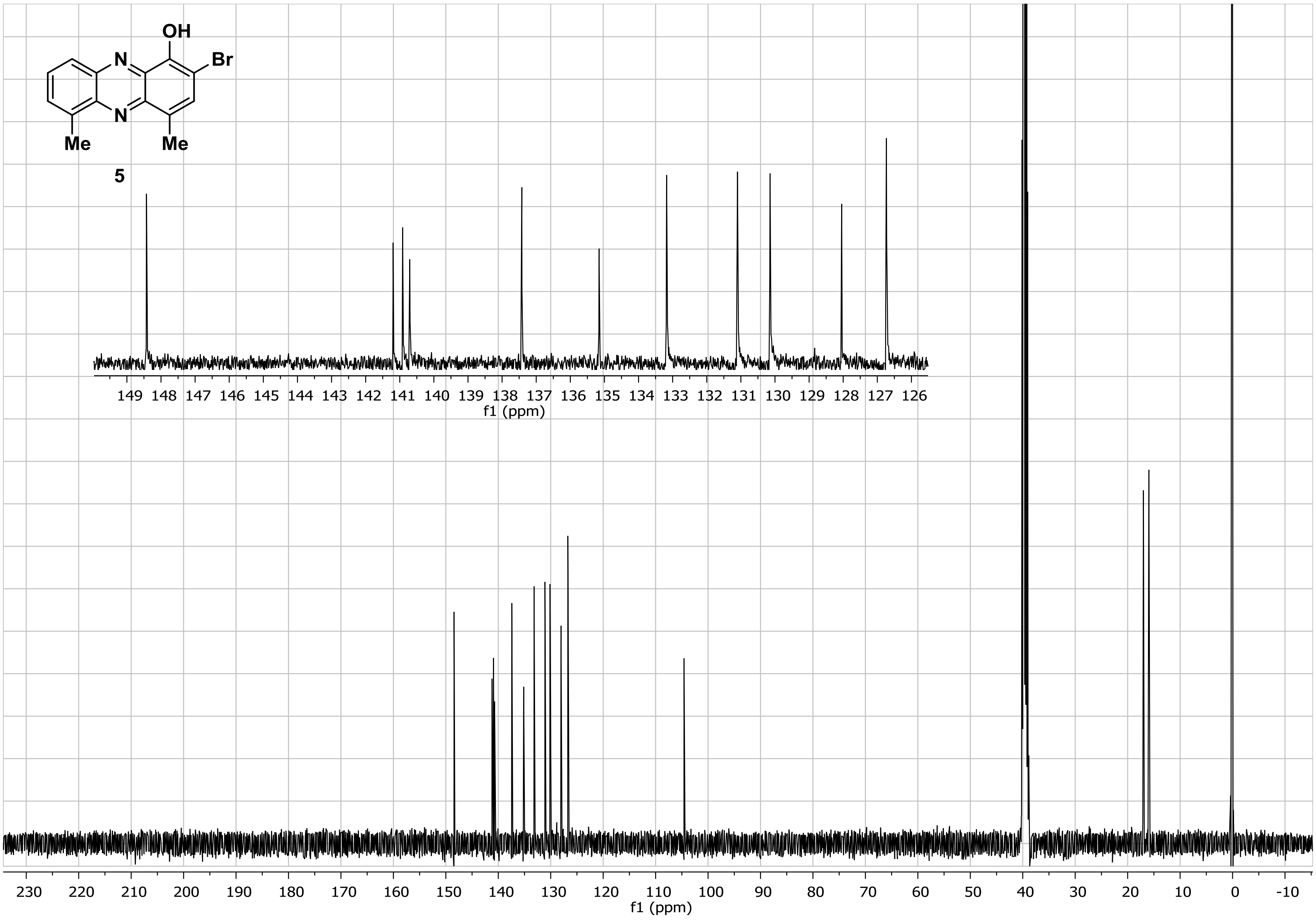
OH
Br
N
N
Me
Me
5
149 148 147 146 145 144 143 142 141 140 139 138 137 136 135 134 133 132 131 130 129 128 127 126
f1 (ppm)
230 220 210 200 190 180 170 160 150 140 130 120 110 100 90 80 70 60 50 40 30 20 10 0 -10
f1 (ppm)

NMR-4

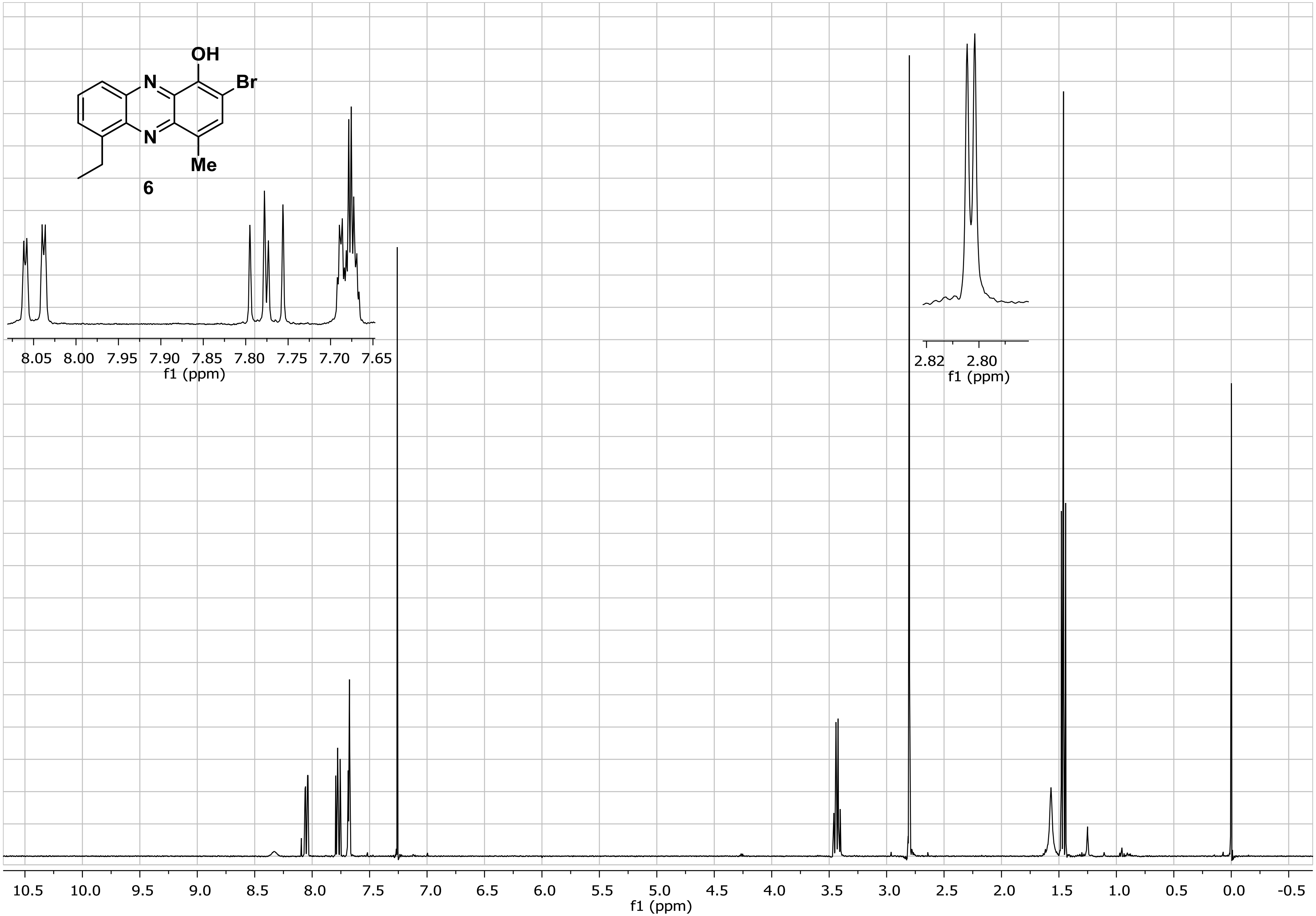
OH
Br
N
N
Me
6
8.05 8.00 7.95 7.90 7.85 7.80 7.75 7.70 7.65
f1 (ppm)
2.82 2.80
f1 (ppm)
10.5 10.0 9.5 9.0 8.5 8.0 7.5 7.0 6.5 6.0 5.5 5.0 4.5 4.0 3.5 3.0 2.5 2.0 1.5 1.0 0.5 0.0 -0.5
f1 (ppm)

NMR-5

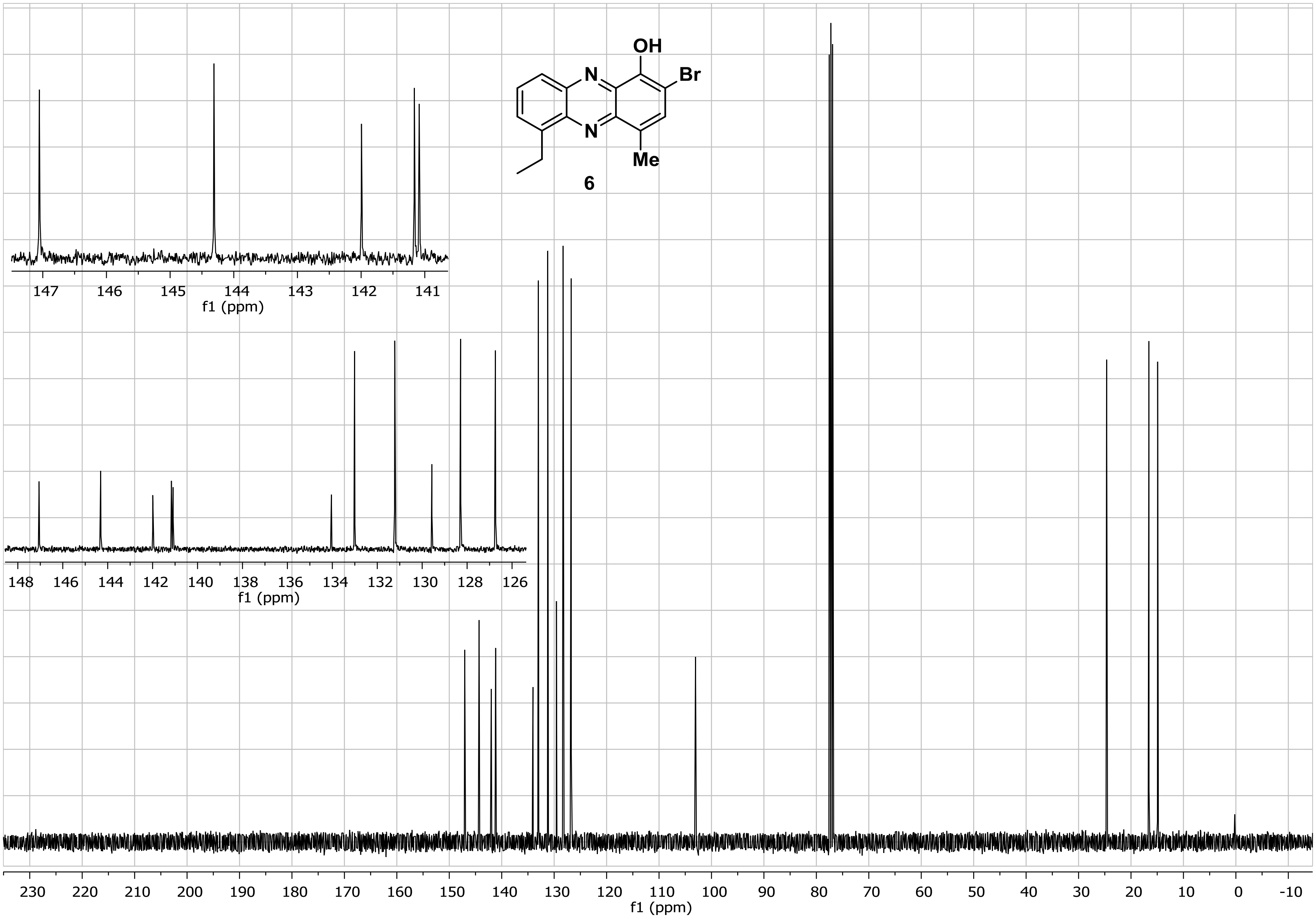
OH
Br
N
N
Me
6
147
146
145
144
143
142
141
f1 (ppm)
148
146
144
142
140
138
136
134
132
130
128
126
f1 (ppm)
230
220
210
200
190
180
170
160
150
140
130
120
110
100
90
80
70
60
50
40
30
20
10
0
-10
f1 (ppm)

NMR-6

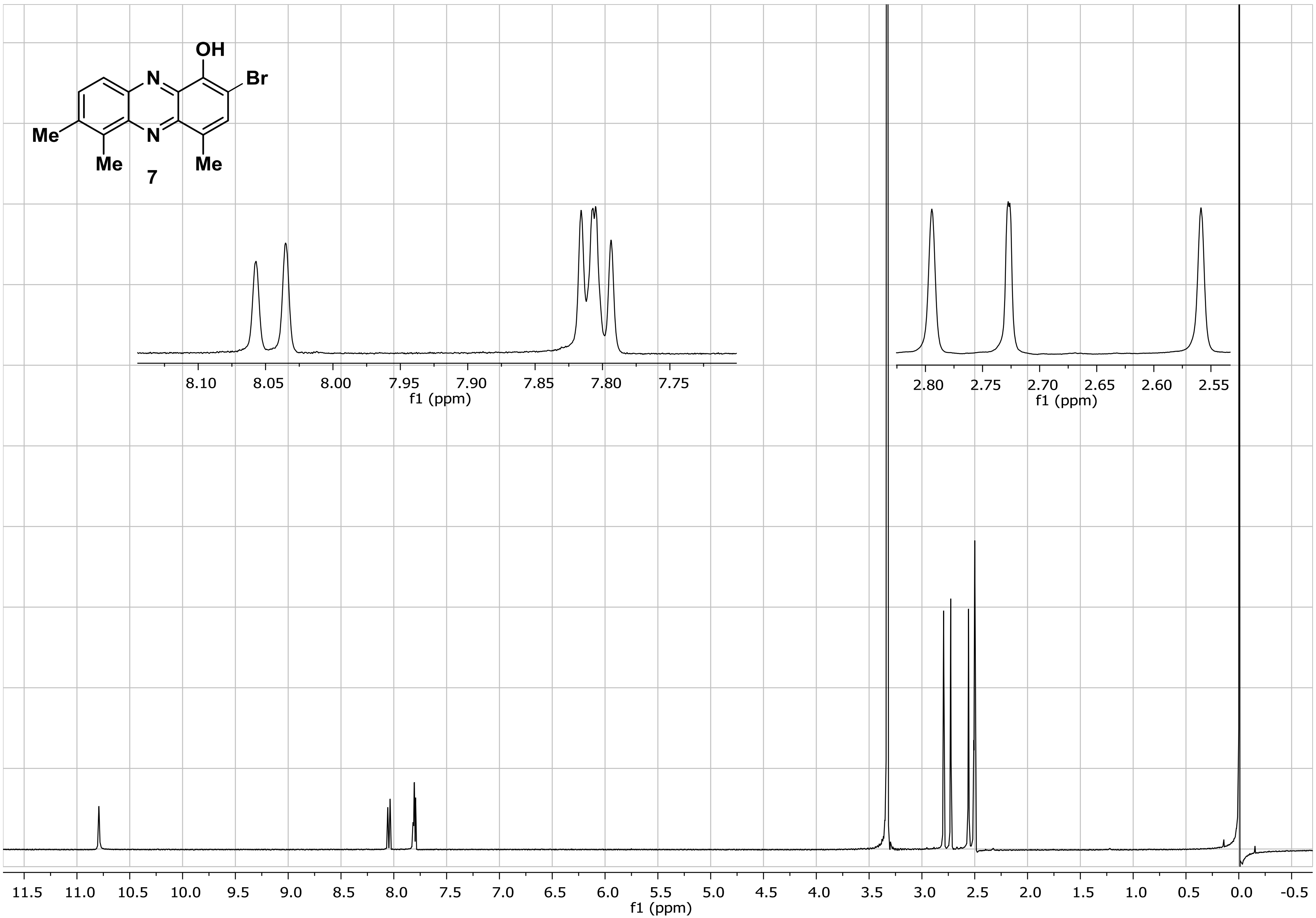

NMR-7

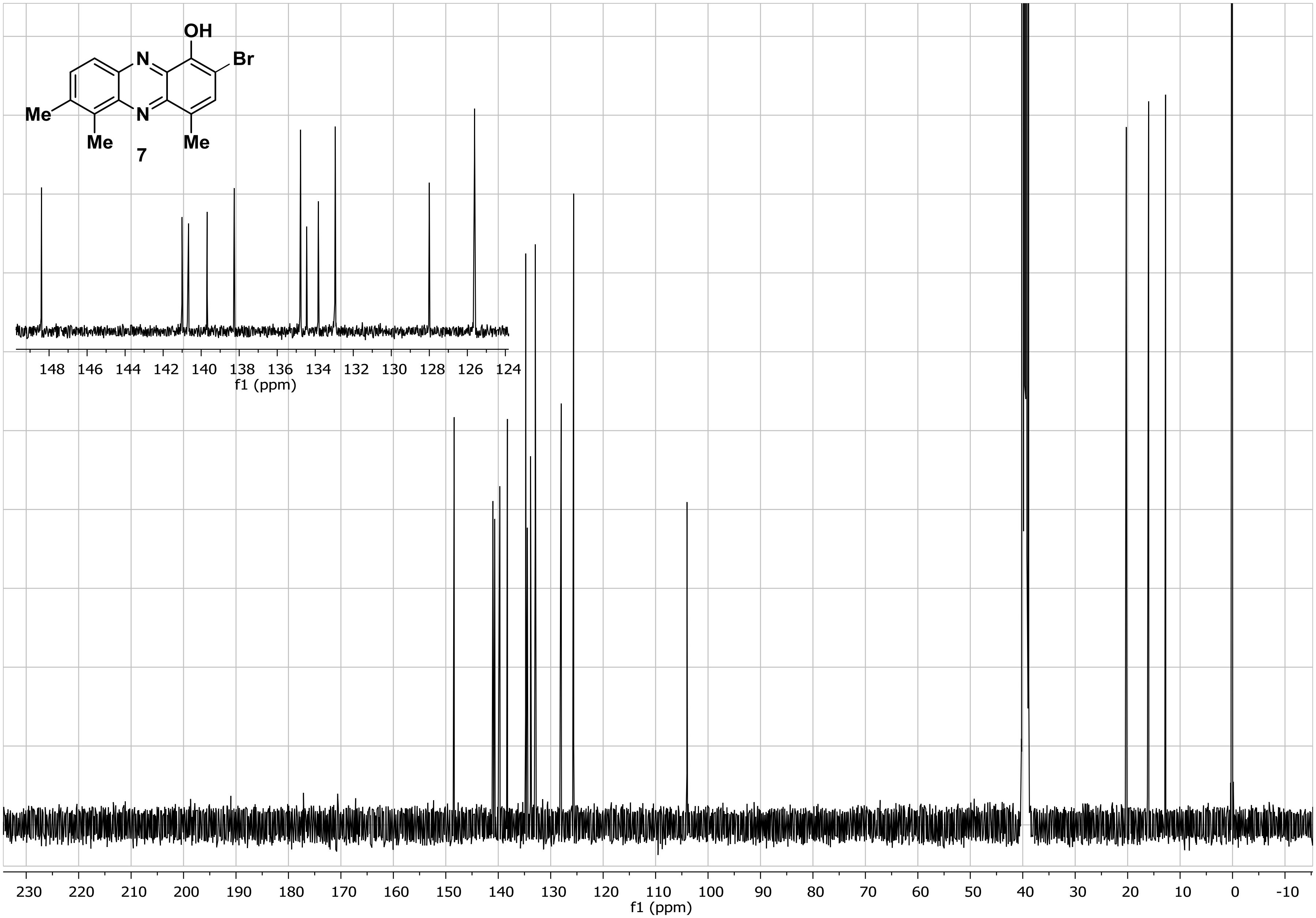

NMR-8

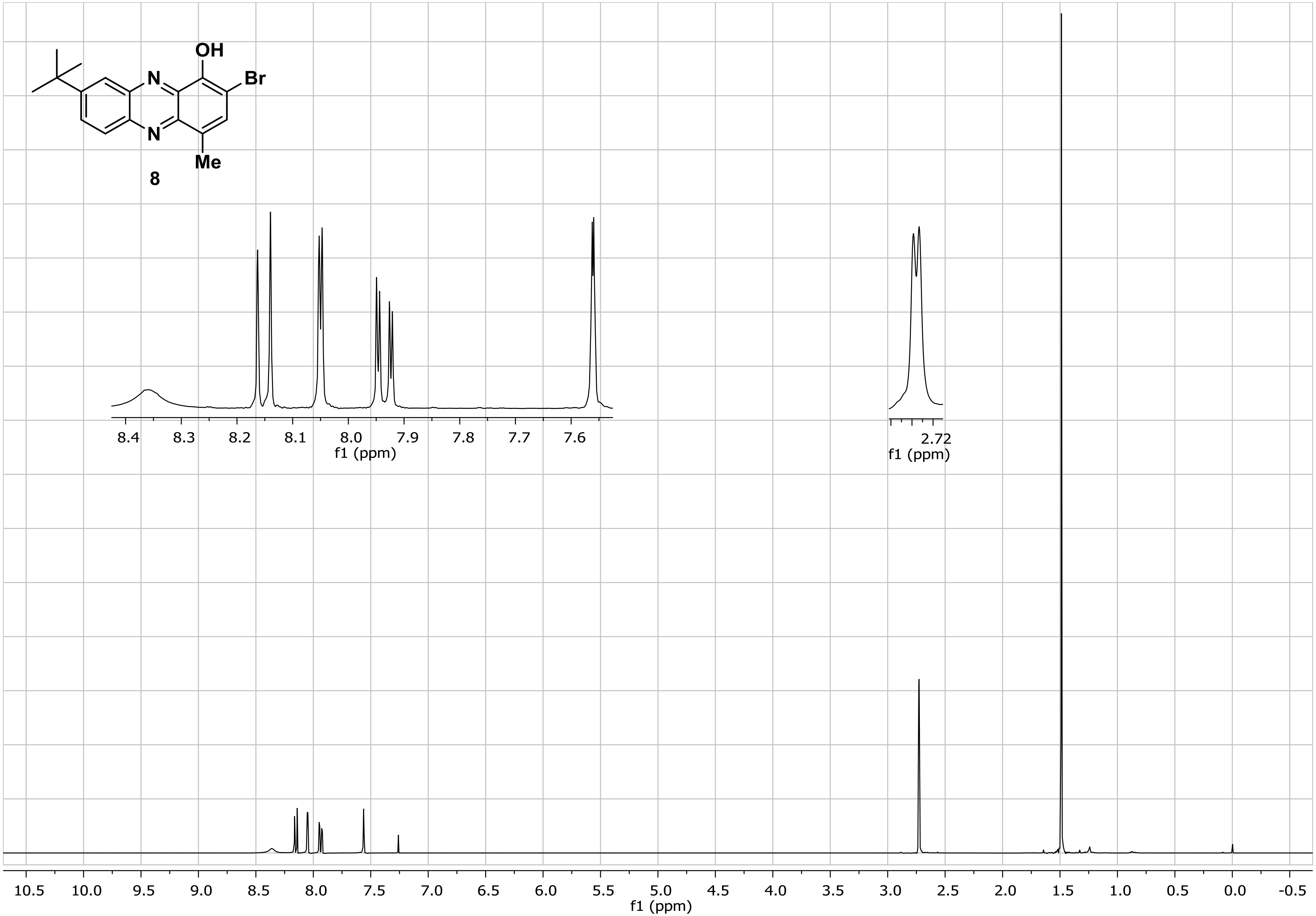
OH
Br
N
N
Me
8
8.4
8.3
8.2
8.1
8.0
7.9
7.8
7.7
7.6
f1 (ppm)
2.72
f1 (ppm)
10.5
10.0
9.5
9.0
8.5
8.0
7.5
7.0
6.5
6.0
5.5
5.0
4.5
4.0
3.5
3.0
2.5
2.0
1.5
1.0
0.5
0.0
-0.5
f1 (ppm)

NMR-9

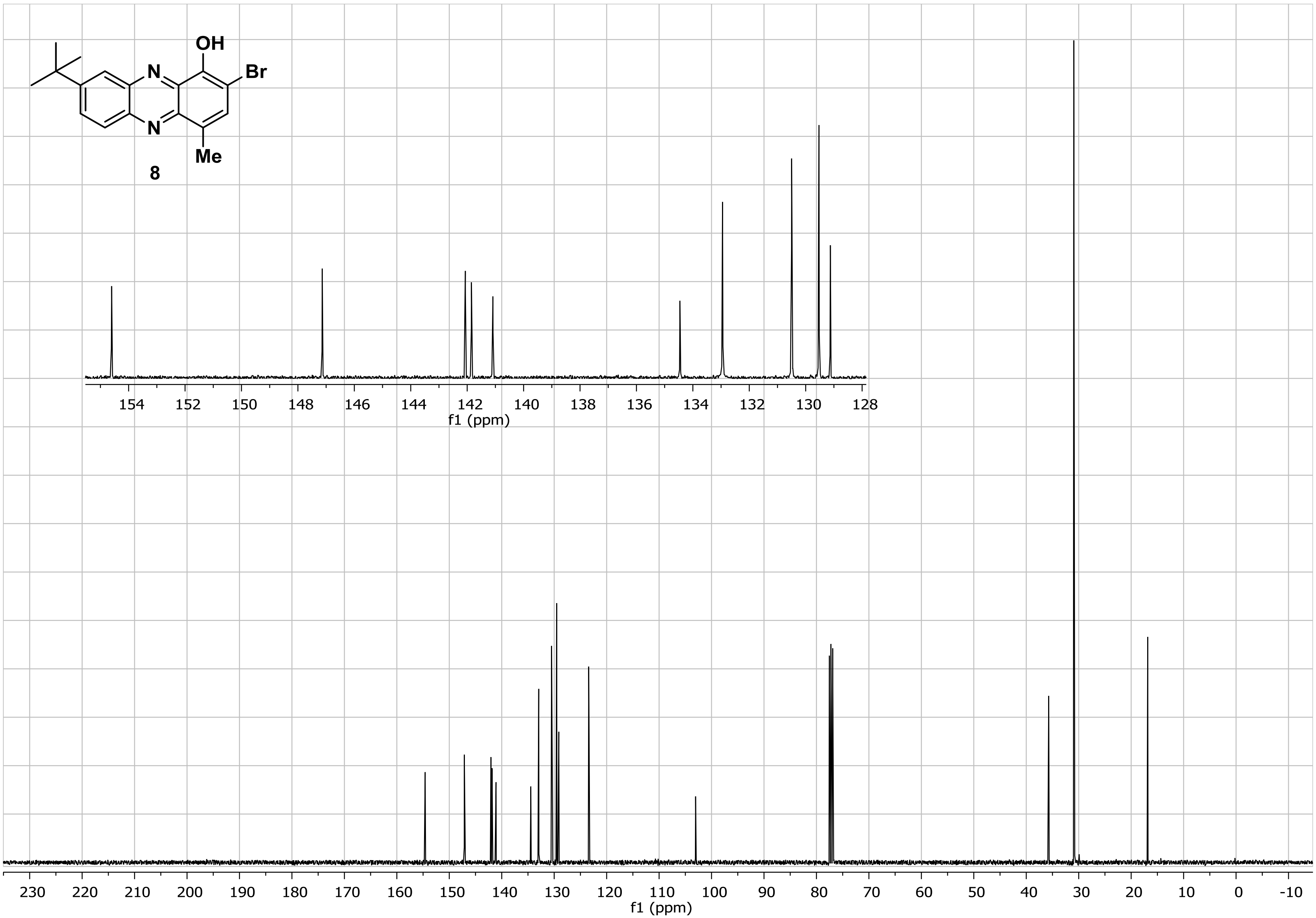
OH
Br
N
N
Me
8
154 152 150 148 146 144 142 140 138 136 134 132 130 128
f1 (ppm)
230 220 210 200 190 180 170 160 150 140 130 120 110 100 90 80 70 60 50 40 30 20 10 0 -10
f1 (ppm)

NMR-10

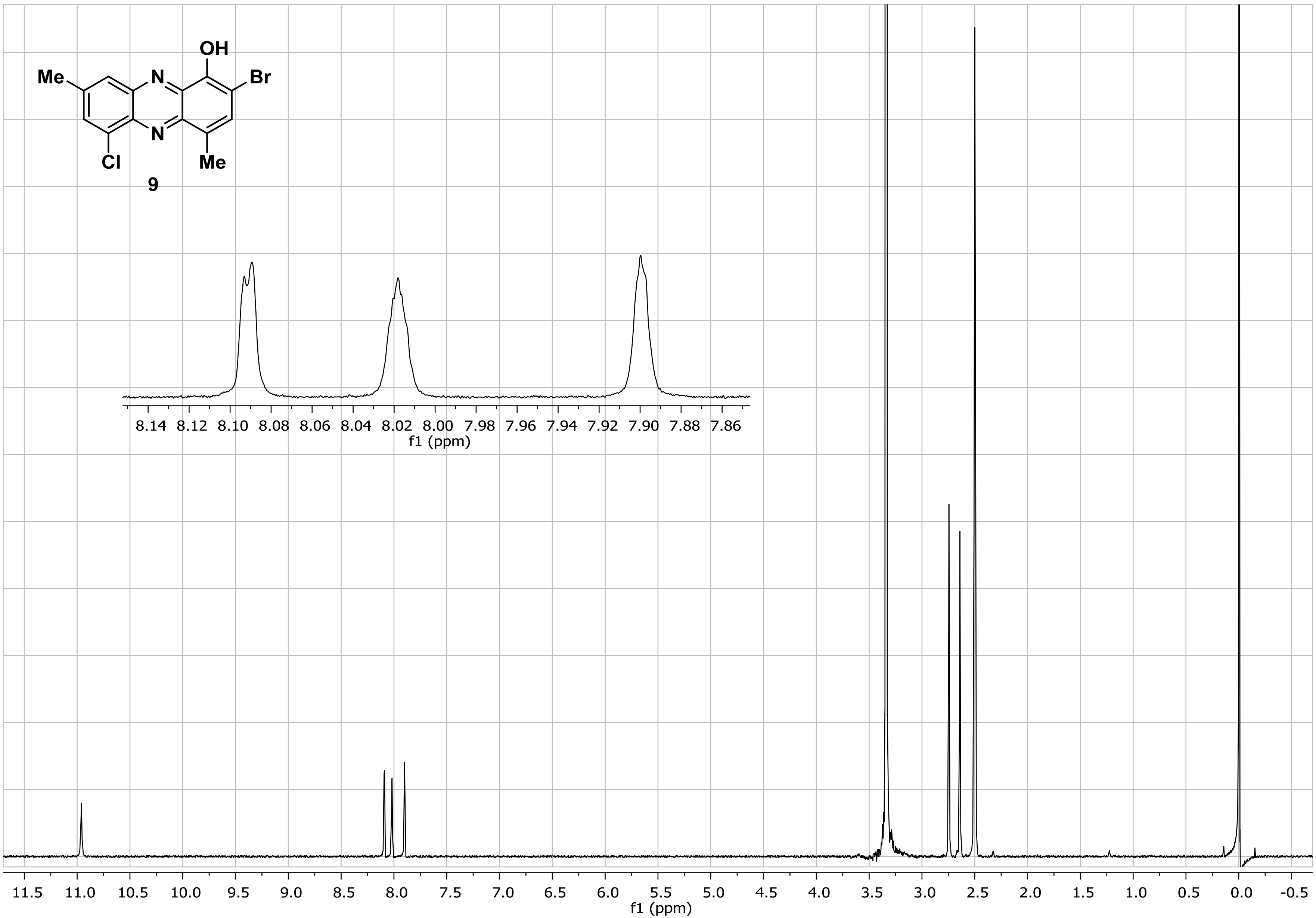

OH
Me
Br
N
N
Cl
Me
9
8.14 8.12 8.10 8.08 8.06 8.04 8.02 8.00 7.98 7.96 7.94 7.92 7.90 7.88 7.86
f1 (ppm)
11.5 11.0 10.5 10.0 9.5 9.0 8.5 8.0 7.5 7.0 6.5 6.0 5.5 5.0 4.5 4.0 3.5 3.0 2.5 2.0 1.5 1.0 0.5 0.0 -0.5
f1 (ppm)

NMR-11

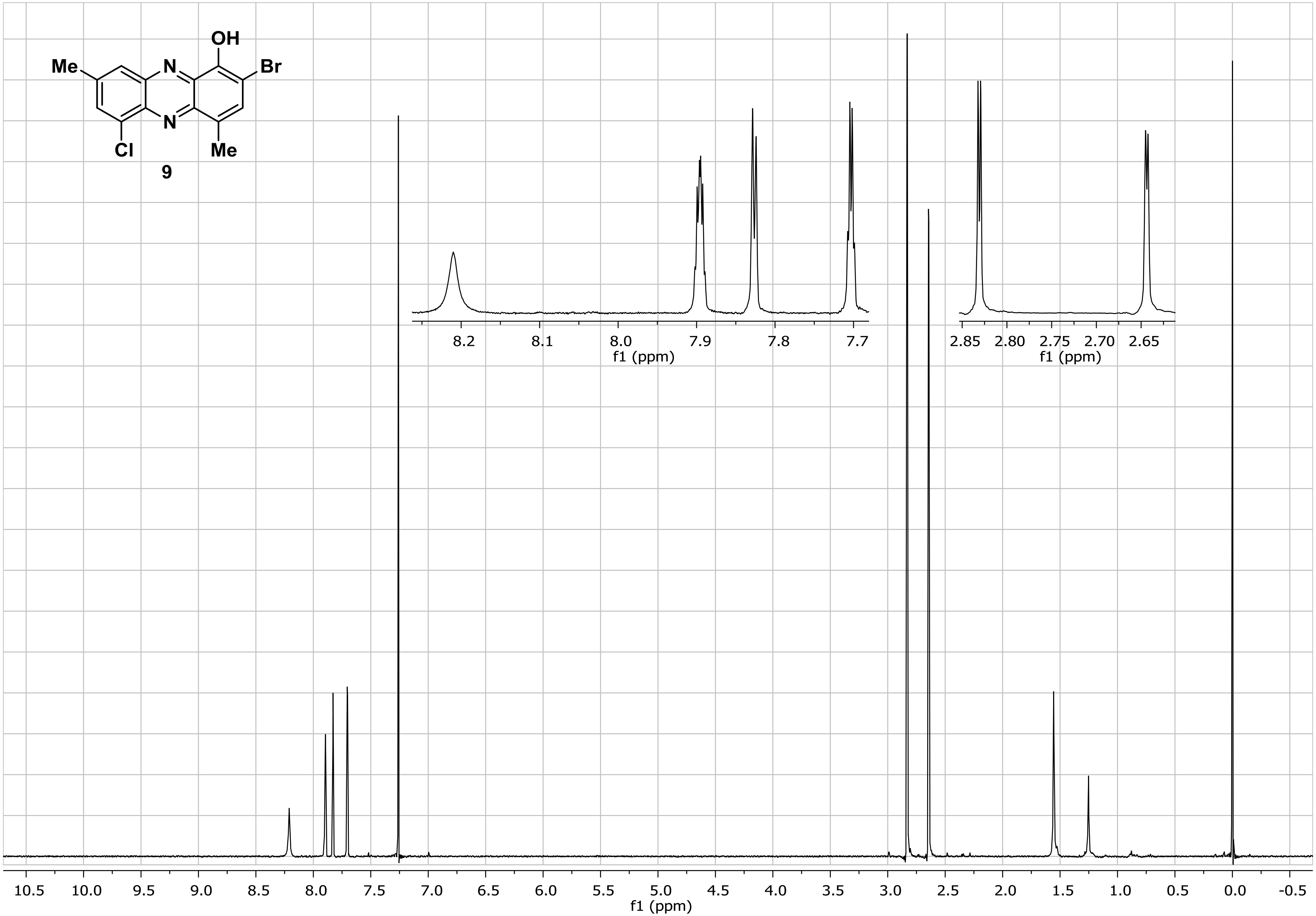
OH
Me
N
Br
N
Cl
Me
9
8.2
8.1
8.0
7.9
7.8
7.7
f1 (ppm)
2.85
2.80
2.75
2.70
2.65
f1 (ppm)
10.5
10.0
9.5
9.0
8.5
8.0
7.5
7.0
6.5
6.0
5.5
5.0
4.5
4.0
3.5
3.0
2.5
2.0
1.5
1.0
0.5
0.0
-0.5
f1 (ppm)

NMR-12

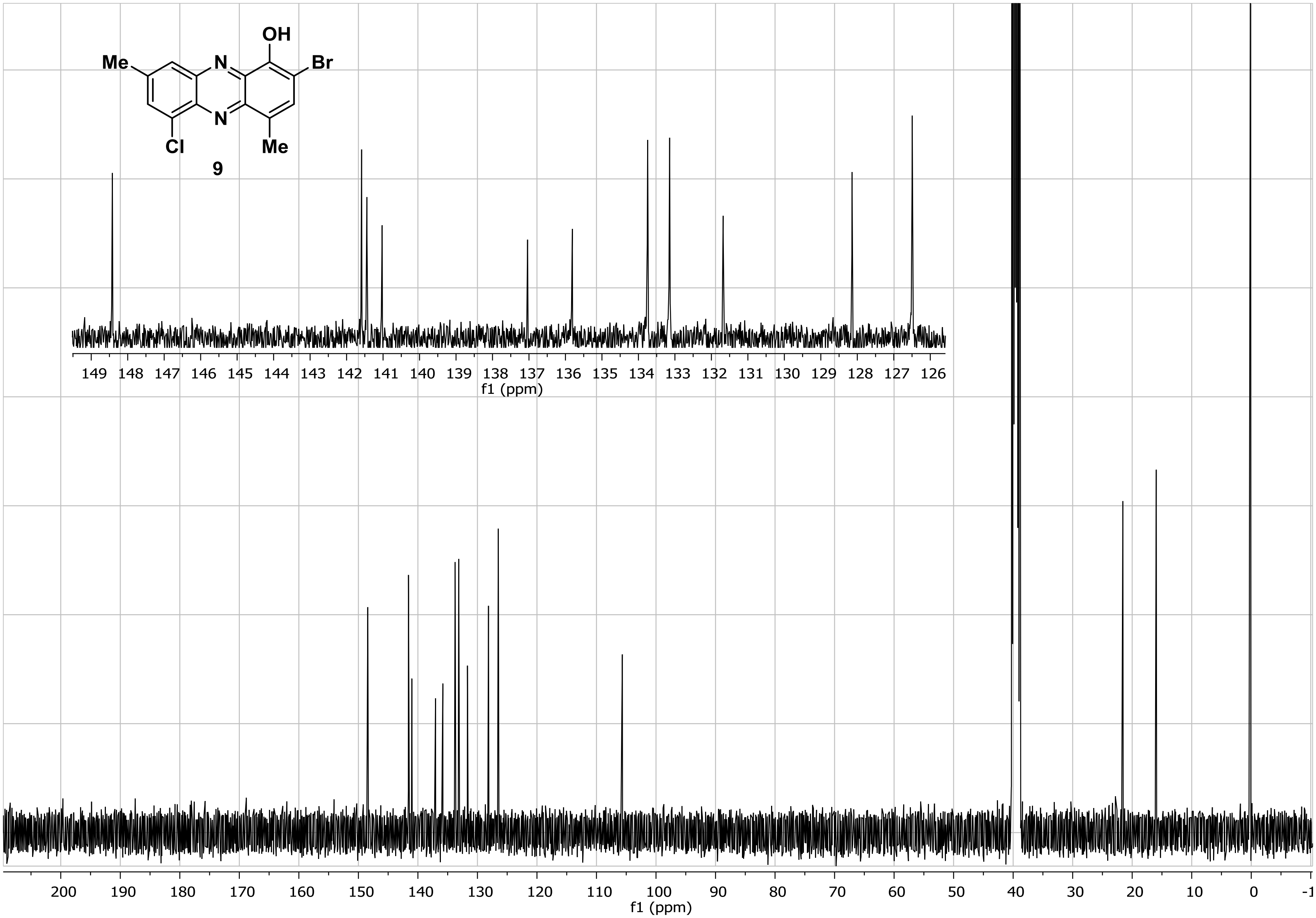
OH
Br
Me
N
N
Cl
Me
9
149 148 147 146 145 144 143 142 141 140 139 138 137 136 135 134 133 132 131 130 129 128 127 126
f1 (ppm)
200 190 180 170 160 150 140 130 120 110 100 90 80 70 60 50 40 30 20 10 0 -1
f1 (ppm)

NMR-13

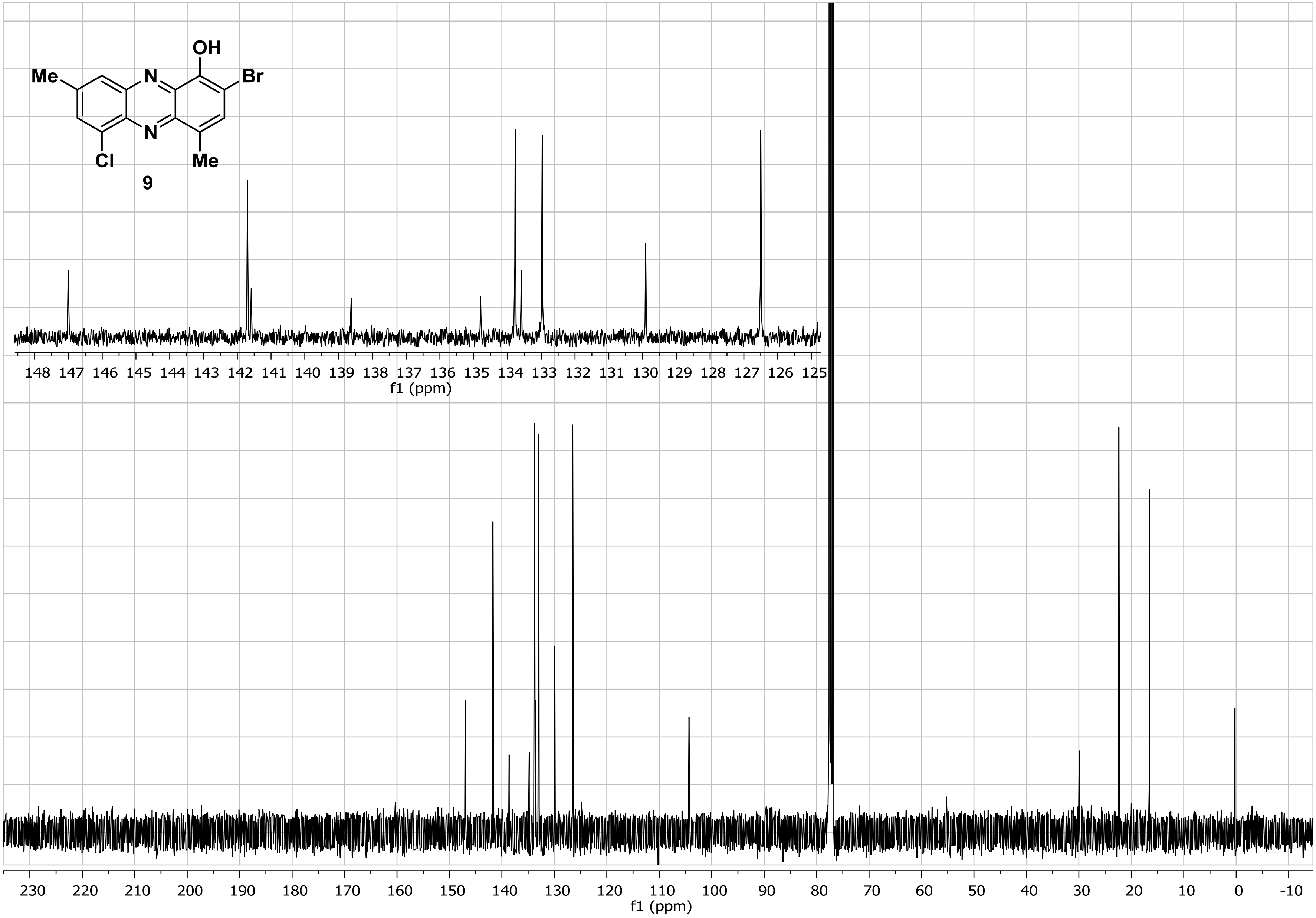
OH
Br
Me
N
N
Cl
Me
9
148 147 146 145 144 143 142 141 140 139 138 137 136 135 134 133 132 131 130 129 128 127 126 125
f1 (ppm)
230 220 210 200 190 180 170 160 150 140 130 120 110 100 90 80 70 60 50 40 30 20 10 0 -10
f1 (ppm)

NMR-14

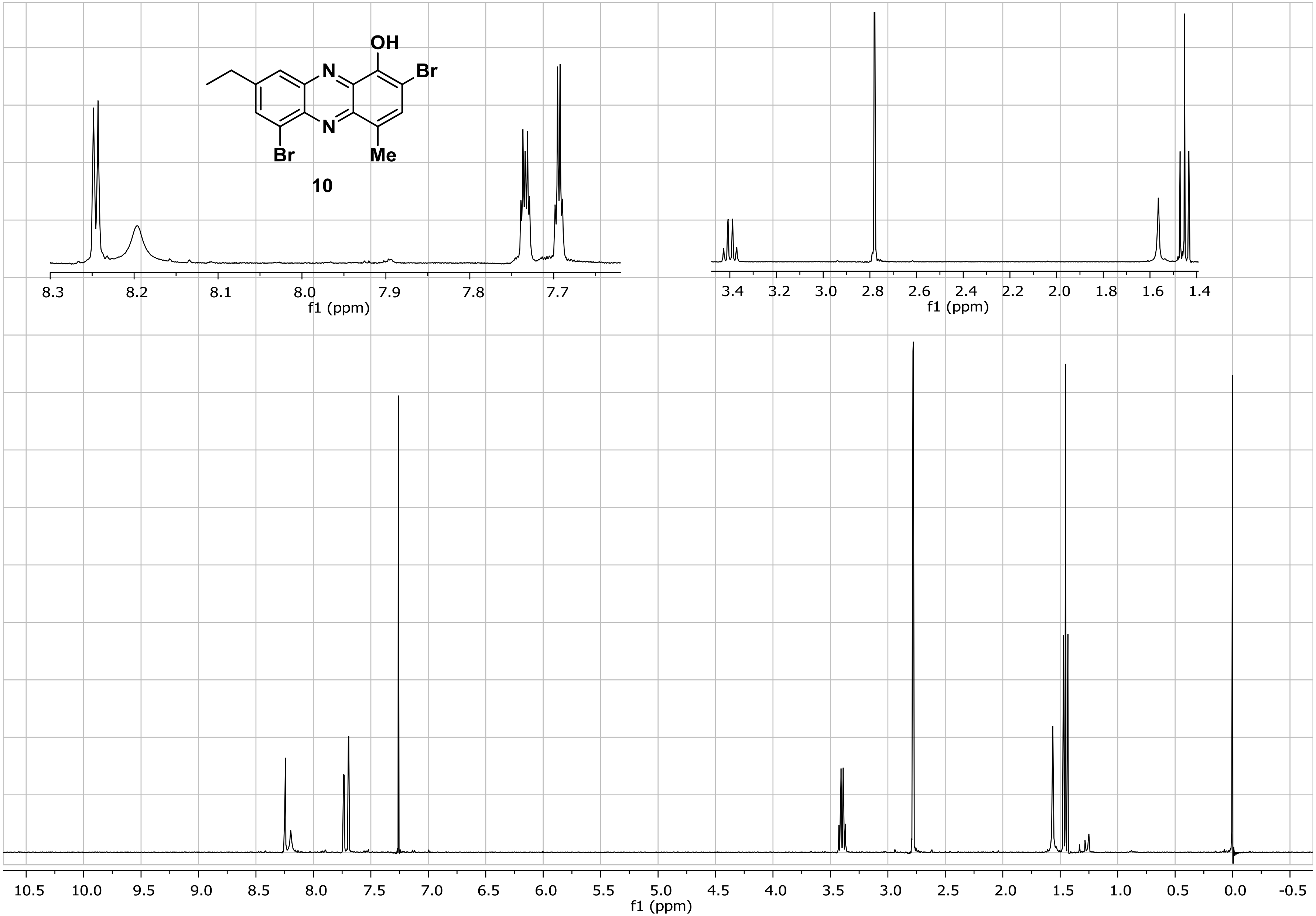
OH
Br
N
N
Br
Me
10
8.3
8.2
8.1
8.0
7.9
7.8
7.7
f1 (ppm)
3.4
3.2
3.0
2.8
2.6
2.4
2.2
2.0
1.8
1.6
1.4
f1 (ppm)
10.5
10.0
9.5
9.0
8.5
8.0
7.5
7.0
6.5
6.0
5.5
5.0
4.5
4.0
3.5
3.0
2.5
2.0
1.5
1.0
0.5
0.0
-0.5
f1 (ppm)

NMR-15

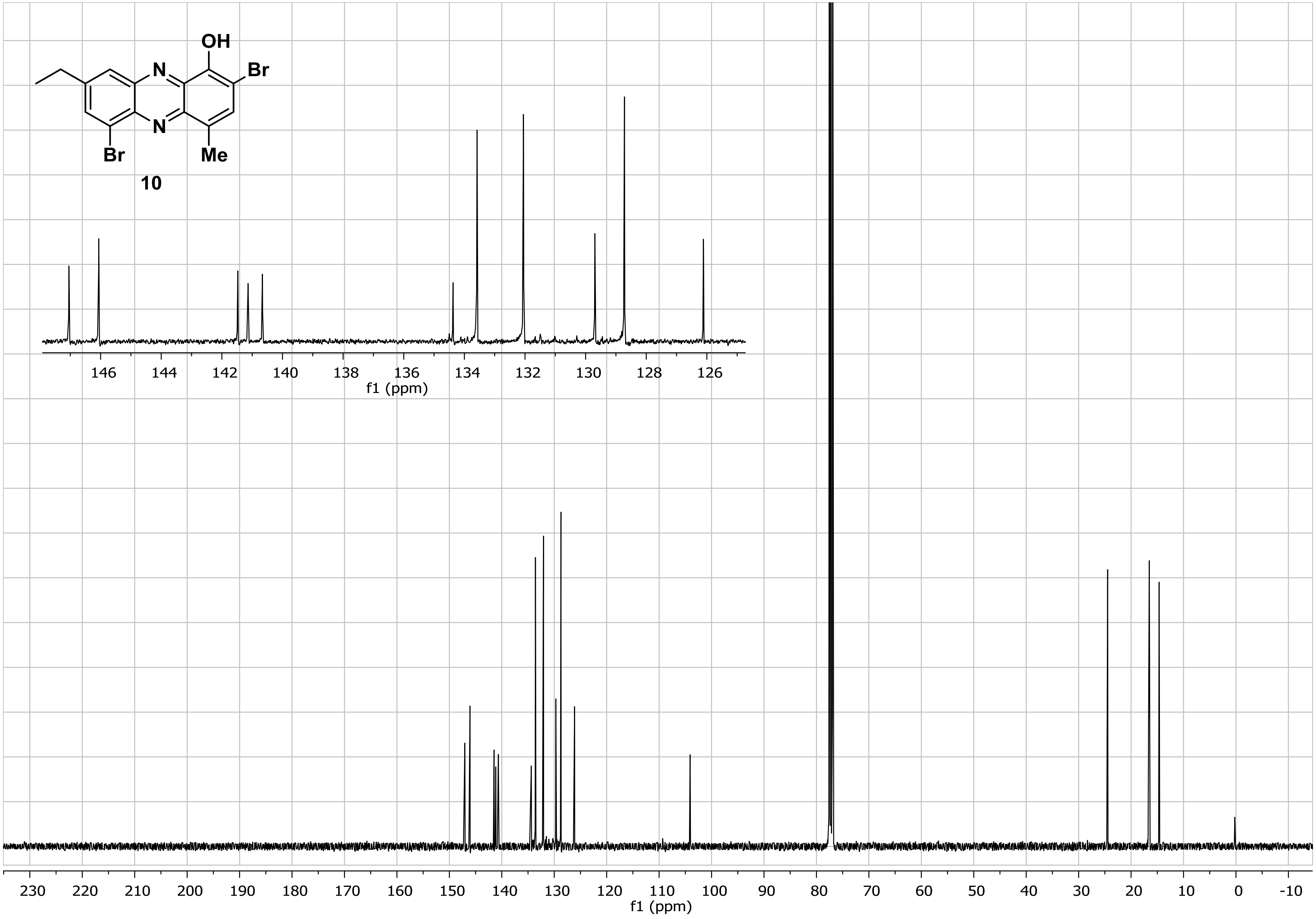
OH
Br
N
N
Br
Me
10
146
144
142
140
138
136
134
132
130
128
126
f1 (ppm)
230
220
210
200
190
180
170
160
150
140
130
120
110
100
90
80
70
60
50
40
30
20
10
0
-10
f1 (ppm)

NMR-16

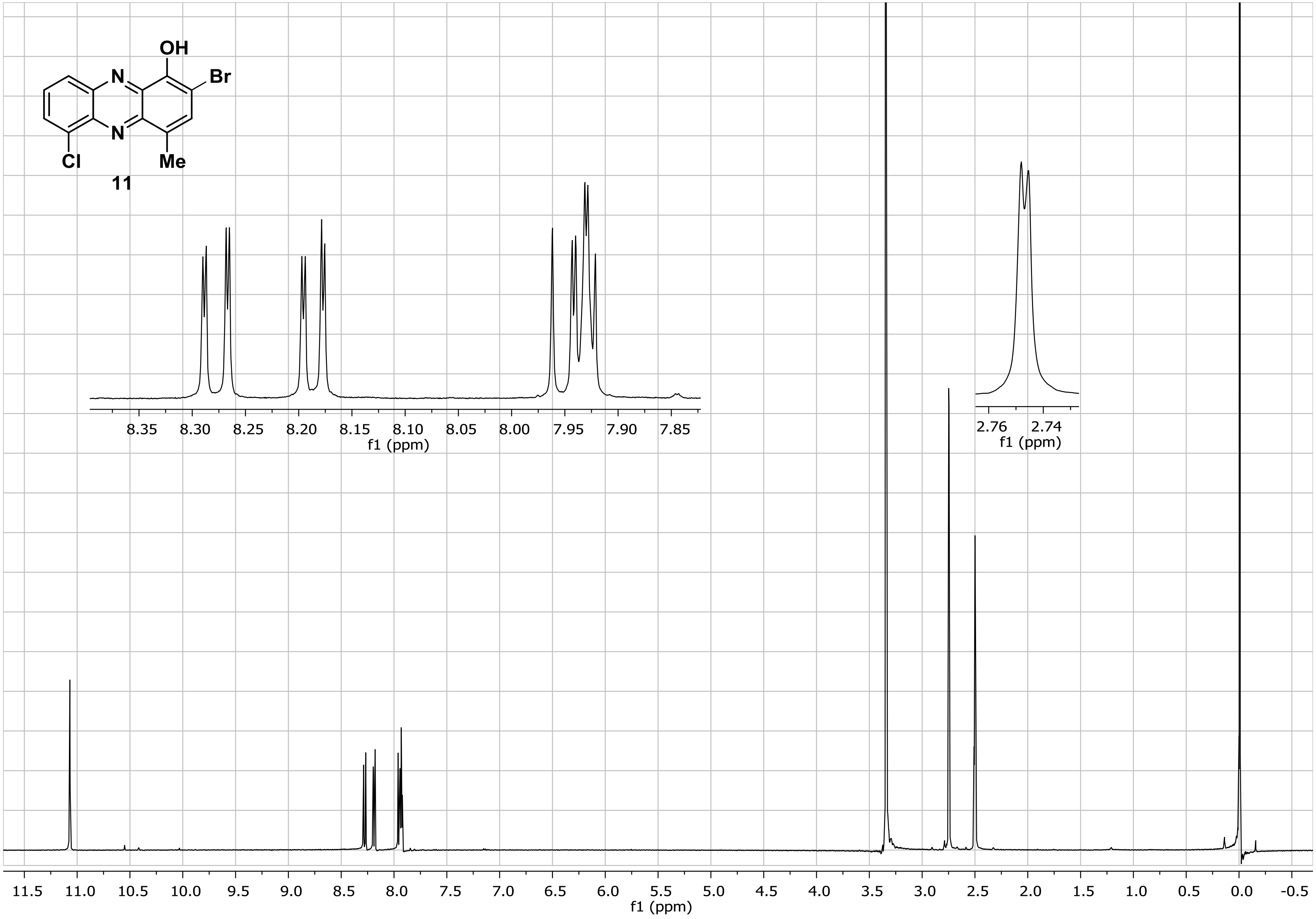

OH
N
Br
N
Cl
Me
11
8.35
8.30
8.25
8.20
8.15
8.10
8.05
8.00
7.95
7.90
7.85
f1 (ppm)
2.76
2.74
f1 (ppm)
11.5
11.0
10.5
10.0
9.5
9.0
8.5
8.0
7.5
7.0
6.5
6.0
5.5
5.0
4.5
4.0
3.5
3.0
2.5
2.0
1.5
1.0
0.5
0.0
-0.5
f1 (ppm)

NMR-17

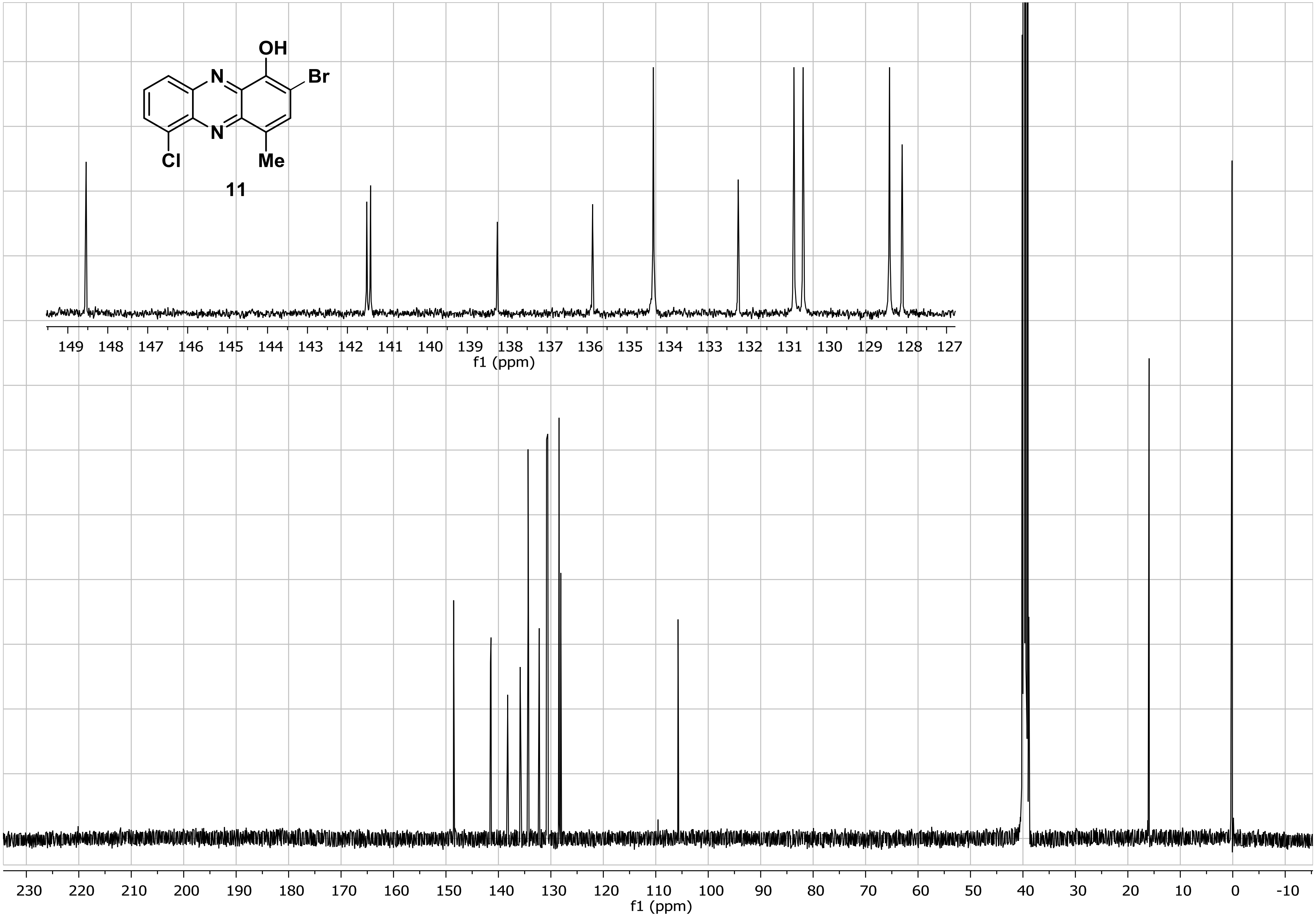
OH
Br
N
N
Cl
Me
11
149 148 147 146 145 144 143 142 141 140 139 138 137 136 135 134 133 132 131 130 129 128 127
f1 (ppm)
230 220 210 200 190 180 170 160 150 140 130 120 110 100 90 80 70 60 50 40 30 20 10 0 -10
f1 (ppm)

NMR-18

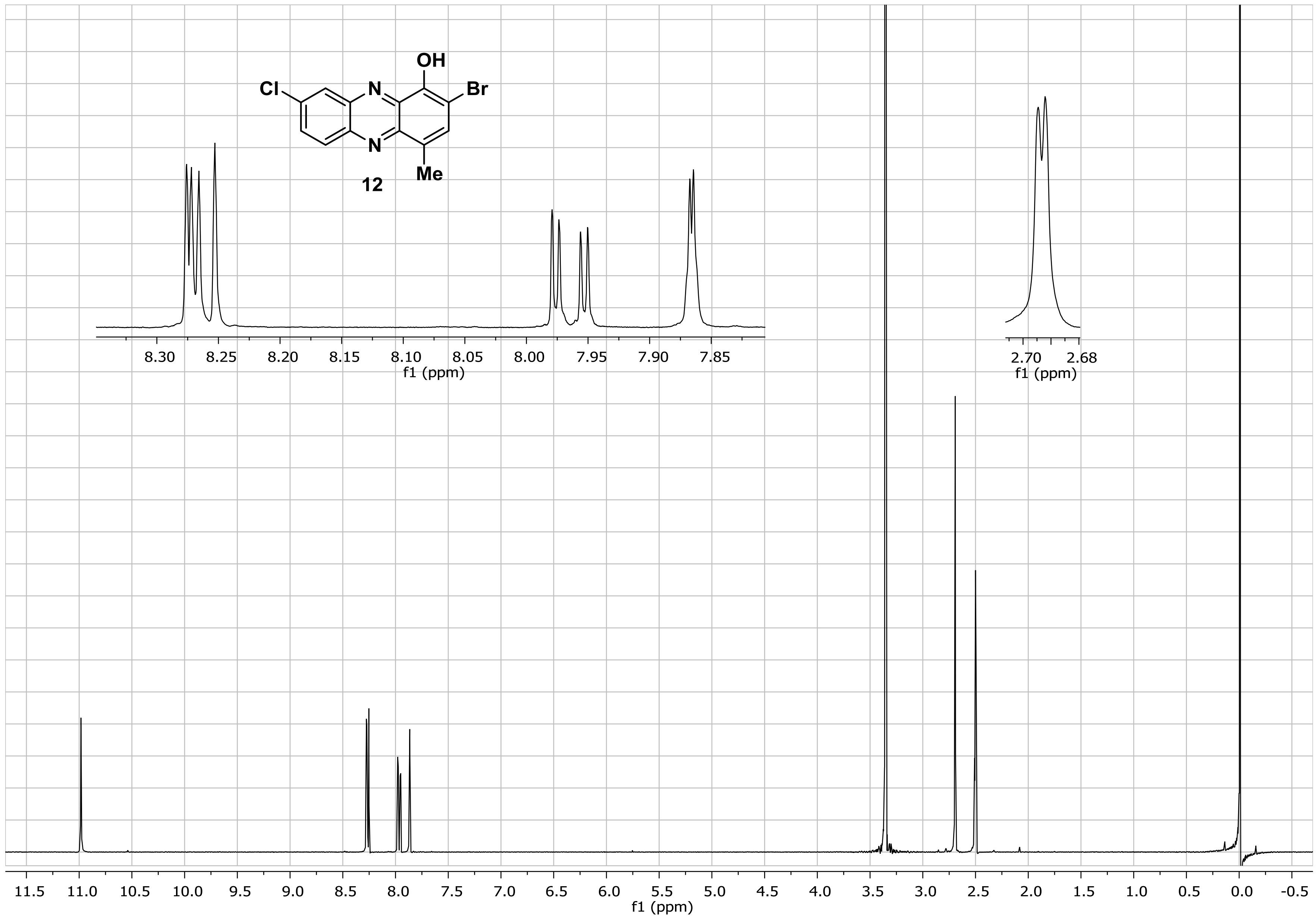

OH
Cl
N
Br
N
Me
12
8.30 8.25 8.20 8.15 8.10 8.05 8.00 7.95 7.90 7.85
f1 (ppm)
2.70 2.68
f1 (ppm)
11.5 11.0 10.5 10.0 9.5 9.0 8.5 8.0 7.5 7.0 6.5 6.0 5.5 5.0 4.5 4.0 3.5 3.0 2.5 2.0 1.5 1.0 0.5 0.0 -0.5
f1 (ppm)

NMR-19

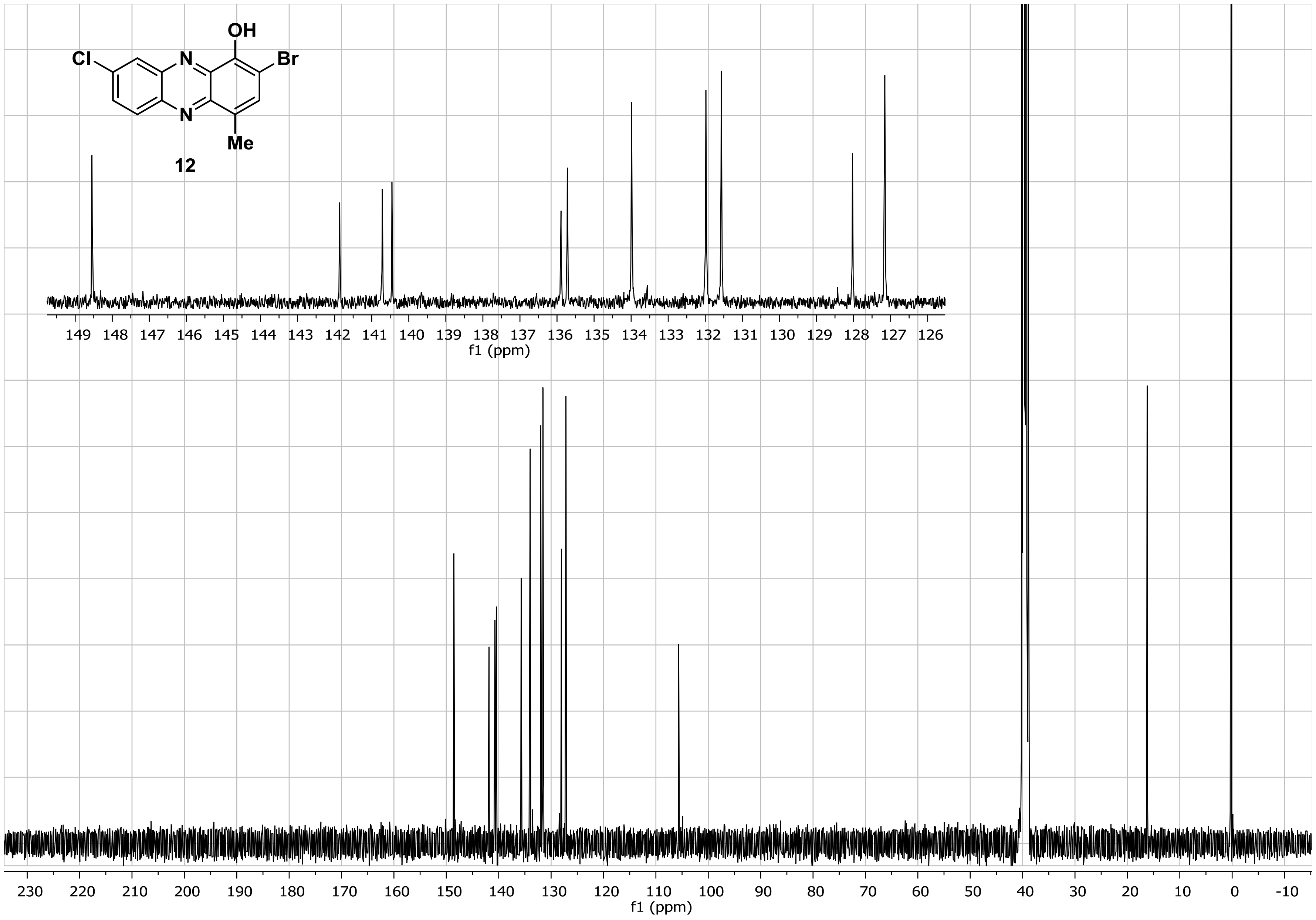
OH
Cl
N
N
Br
Me
12
149 148 147 146 145 144 143 142 141 140 139 138 137 136 135 134 133 132 131 130 129 128 127 126
f1 (ppm)
230 220 210 200 190 180 170 160 150 140 130 120 110 100 90 80 70 60 50 40 30 20 10 0 -10
f1 (ppm)

NMR-20

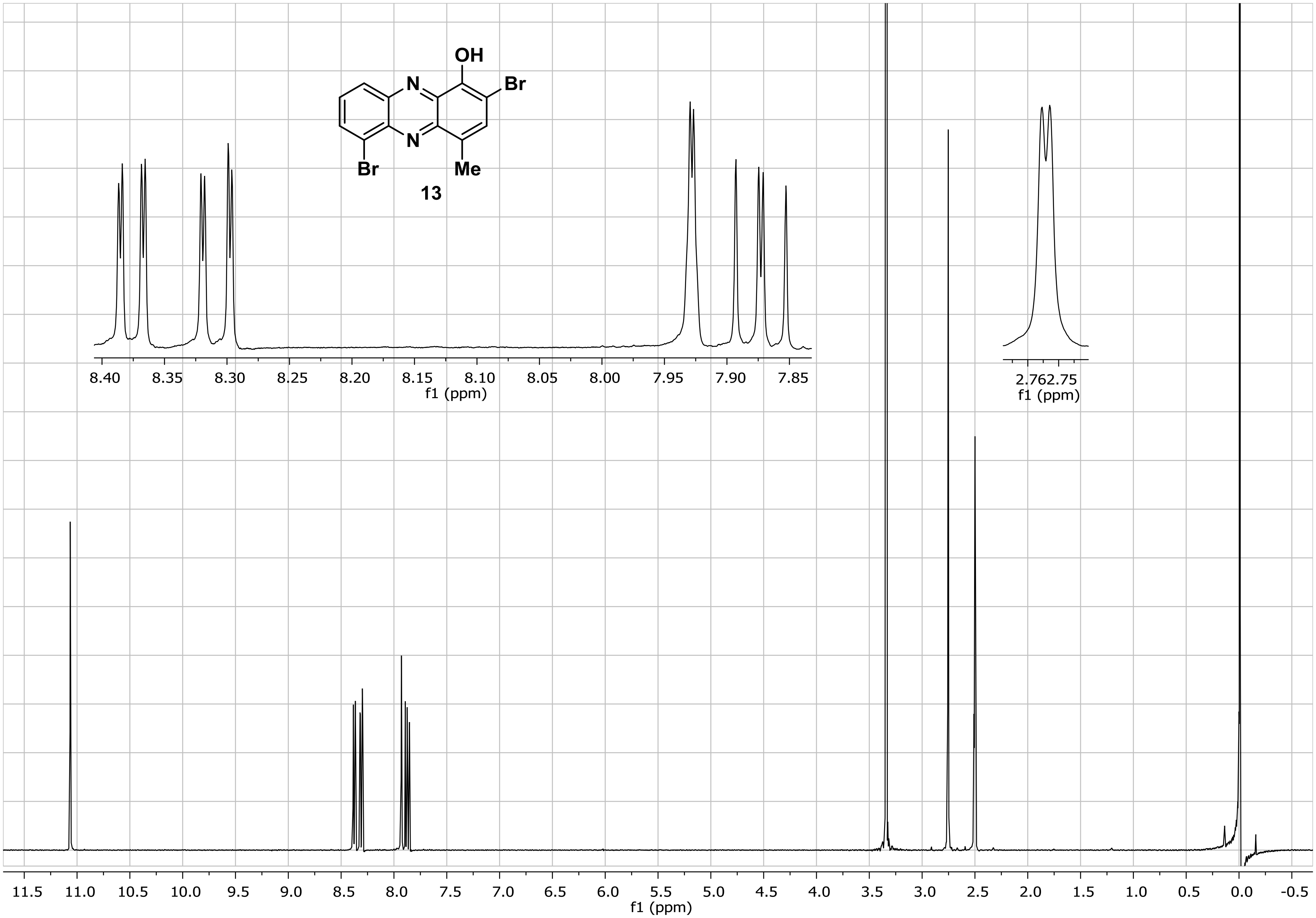
OH
Br
N
N
Br
Me
13
8.40 8.35 8.30 8.25 8.20 8.15 8.10 8.05 8.00 7.95 7.90 7.85
f1 (ppm)
2.76 2.75
f1 (ppm)
11.5 11.0 10.5 10.0 9.5 9.0 8.5 8.0 7.5 7.0 6.5 6.0 5.5 5.0 4.5 4.0 3.5 3.0 2.5 2.0 1.5 1.0 0.5 0.0 -0.5
f1 (ppm)

NMR-21

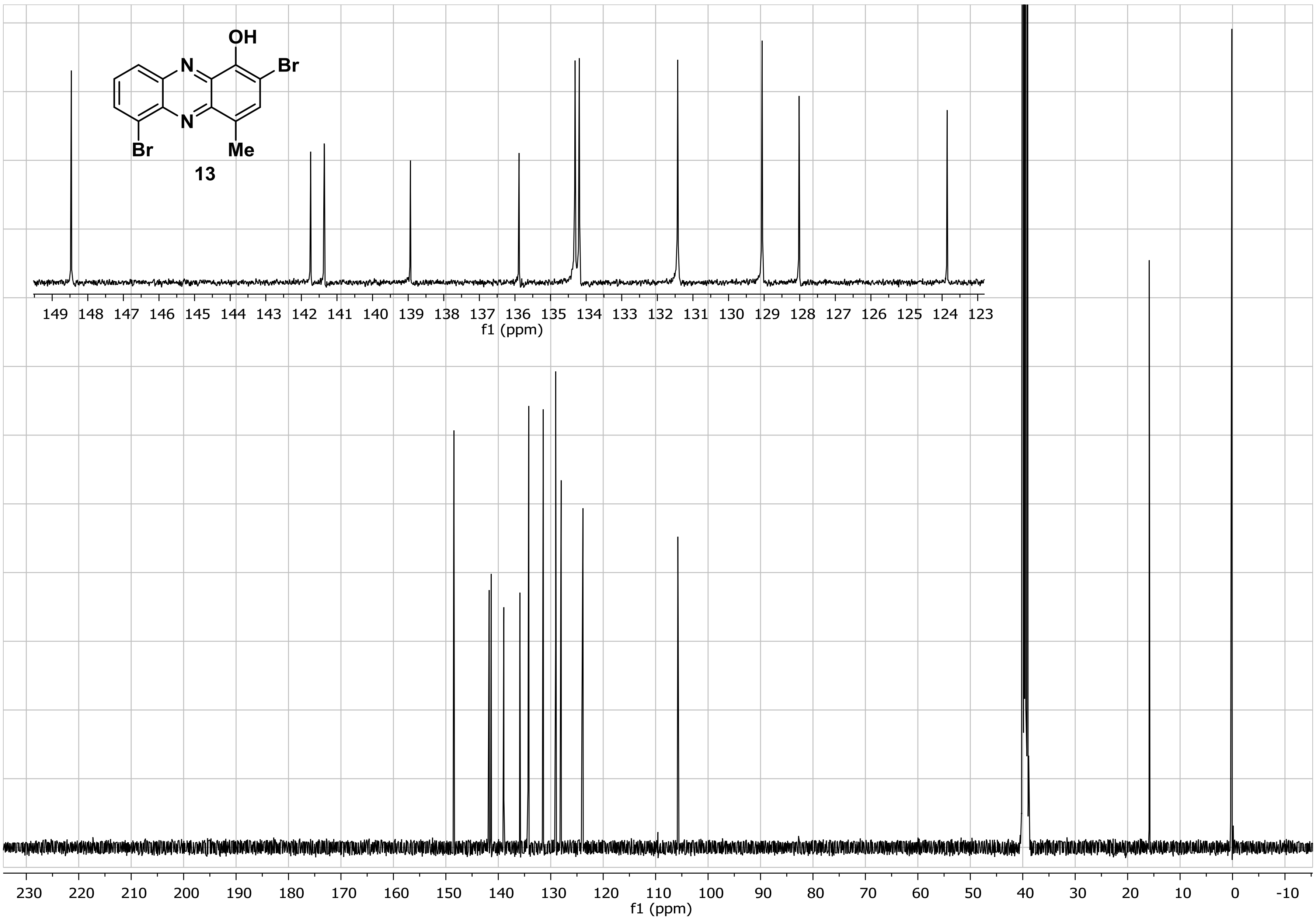
OH
Br
N
N
Br
Me
13
149 148 147 146 145 144 143 142 141 140 139 138 137 136 135 134 133 132 131 130 129 128 127 126 125 124 123
f1 (ppm)
230 220 210 200 190 180 170 160 150 140 130 120 110 100 90 80 70 60 50 40 30 20 10 0 -10
f1 (ppm)

NMR-22

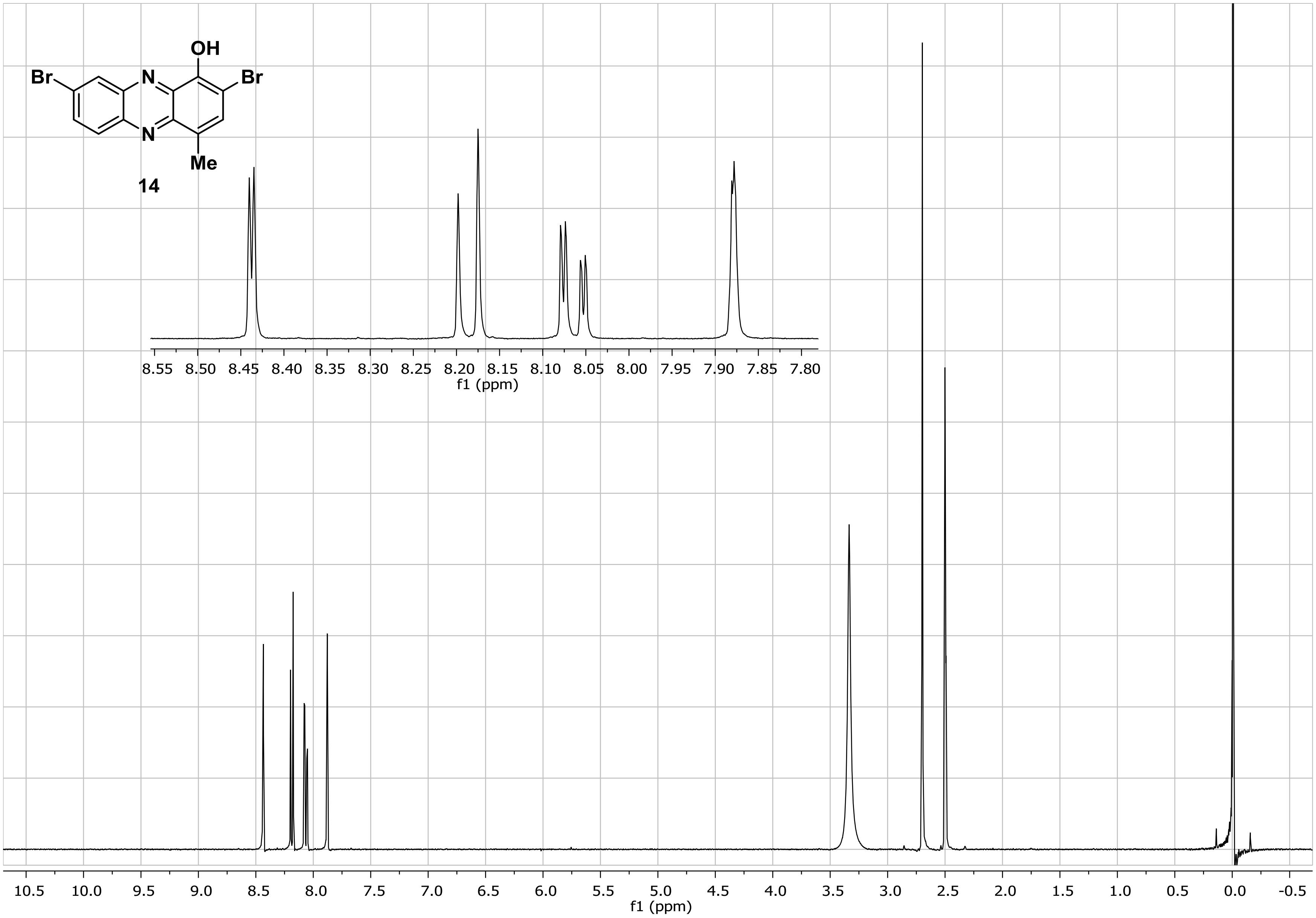
OH
Br
Br
N
N
Me
14
8.55 8.50 8.45 8.40 8.35 8.30 8.25 8.20 8.15 8.10 8.05 8.00 7.95 7.90 7.85 7.80
f1 (ppm)
10.5 10.0 9.5 9.0 8.5 8.0 7.5 7.0 6.5 6.0 5.5 5.0 4.5 4.0 3.5 3.0 2.5 2.0 1.5 1.0 0.5 0.0 -0.5
f1 (ppm)

NMR-23

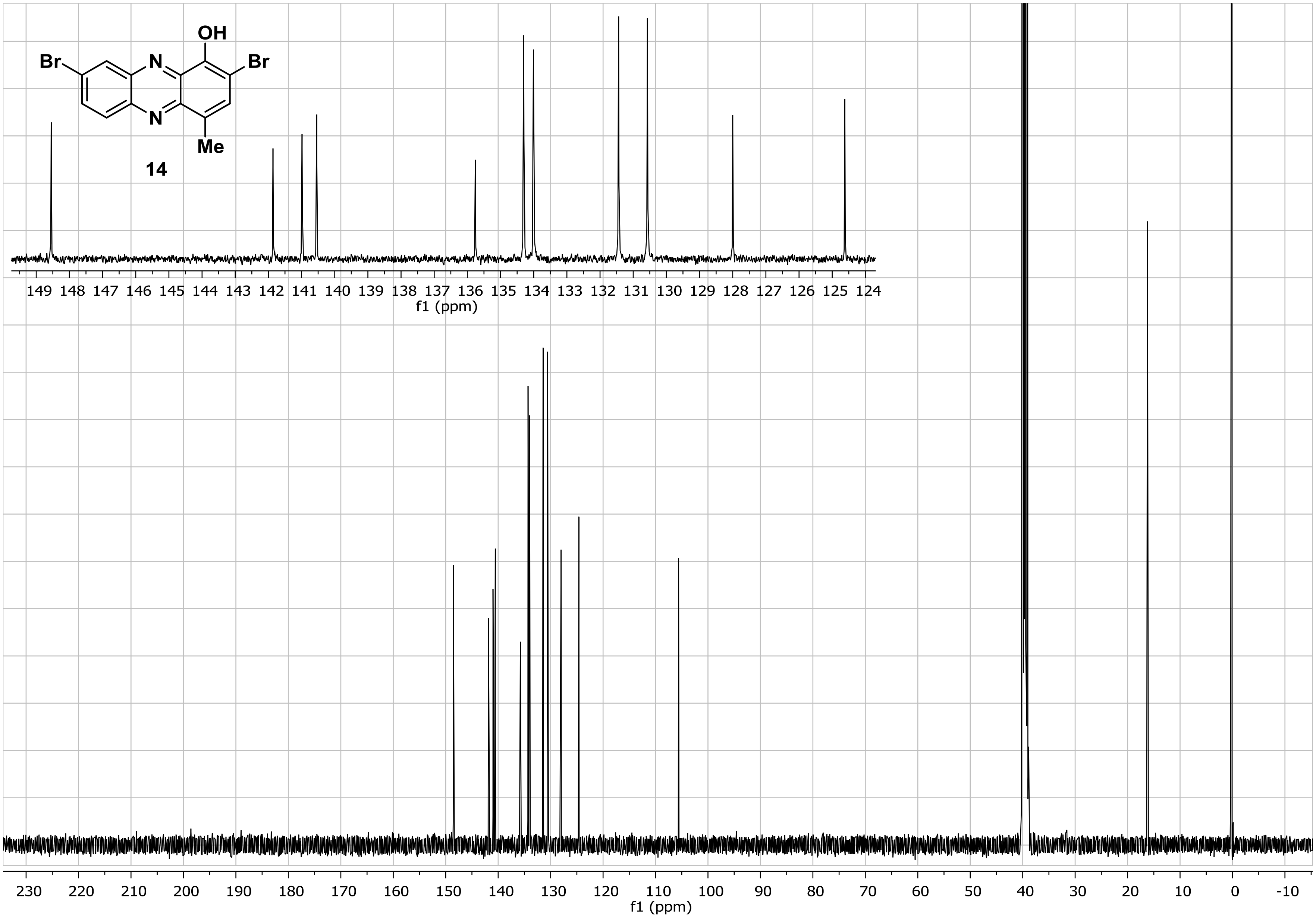
OH
Br
Br
N
N
Me
14
149 148 147 146 145 144 143 142 141 140 139 138 137 136 135 134 133 132 131 130 129 128 127 126 125 124
f1 (ppm)
230 220 210 200 190 180 170 160 150 140 130 120 110 100 90 80 70 60 50 40 30 20 10 0 -10
f1 (ppm)

NMR-24

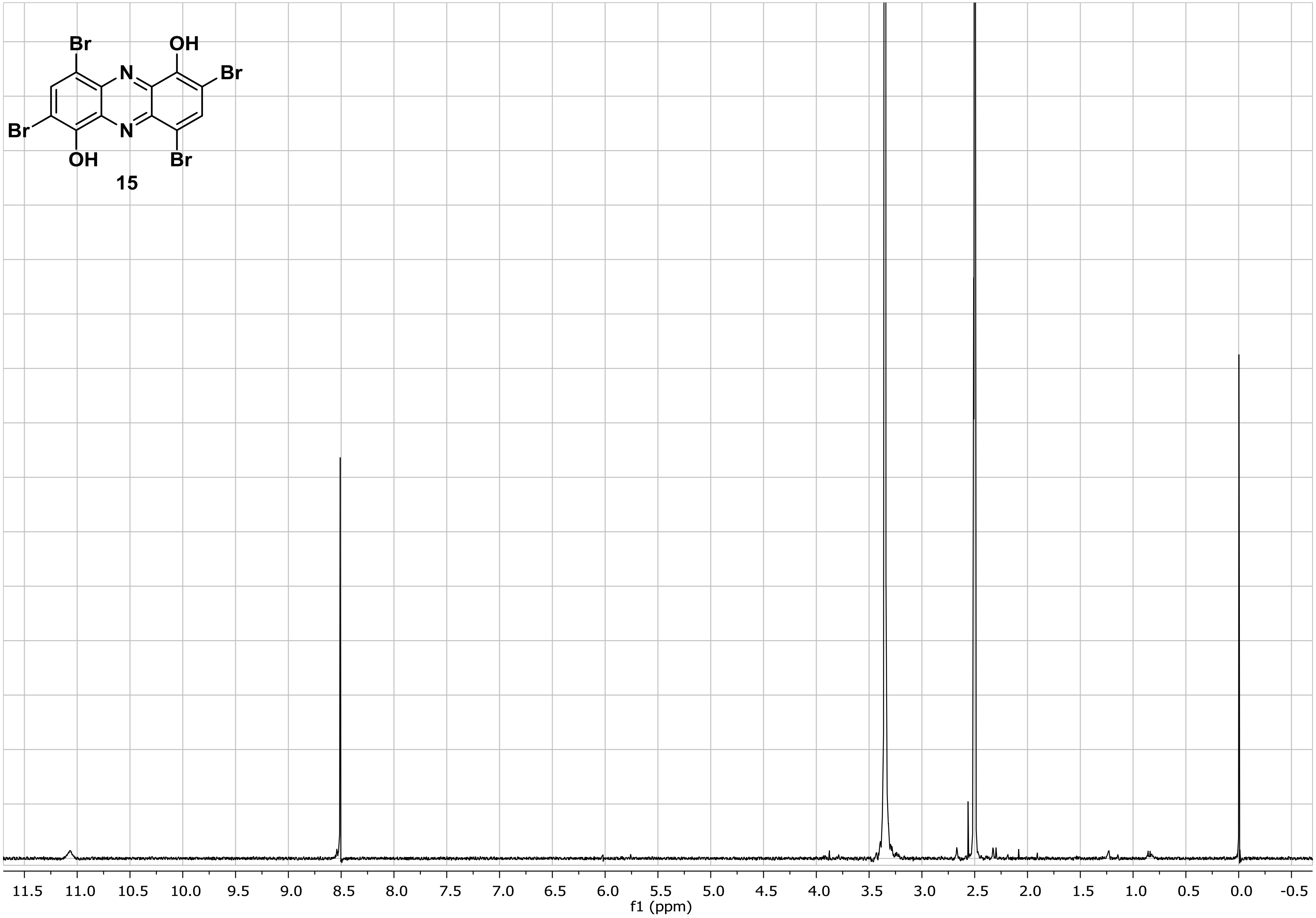
Br
OH
Br
N
N
Br
OH
Br
15
11.5 11.0 10.5 10.0 9.5 9.0 8.5 8.0 7.5 7.0 6.5 6.0 5.5 5.0 4.5 4.0 3.5 3.0 2.5 2.0 1.5 1.0 0.5 0.0 -0.5
f1 (ppm)

NMR-25

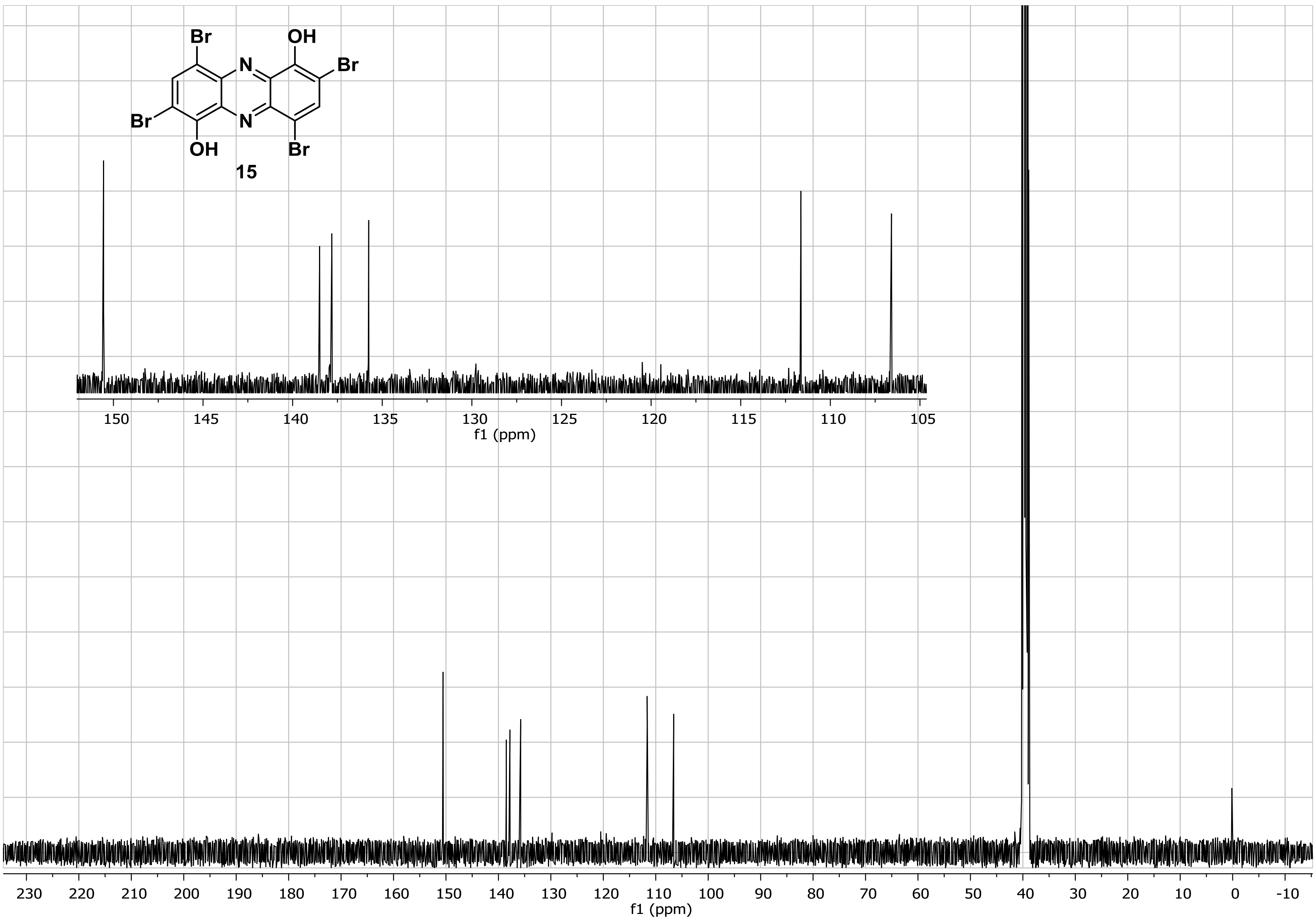
Br
OH
Br
N
N
Br
OH
Br
15
150
145
140
135
130
125
120
115
110
105
f1 (ppm)
230
220
210
200
190
180
170
160
150
140
130
120
110
100
90
80
70
60
50
40
30
20
10
0
-10
f1 (ppm)

NMR-26

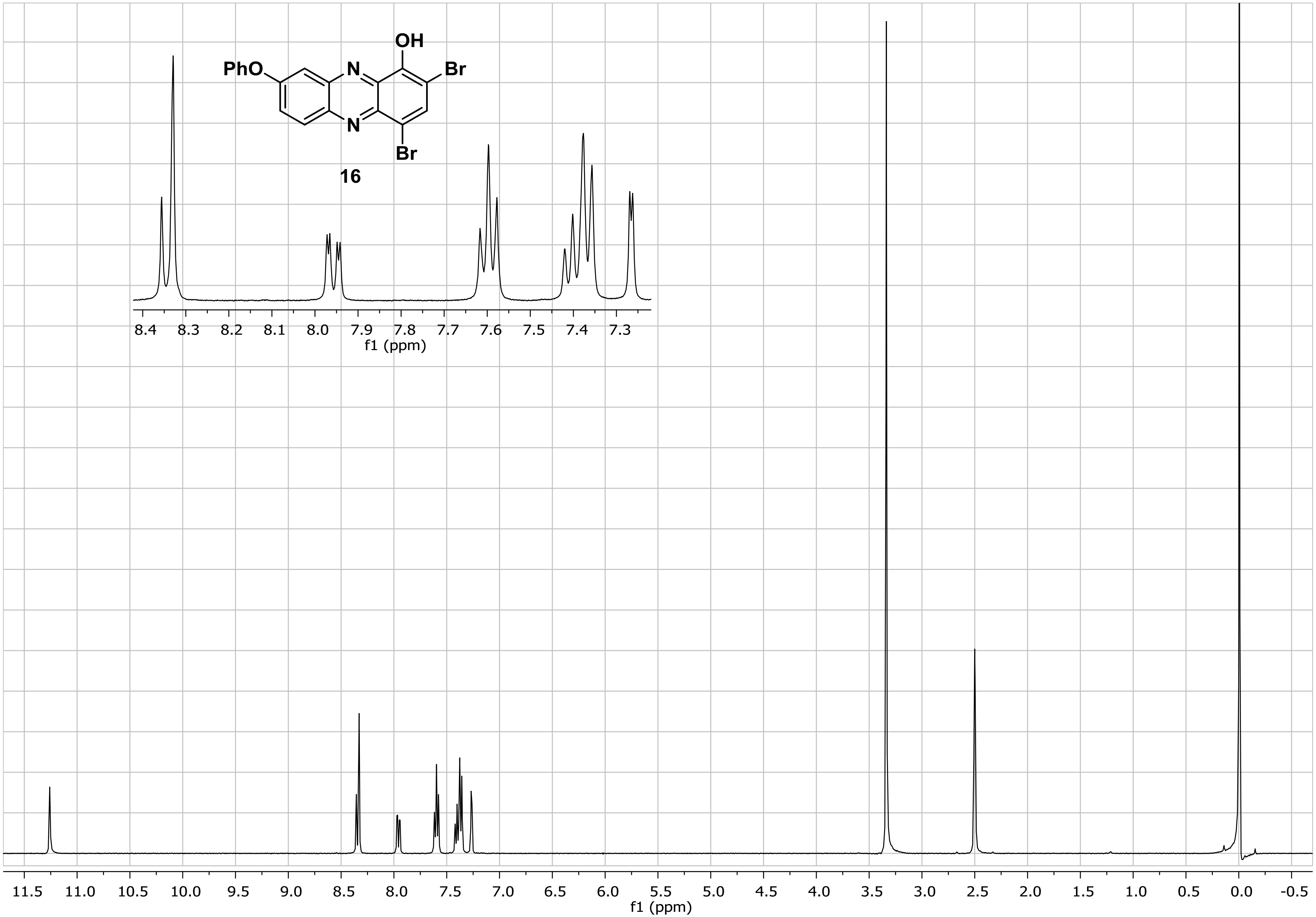
PhO
OH
Br
N
N
Br
16
8.4 8.3 8.2 8.1 8.0 7.9 7.8 7.7 7.6 7.5 7.4 7.3
f1 (ppm)
11.5 11.0 10.5 10.0 9.5 9.0 8.5 8.0 7.5 7.0 6.5 6.0 5.5 5.0 4.5 4.0 3.5 3.0 2.5 2.0 1.5 1.0 0.5 0.0 -0.5
f1 (ppm)

NMR-27

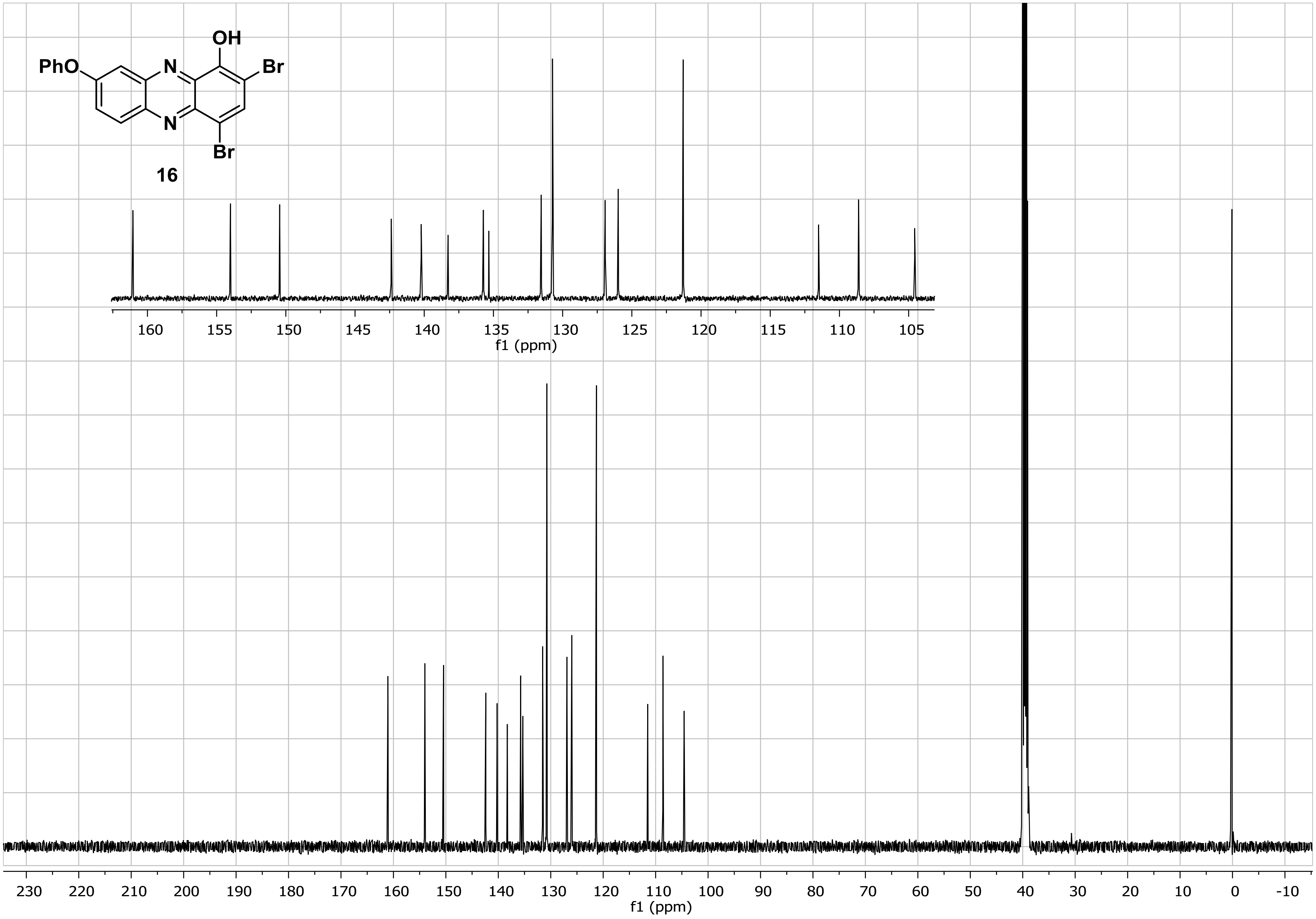

NMR-28

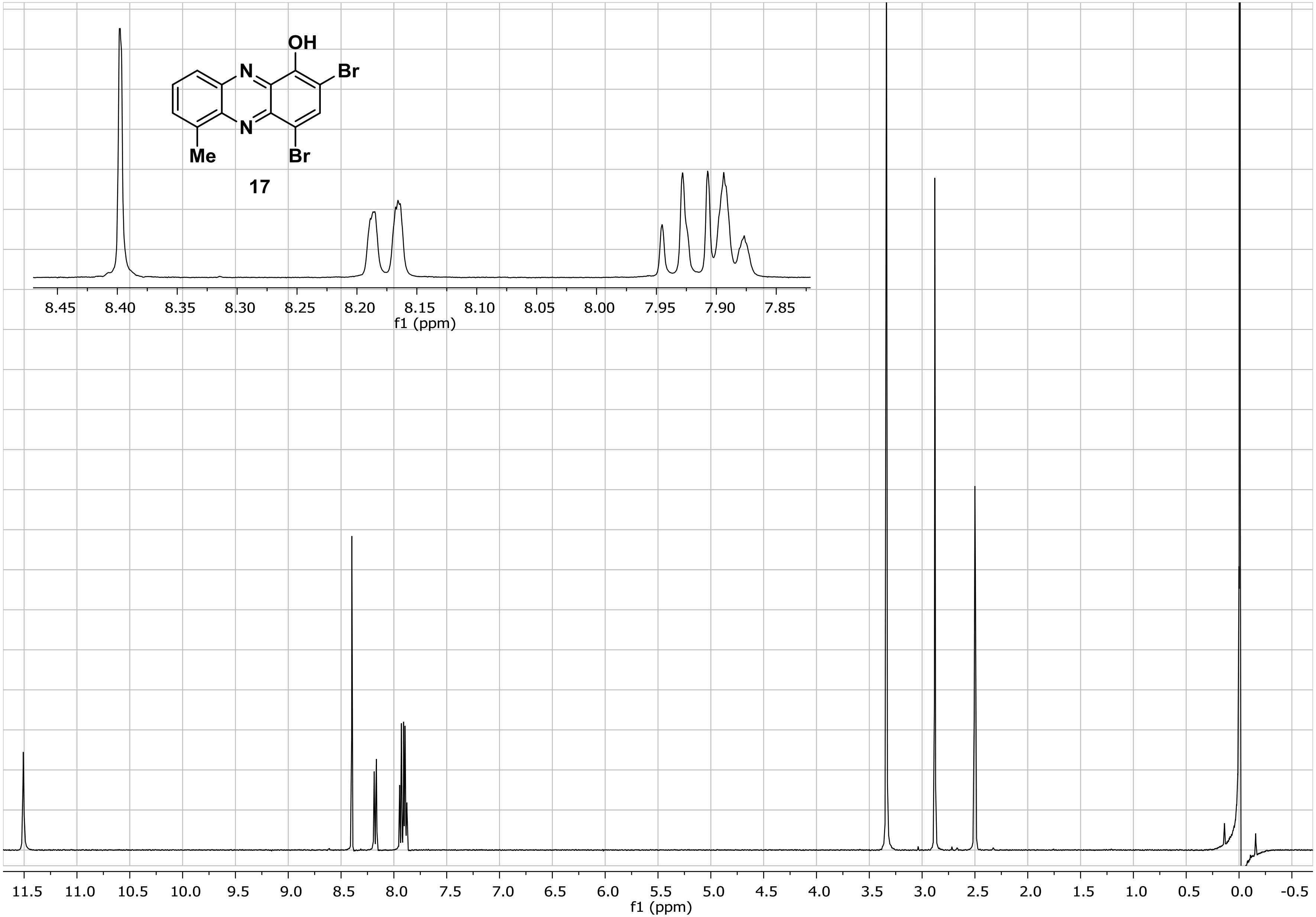

OH
Br
N
N
Me
Br
17
8.45
8.40
8.35
8.30
8.25
8.20
8.15
8.10
8.05
8.00
7.95
7.90
7.85
f1 (ppm)
11.5
11.0
10.5
10.0
9.5
9.0
8.5
8.0
7.5
7.0
6.5
6.0
5.5
5.0
4.5
4.0
3.5
3.0
2.5
2.0
1.5
1.0
0.5
0.0
-0.5
f1 (ppm)

NMR-29

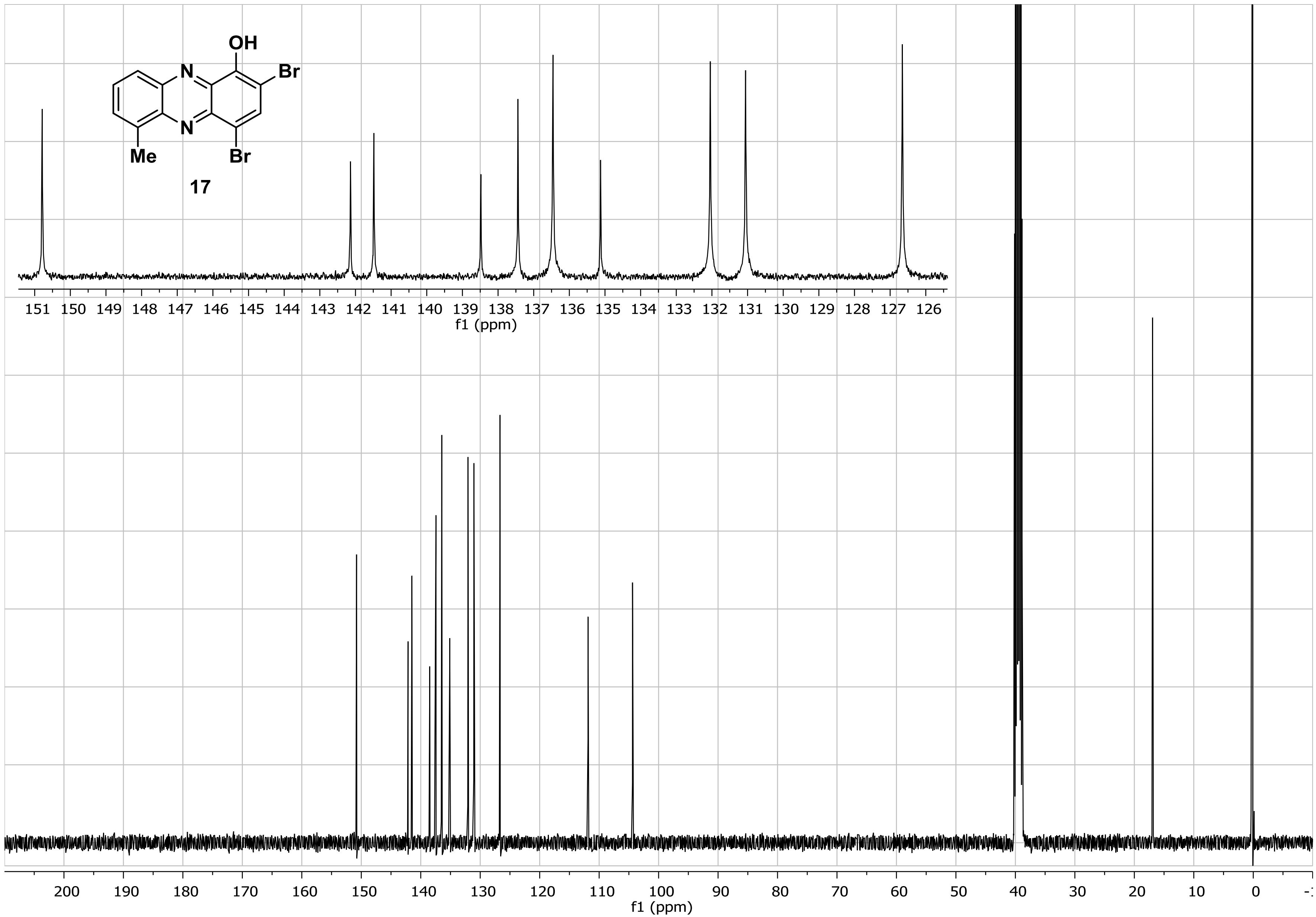

NMR-30

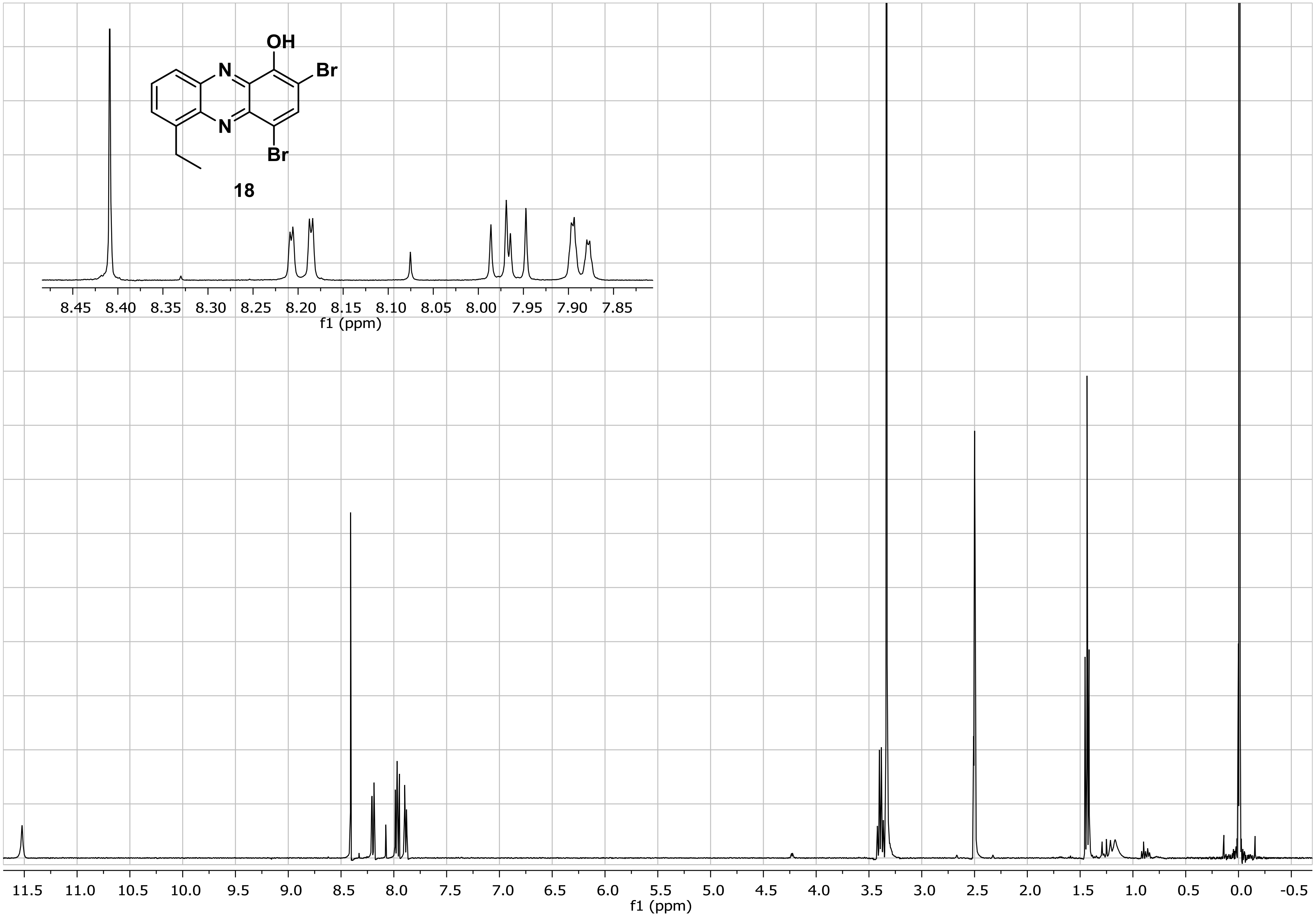
OH
Br
N
N
Br
18
8.45 8.40 8.35 8.30 8.25 8.20 8.15 8.10 8.05 8.00 7.95 7.90 7.85
f1 (ppm)
11.5 11.0 10.5 10.0 9.5 9.0 8.5 8.0 7.5 7.0 6.5 6.0 5.5 5.0 4.5 4.0 3.5 3.0 2.5 2.0 1.5 1.0 0.5 0.0 -0.5
f1 (ppm)

NMR-31

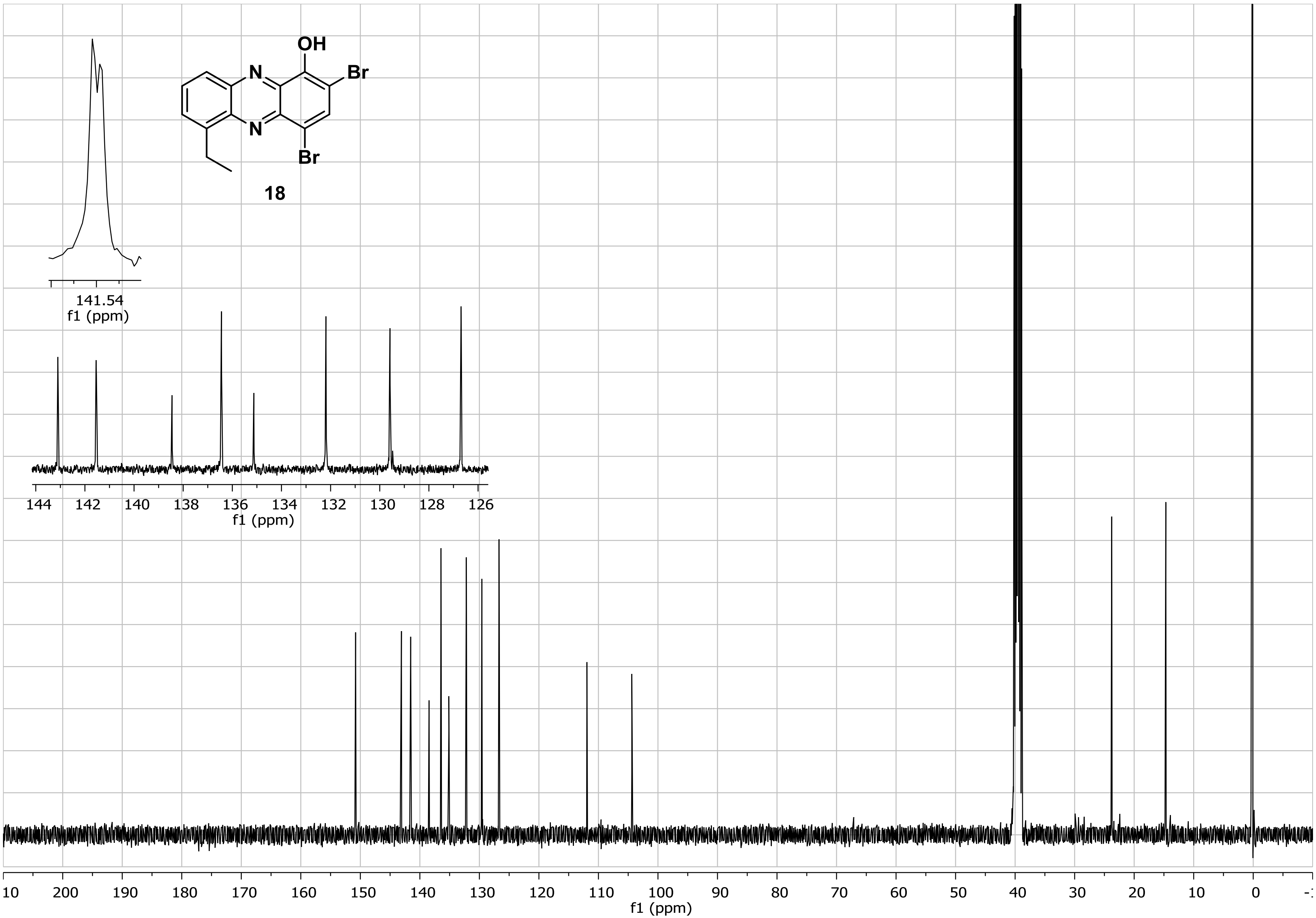
OH
Br
N
N
Br
18
141.54
f1 (ppm)
144 142 140 138 136 134 132 130 128 126
f1 (ppm)
10 200 190 180 170 160 150 140 130 120 110 100 90 80 70 60 50 40 30 20 10 0
f1 (ppm)

NMR-32

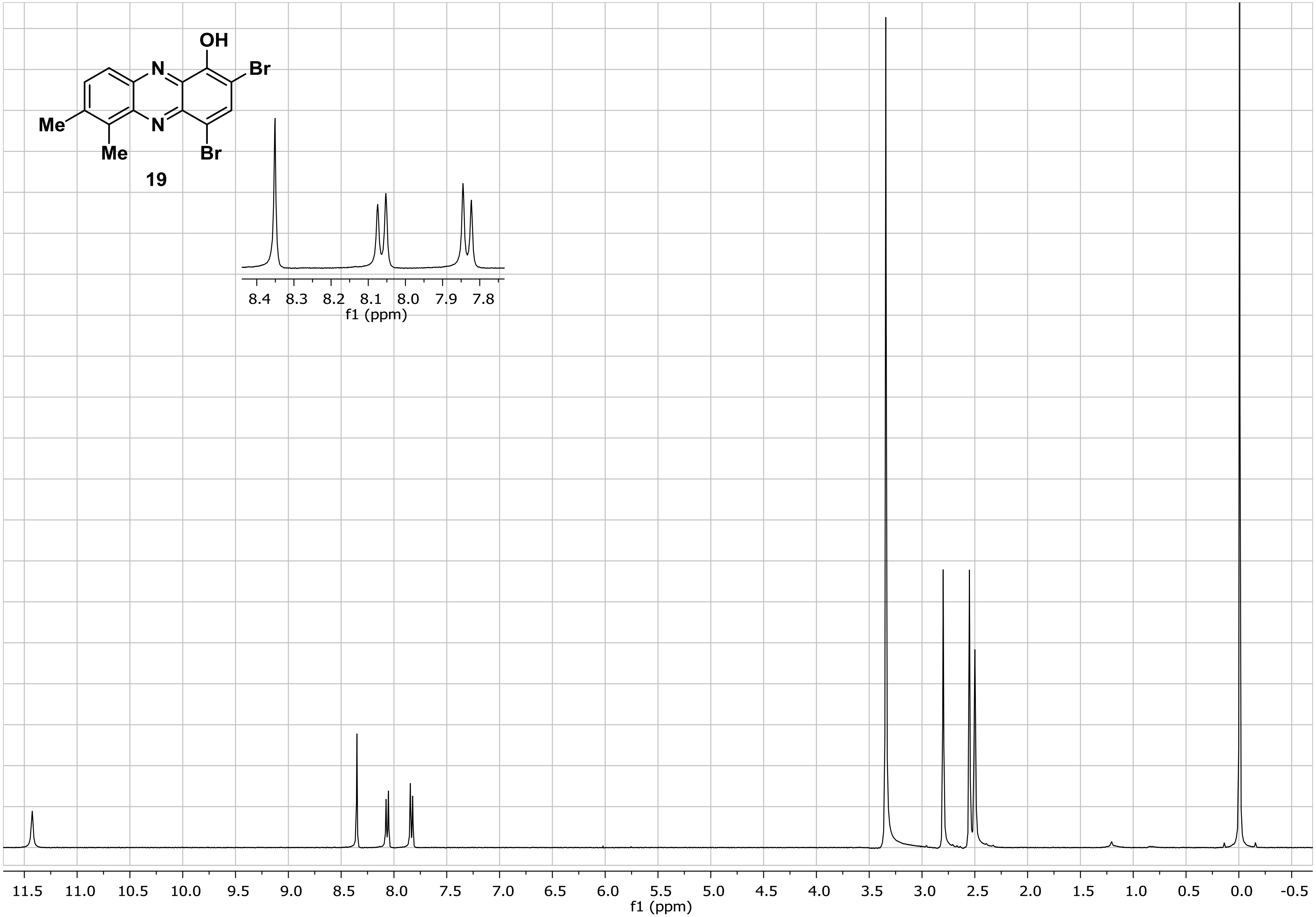
OH
Br
N
N
Me
Me
Br
19
8.4 8.3 8.2 8.1 8.0 7.9 7.8
f1 (ppm)
11.5 11.0 10.5 10.0 9.5 9.0 8.5 8.0 7.5 7.0 6.5 6.0 5.5 5.0 4.5 4.0 3.5 3.0 2.5 2.0 1.5 1.0 0.5 0.0 -0.5
f1 (ppm)

NMR-33

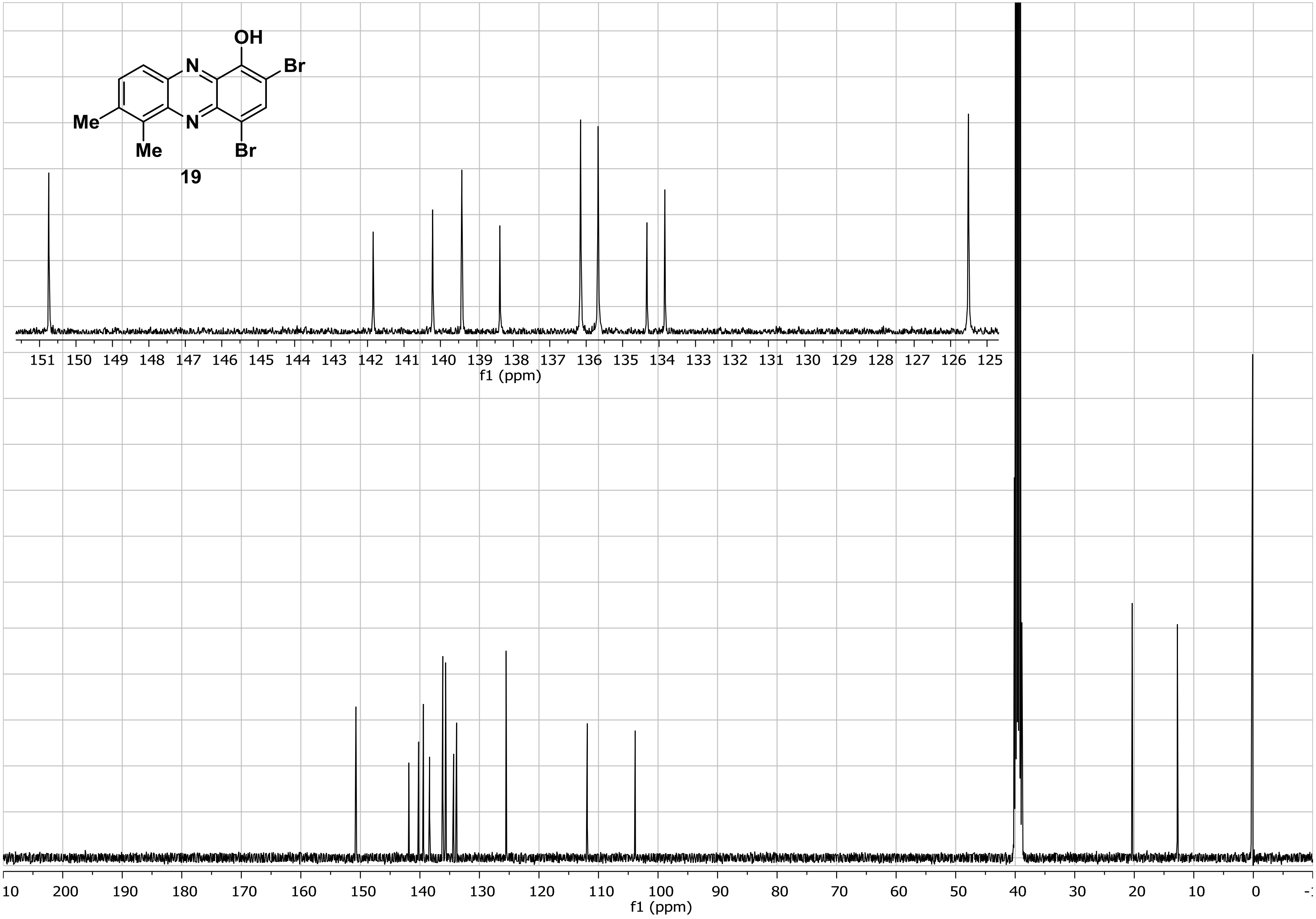
OH
Br
N
N
Me
Me
Br
19
151 150 149 148 147 146 145 144 143 142 141 140 139 138 137 136 135 134 133 132 131 130 129 128 127 126 125
f1 (ppm)
10 200 190 180 170 160 150 140 130 120 110 100 90 80 70 60 50 40 30 20 10 0
f1 (ppm)

NMR-34

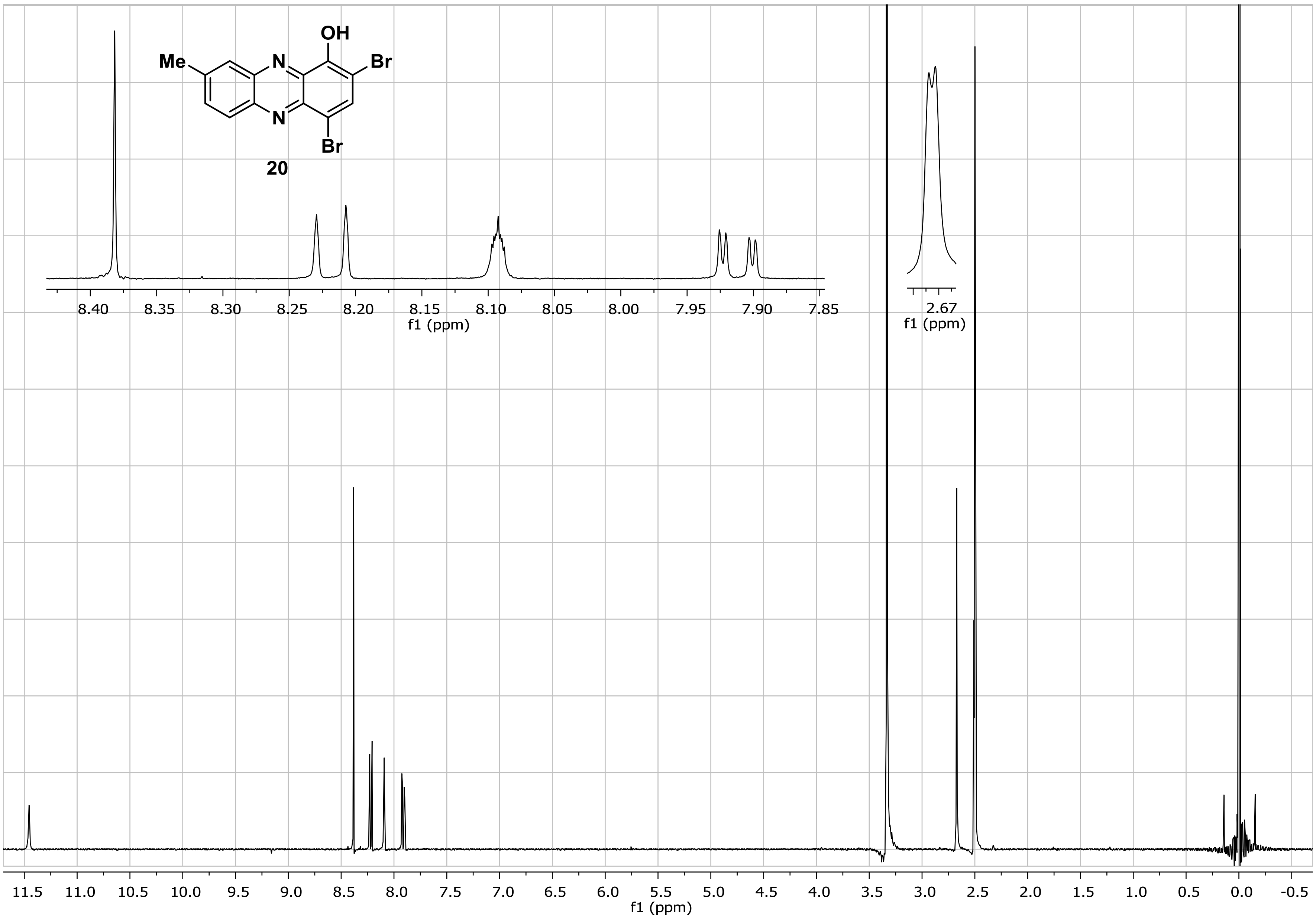

NMR-35

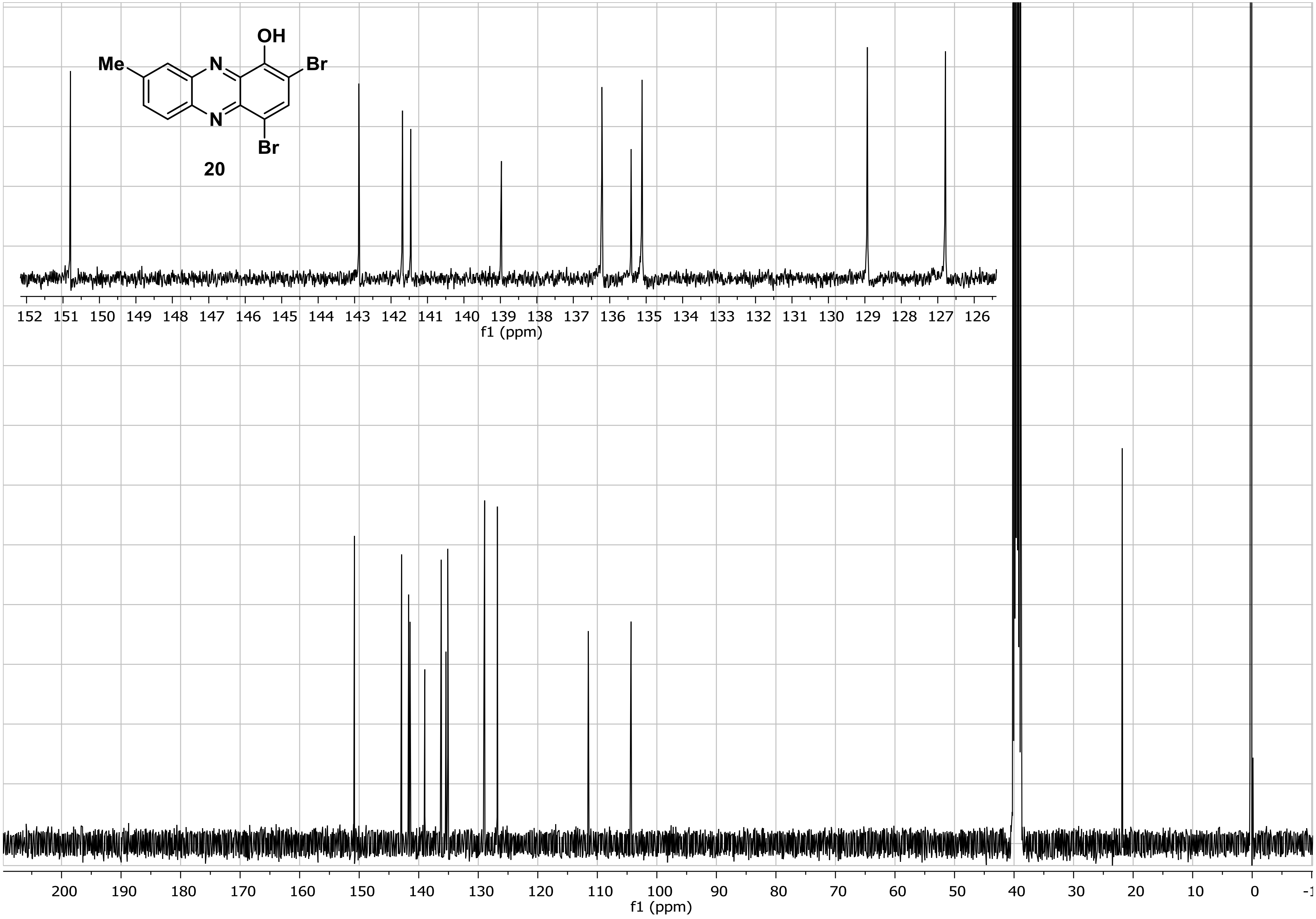

OH
Me
N
N
Br
Br
20
152 151 150 149 148 147 146 145 144 143 142 141 140 139 138 137 136 135 134 133 132 131 130 129 128 127 126
f1 (ppm)
200 190 180 170 160 150 140 130 120 110 100 90 80 70 60 50 40 30 20 10 0
f1 (ppm)

NMR-36

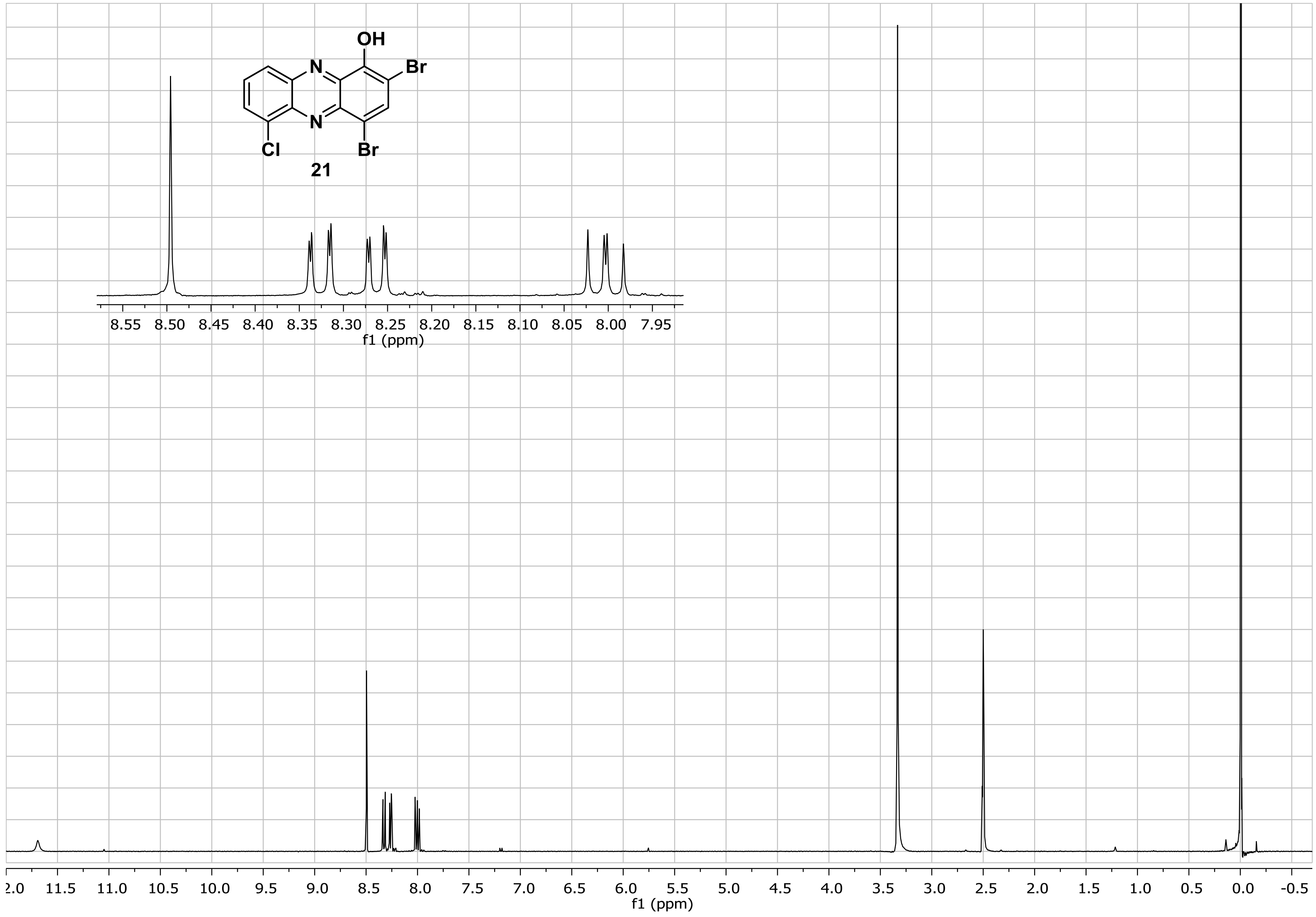
OH
Br
N
N
Cl
Br
21
8.55 8.50 8.45 8.40 8.35 8.30 8.25 8.20 8.15 8.10 8.05 8.00 7.95
f1 (ppm)
2.0 11.5 11.0 10.5 10.0 9.5 9.0 8.5 8.0 7.5 7.0 6.5 6.0 5.5 5.0 4.5 4.0 3.5 3.0 2.5 2.0 1.5 1.0 0.5 0.0 -0.5
f1 (ppm)

NMR-37

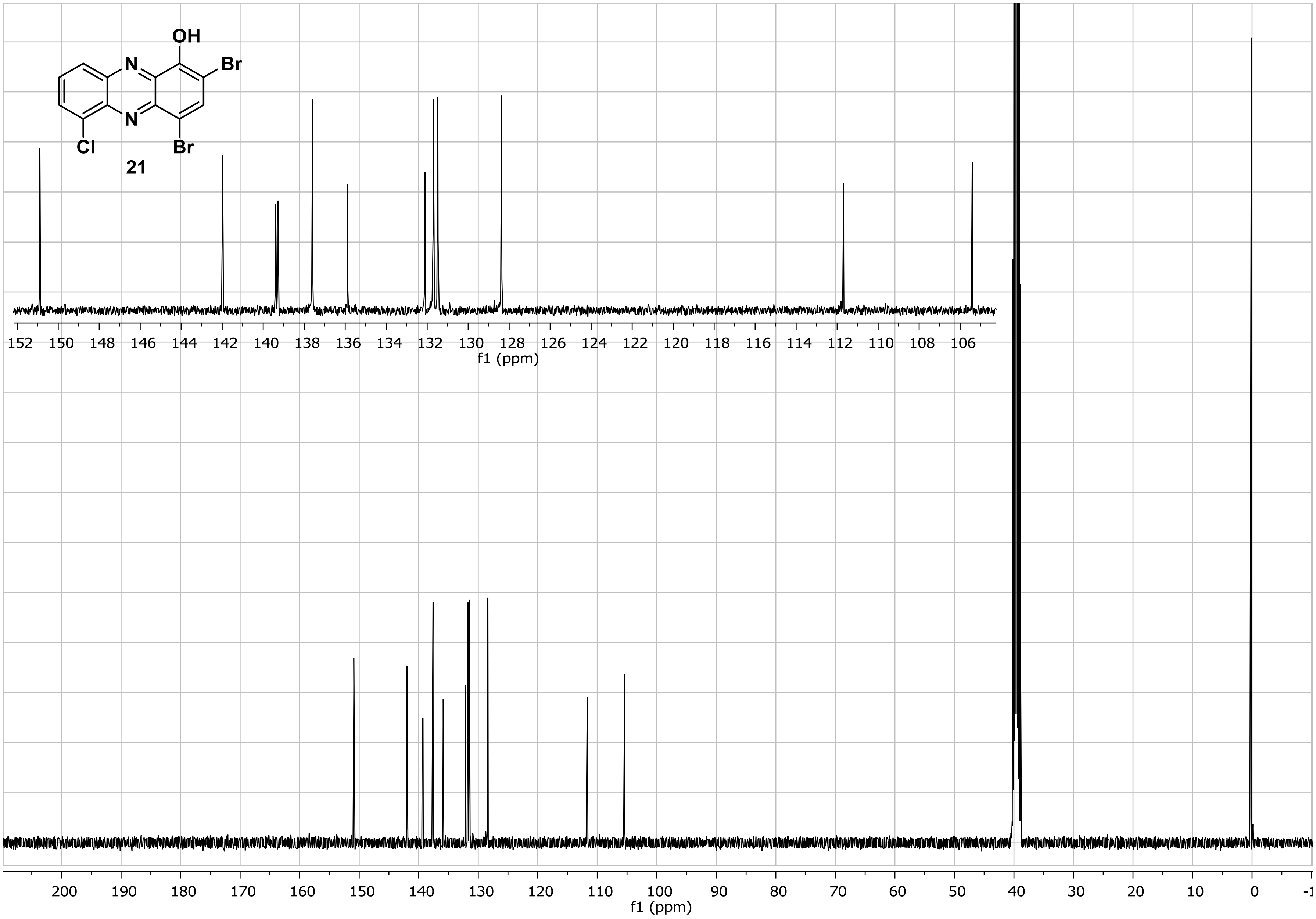
OH
Br
N
N
Cl
Br
21
152 150 148 146 144 142 140 138 136 134 132 130 128 126 124 122 120 118 116 114 112 110 108 106
f1 (ppm)
200 190 180 170 160 150 140 130 120 110 100 90 80 70 60 50 40 30 20 10 0
f1 (ppm)

NMR-38

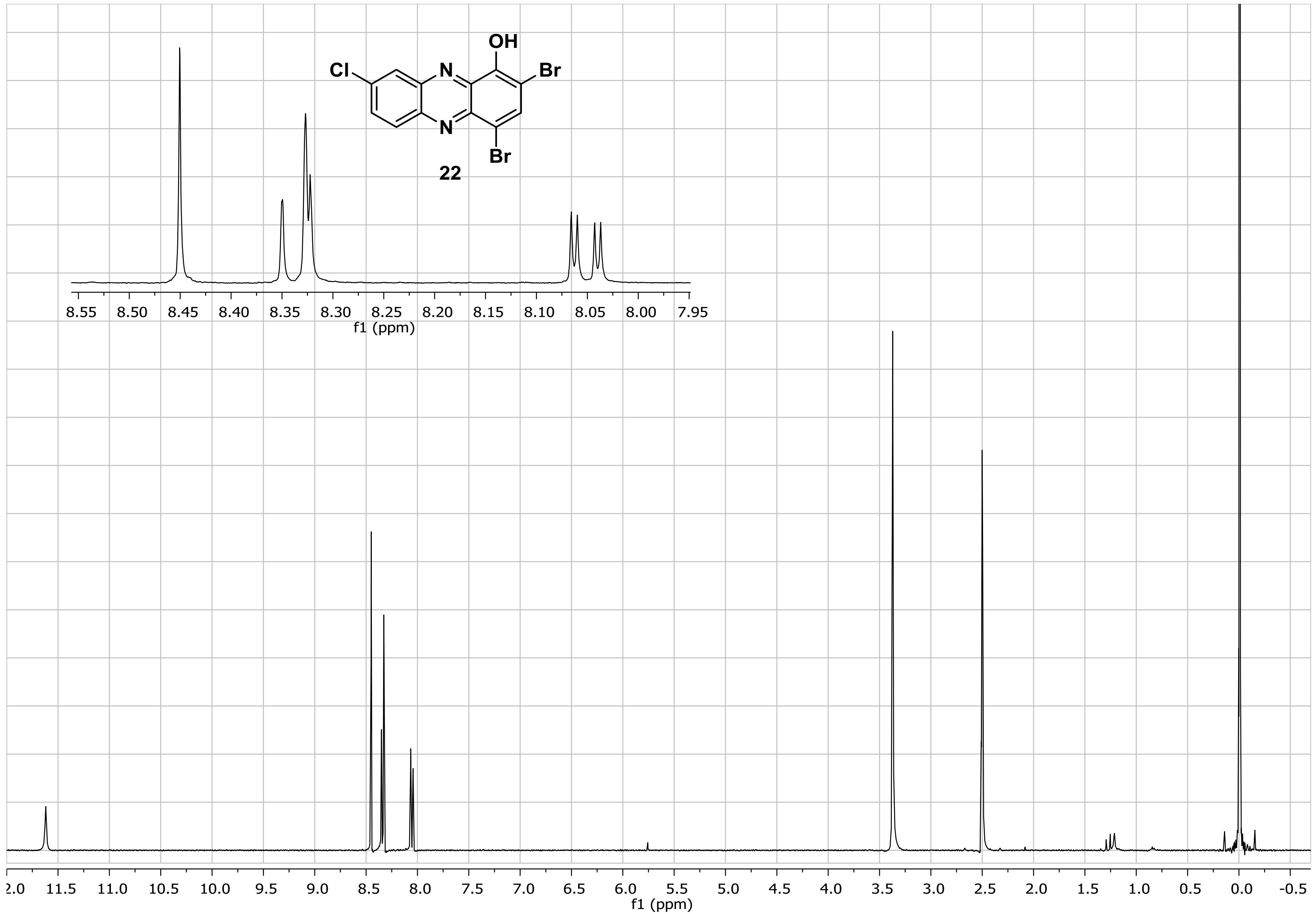
OH
Cl
N
Br
N
Br
22
8.55 8.50 8.45 8.40 8.35 8.30 8.25 8.20 8.15 8.10 8.05 8.00 7.95
f1 (ppm)
2.0 11.5 11.0 10.5 10.0 9.5 9.0 8.5 8.0 7.5 7.0 6.5 6.0 5.5 5.0 4.5 4.0 3.5 3.0 2.5 2.0 1.5 1.0 0.5 0.0 -0.5
f1 (ppm)

NMR-39

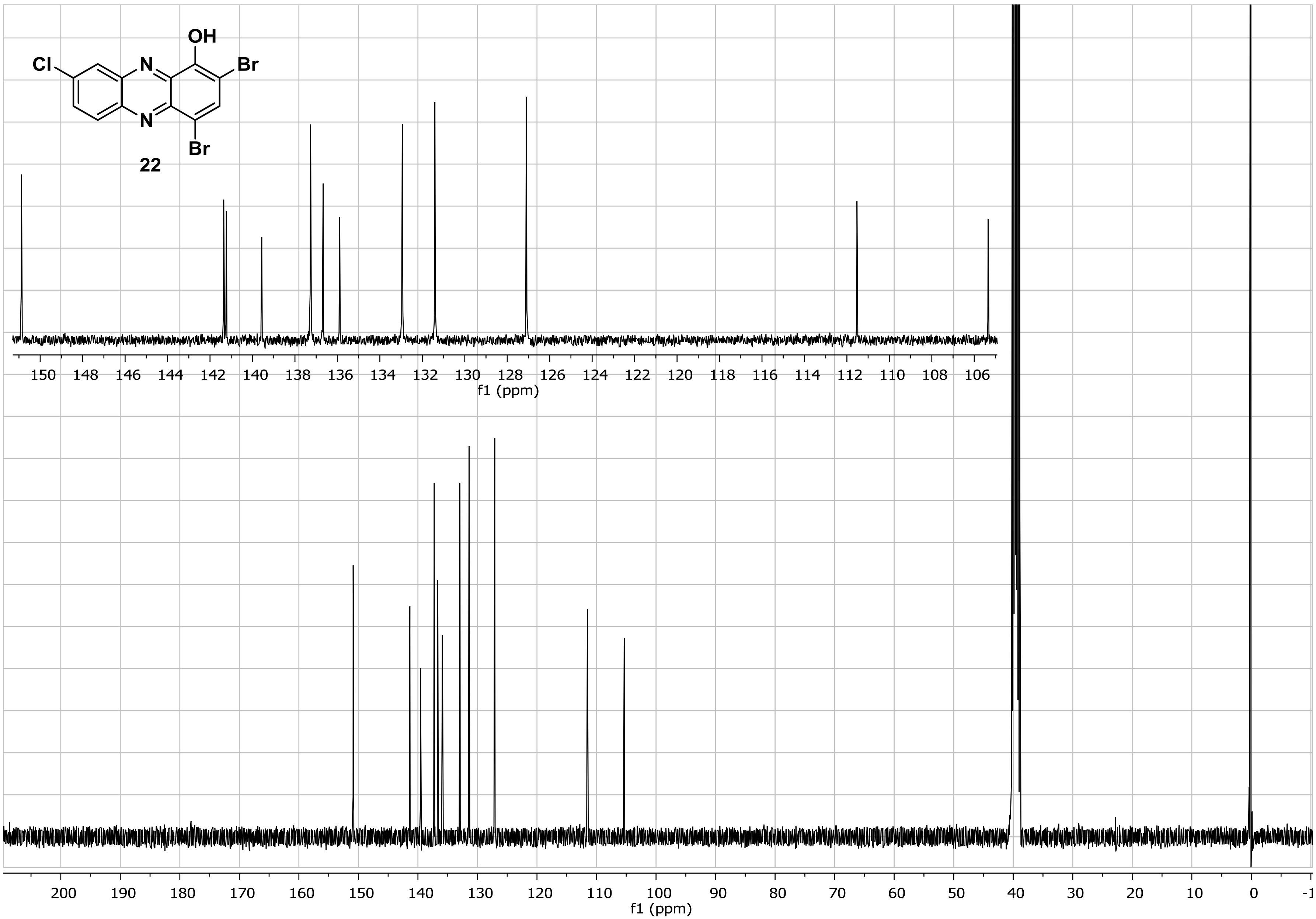
OH
Cl
N
N
Br
Br
22
150 148 146 144 142 140 138 136 134 132 130 128 126 124 122 120 118 116 114 112 110 108 106
f1 (ppm)
200 190 180 170 160 150 140 130 120 110 100 90 80 70 60 50 40 30 20 10 0 -1
f1 (ppm)

NMR-40

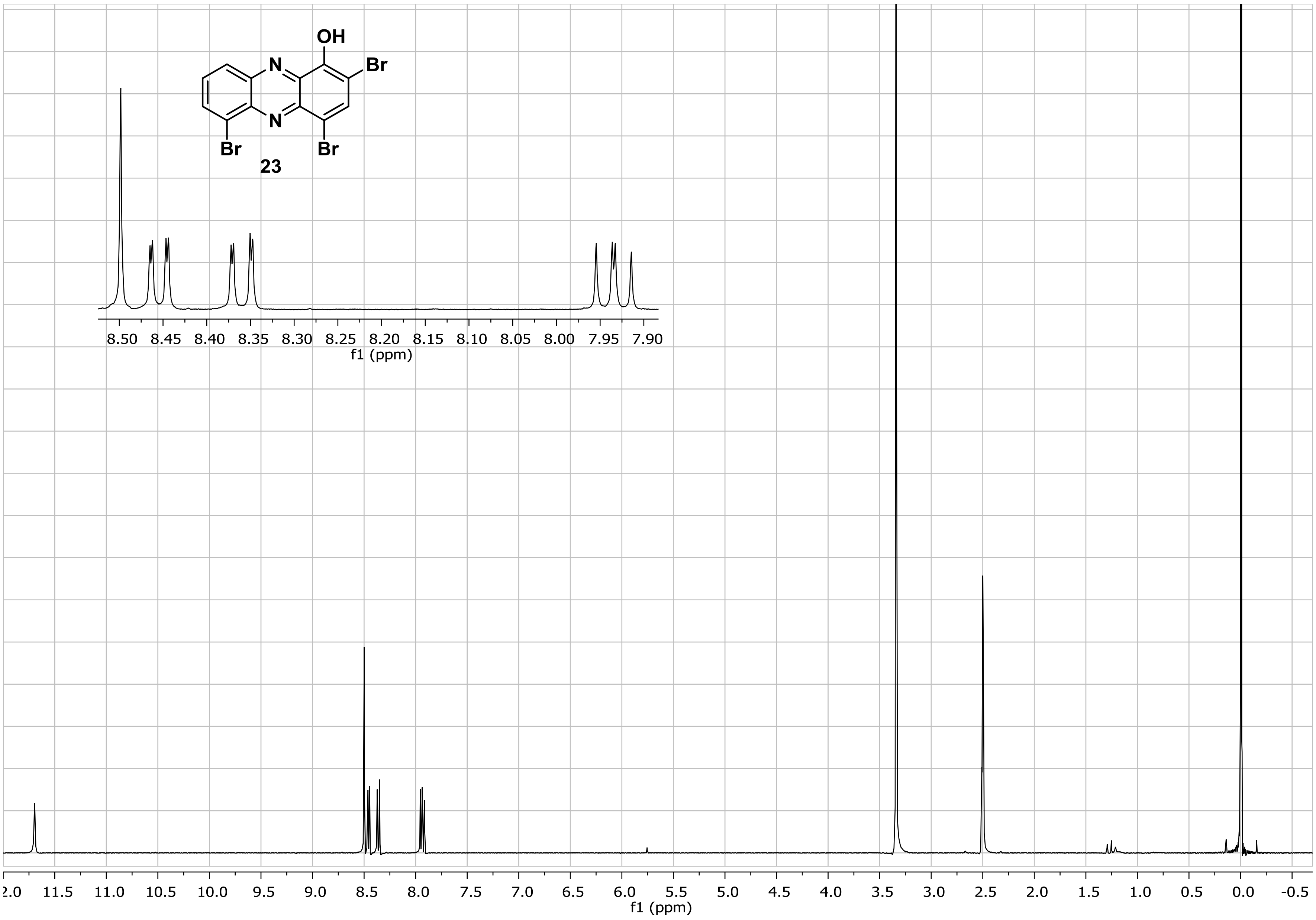
OH
Br
N
N
Br
Br
23
8.50 8.45 8.40 8.35 8.30 8.25 8.20 8.15 8.10 8.05 8.00 7.95 7.90
f1 (ppm)
2.0 11.5 11.0 10.5 10.0 9.5 9.0 8.5 8.0 7.5 7.0 6.5 6.0 5.5 5.0 4.5 4.0 3.5 3.0 2.5 2.0 1.5 1.0 0.5 0.0 -0.5
f1 (ppm)

NMR-41

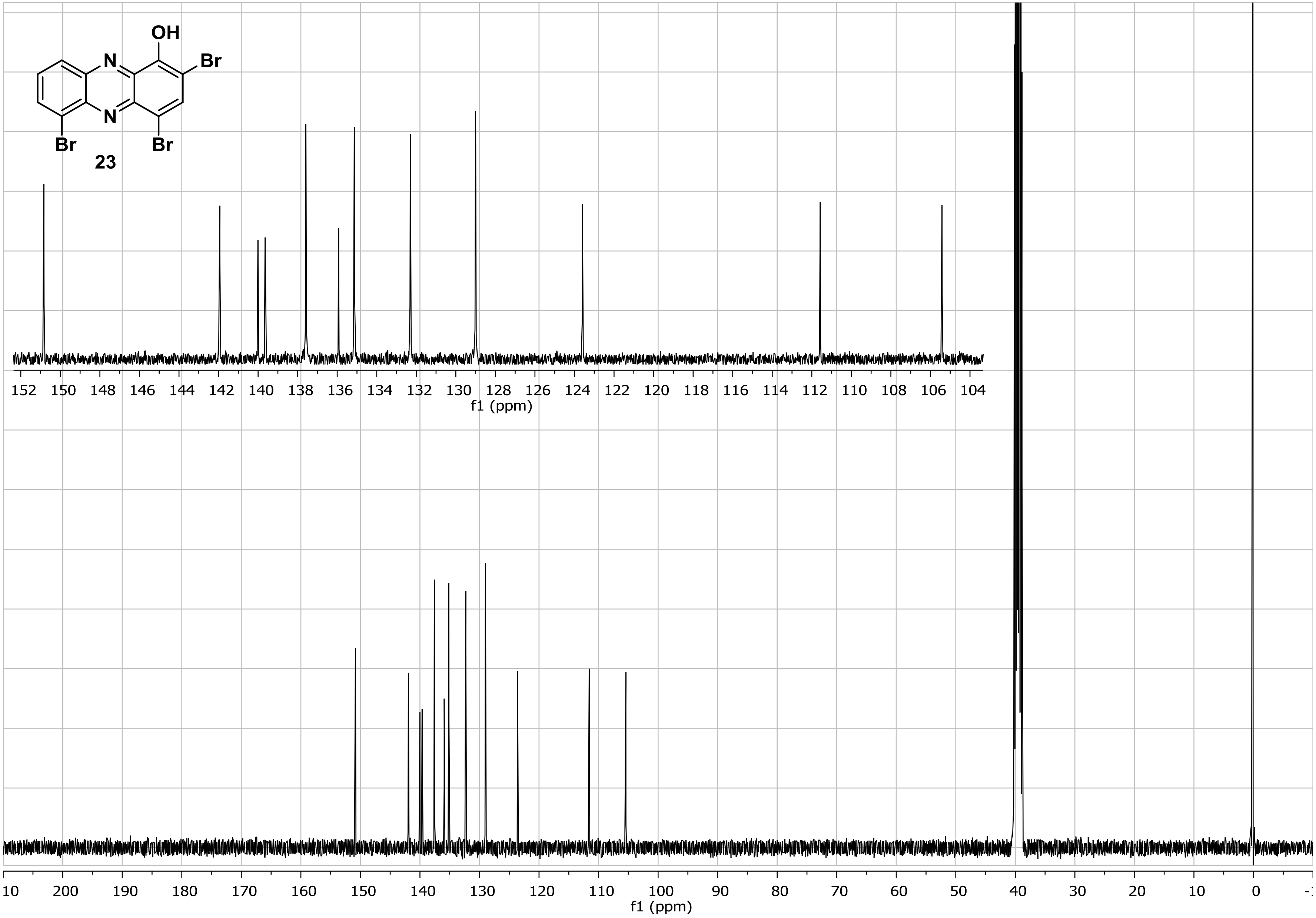
OH
Br
N
N
Br
Br
23
152 150 148 146 144 142 140 138 136 134 132 130 128 126 124 122 120 118 116 114 112 110 108 106 104
f1 (ppm)
200 190 180 170 160 150 140 130 120 110 100 90 80 70 60 50 40 30 20 10 0
f1 (ppm)

NMR-42

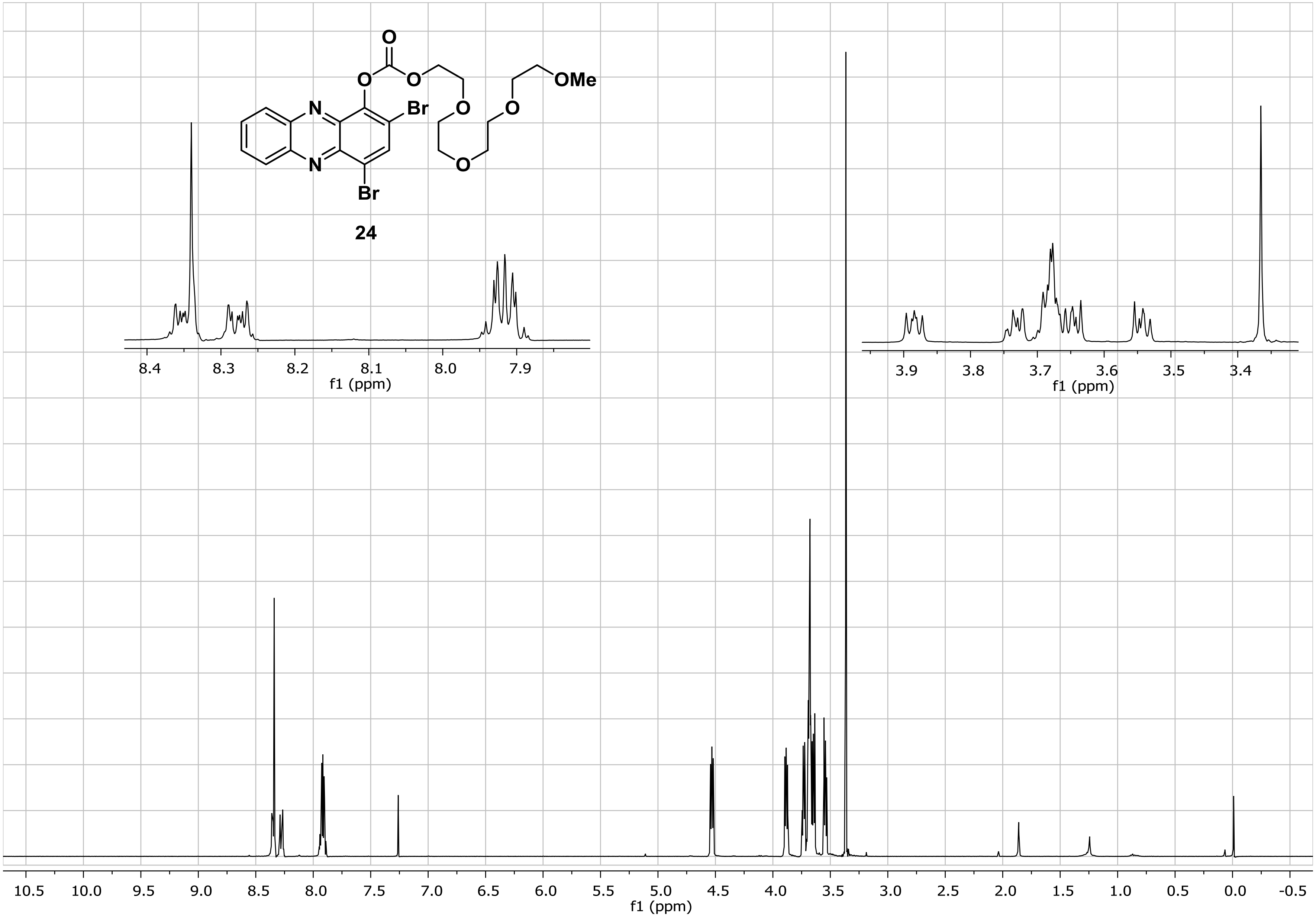

O
O
O
OMe
N
Br
O
O
N
O
Br
24
8.4
8.3
8.2
8.1
8.0
7.9
f1 (ppm)
3.9
3.8
3.7
3.6
3.5
3.4
f1 (ppm)
10.5
10.0
9.5
9.0
8.5
8.0
7.5
7.0
6.5
6.0
5.5
5.0
4.5
4.0
3.5
3.0
2.5
2.0
1.5
1.0
0.5
0.0
-0.5
f1 (ppm)

NMR-43

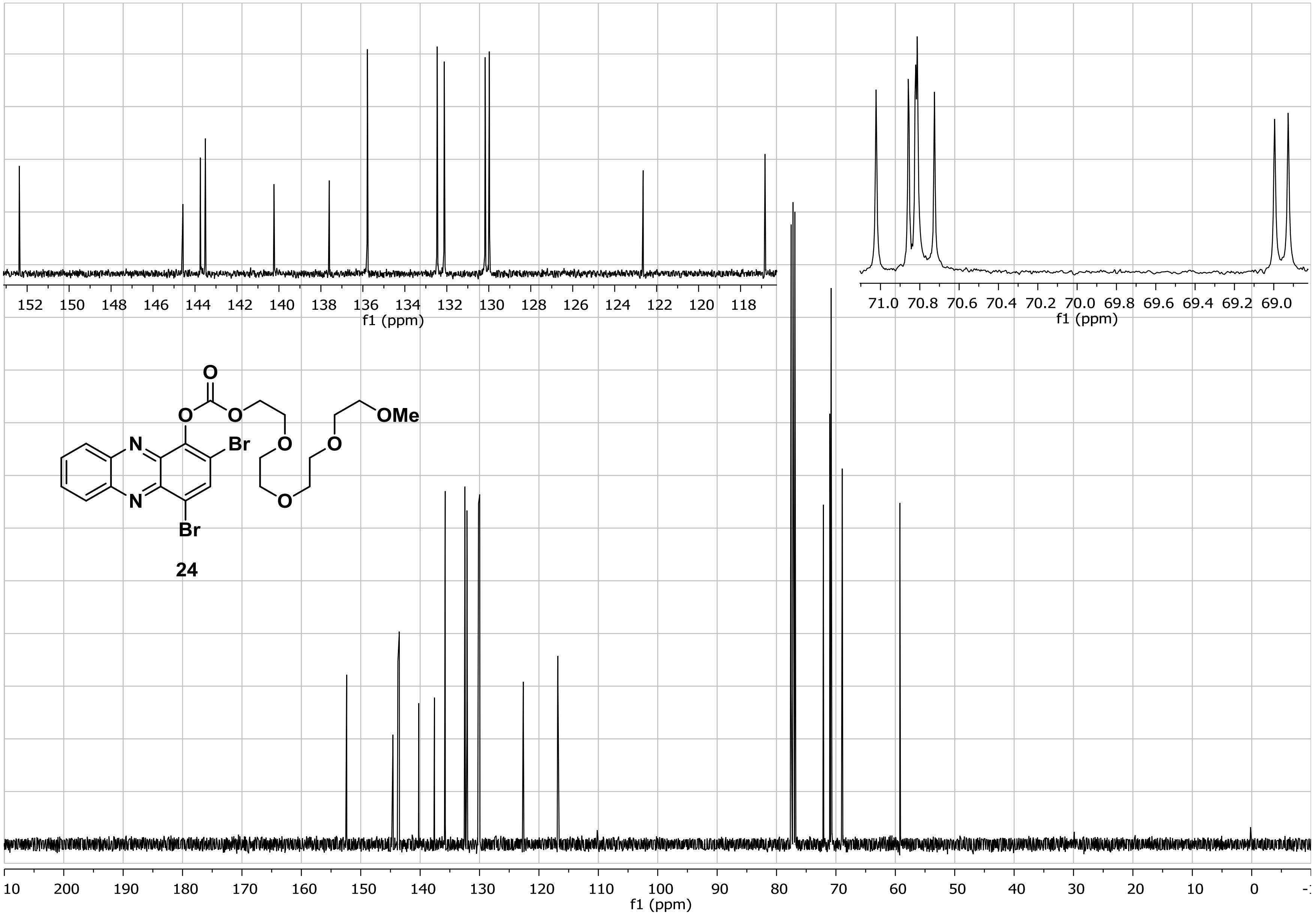

NMR-44

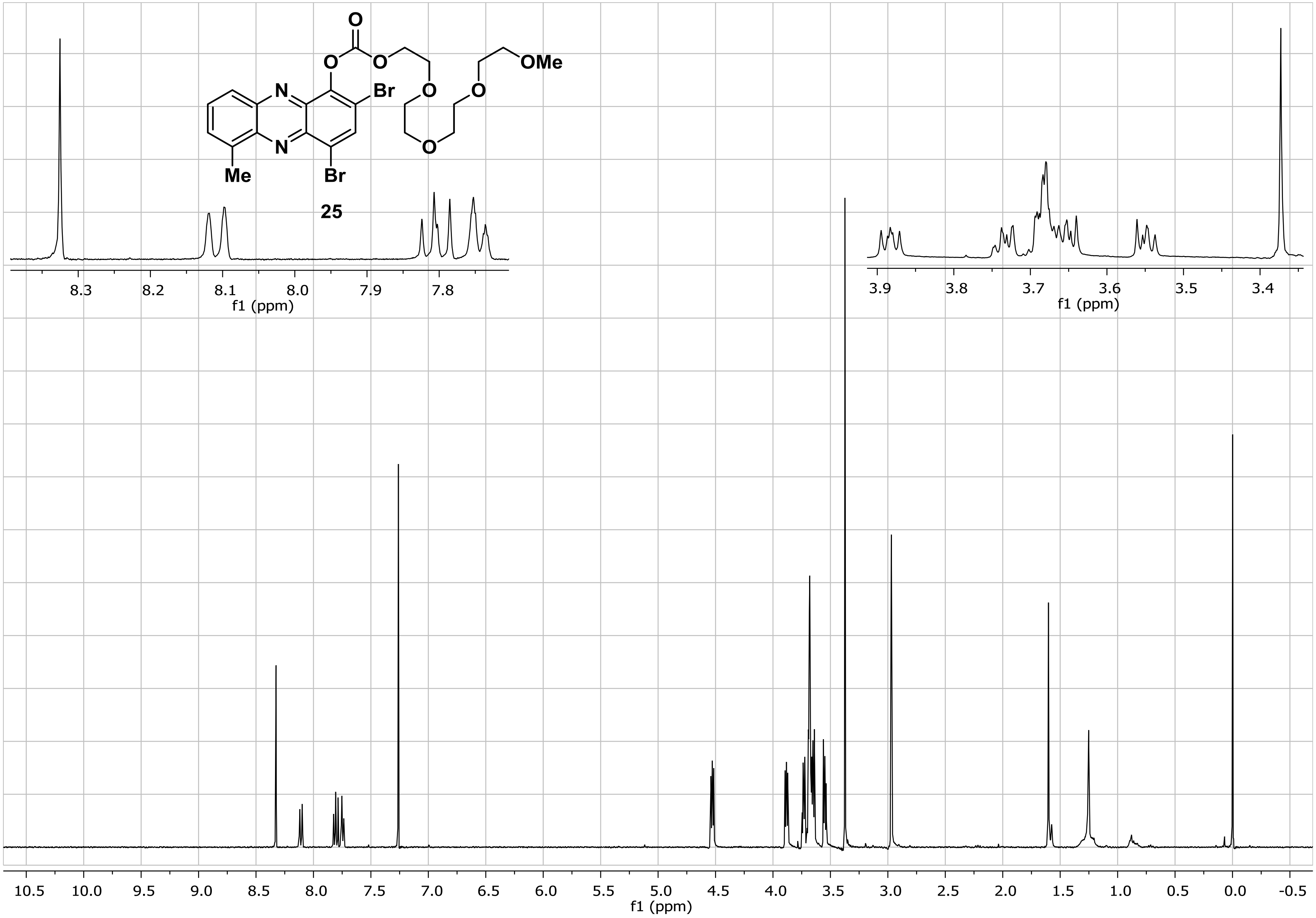

NMR-45

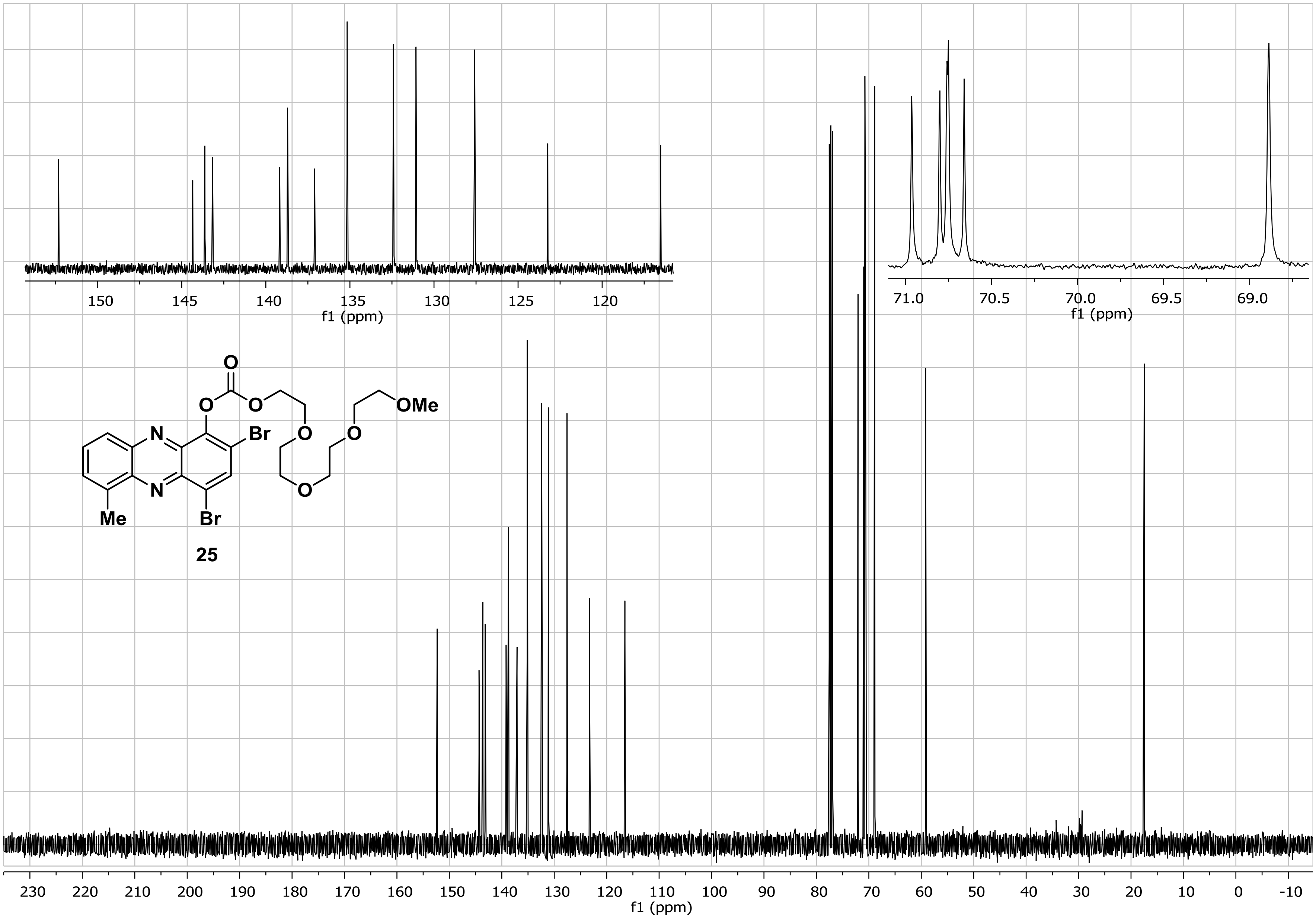

NMR-46

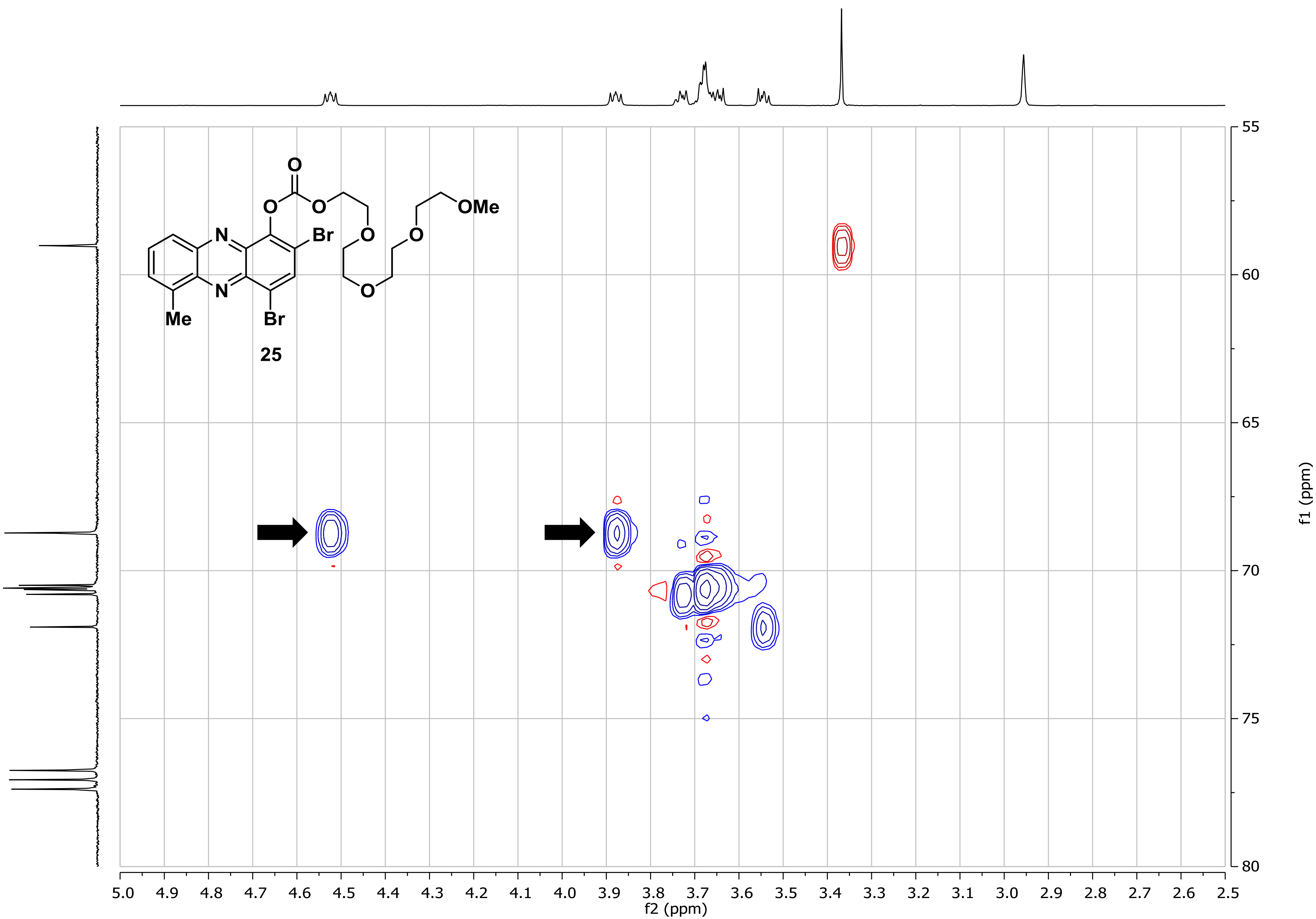

O
O
O
OMe
N
Br
O
O
N
O
Me
Br
25
55
60
65
70
75
80
f1 (ppm)
5.0 4.9 4.8 4.7 4.6 4.5 4.4 4.3 4.2 4.1 4.0 3.9 3.8 3.7 3.6 3.5 3.4 3.3 3.2 3.1 3.0 2.9 2.8 2.7 2.6 2.5
f2 (ppm)

NMR-47

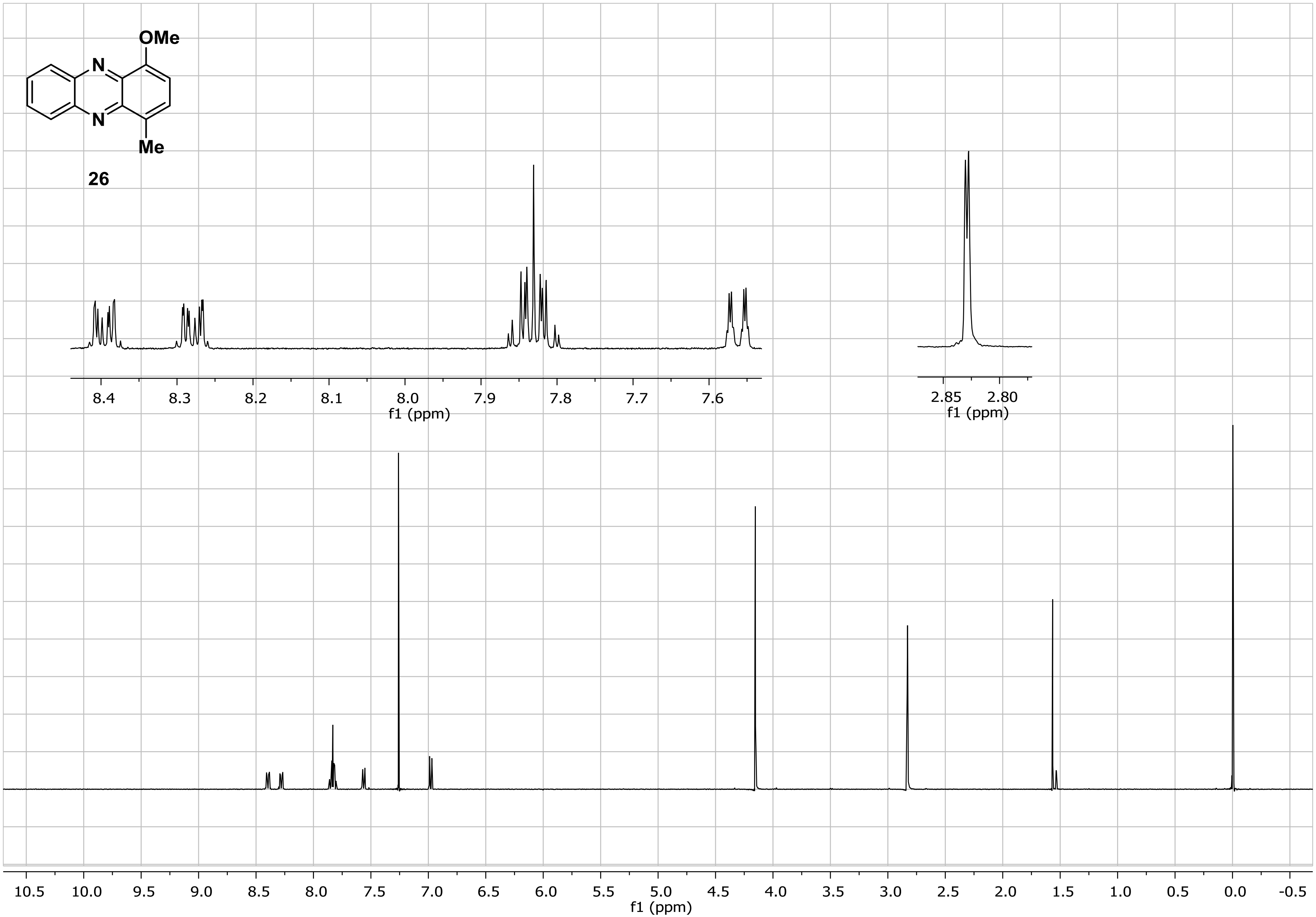
OMe
N
N
Me
26
8.4
8.3
8.2
8.1
8.0
7.9
7.8
7.7
7.6
f1 (ppm)
2.85
2.80
f1 (ppm)
10.5
10.0
9.5
9.0
8.5
8.0
7.5
7.0
6.5
6.0
5.5
5.0
4.5
4.0
3.5
3.0
2.5
2.0
1.5
1.0
0.5
0.0
-0.5
f1 (ppm)

NMR-48

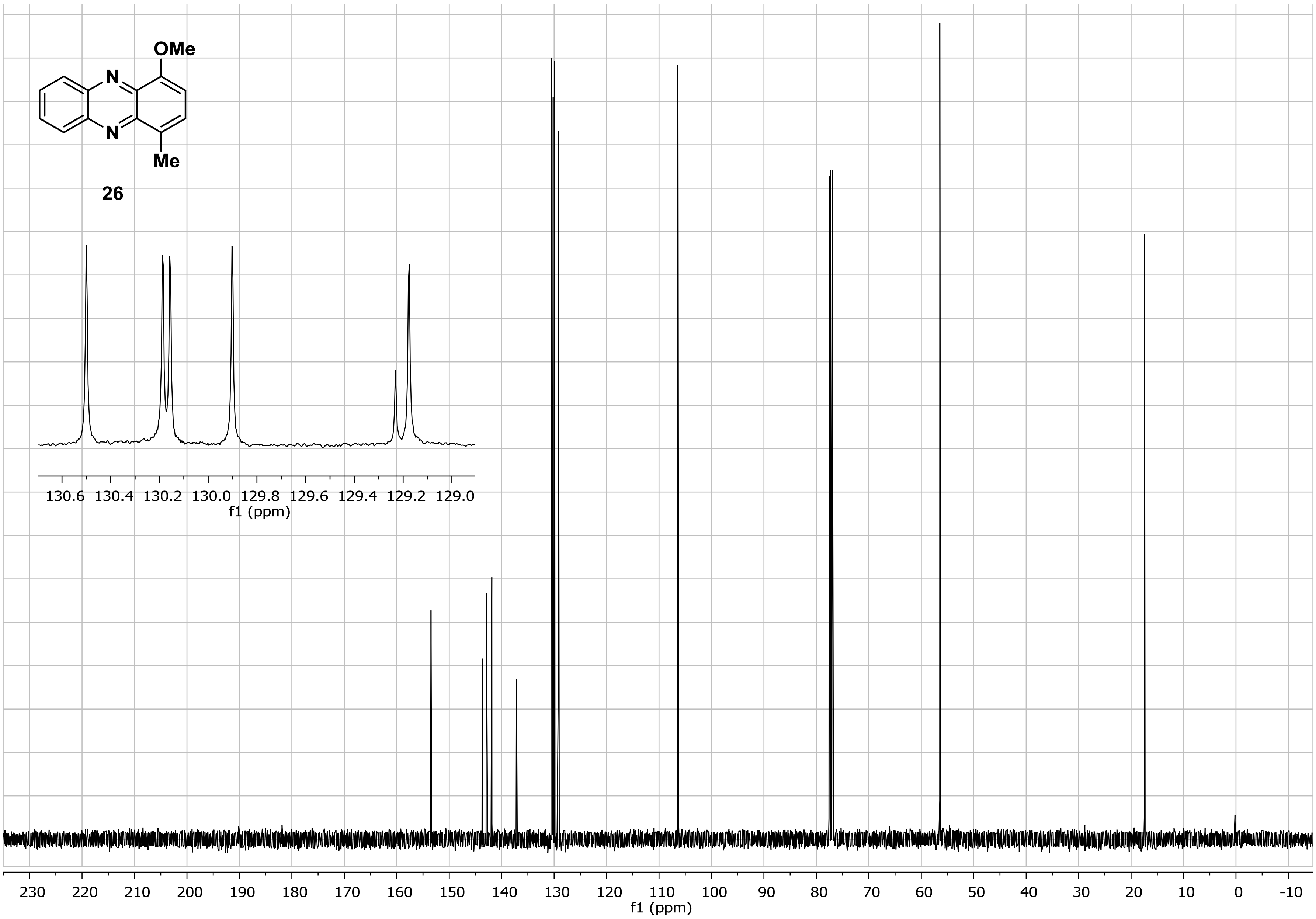
OMe
N
N
Me
26
130.6 130.4 130.2 130.0 129.8 129.6 129.4 129.2 129.0
f1 (ppm)
230 220 210 200 190 180 170 160 150 140 130 120 110 100 90 80 70 60 50 40 30 20 10 0 -10
f1 (ppm)

NMR-49

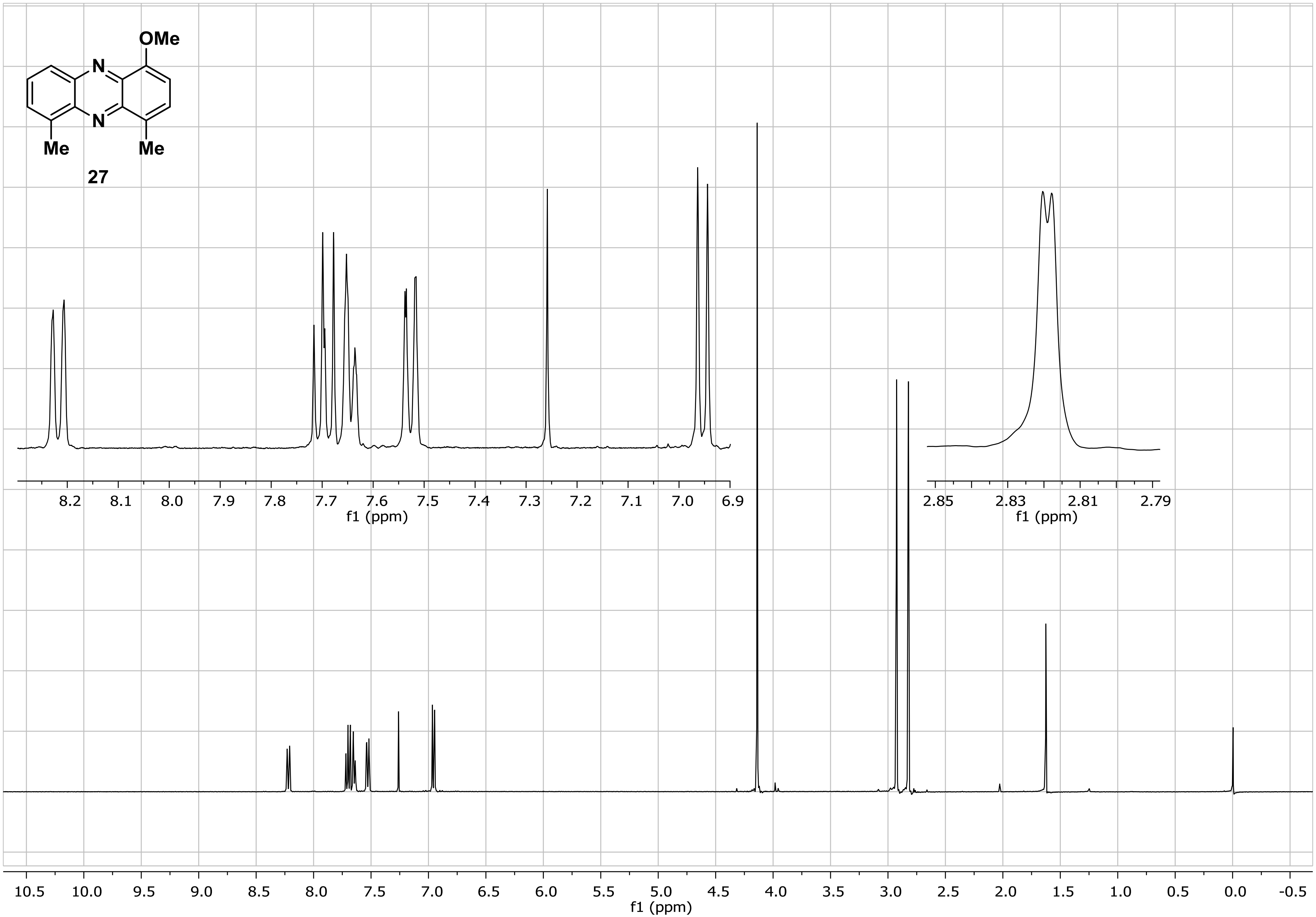
OMe
N
N
Me
Me
27
8.2
8.1
8.0
7.9
7.8
7.7
7.6
7.5
7.4
7.3
7.2
7.1
7.0
6.9
f1 (ppm)
2.85
2.83
2.81
2.79
f1 (ppm)
10.5
10.0
9.5
9.0
8.5
8.0
7.5
7.0
6.5
6.0
5.5
5.0
4.5
4.0
3.5
3.0
2.5
2.0
1.5
1.0
0.5
0.0
-0.5
f1 (ppm)

NMR-50

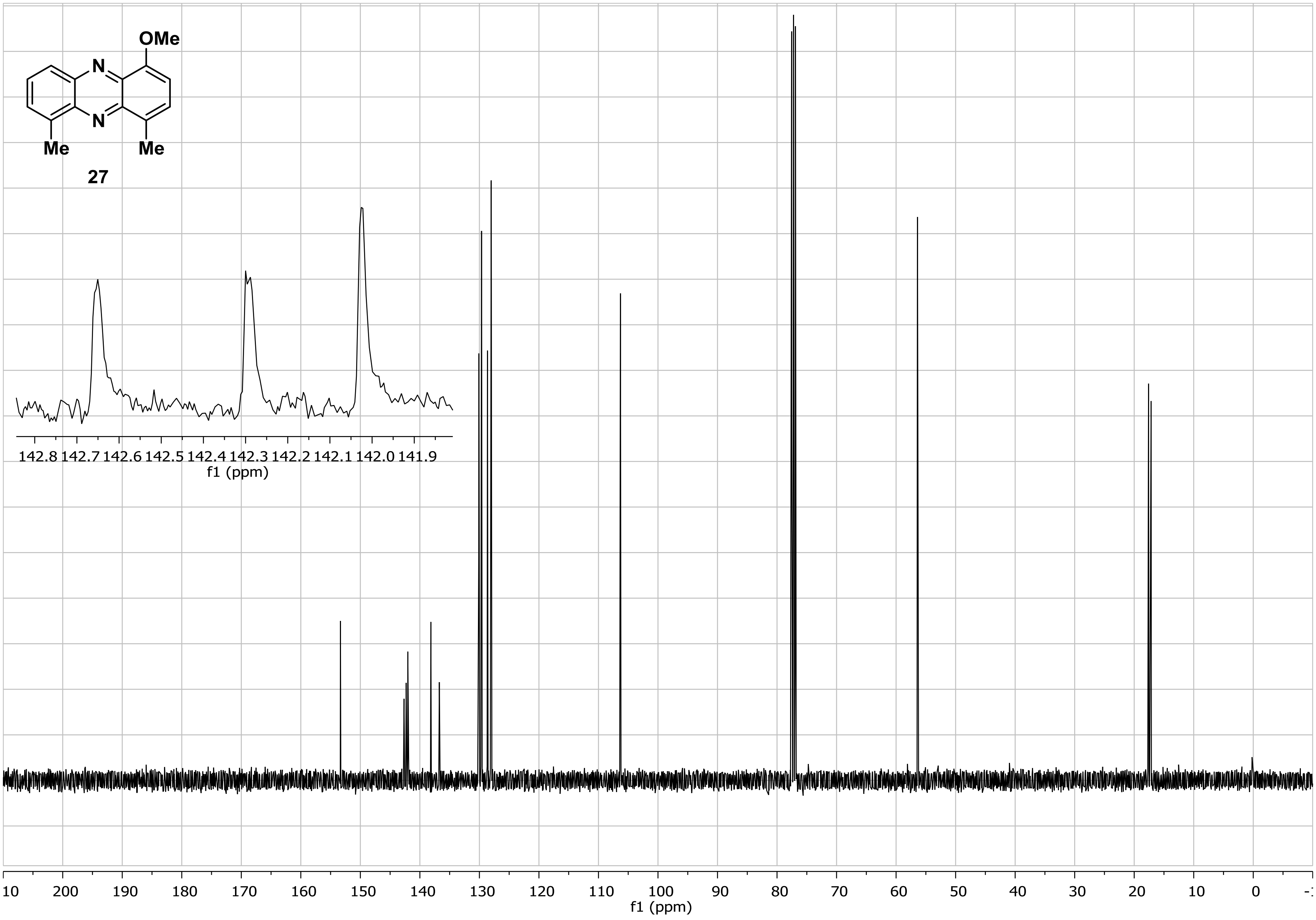

OMe
N
N
Me
Me
27
142.8 142.7 142.6 142.5 142.4 142.3 142.2 142.1 142.0 141.9
f1 (ppm)
10 200 190 180 170 160 150 140 130 120 110 100 90 80 70 60 50 40 30 20 10 0
f1 (ppm)

NMR-51

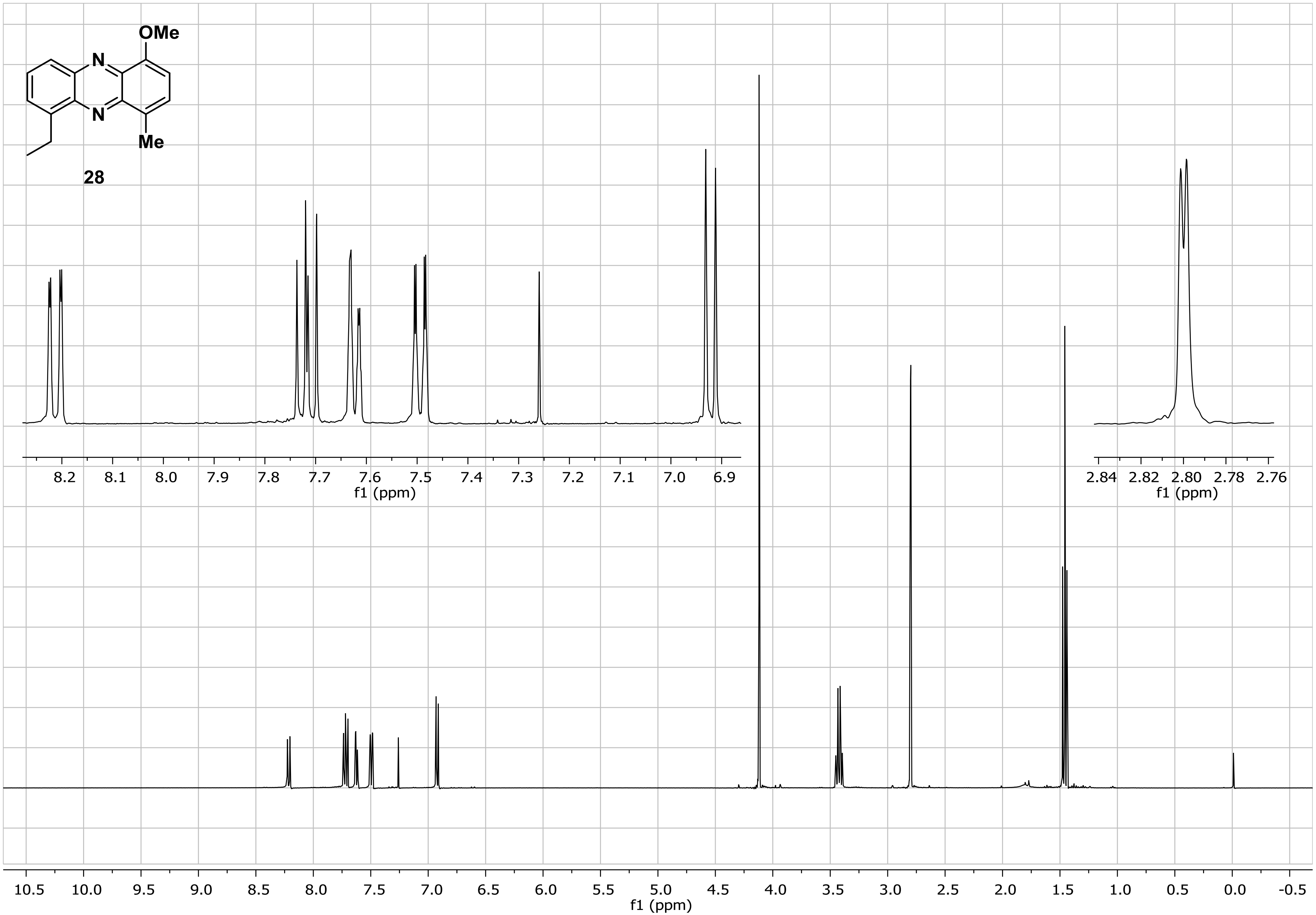

NMR-52

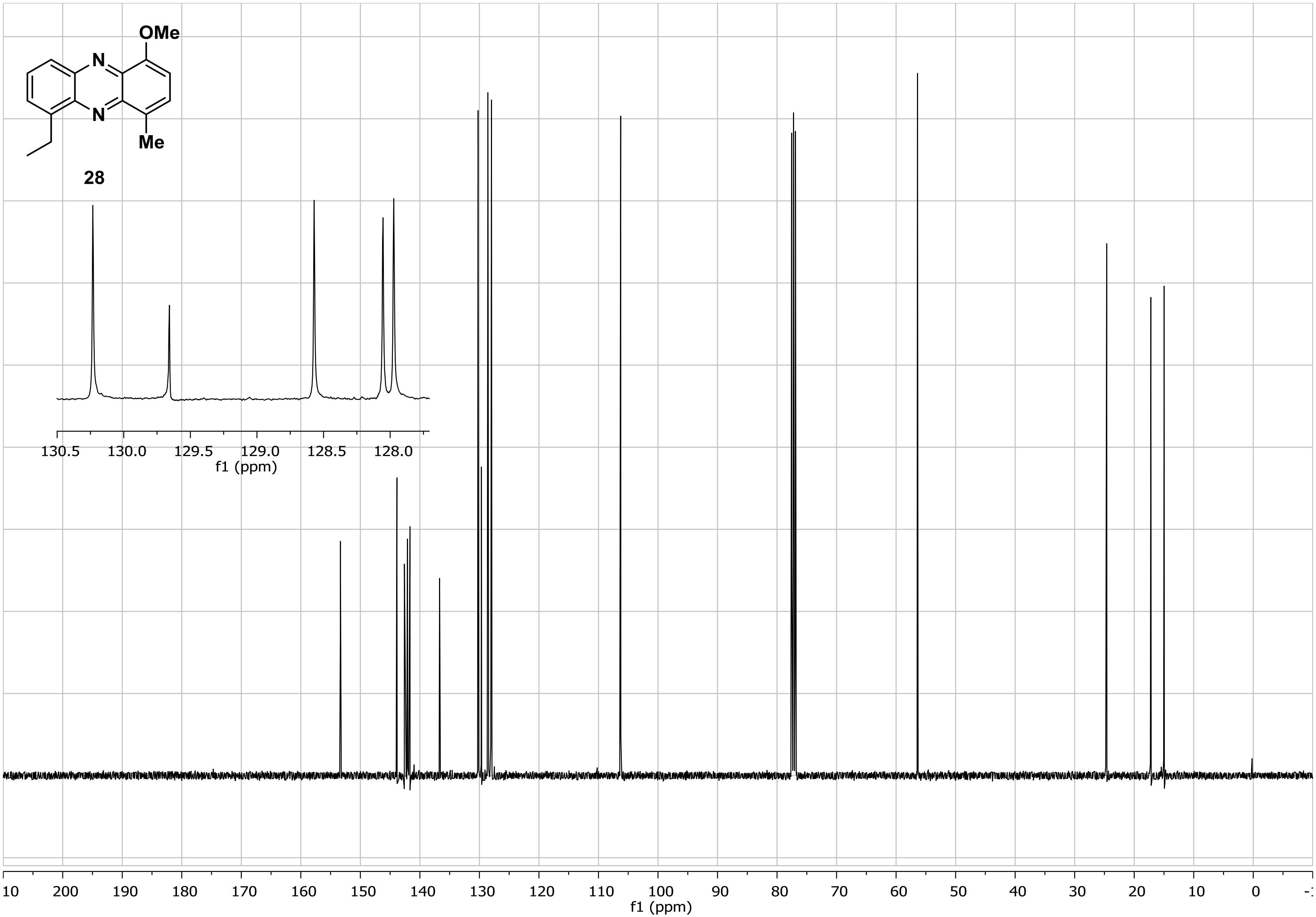

OMe
N
N
Me
28
130.5
130.0
129.5
129.0
128.5
128.0
f1 (ppm)
10
200
190
180
170
160
150
140
130
120
110
100
90
80
70
60
50
40
30
20
10
0
f1 (ppm)

NMR-53

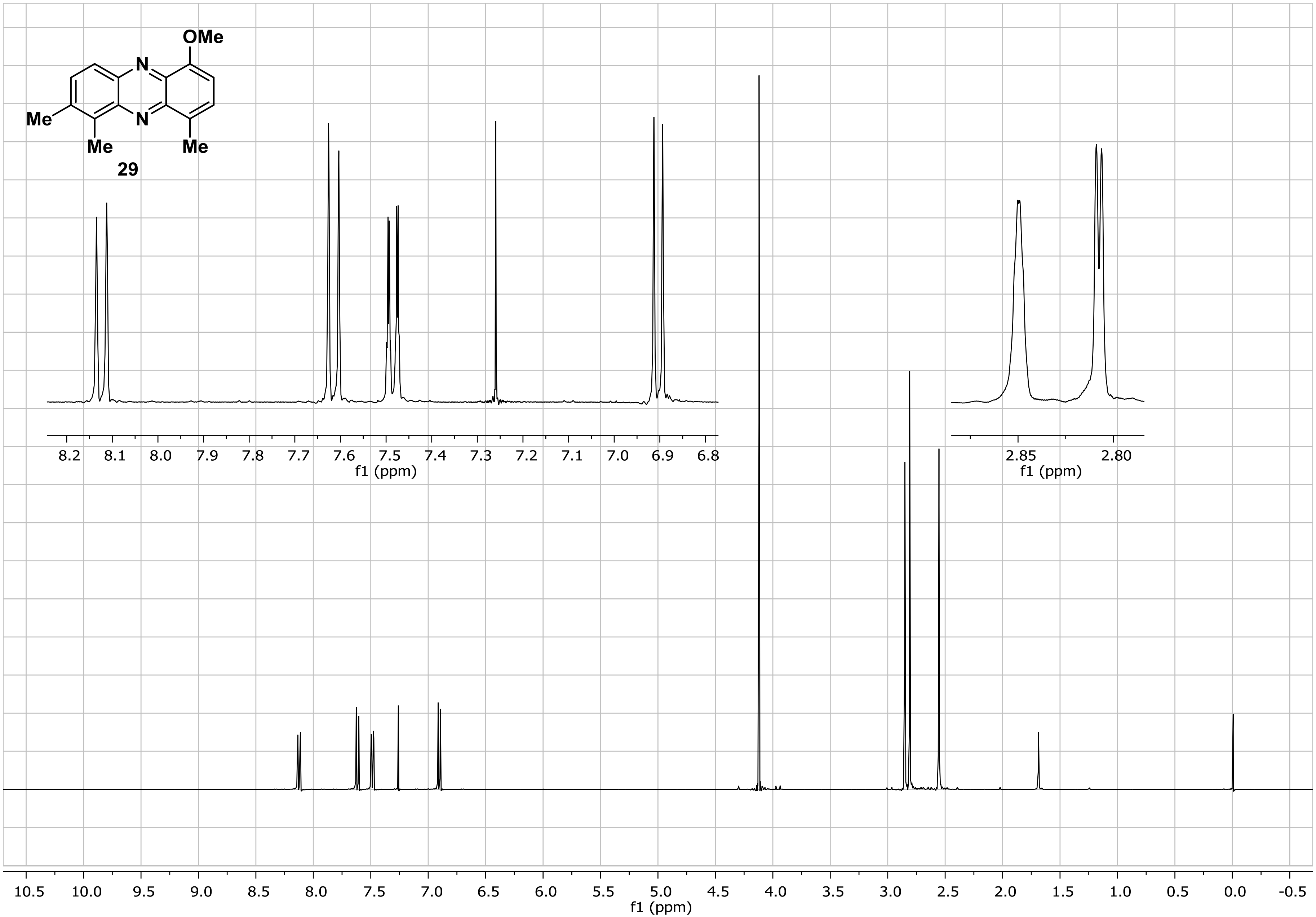
OMe
N
N
Me
Me
Me
29
8.2
8.1
8.0
7.9
7.8
7.7
7.6
7.5
7.4
7.3
7.2
7.1
7.0
6.9
6.8
f1 (ppm)
2.85
2.80
f1 (ppm)
10.5
10.0
9.5
9.0
8.5
8.0
7.5
7.0
6.5
6.0
5.5
5.0
4.5
4.0
3.5
3.0
2.5
2.0
1.5
1.0
0.5
0.0
-0.5
f1 (ppm)

NMR-54

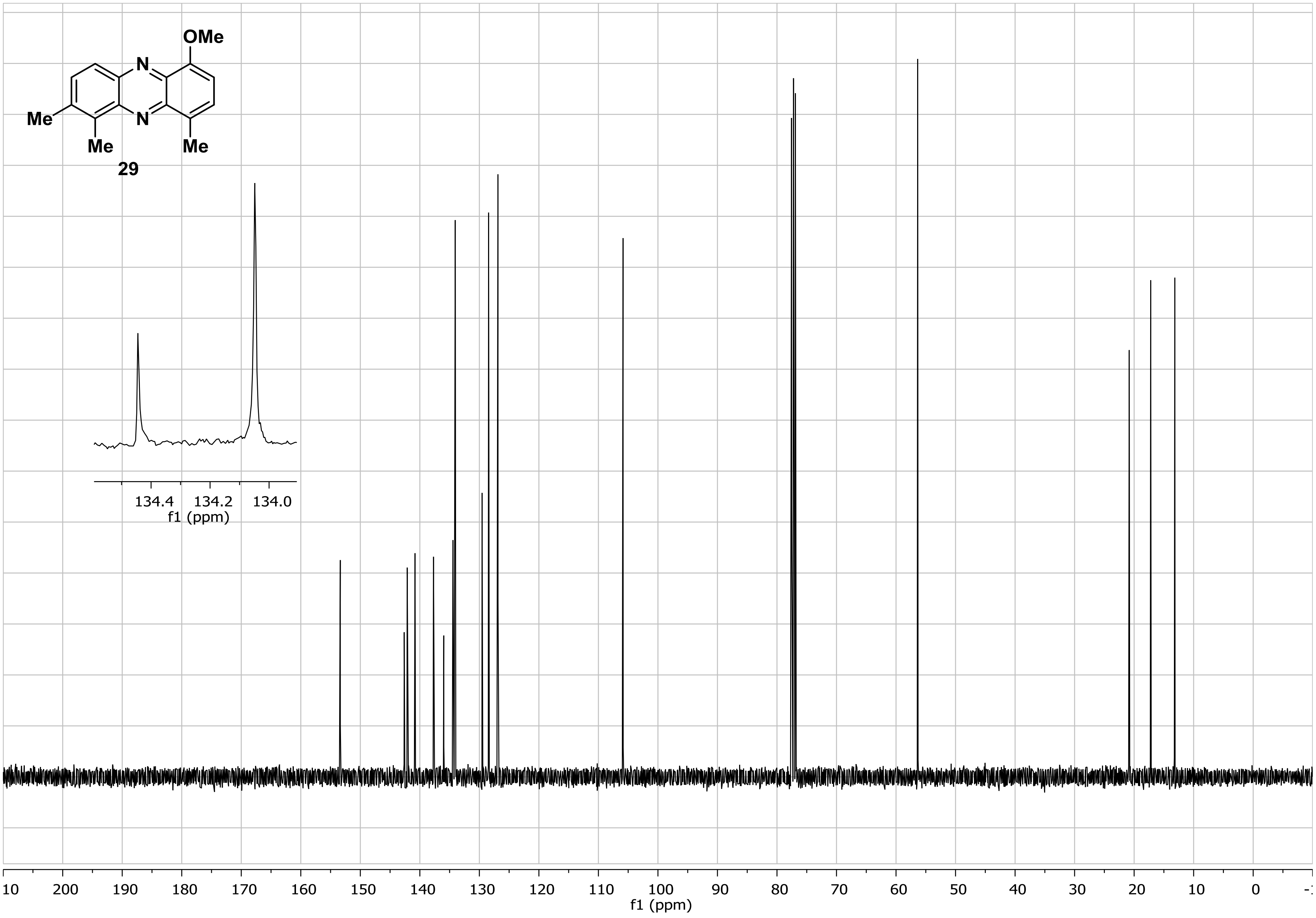
OMe
N
N
Me
Me
Me
29
134.4
134.2
134.0
f1 (ppm)
210
200
190
180
170
160
150
140
130
120
110
100
90
80
70
60
50
40
30
20
10
0
-10
f1 (ppm)

NMR-55

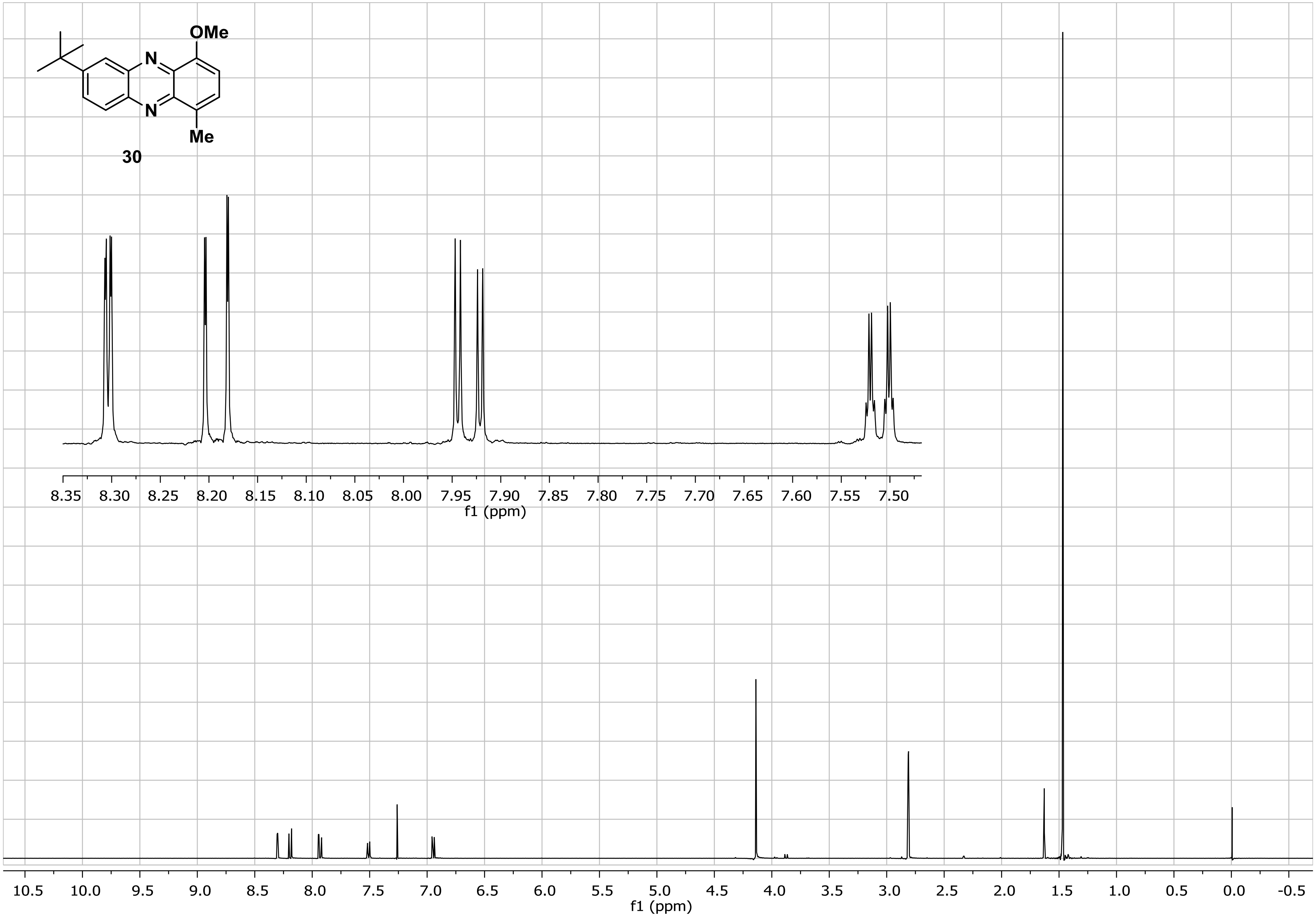

OMe
N
N
Me
30
8.35 8.30 8.25 8.20 8.15 8.10 8.05 8.00 7.95 7.90 7.85 7.80 7.75 7.70 7.65 7.60 7.55 7.50
f1 (ppm)
10.5 10.0 9.5 9.0 8.5 8.0 7.5 7.0 6.5 6.0 5.5 5.0 4.5 4.0 3.5 3.0 2.5 2.0 1.5 1.0 0.5 0.0 -0.5
f1 (ppm)

NMR-56

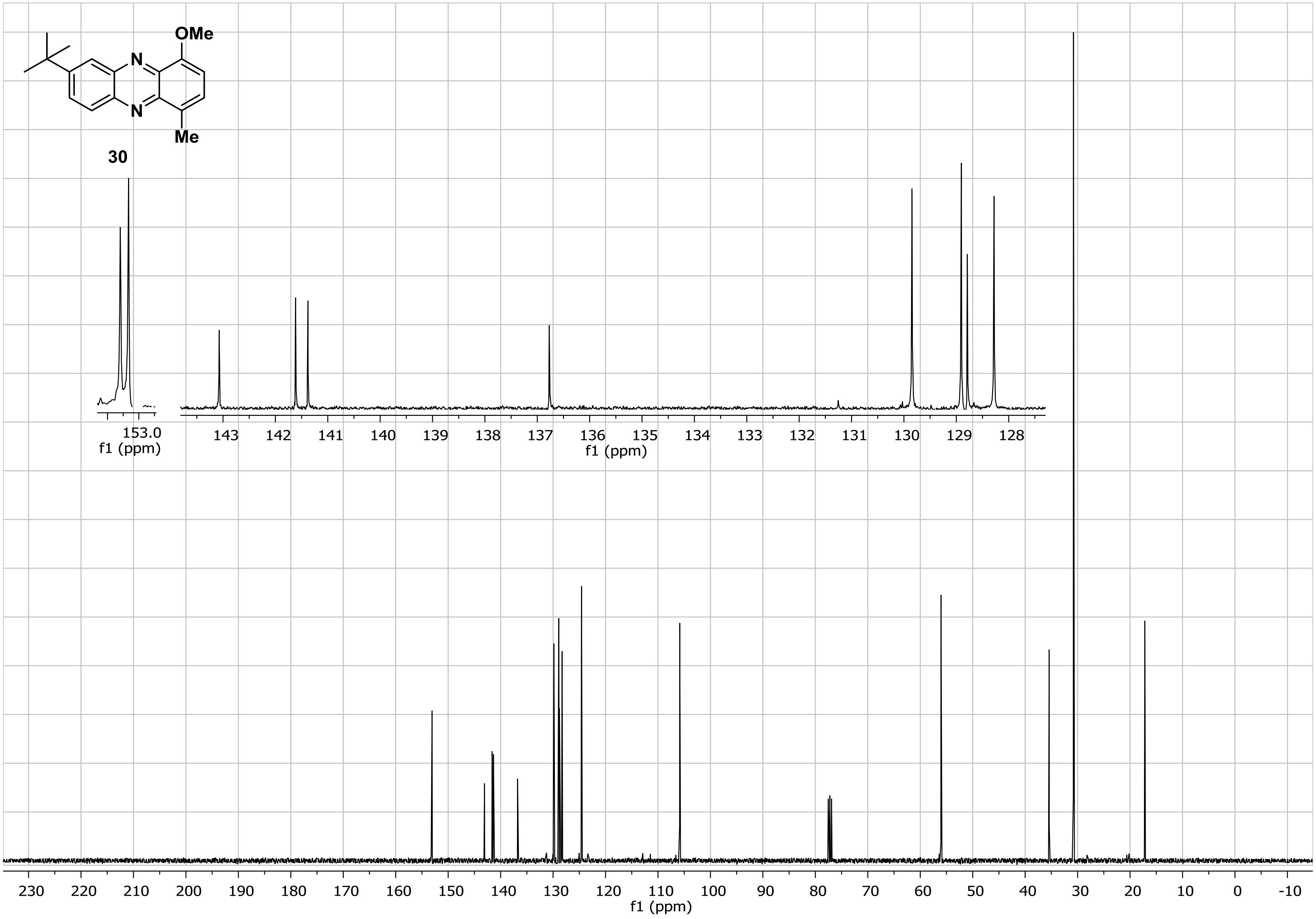
OMe
N
N
Me
30
153.0
f1 (ppm)
143 142 141 140 139 138 137 136 135 134 133 132 131 130 129 128
f1 (ppm)
230 220 210 200 190 180 170 160 150 140 130 120 110 100 90 80 70 60 50 40 30 20 10 0 -10
f1 (ppm)

NMR-57

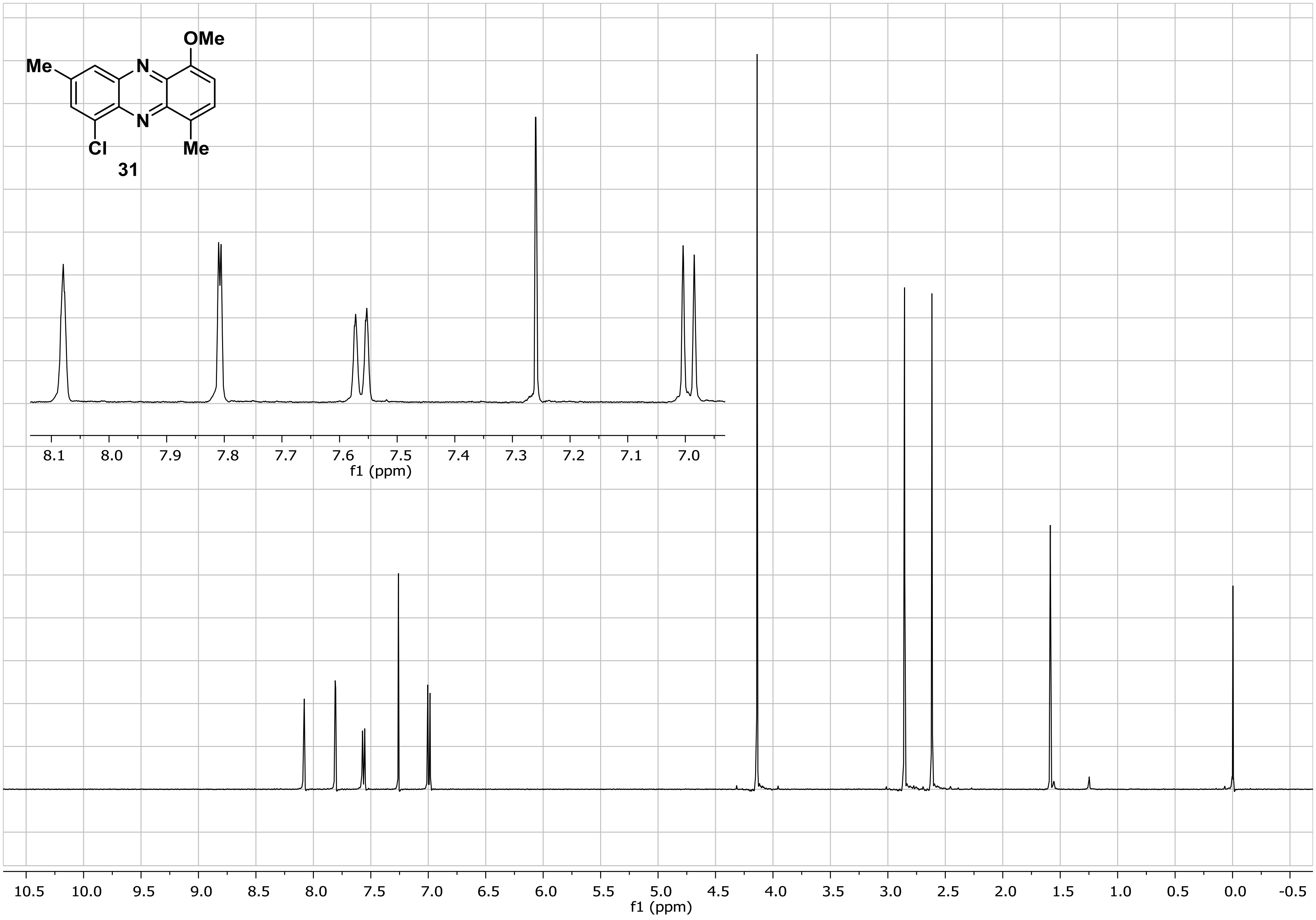
OMe
Me
N
N
Cl
Me
31
8.1 8.0 7.9 7.8 7.7 7.6 7.5 7.4 7.3 7.2 7.1 7.0
f1 (ppm)
10.5 10.0 9.5 9.0 8.5 8.0 7.5 7.0 6.5 6.0 5.5 5.0 4.5 4.0 3.5 3.0 2.5 2.0 1.5 1.0 0.5 0.0 -0.5
f1 (ppm)

NMR-58

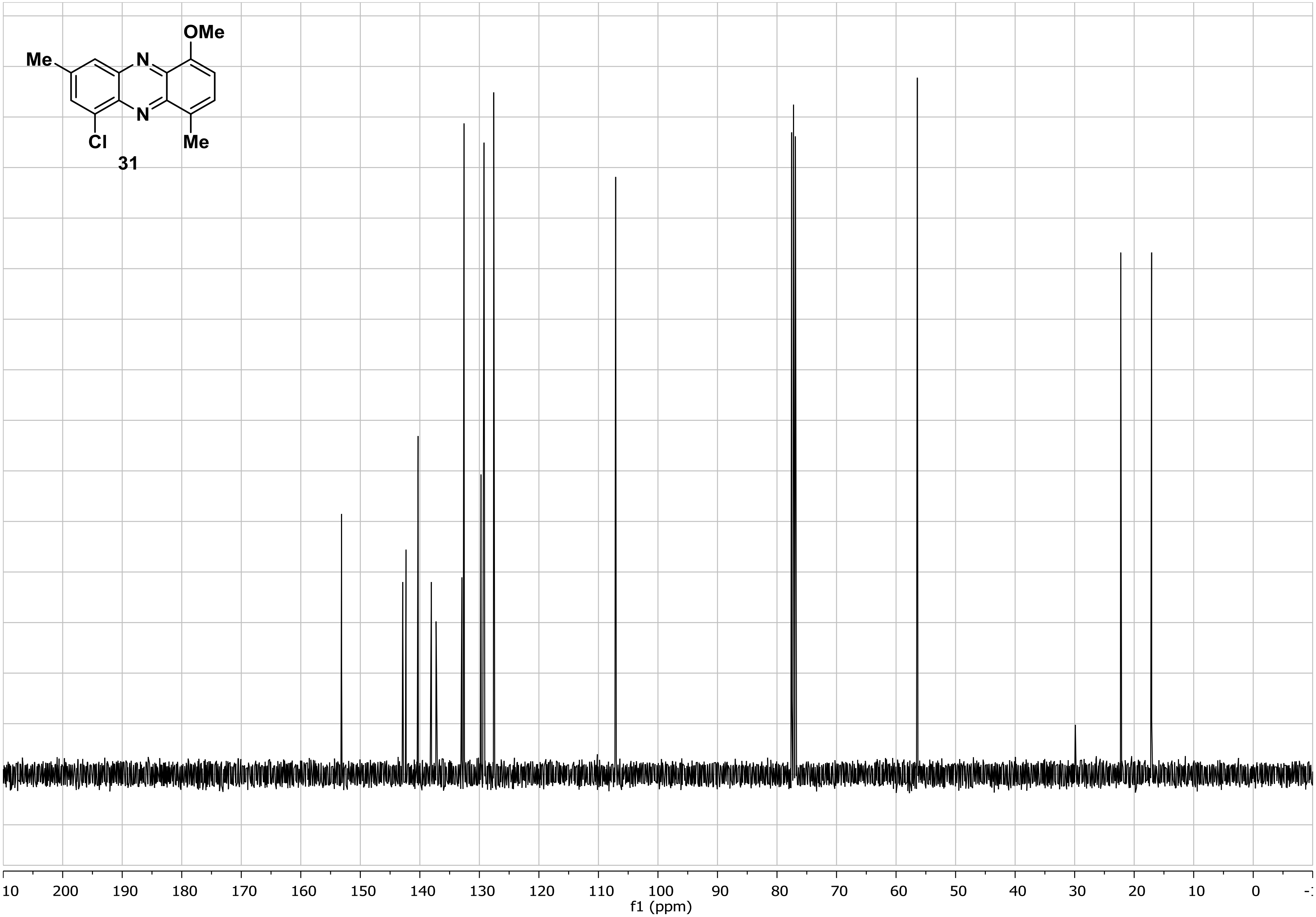
OMe
Me
N
N
Cl
Me
31
210
200
190
180
170
160
150
140
130
120
110
100
90
80
70
60
50
40
30
20
10
0
f1 (ppm)

NMR-59

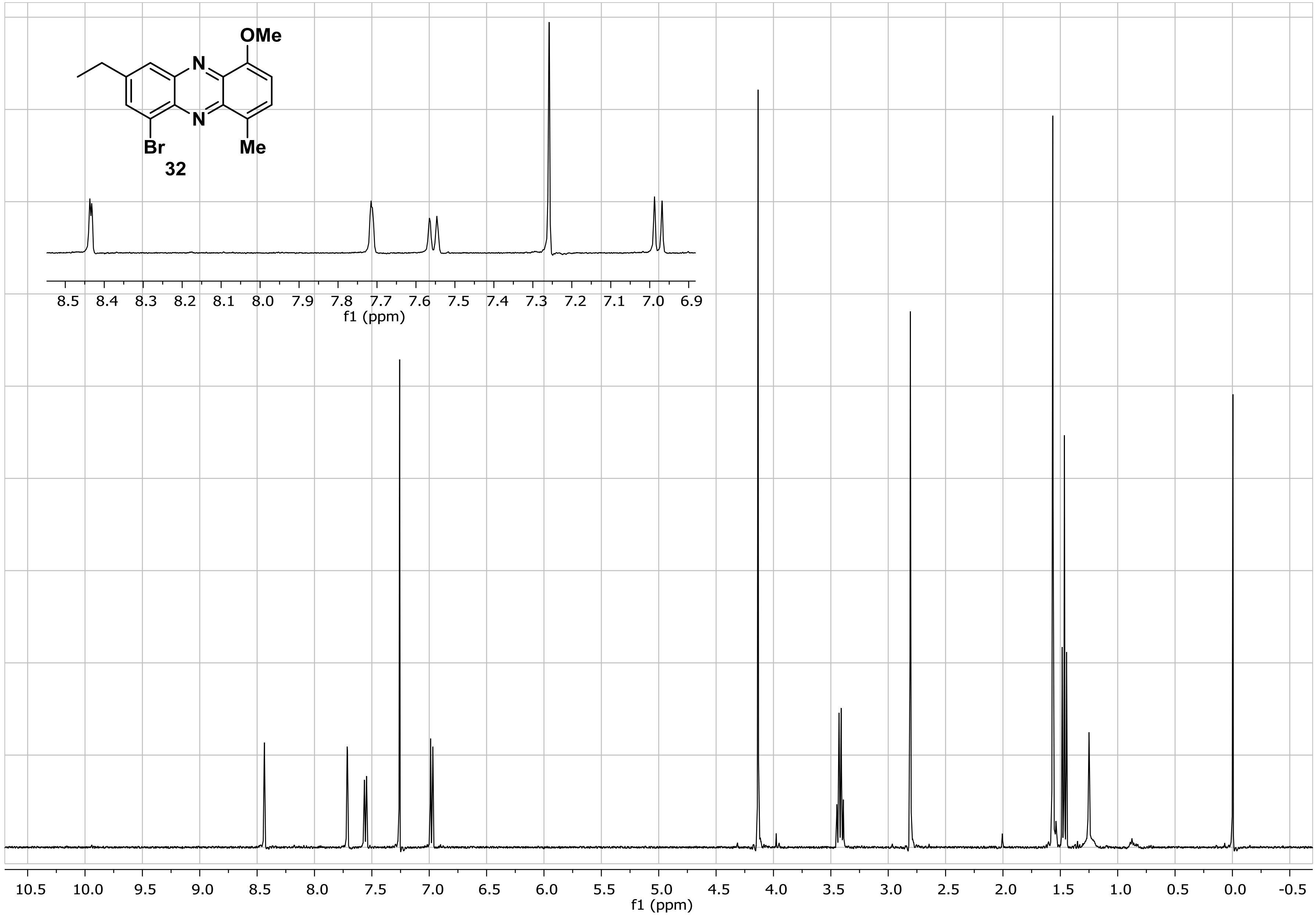
OMe
N
N
Br
Me
32
8.5 8.4 8.3 8.2 8.1 8.0 7.9 7.8 7.7 7.6 7.5 7.4 7.3 7.2 7.1 7.0 6.9
f1 (ppm)
10.5 10.0 9.5 9.0 8.5 8.0 7.5 7.0 6.5 6.0 5.5 5.0 4.5 4.0 3.5 3.0 2.5 2.0 1.5 1.0 0.5 0.0 -0.5
f1 (ppm)

NMR-60

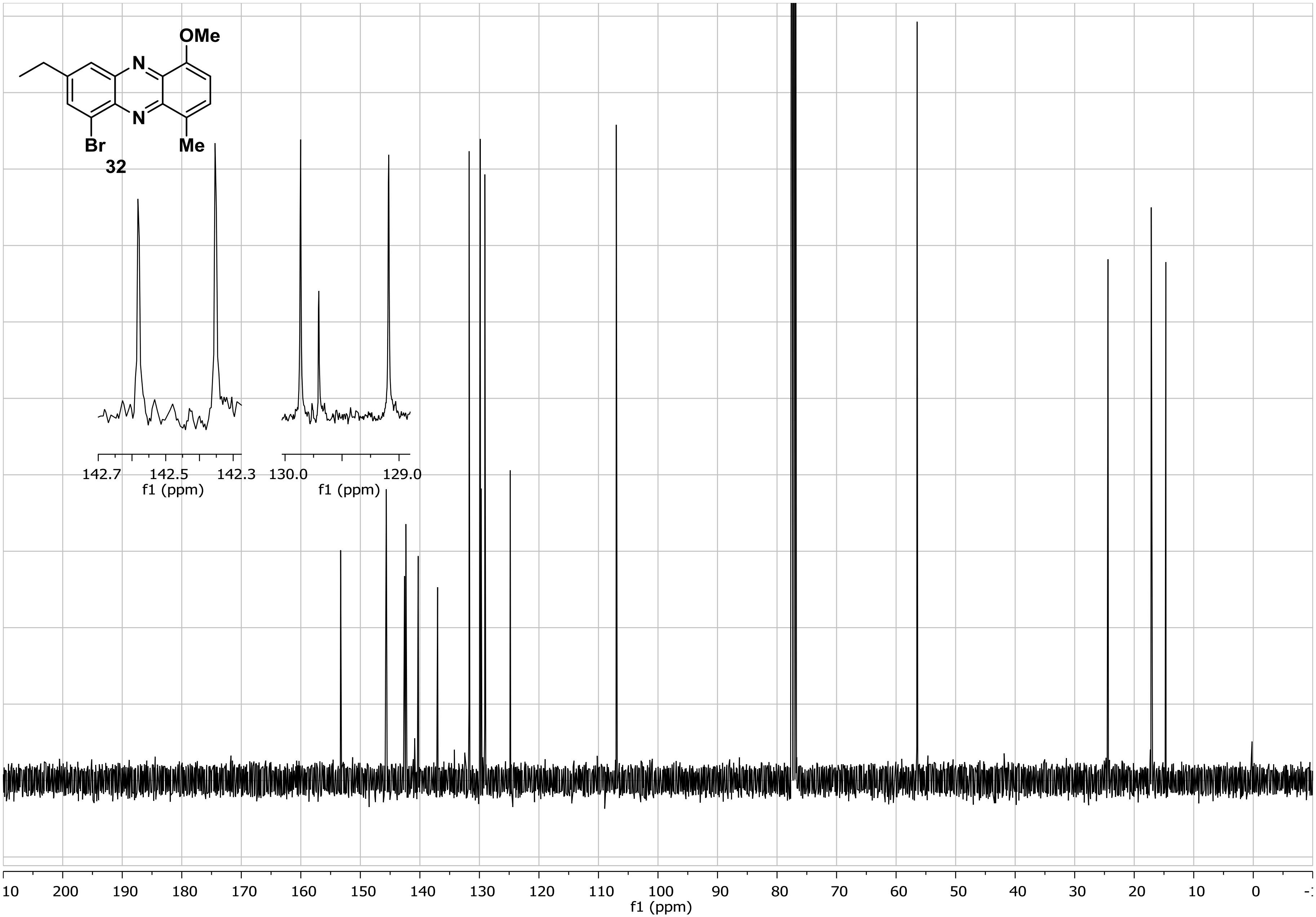
OMe
N
N
Br
Me
32
142.7
142.5
142.3
f1 (ppm)
130.0
129.0
f1 (ppm)
10
200
190
180
170
160
150
140
130
120
110
100
90
80
70
60
50
40
30
20
10
0
f1 (ppm)

NMR-61

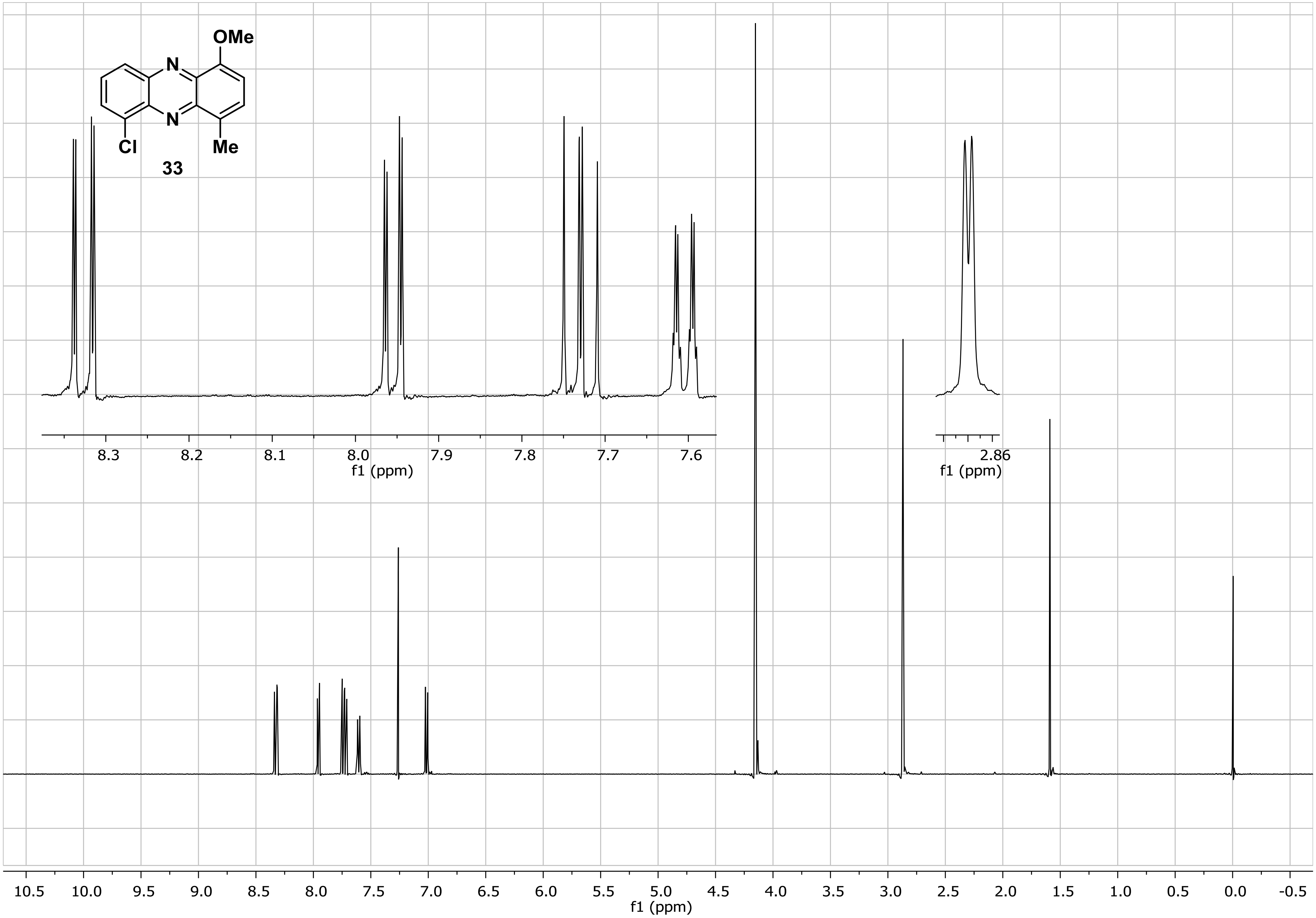
OMe
N
N
Cl
Me
33
8.3
8.2
8.1
8.0
7.9
7.8
7.7
7.6
f1 (ppm)
2.86
f1 (ppm)
10.5
10.0
9.5
9.0
8.5
8.0
7.5
7.0
6.5
6.0
5.5
5.0
4.5
4.0
3.5
3.0
2.5
2.0
1.5
1.0
0.5
0.0
-0.5
f1 (ppm)

NMR-62

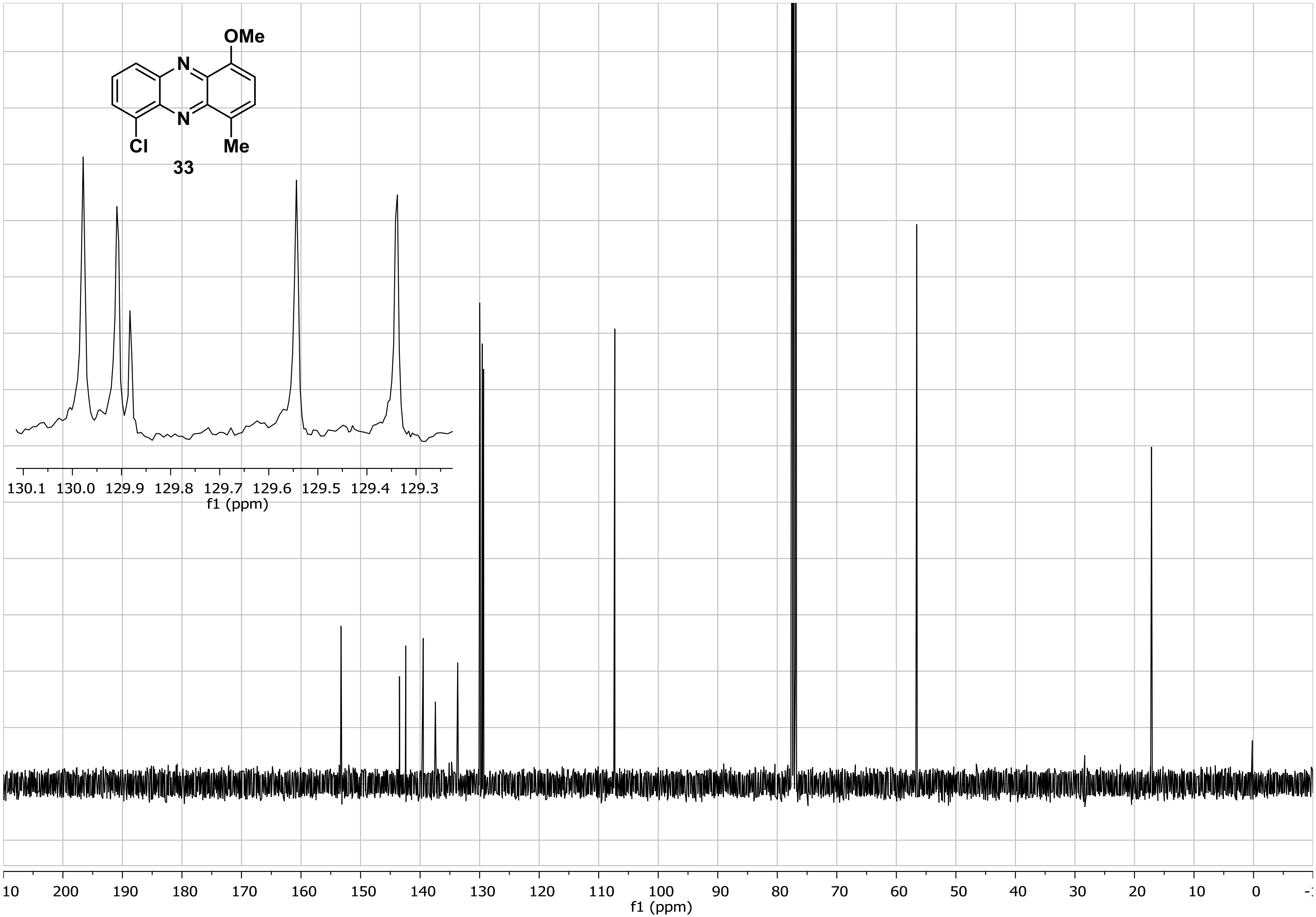
OMe
N
N
Cl
Me
33
130.1 130.0 129.9 129.8 129.7 129.6 129.5 129.4 129.3
f1 (ppm)
10 200 190 180 170 160 150 140 130 120 110 100 90 80 70 60 50 40 30 20 10 0
f1 (ppm)

NMR-63

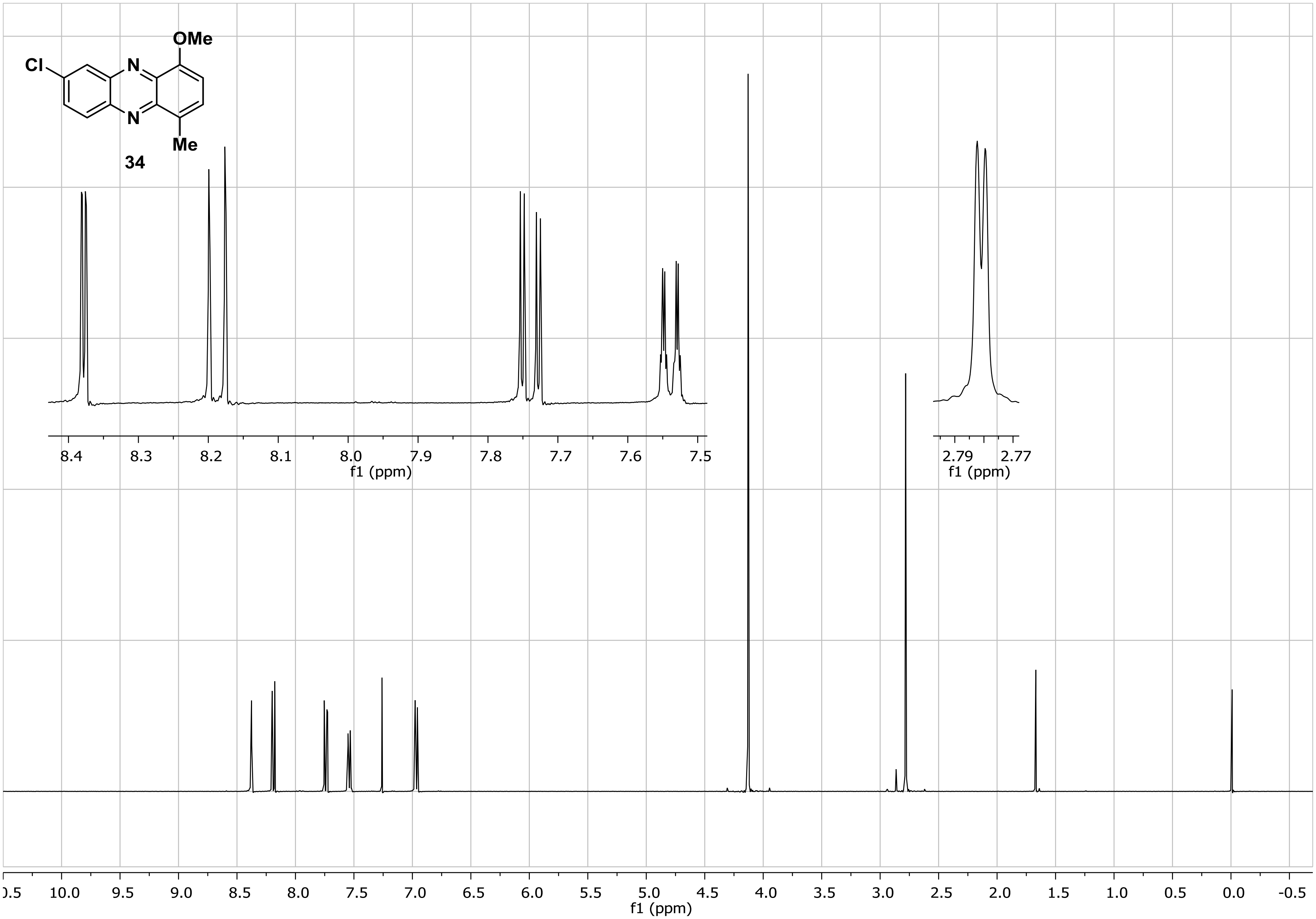
OMe
Cl
N
N
Me
34
8.4 8.3 8.2 8.1 8.0 7.9 7.8 7.7 7.6 7.5
f1 (ppm)
2.79 2.77
f1 (ppm)
0.5 10.0 9.5 9.0 8.5 8.0 7.5 7.0 6.5 6.0 5.5 5.0 4.5 4.0 3.5 3.0 2.5 2.0 1.5 1.0 0.5 0.0 -0.5
f1 (ppm)

NMR-64

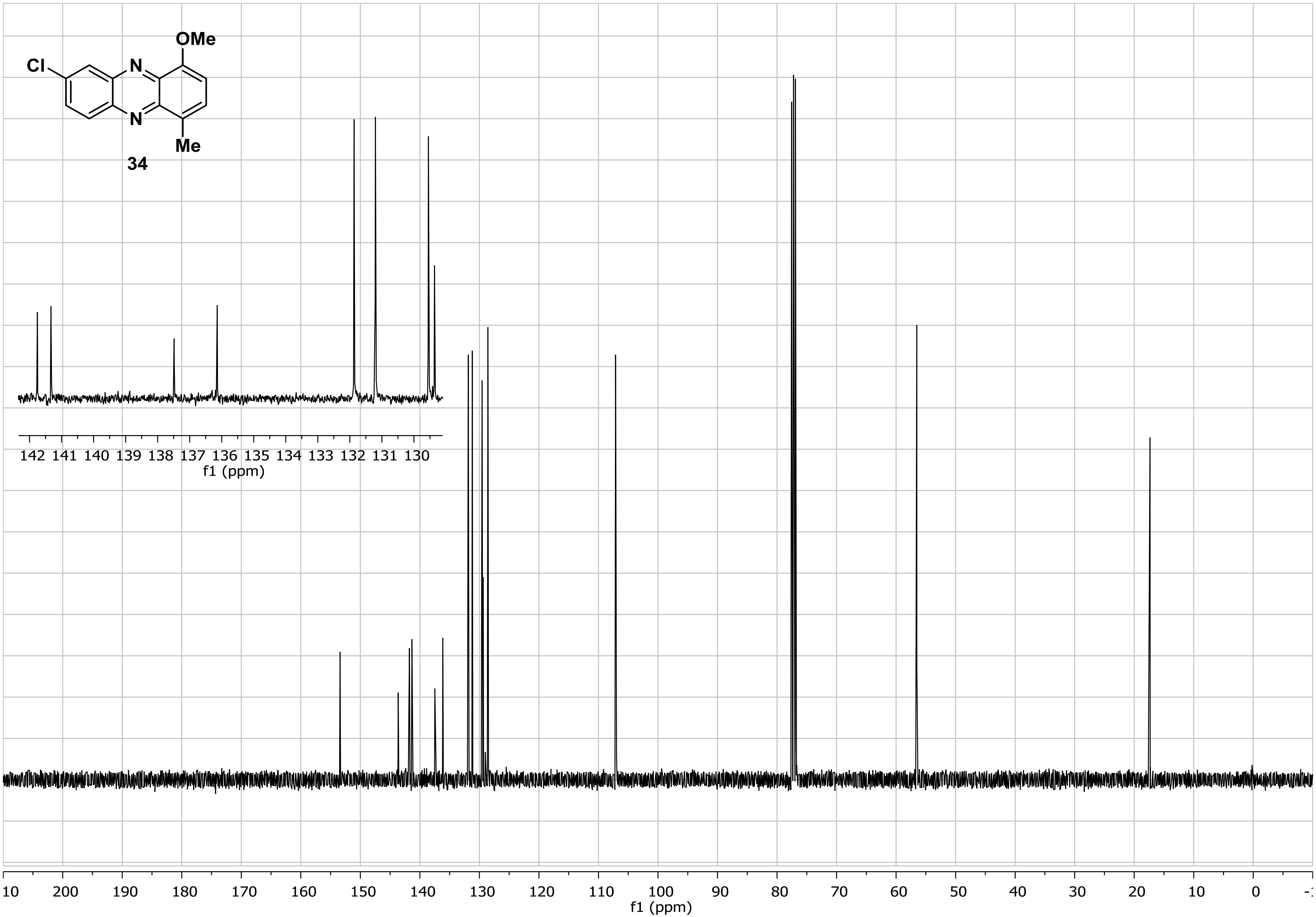

OMe
Cl
N
N
Me
34
142 141 140 139 138 137 136 135 134 133 132 131 130
f1 (ppm)
10 200 190 180 170 160 150 140 130 120 110 100 90 80 70 60 50 40 30 20 10 0
f1 (ppm)

NMR-65

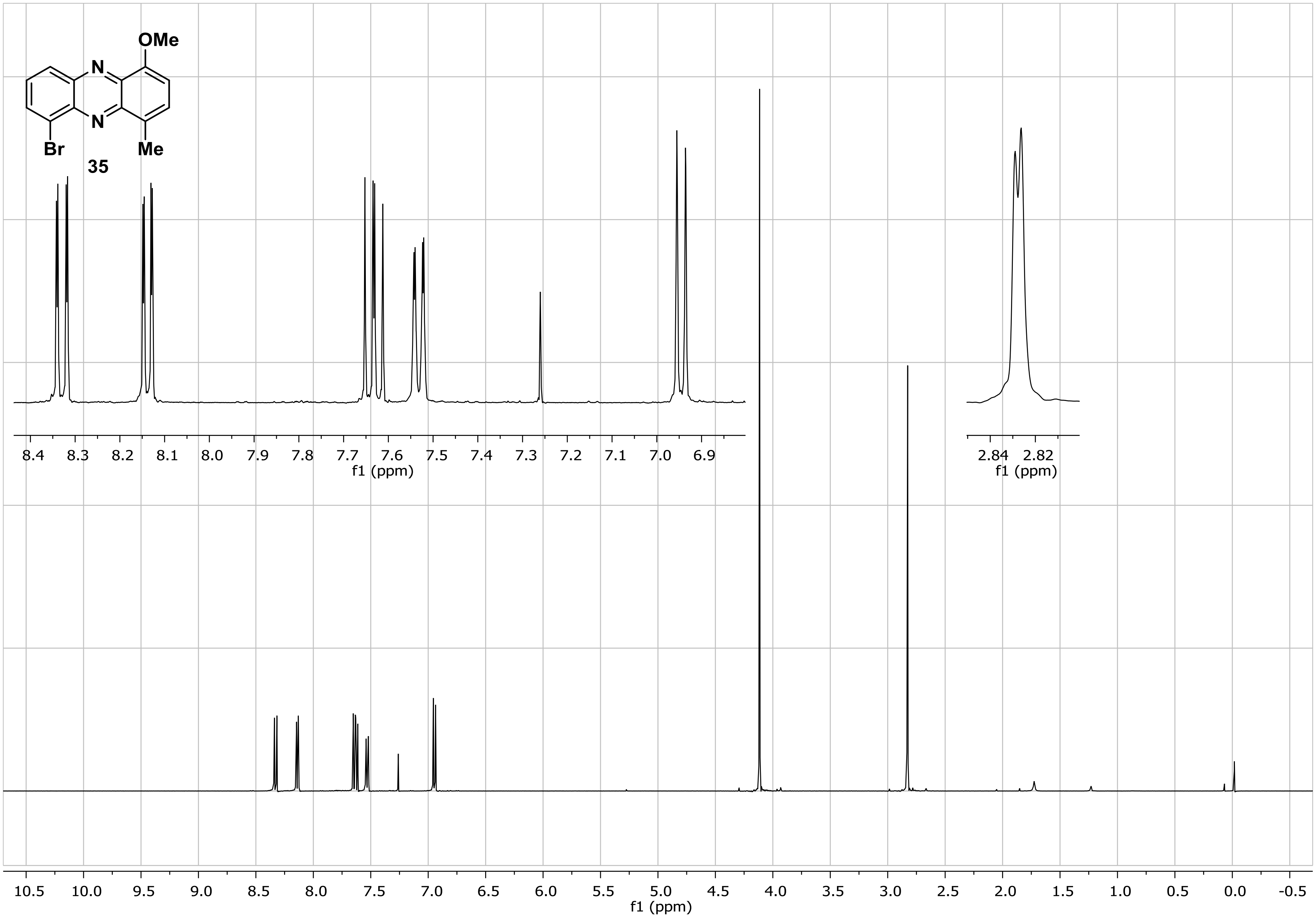

NMR-66

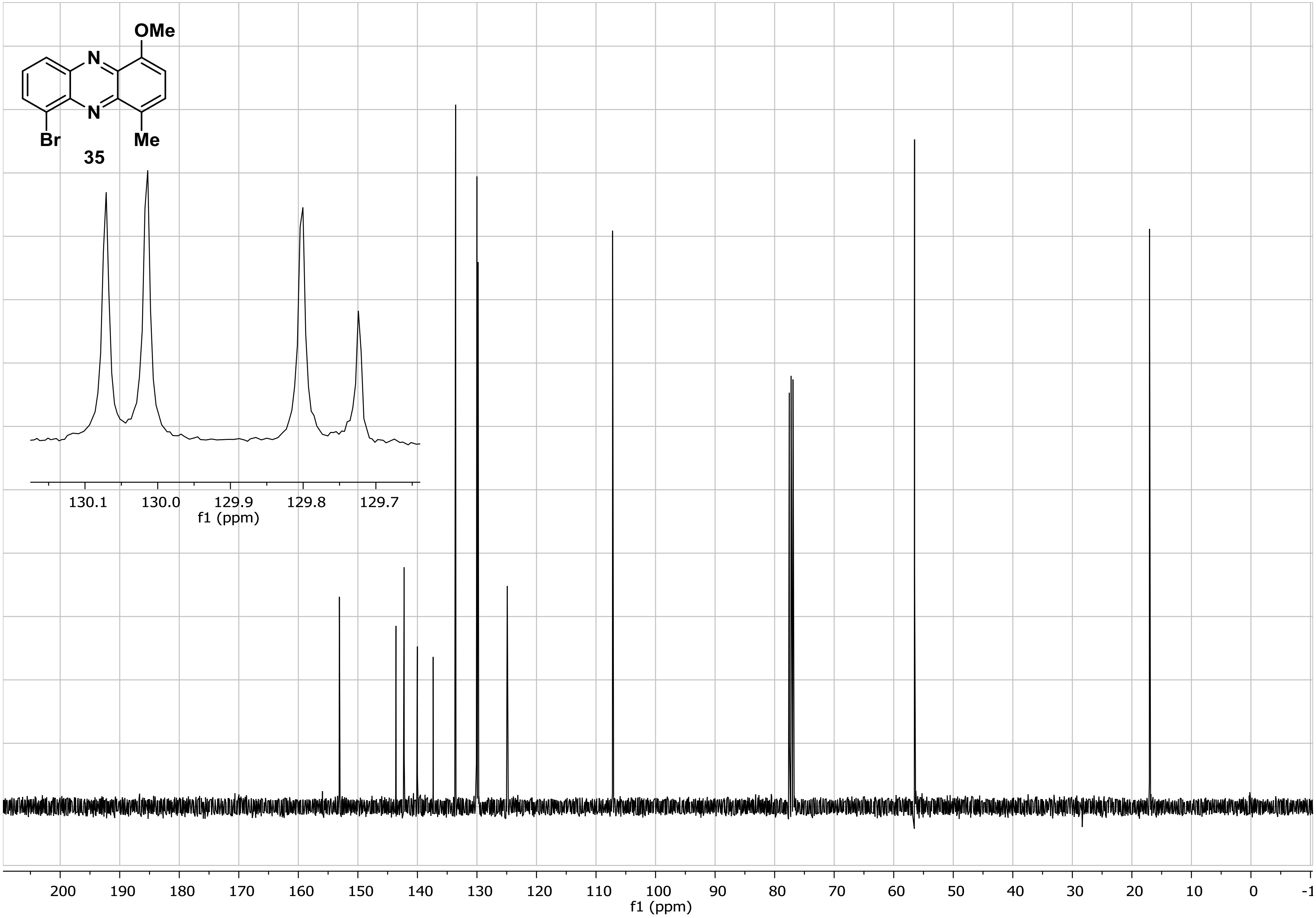
OMe
N
N
Br
Me
35
130.1
130.0
129.9
129.8
129.7
f1 (ppm)
200
190
180
170
160
150
140
130
120
110
100
90
80
70
60
50
40
30
20
10
0
-1
f1 (ppm)

NMR-67

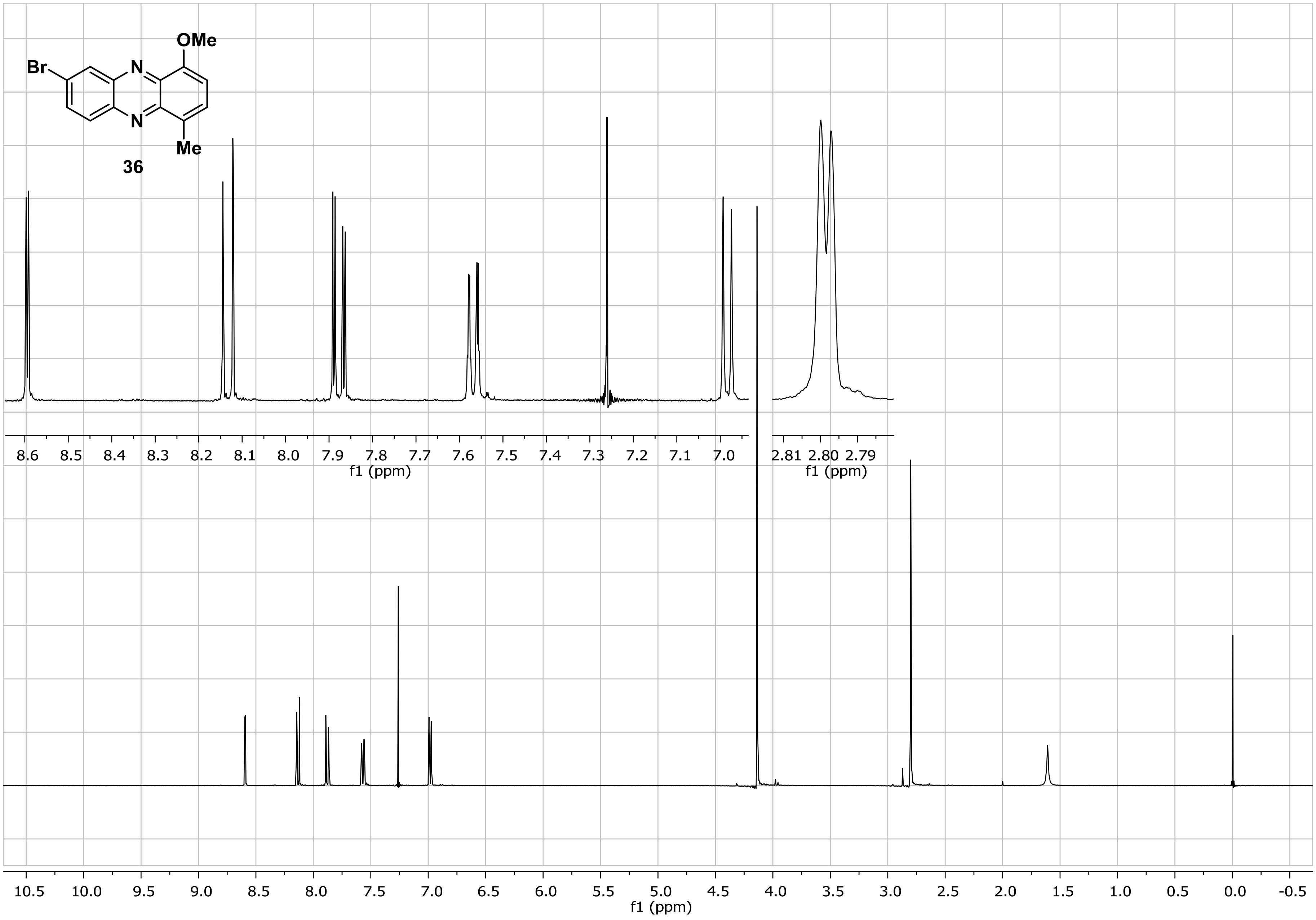

NMR-68

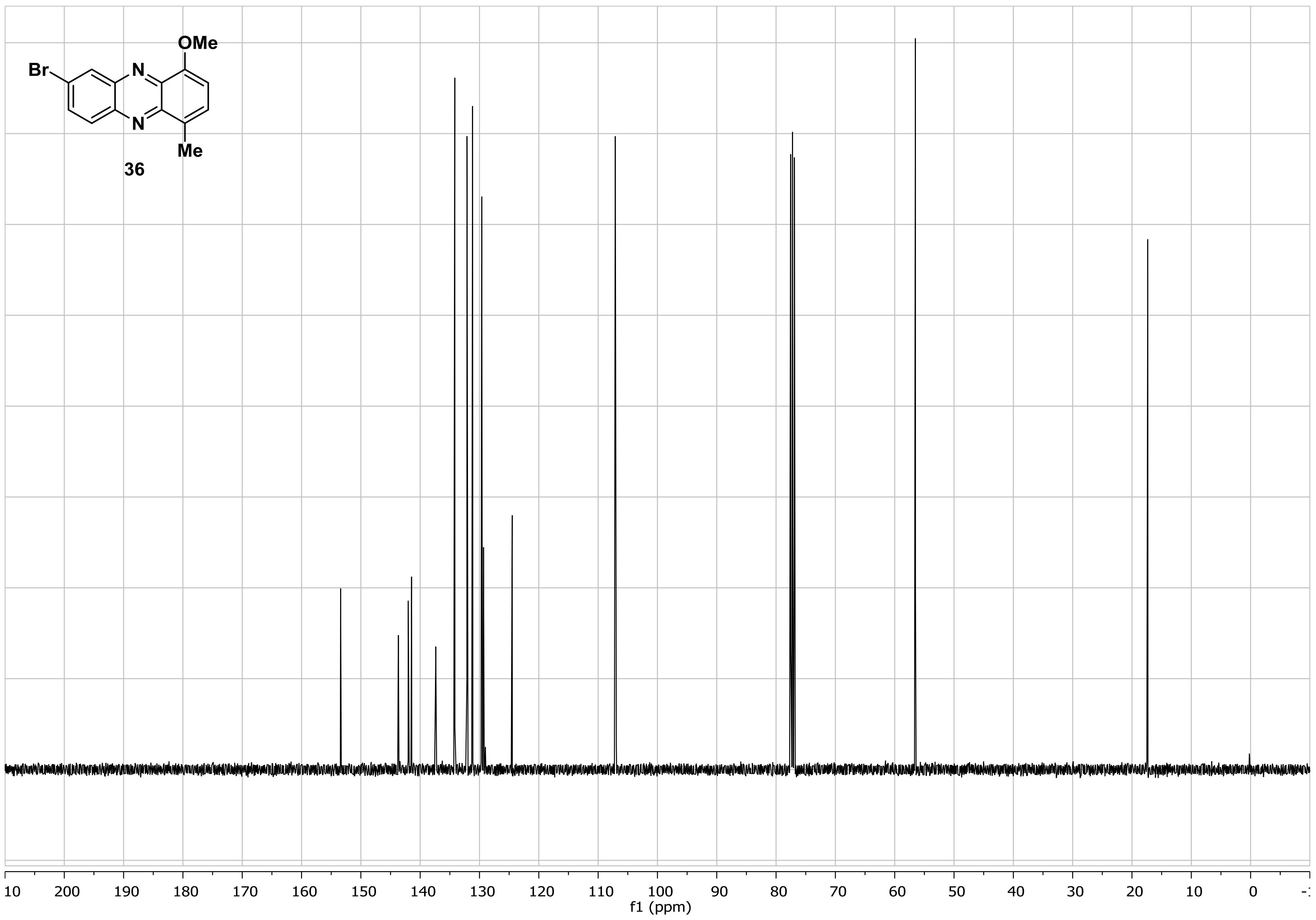

NMR-69

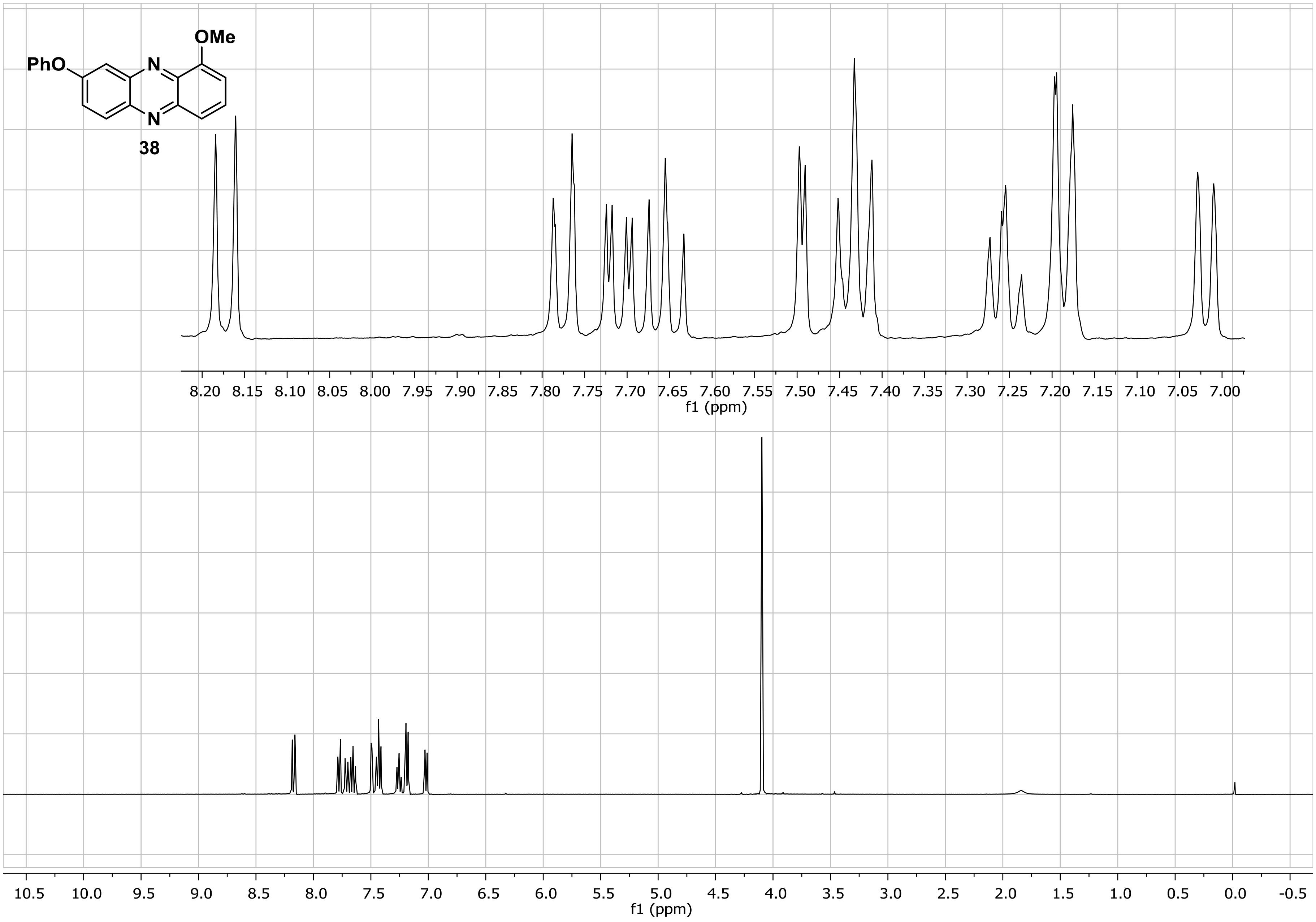
OMe
PhO
N
N
38
8.20 8.15 8.10 8.05 8.00 7.95 7.90 7.85 7.80 7.75 7.70 7.65 7.60 7.55 7.50 7.45 7.40 7.35 7.30 7.25 7.20 7.15 7.10 7.05 7.00
f1 (ppm)
10.5 10.0 9.5 9.0 8.5 8.0 7.5 7.0 6.5 6.0 5.5 5.0 4.5 4.0 3.5 3.0 2.5 2.0 1.5 1.0 0.5 0.0 -0.5
f1 (ppm)

NMR-70

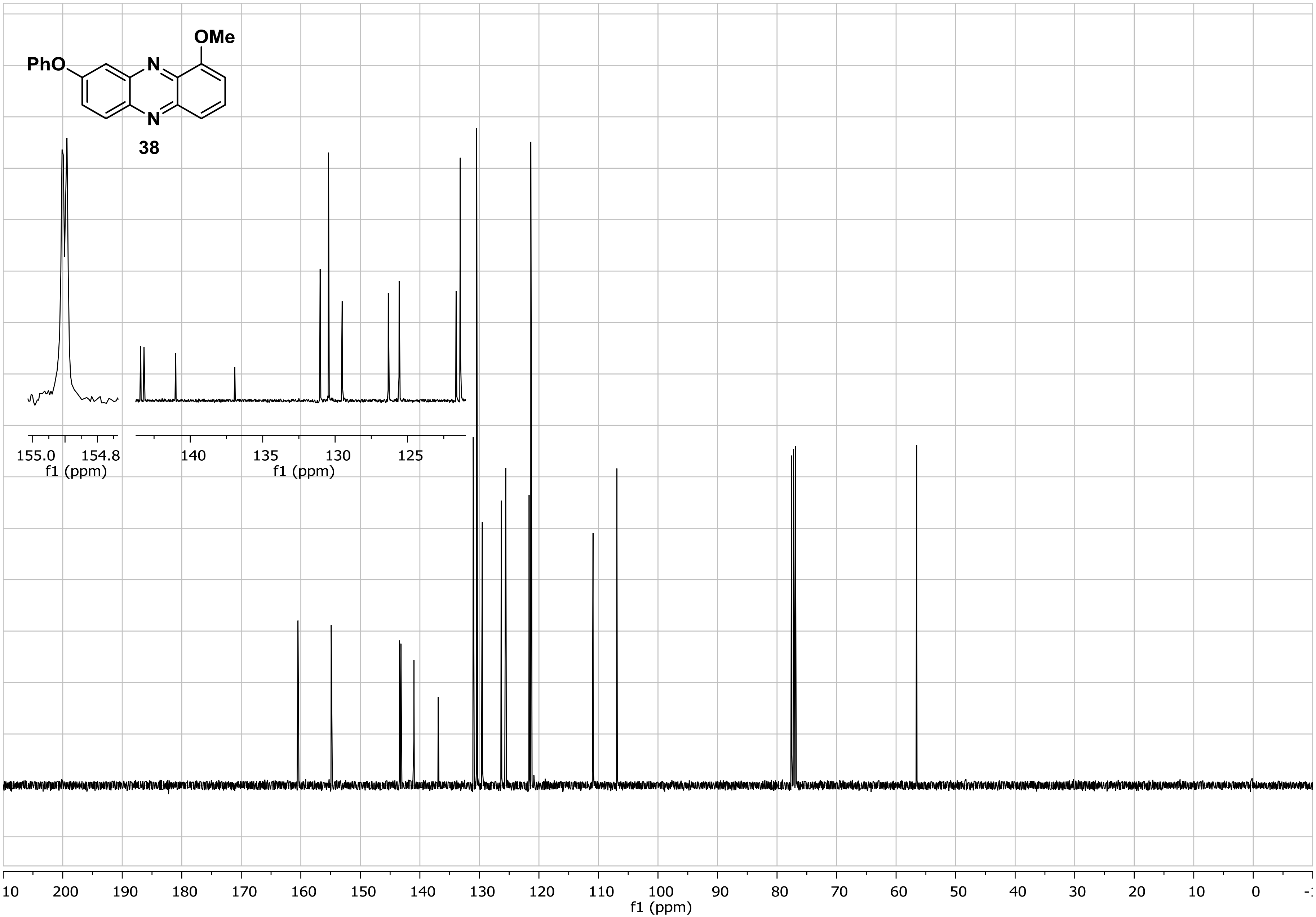
OMe
PhO
N
N
38
155.0
154.8
f1 (ppm)
140
135
130
125
f1 (ppm)
200
190
180
170
160
150
140
130
120
110
100
90
80
70
60
50
40
30
20
10
0
f1 (ppm)

NMR-71

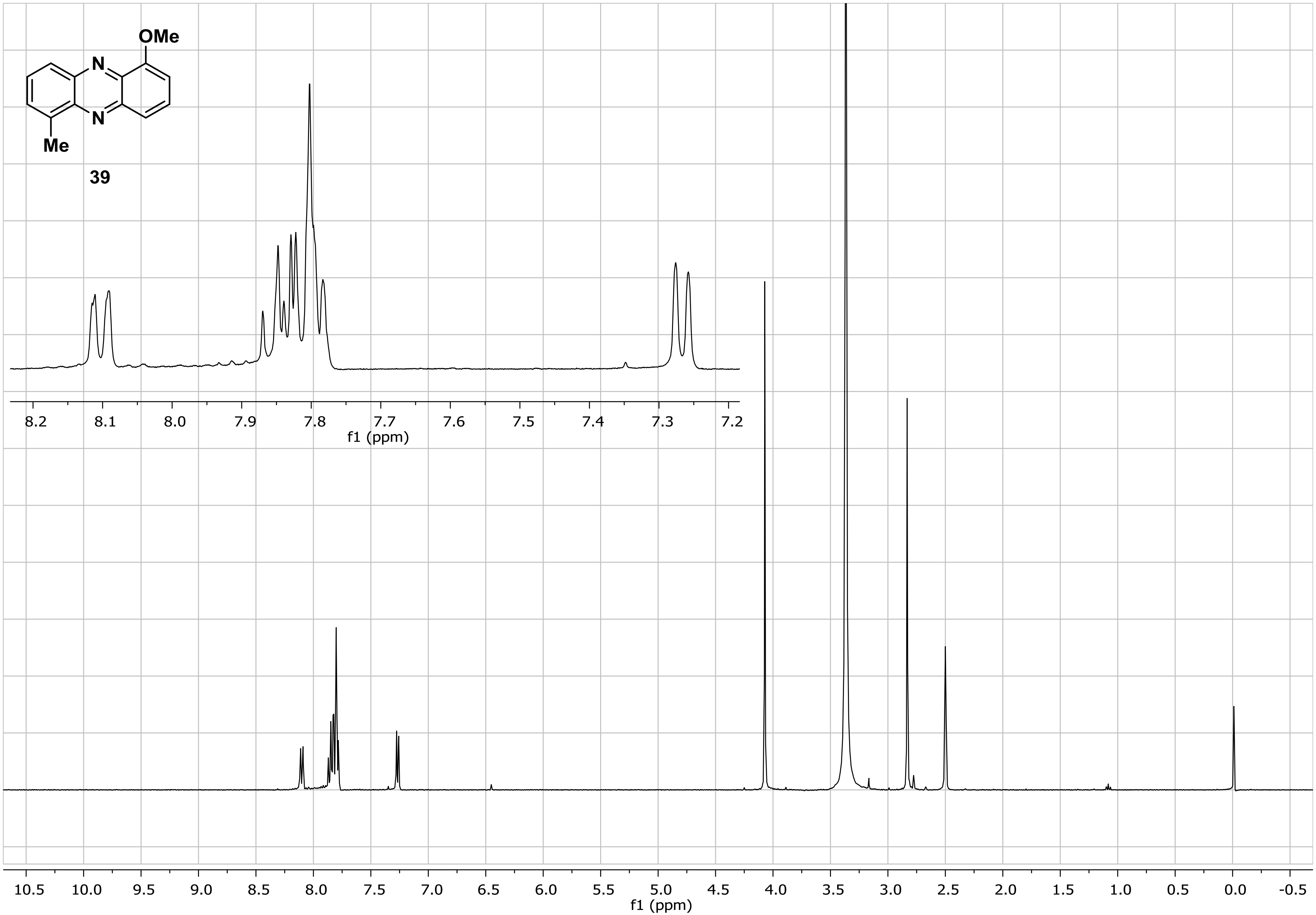

NMR-72

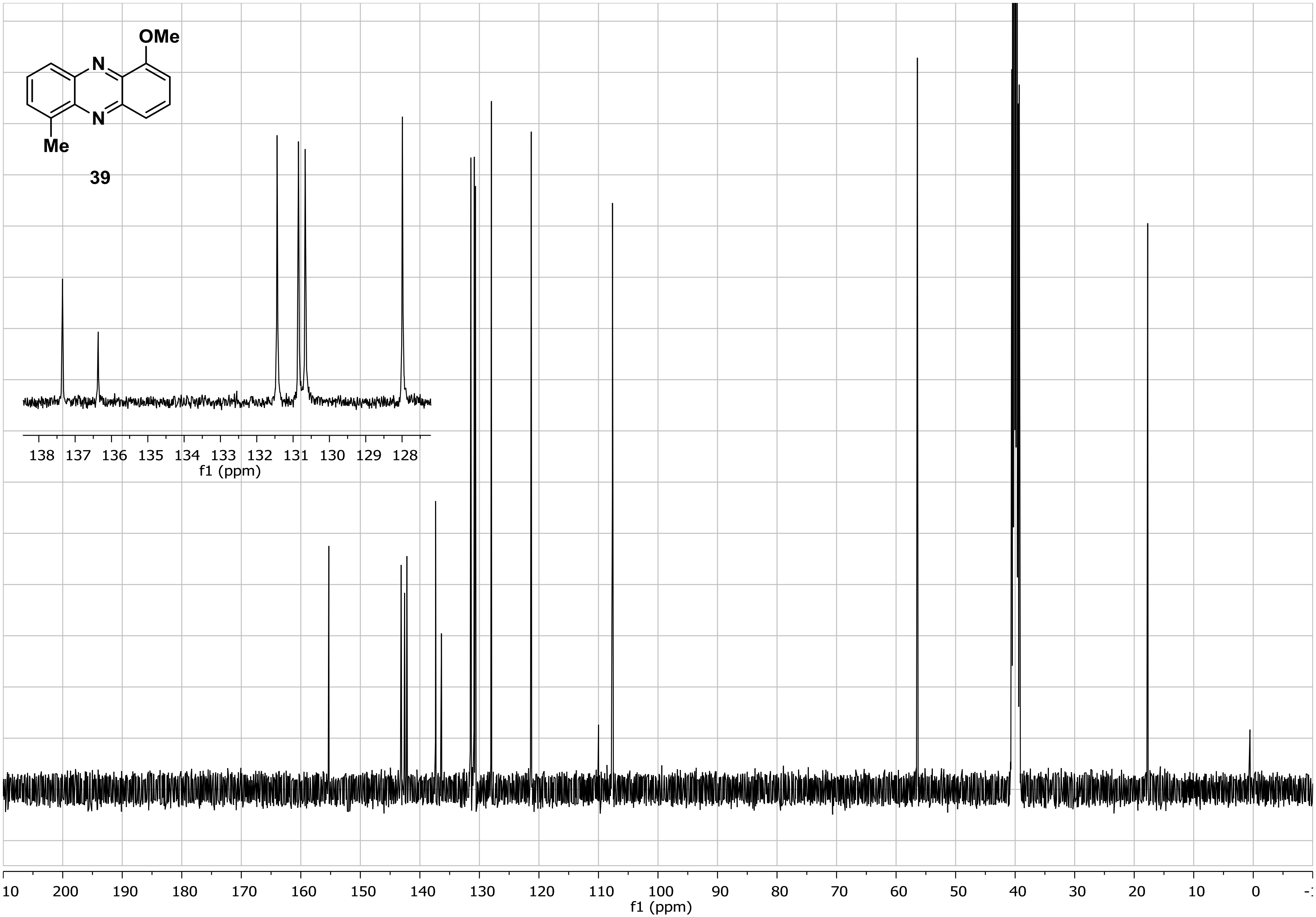
OMe
N
N
Me
39
138 137 136 135 134 133 132 131 130 129 128
f1 (ppm)
10 200 190 180 170 160 150 140 130 120 110 100 90 80 70 60 50 40 30 20 10 0
f1 (ppm)

NMR-73

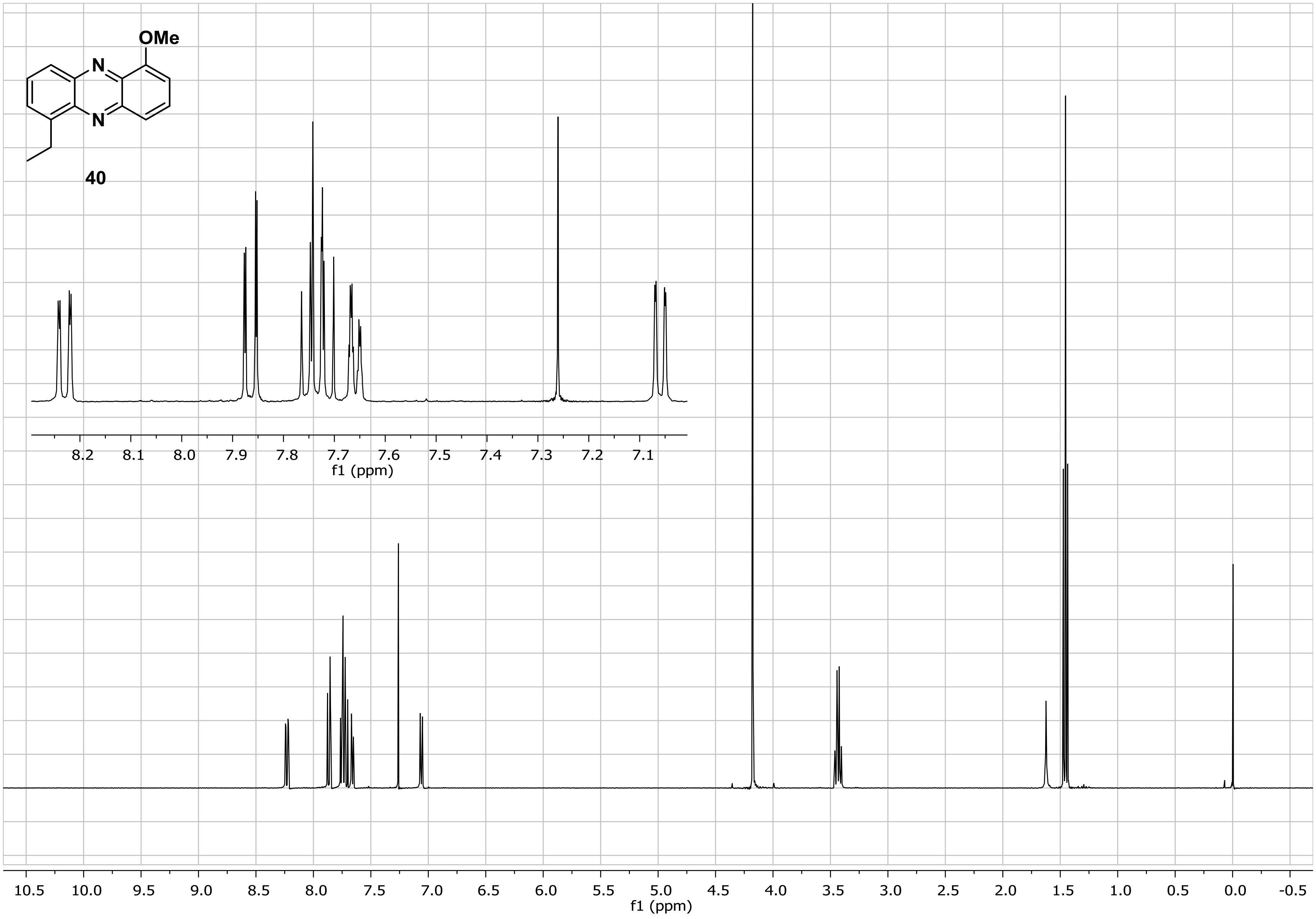
OMe
N
N
40
8.2
8.1
8.0
7.9
7.8
7.7
7.6
7.5
7.4
7.3
7.2
7.1
f1 (ppm)
10.5
10.0
9.5
9.0
8.5
8.0
7.5
7.0
6.5
6.0
5.5
5.0
4.5
4.0
3.5
3.0
2.5
2.0
1.5
1.0
0.5
0.0
-0.5
f1 (ppm)

NMR-74

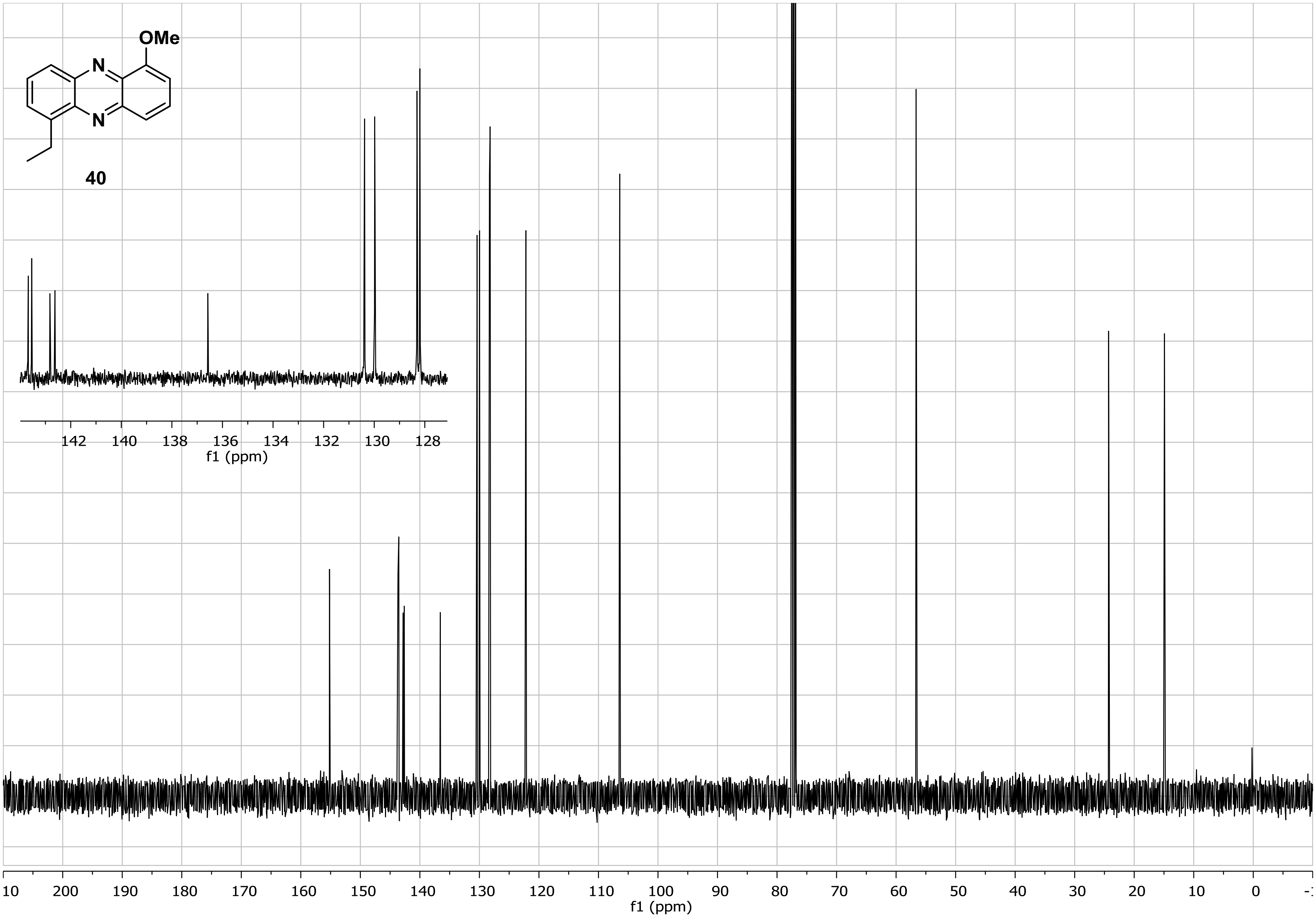
OMe
N
N
40
142
140
138
136
134
132
130
128
f1 (ppm)
10
200
190
180
170
160
150
140
130
120
110
100
90
80
70
60
50
40
30
20
10
0
f1 (ppm)

NMR-75

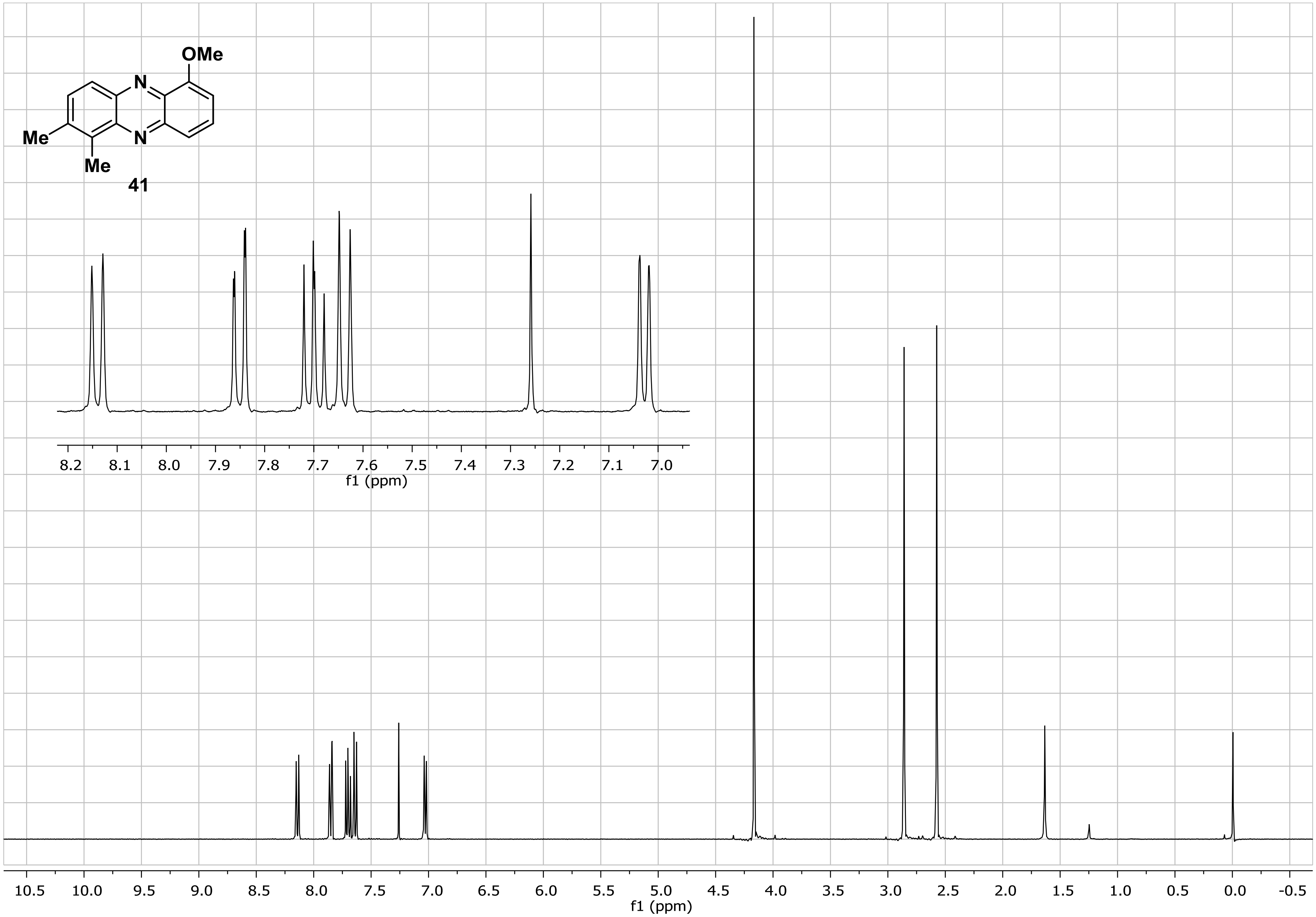
OMe
N
N
Me
Me
41
8.2 8.1 8.0 7.9 7.8 7.7 7.6 7.5 7.4 7.3 7.2 7.1 7.0
f1 (ppm)
10.5 10.0 9.5 9.0 8.5 8.0 7.5 7.0 6.5 6.0 5.5 5.0 4.5 4.0 3.5 3.0 2.5 2.0 1.5 1.0 0.5 0.0 -0.5
f1 (ppm)

NMR-76

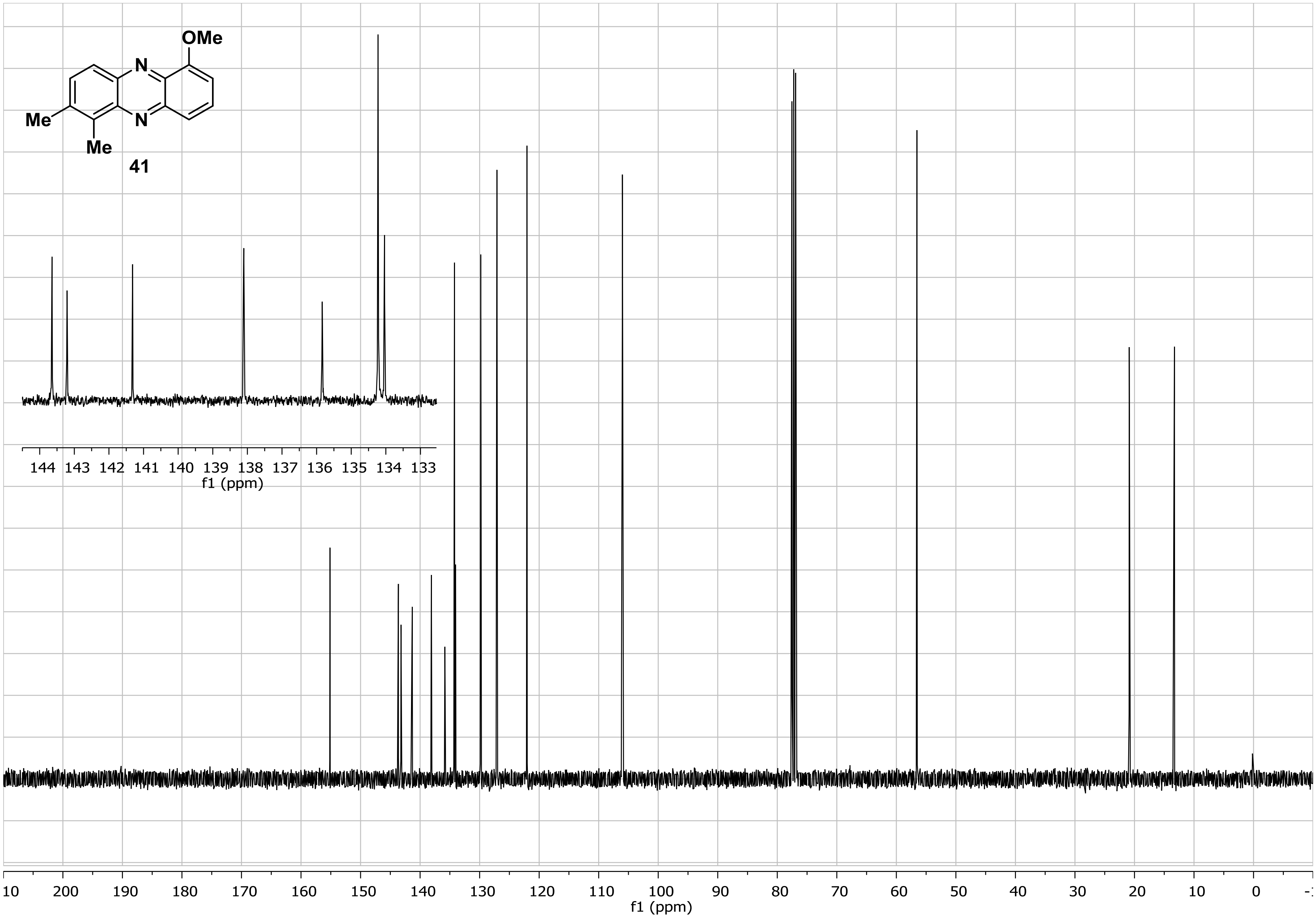
OMe
N
N
Me
Me
41
144 143 142 141 140 139 138 137 136 135 134 133
f1 (ppm)
10 200 190 180 170 160 150 140 130 120 110 100 90 80 70 60 50 40 30 20 10 0
f1 (ppm)

NMR-77

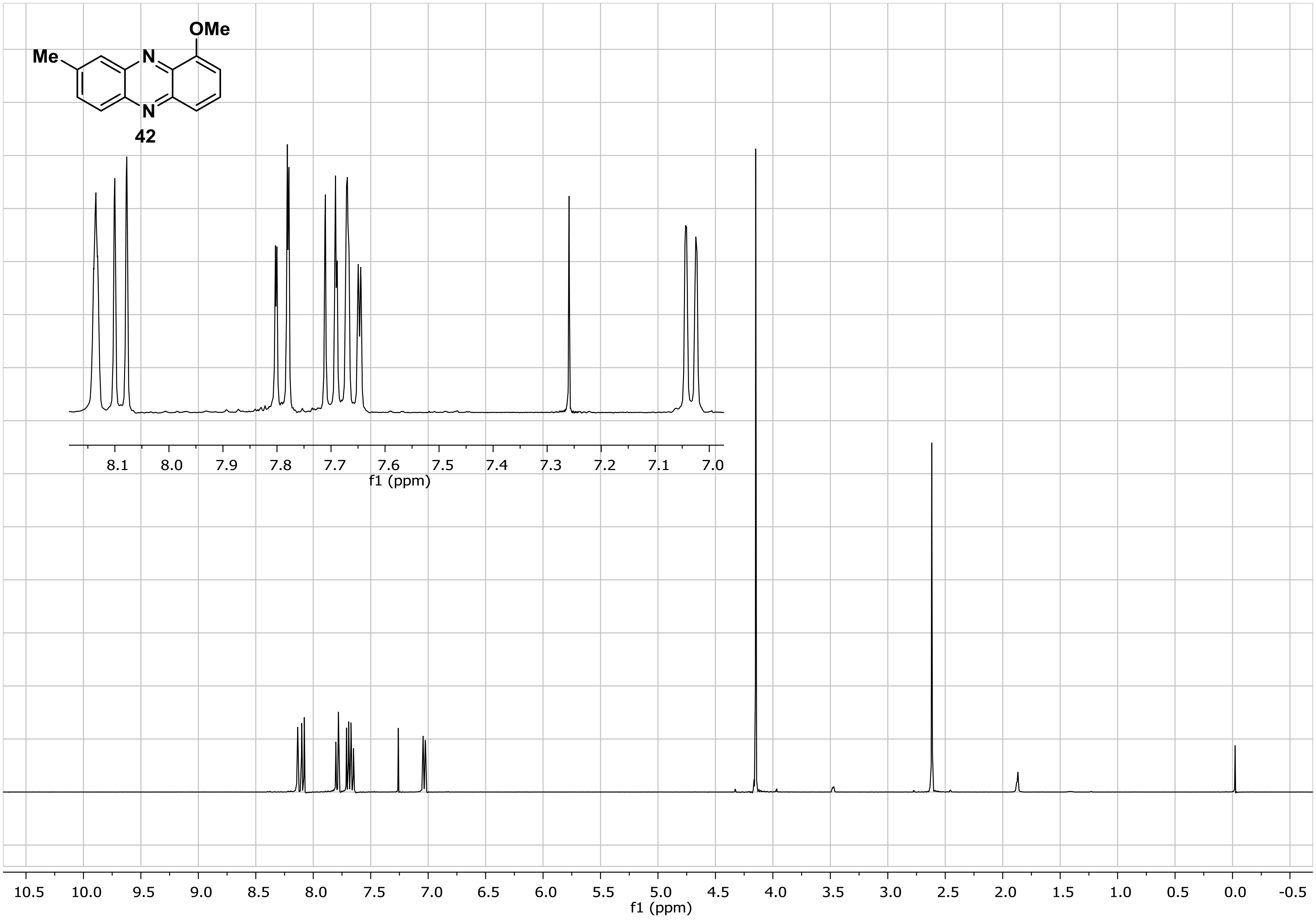
OMe
Me
N
N
42
8.1
8.0
7.9
7.8
7.7
7.6
7.5
7.4
7.3
7.2
7.1
7.0
f1 (ppm)
10.5
10.0
9.5
9.0
8.5
8.0
7.5
7.0
6.5
6.0
5.5
5.0
4.5
4.0
3.5
3.0
2.5
2.0
1.5
1.0
0.5
0.0
-0.5
f1 (ppm)

NMR-78

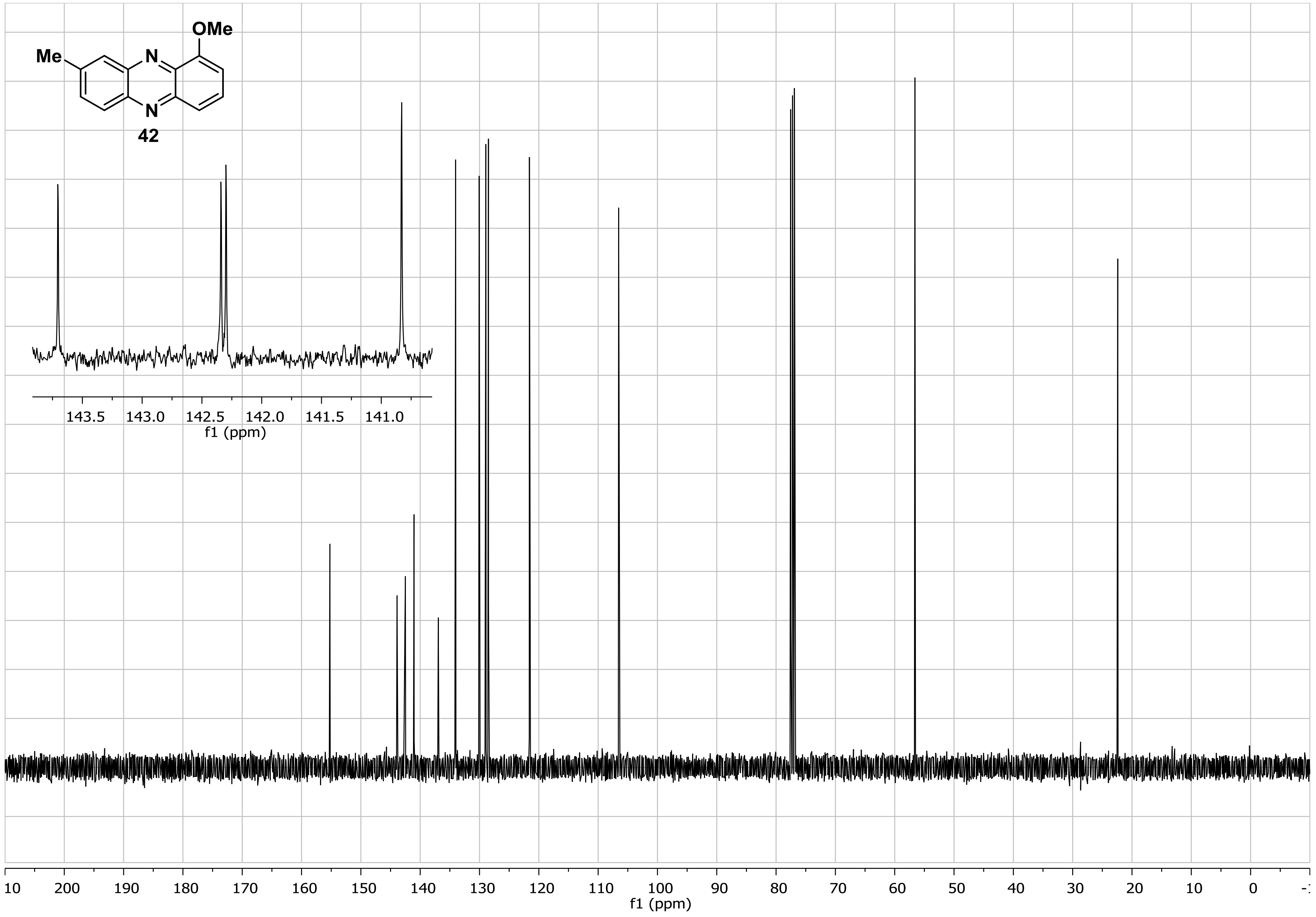
OMe
Me
N
N
42
143.5
143.0
142.5
142.0
141.5
141.0
f1 (ppm)
200
190
180
170
160
150
140
130
120
110
100
90
80
70
60
50
40
30
20
10
0
f1 (ppm)

NMR-79

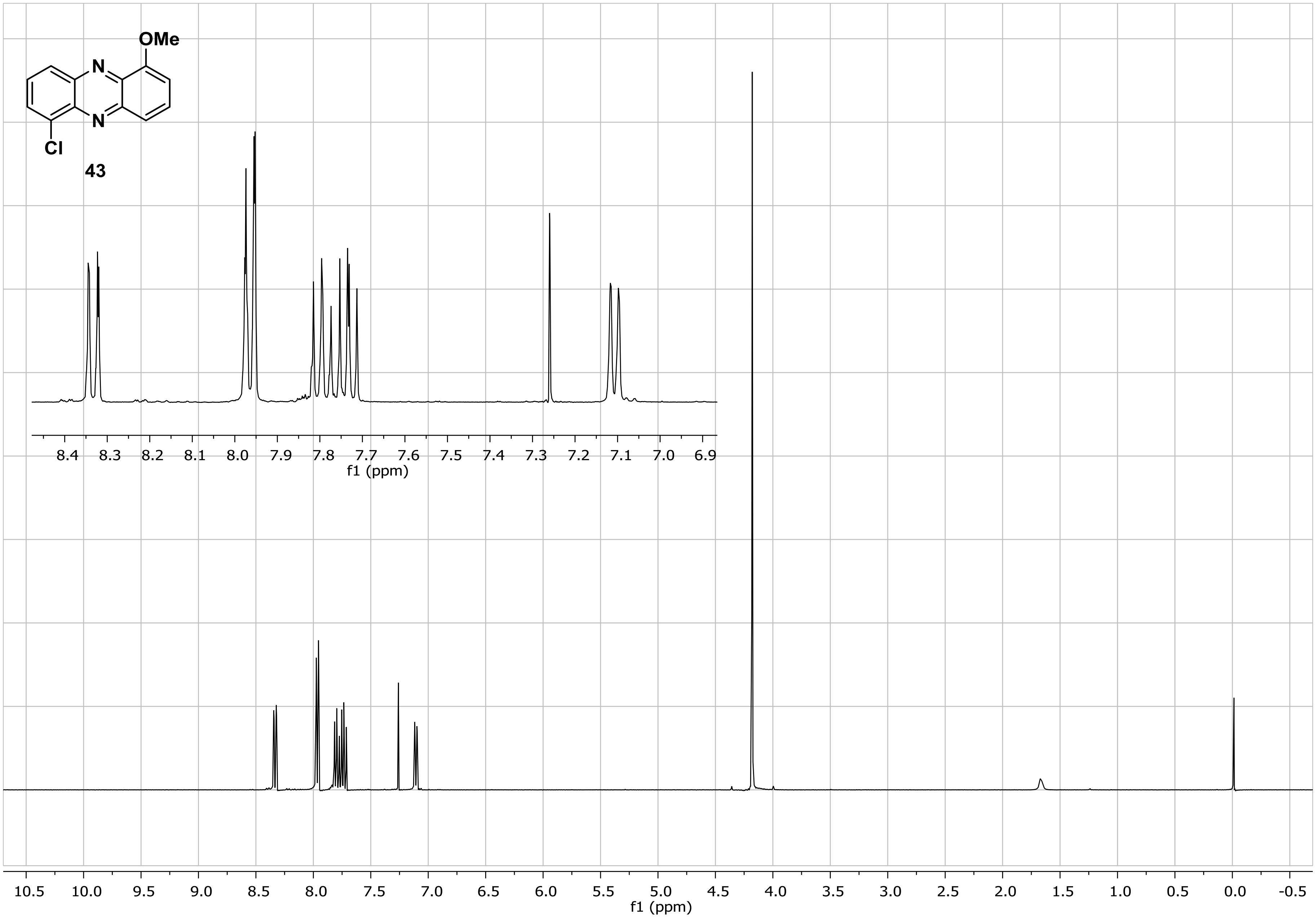
OMe
N
N
Cl
43
8.4 8.3 8.2 8.1 8.0 7.9 7.8 7.7 7.6 7.5 7.4 7.3 7.2 7.1 7.0 6.9
f1 (ppm)
10.5 10.0 9.5 9.0 8.5 8.0 7.5 7.0 6.5 6.0 5.5 5.0 4.5 4.0 3.5 3.0 2.5 2.0 1.5 1.0 0.5 0.0 -0.5
f1 (ppm)

NMR-80

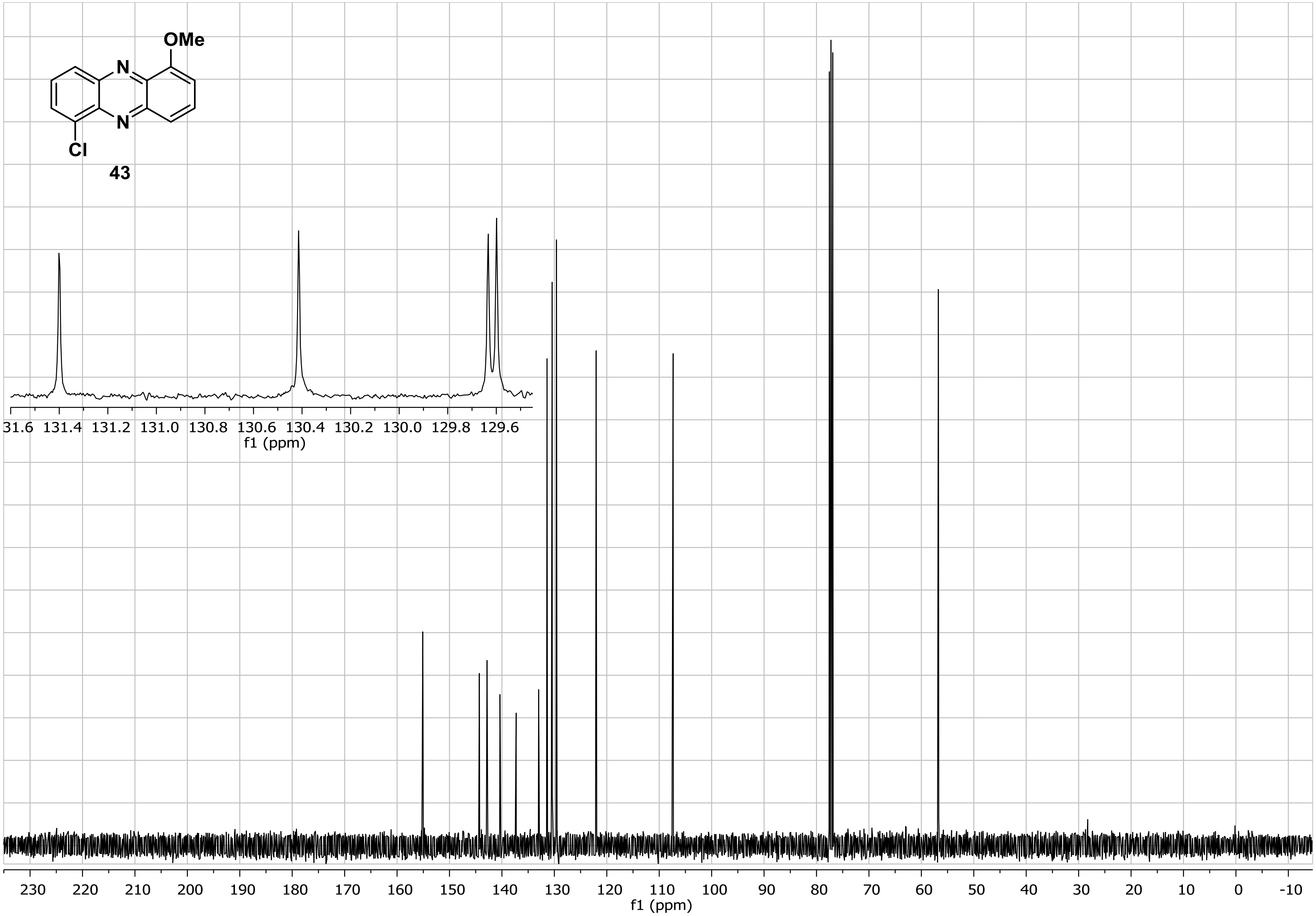
OMe
N
N
Cl
43
31.6
131.4
131.2
131.0
130.8
130.6
130.4
130.2
130.0
129.8
129.6
f1 (ppm)
230
220
210
200
190
180
170
160
150
140
130
120
110
100
90
80
70
60
50
40
30
20
10
0
-10
f1 (ppm)

NMR-81

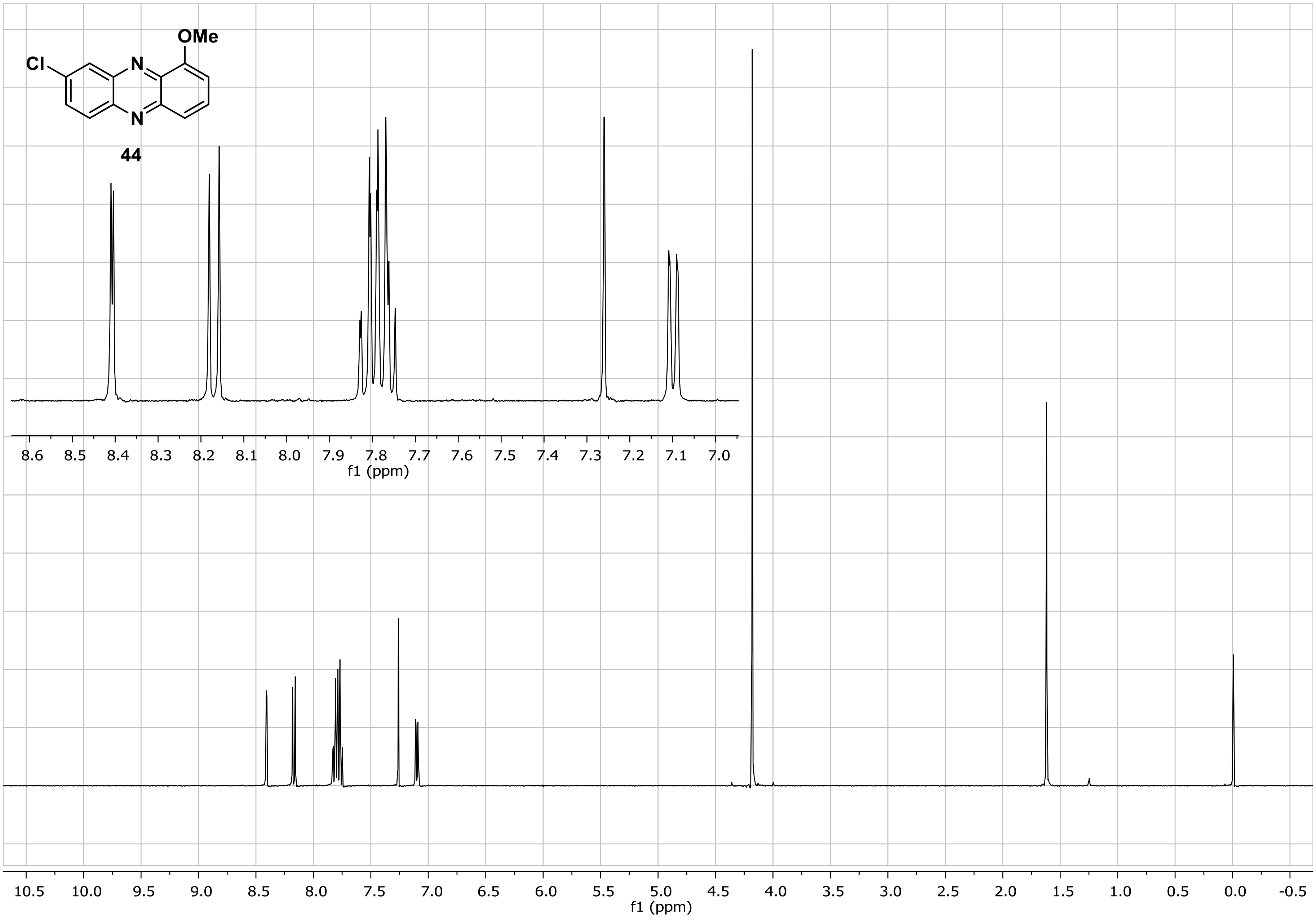
OMe
Cl
N
N
44
8.6 8.5 8.4 8.3 8.2 8.1 8.0 7.9 7.8 7.7 7.6 7.5 7.4 7.3 7.2 7.1 7.0
f1 (ppm)
10.5 10.0 9.5 9.0 8.5 8.0 7.5 7.0 6.5 6.0 5.5 5.0 4.5 4.0 3.5 3.0 2.5 2.0 1.5 1.0 0.5 0.0 -0.5
f1 (ppm)

NMR-82

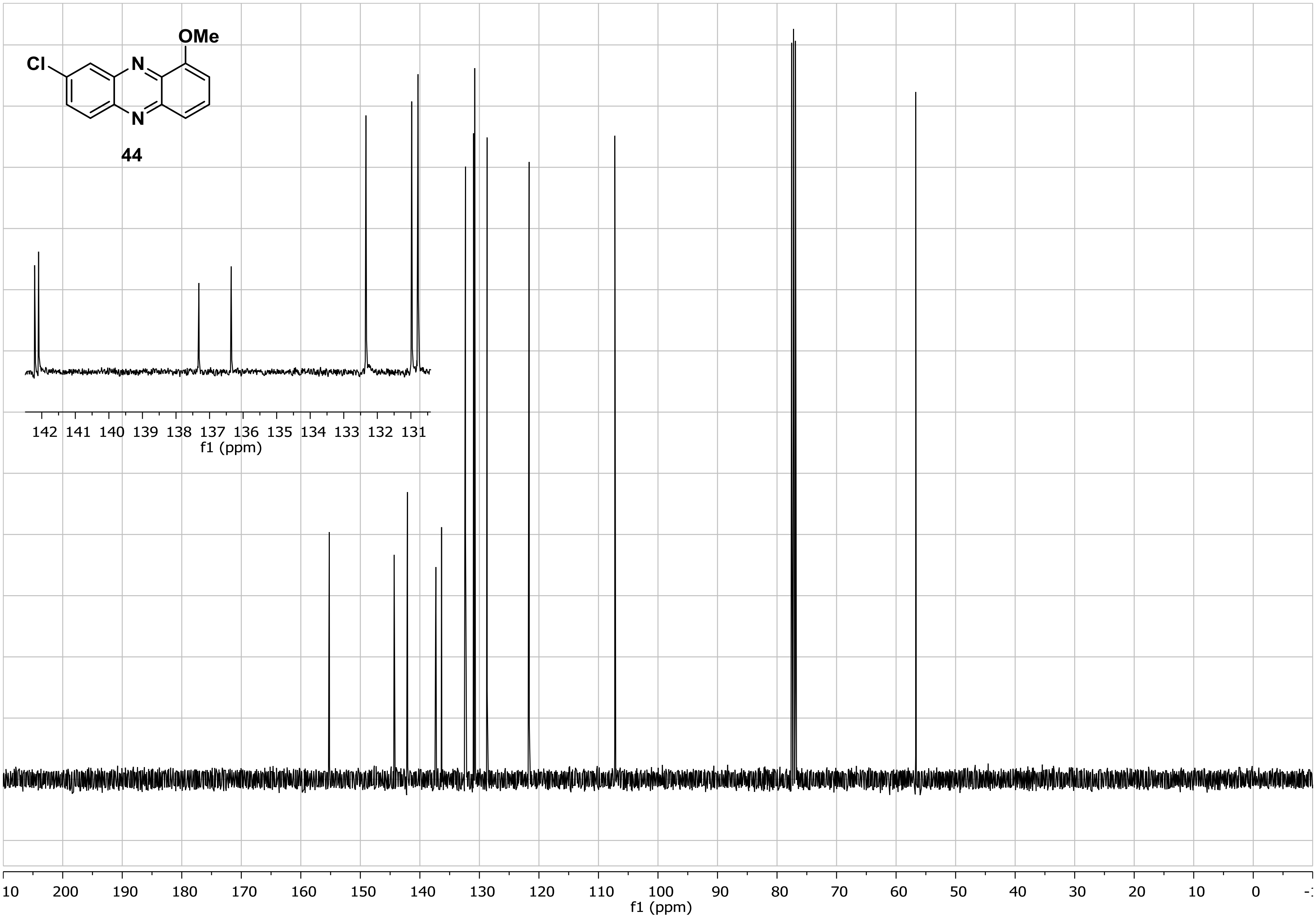
OMe
Cl
N
N
44
142 141 140 139 138 137 136 135 134 133 132 131
f1 (ppm)
10 200 190 180 170 160 150 140 130 120 110 100 90 80 70 60 50 40 30 20 10 0
f1 (ppm)

NMR-83

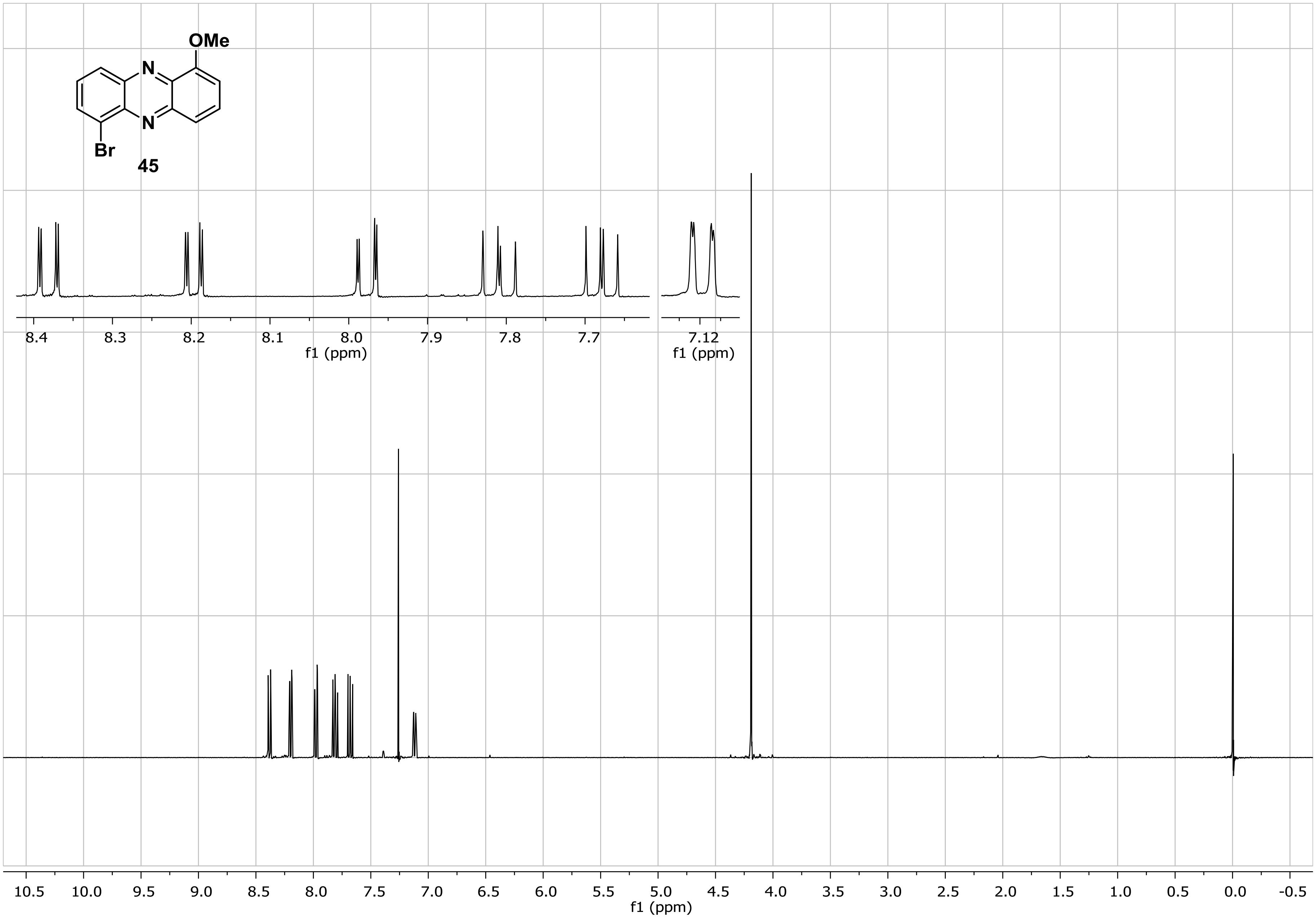
OMe
N
N
Br
45
8.4
8.3
8.2
8.1
8.0
7.9
7.8
7.7
f1 (ppm)
7.12
f1 (ppm)
10.5
10.0
9.5
9.0
8.5
8.0
7.5
7.0
6.5
6.0
5.5
5.0
4.5
4.0
3.5
3.0
2.5
2.0
1.5
1.0
0.5
0.0
-0.5
f1 (ppm)

NMR-84

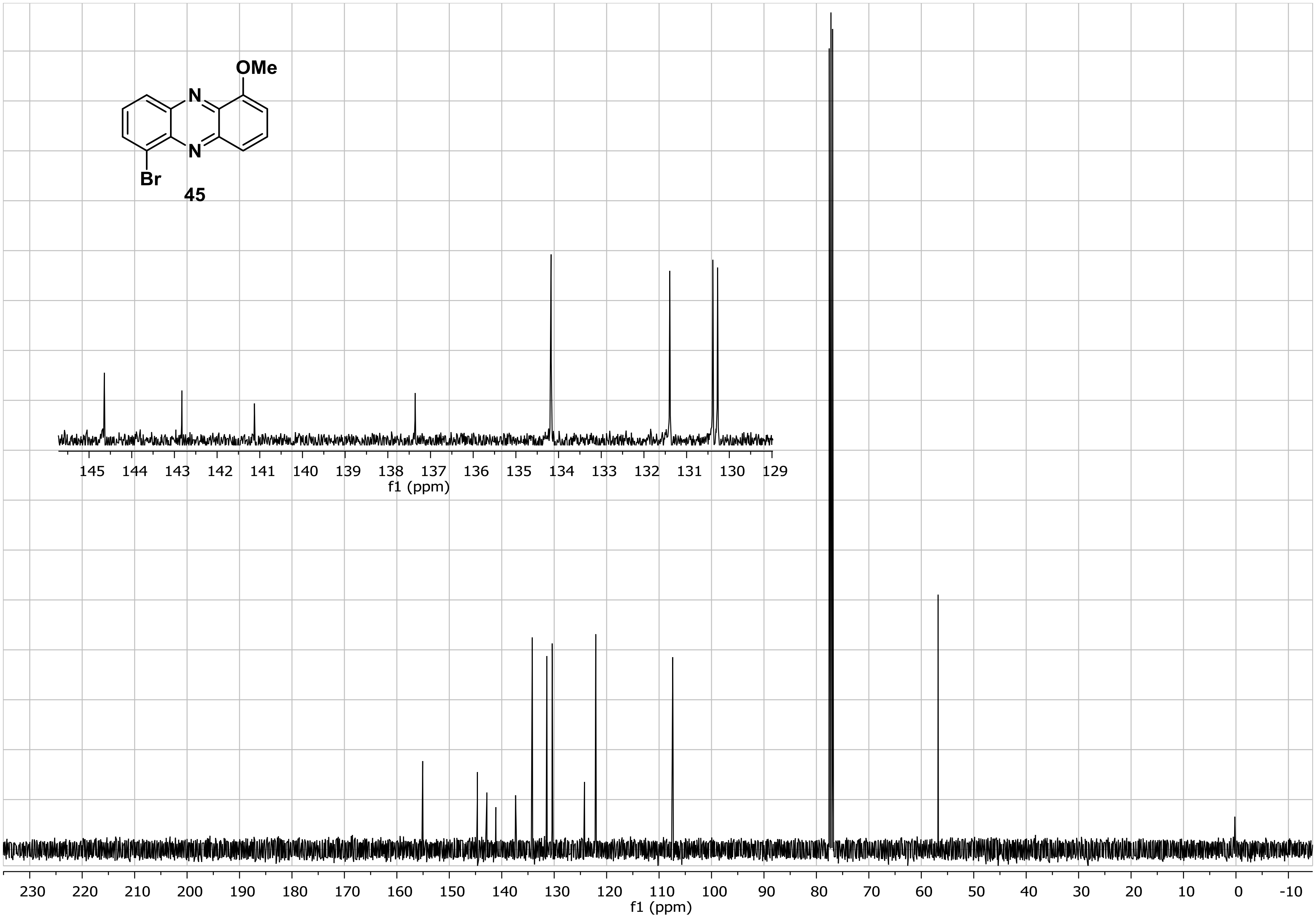
OMe
N
N
Br
45
145
144
143
142
141
140
139
138
137
136
135
134
133
132
131
130
129
f1 (ppm)
230
220
210
200
190
180
170
160
150
140
130
120
110
100
90
80
70
60
50
40
30
20
10
0
-10
f1 (ppm)

NMR-85

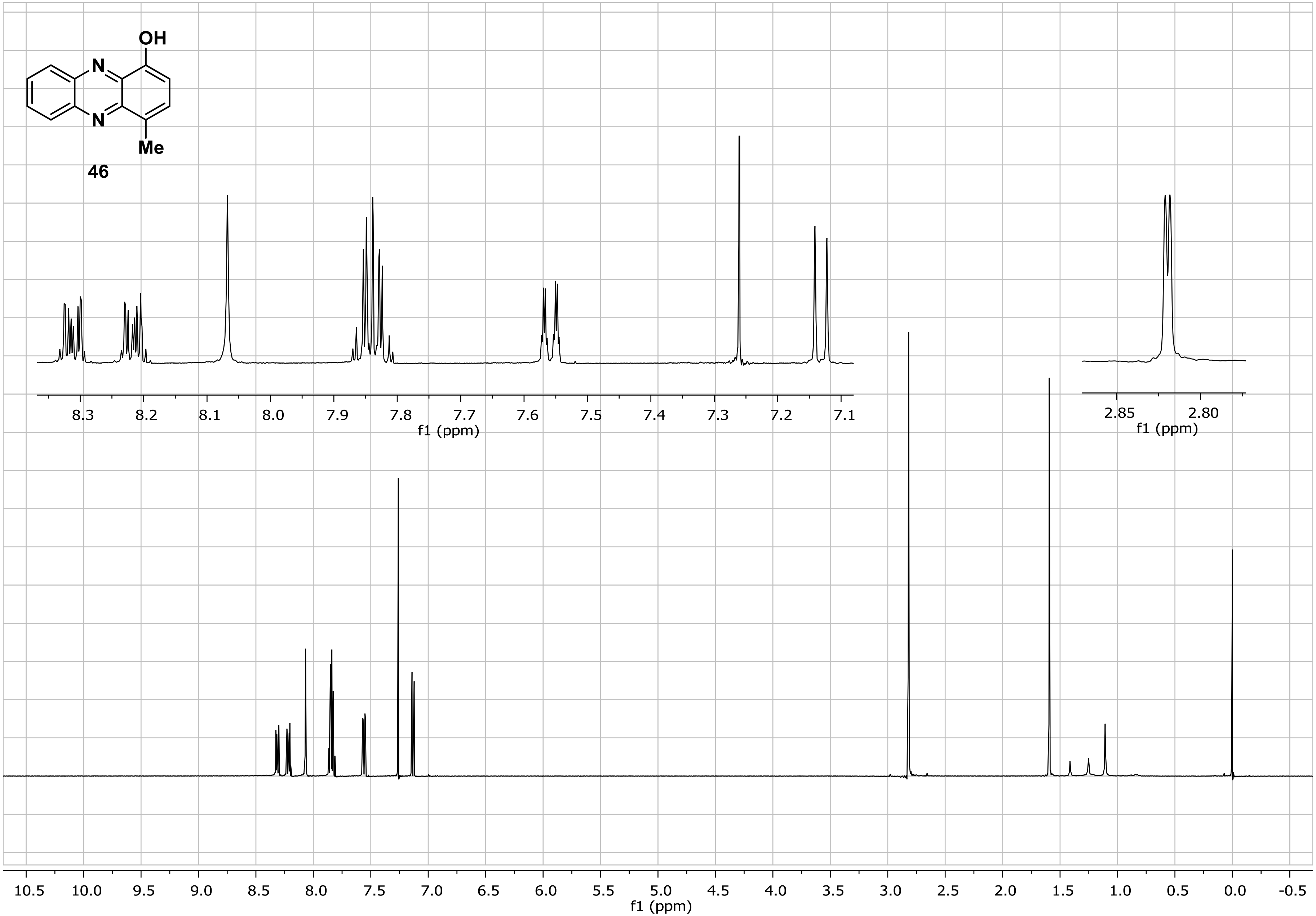

NMR-86

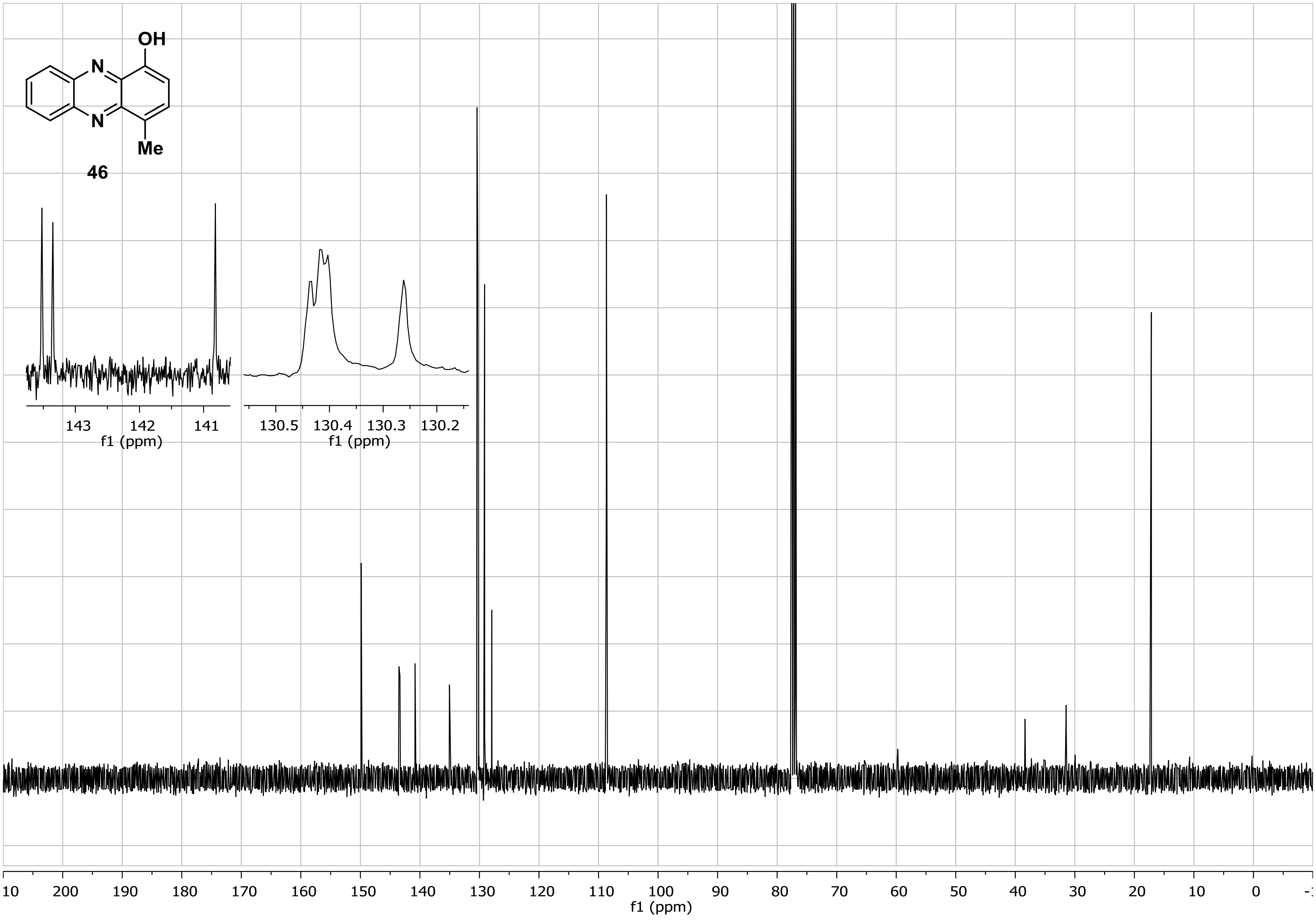

OH
N
N
Me
46
143
142
141
f1 (ppm)
130.5
130.4
130.3
130.2
f1 (ppm)
200
190
180
170
160
150
140
130
120
110
100
90
80
70
60
50
40
30
20
10
0
f1 (ppm)

NMR-87

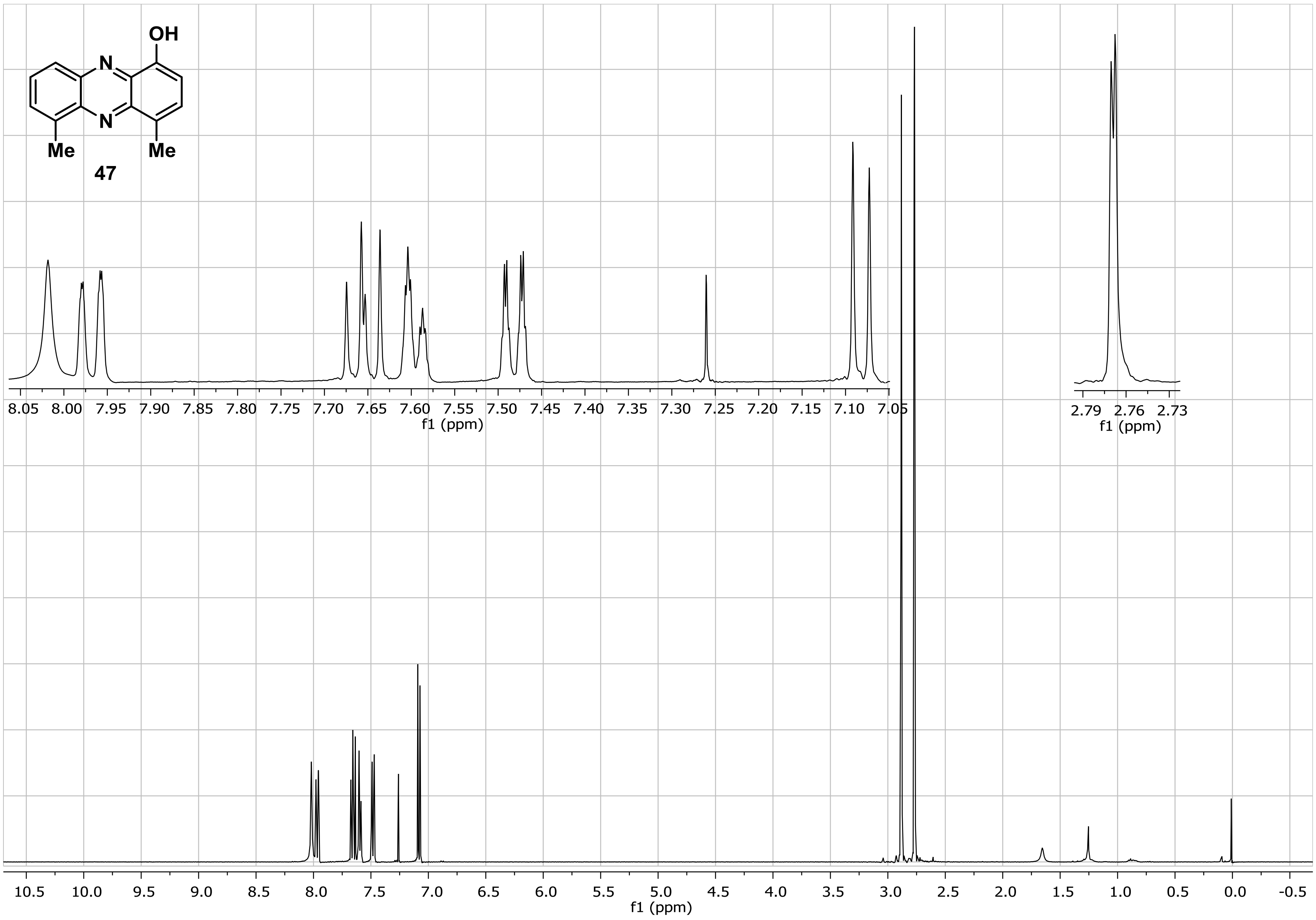

OH
N
N
Me
Me
47
8.05
8.00
7.95
7.90
7.85
7.80
7.75
7.70
7.65
7.60
7.55
7.50
7.45
7.40
7.35
7.30
7.25
7.20
7.15
7.10
7.05
f1 (ppm)
2.79
2.76
2.73
f1 (ppm)
10.5
10.0
9.5
9.0
8.5
8.0
7.5
7.0
6.5
6.0
5.5
5.0
4.5
4.0
3.5
3.0
2.5
2.0
1.5
1.0
0.5
0.0
-0.5
f1 (ppm)

NMR-88

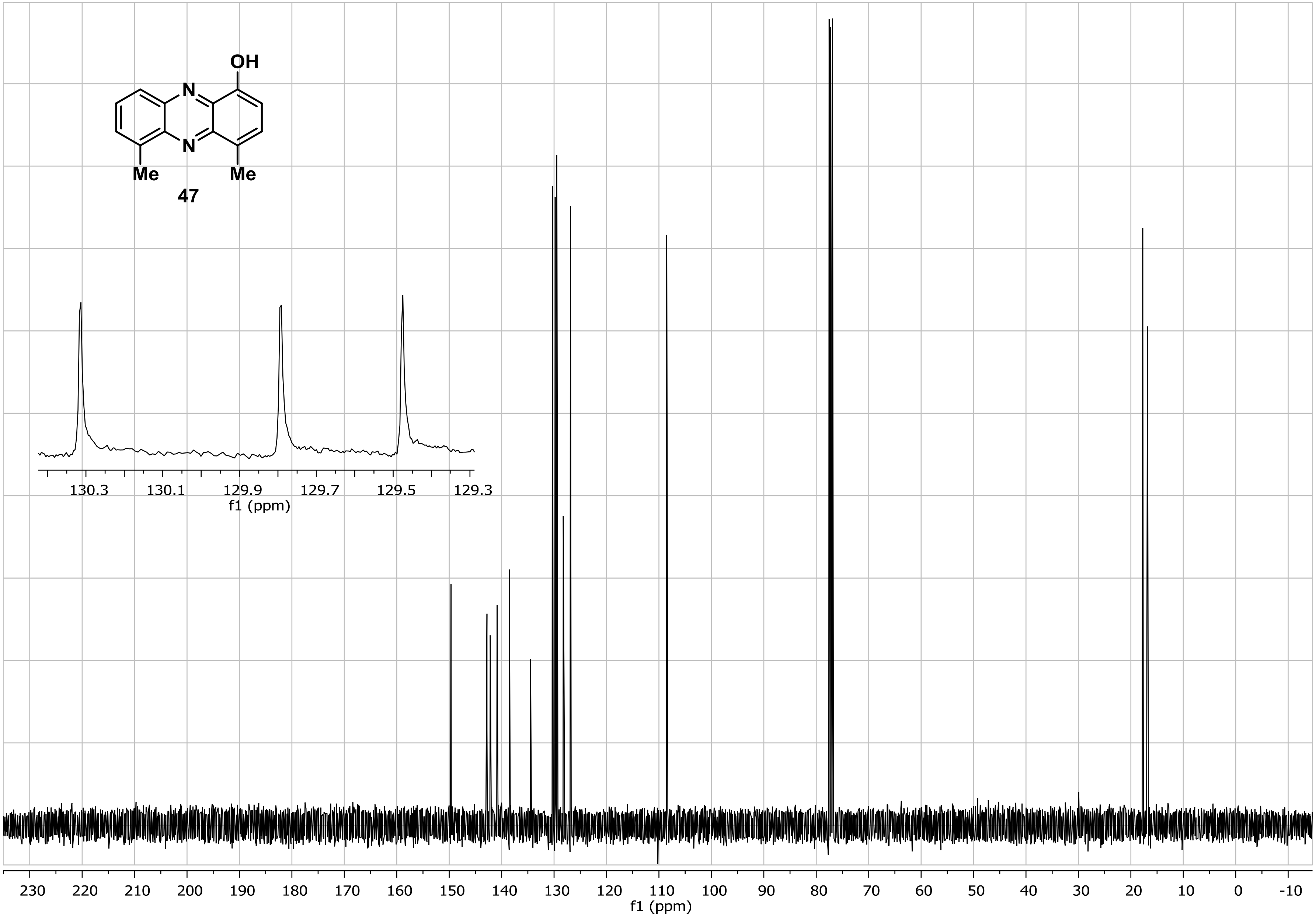

OH
N
N
Me
Me
47
130.3
130.1
129.9
129.7
129.5
129.3
f1 (ppm)
230
220
210
200
190
180
170
160
150
140
130
120
110
100
90
80
70
60
50
40
30
20
10
0
-10
f1 (ppm)

NMR-89

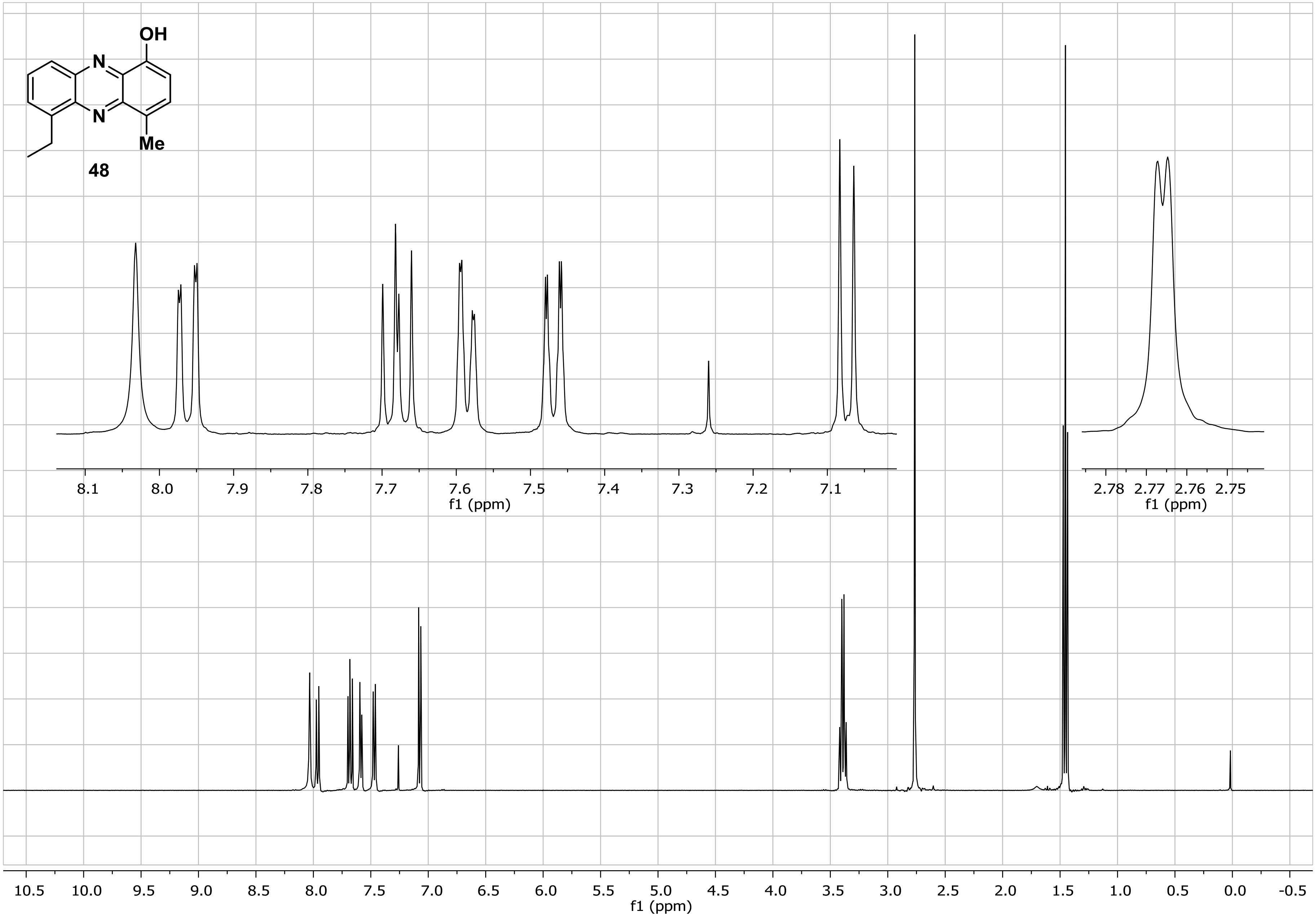

NMR-90

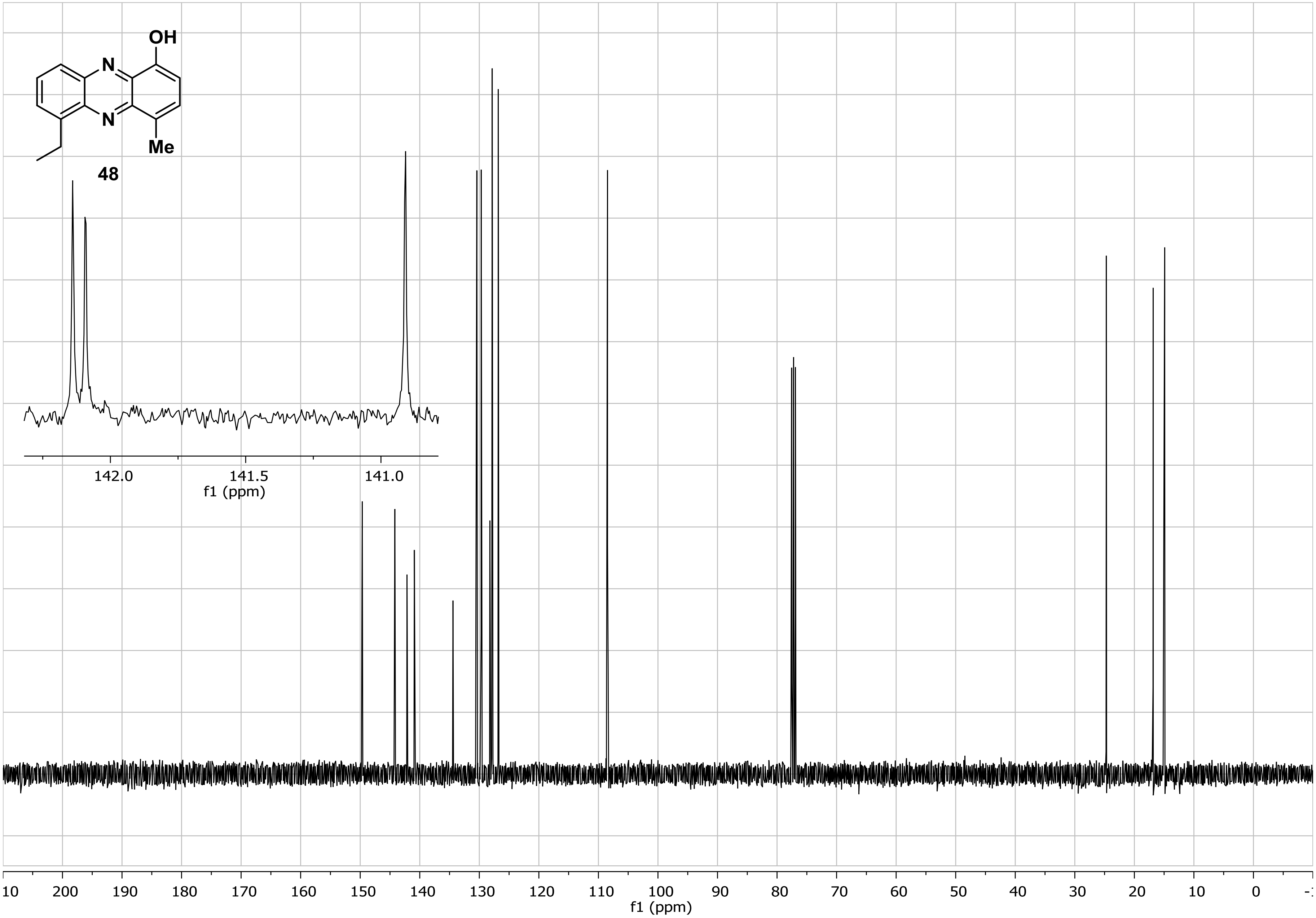
OH
N
N
Me
48
142.0
141.5
141.0
f1 (ppm)
200 190 180 170 160 150 140 130 120 110 100 90 80 70 60 50 40 30 20 10 0
f1 (ppm)

NMR-91

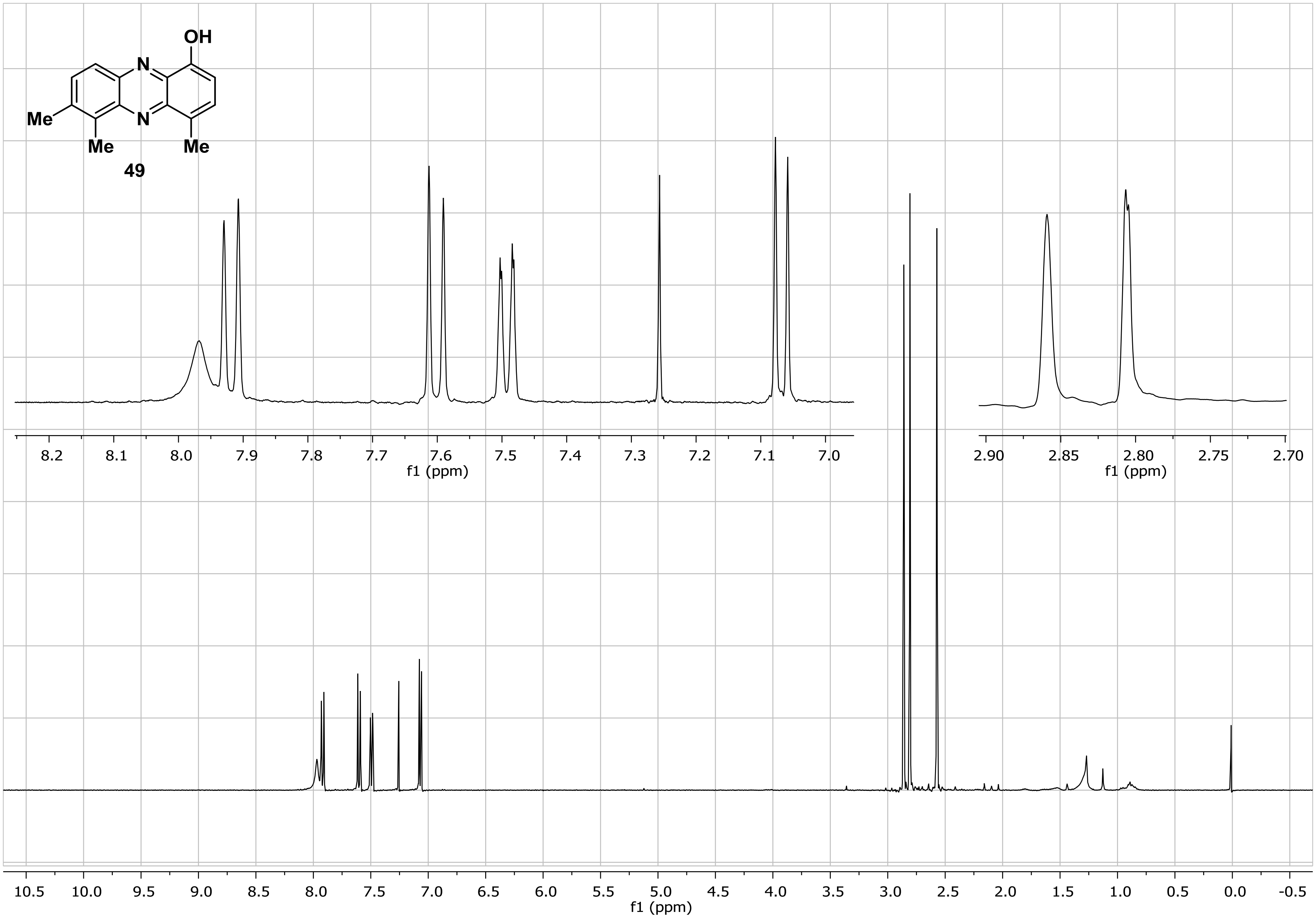
OH
N
N
Me
Me
Me
49
8.2
8.1
8.0
7.9
7.8
7.7
7.6
7.5
7.4
7.3
7.2
7.1
7.0
f1 (ppm)
2.90
2.85
2.80
2.75
2.70
f1 (ppm)
10.5
10.0
9.5
9.0
8.5
8.0
7.5
7.0
6.5
6.0
5.5
5.0
4.5
4.0
3.5
3.0
2.5
2.0
1.5
1.0
0.5
0.0
-0.5
f1 (ppm)

NMR-92

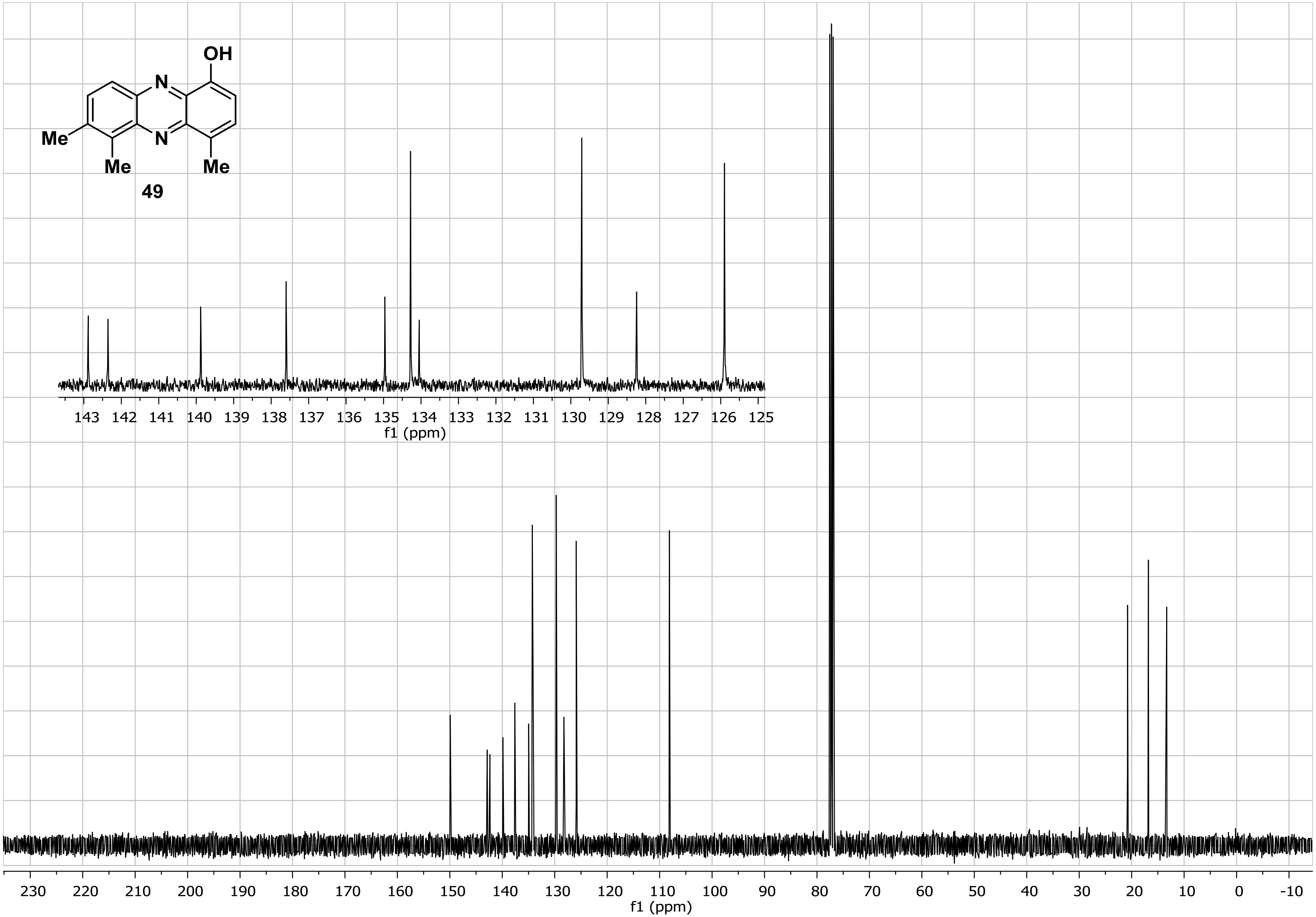

NMR-93

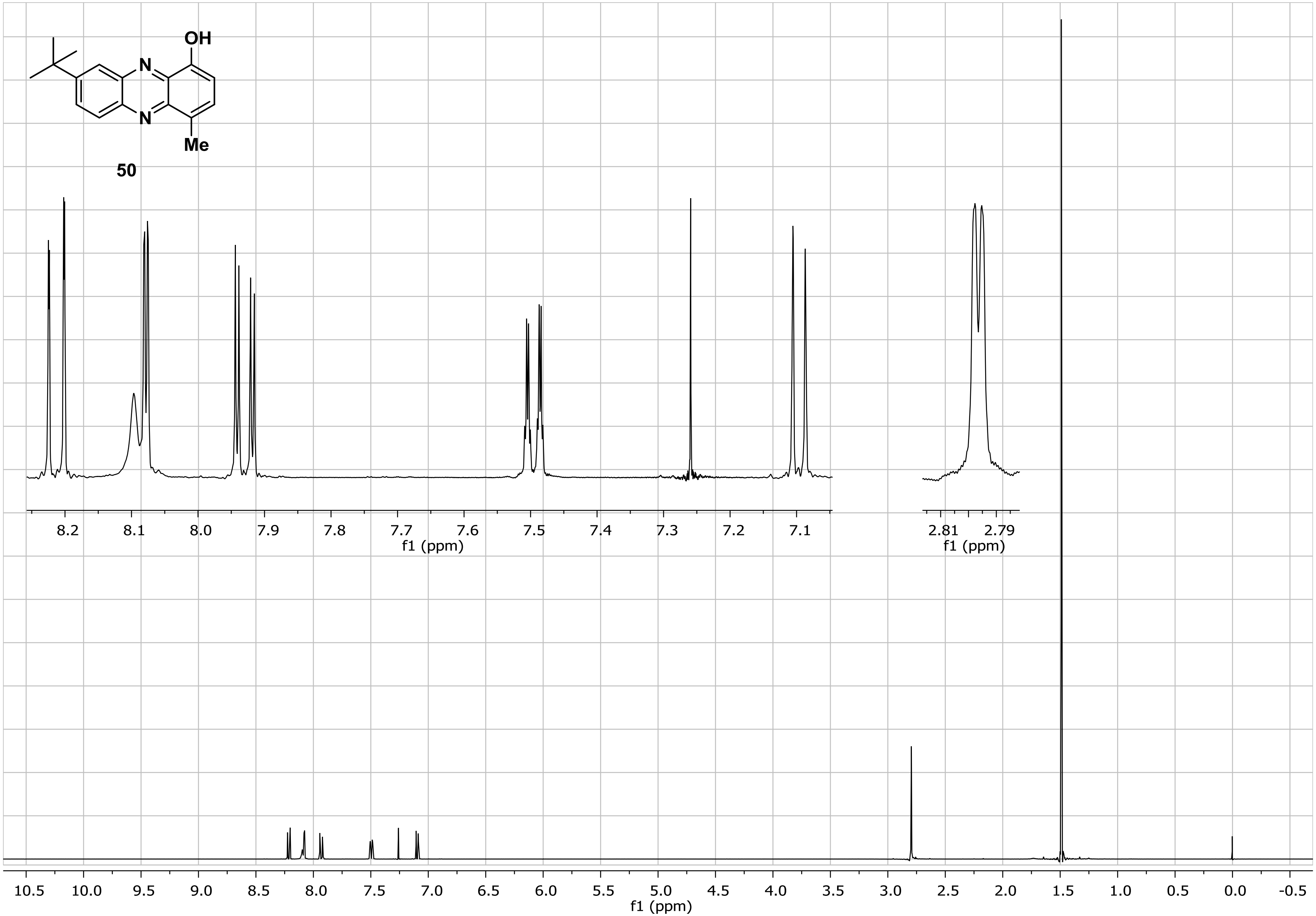
OH
N
N
Me
50
8.2
8.1
8.0
7.9
7.8
7.7
7.6
7.5
7.4
7.3
7.2
7.1
f1 (ppm)
2.81
2.79
f1 (ppm)
10.5
10.0
9.5
9.0
8.5
8.0
7.5
7.0
6.5
6.0
5.5
5.0
4.5
4.0
3.5
3.0
2.5
2.0
1.5
1.0
0.5
0.0
-0.5
f1 (ppm)

NMR-94

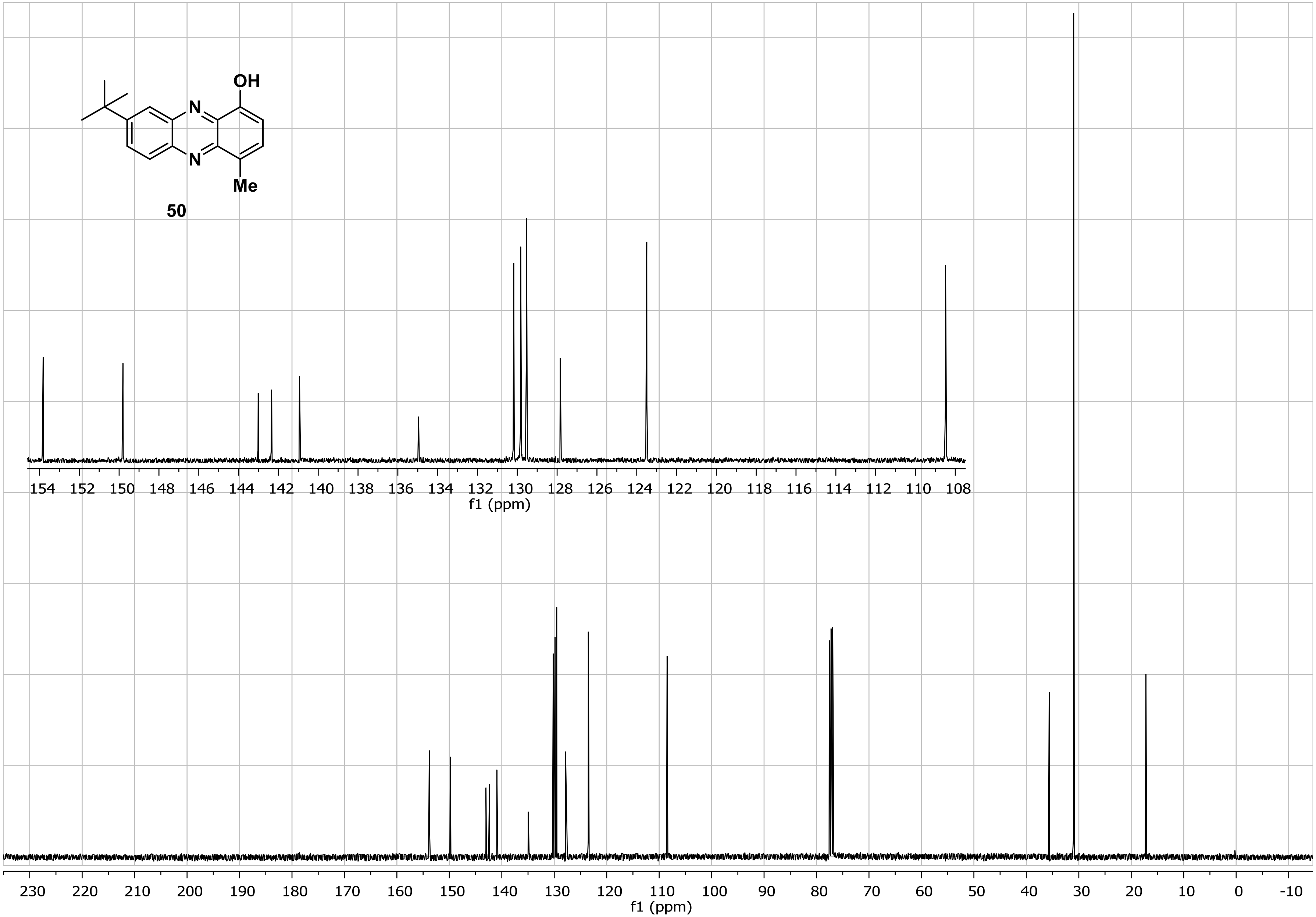

OH
N
N
Me
50
154 152 150 148 146 144 142 140 138 136 134 132 130 128 126 124 122 120 118 116 114 112 110 108
f1 (ppm)
230 220 210 200 190 180 170 160 150 140 130 120 110 100 90 80 70 60 50 40 30 20 10 0 -10
f1 (ppm)

NMR-95

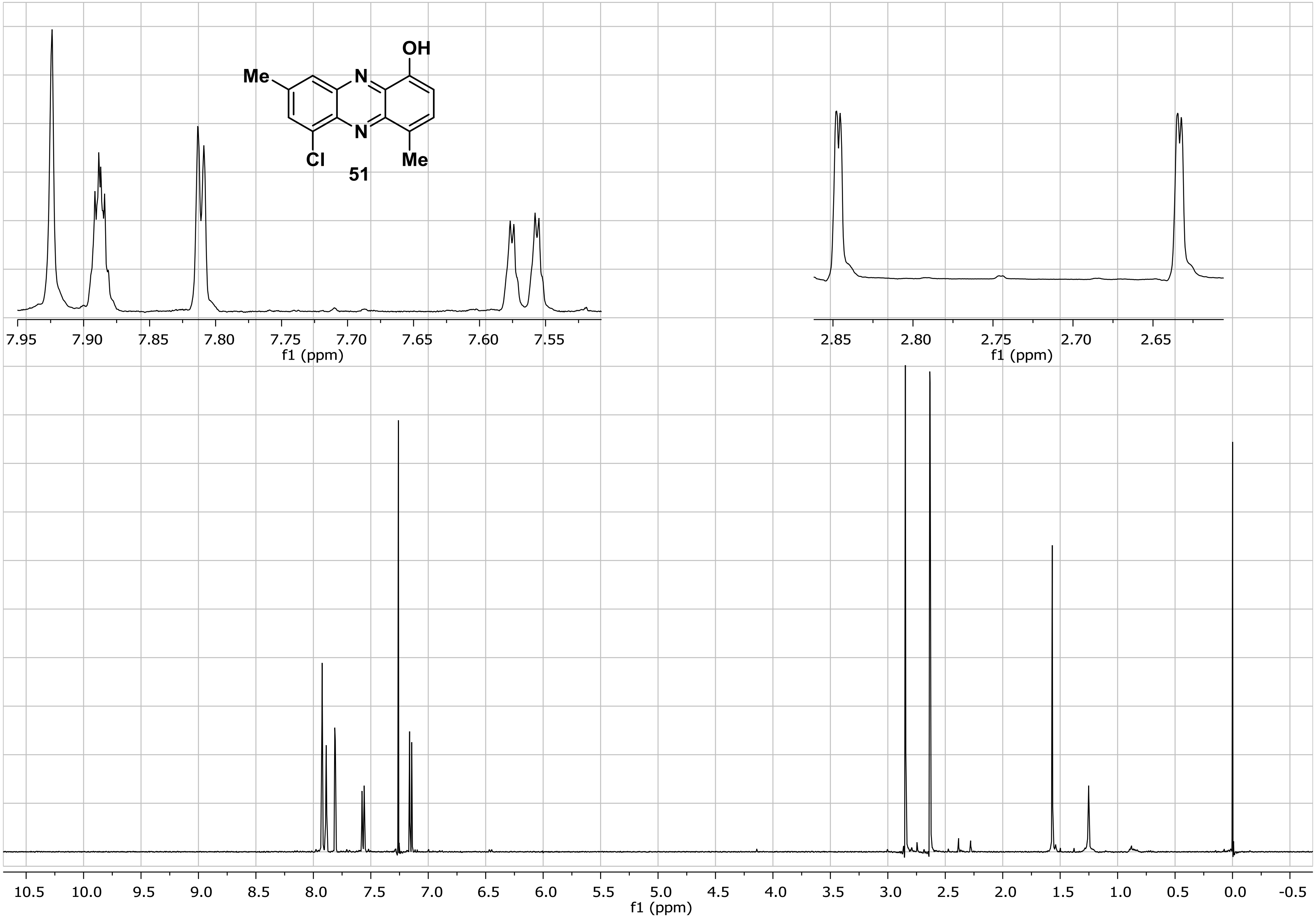

OH
Me
N
N
Cl
Me
51
7.95
7.90
7.85
7.80
7.75
7.70
7.65
7.60
7.55
f1 (ppm)
2.85
2.80
2.75
2.70
2.65
f1 (ppm)
10.5
10.0
9.5
9.0
8.5
8.0
7.5
7.0
6.5
6.0
5.5
5.0
4.5
4.0
3.5
3.0
2.5
2.0
1.5
1.0
0.5
0.0
-0.5
f1 (ppm)

NMR-96

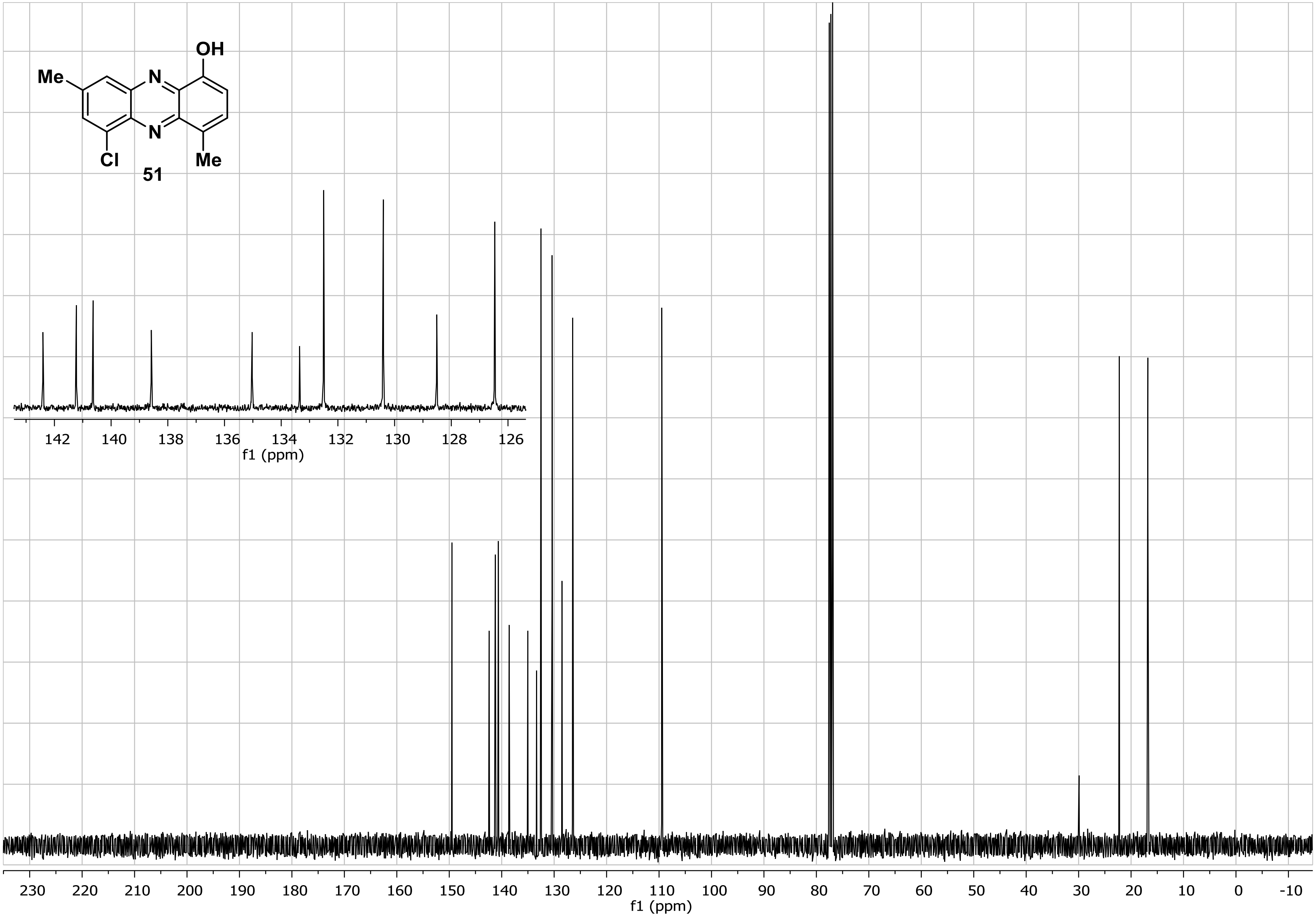
OH
Me
N
N
Cl
Me
51
142
140
138
136
134
132
130
128
126
f1 (ppm)
230
220
210
200
190
180
170
160
150
140
130
120
110
100
90
80
70
60
50
40
30
20
10
0
-10
f1 (ppm)

NMR-97

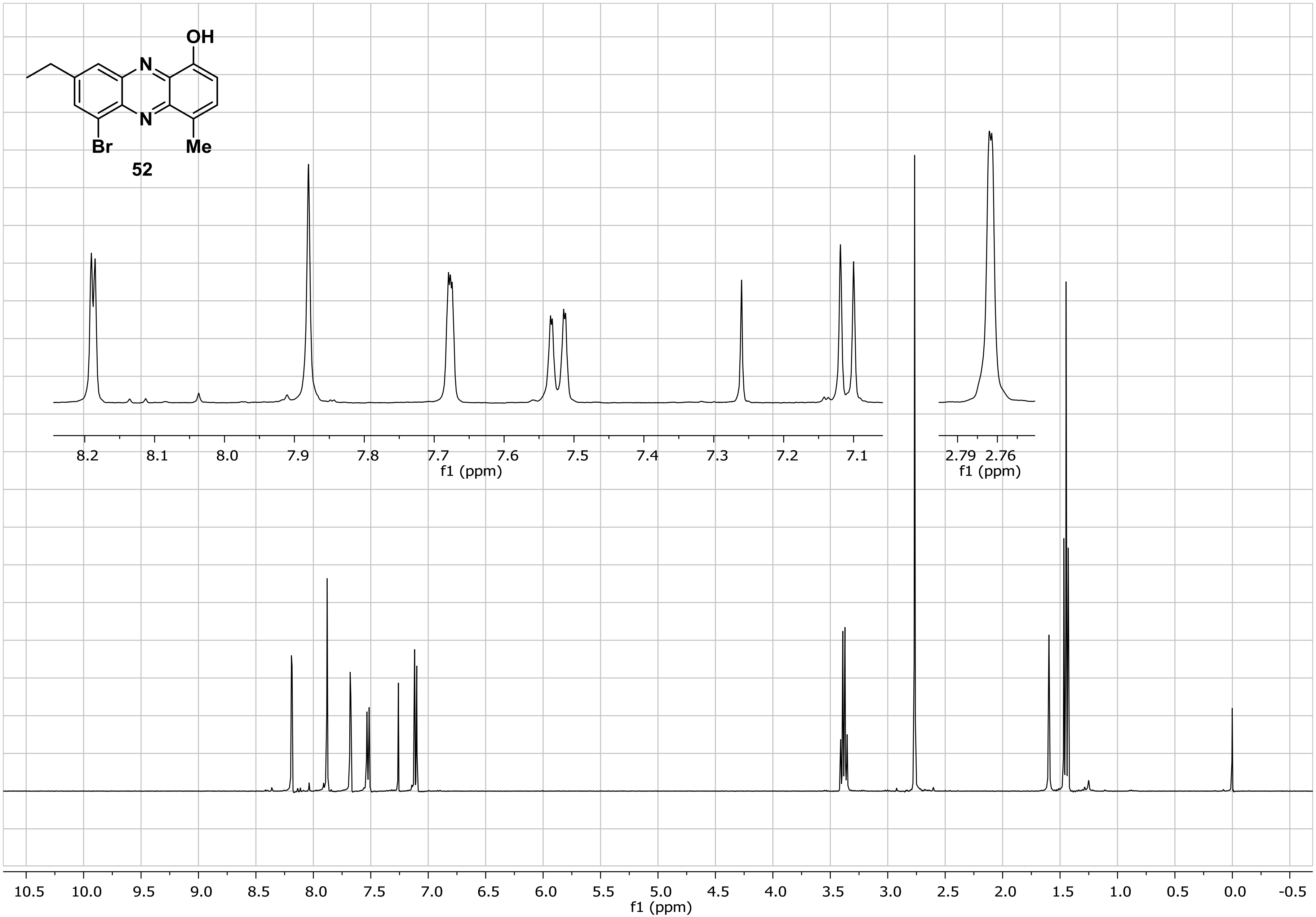
OH
N
N
Br
Me
52
8.2
8.1
8.0
7.9
7.8
7.7
7.6
7.5
7.4
7.3
7.2
7.1
f1 (ppm)
2.79 2.76
f1 (ppm)
10.5
10.0
9.5
9.0
8.5
8.0
7.5
7.0
6.5
6.0
5.5
5.0
4.5
4.0
3.5
3.0
2.5
2.0
1.5
1.0
0.5
0.0
-0.5
f1 (ppm)

NMR-98

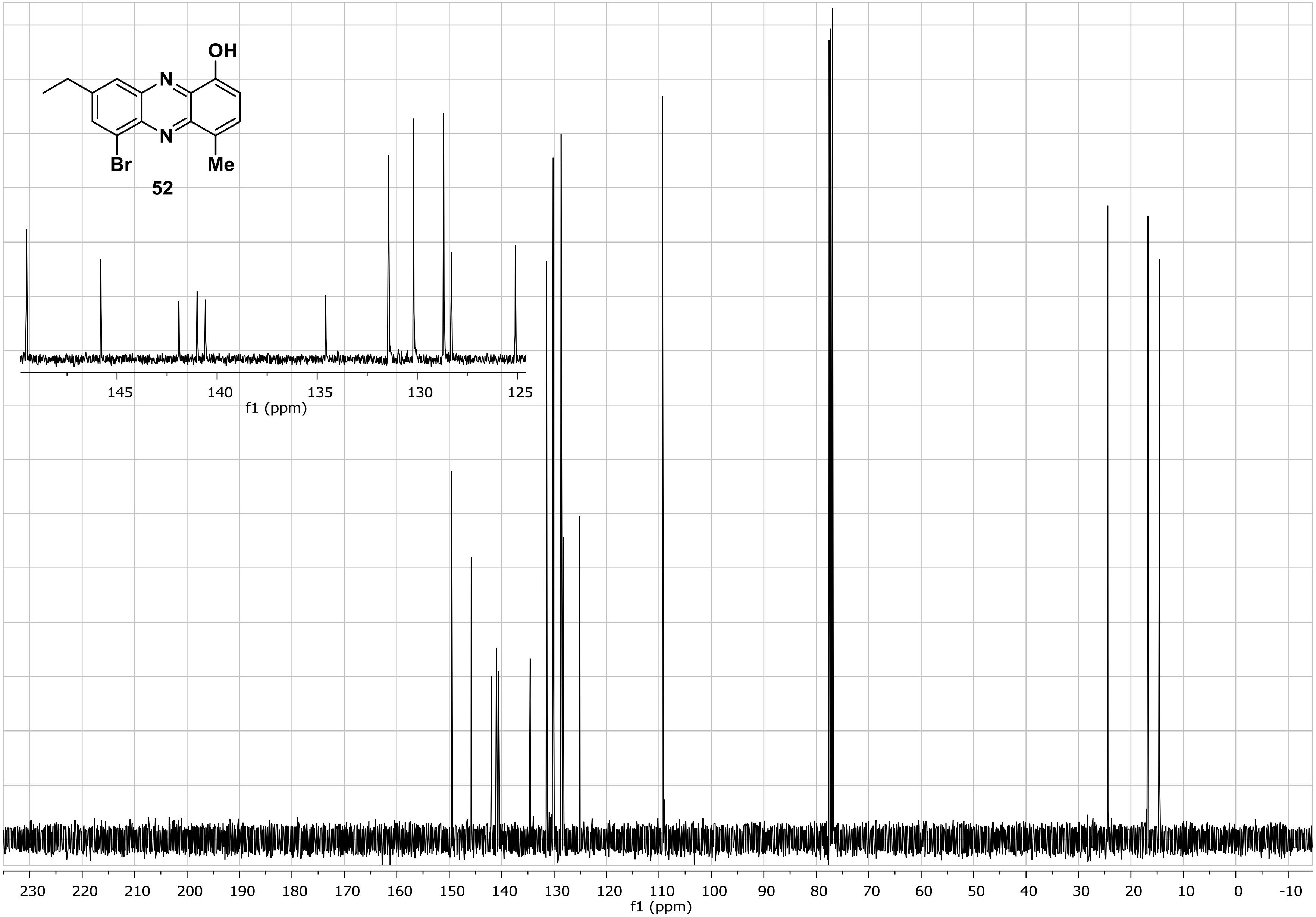
OH
N
N
Br
Me
52
145
140
135
130
125
f1 (ppm)
230
220
210
200
190
180
170
160
150
140
130
120
110
100
90
80
70
60
50
40
30
20
10
0
-10
f1 (ppm)

NMR-99

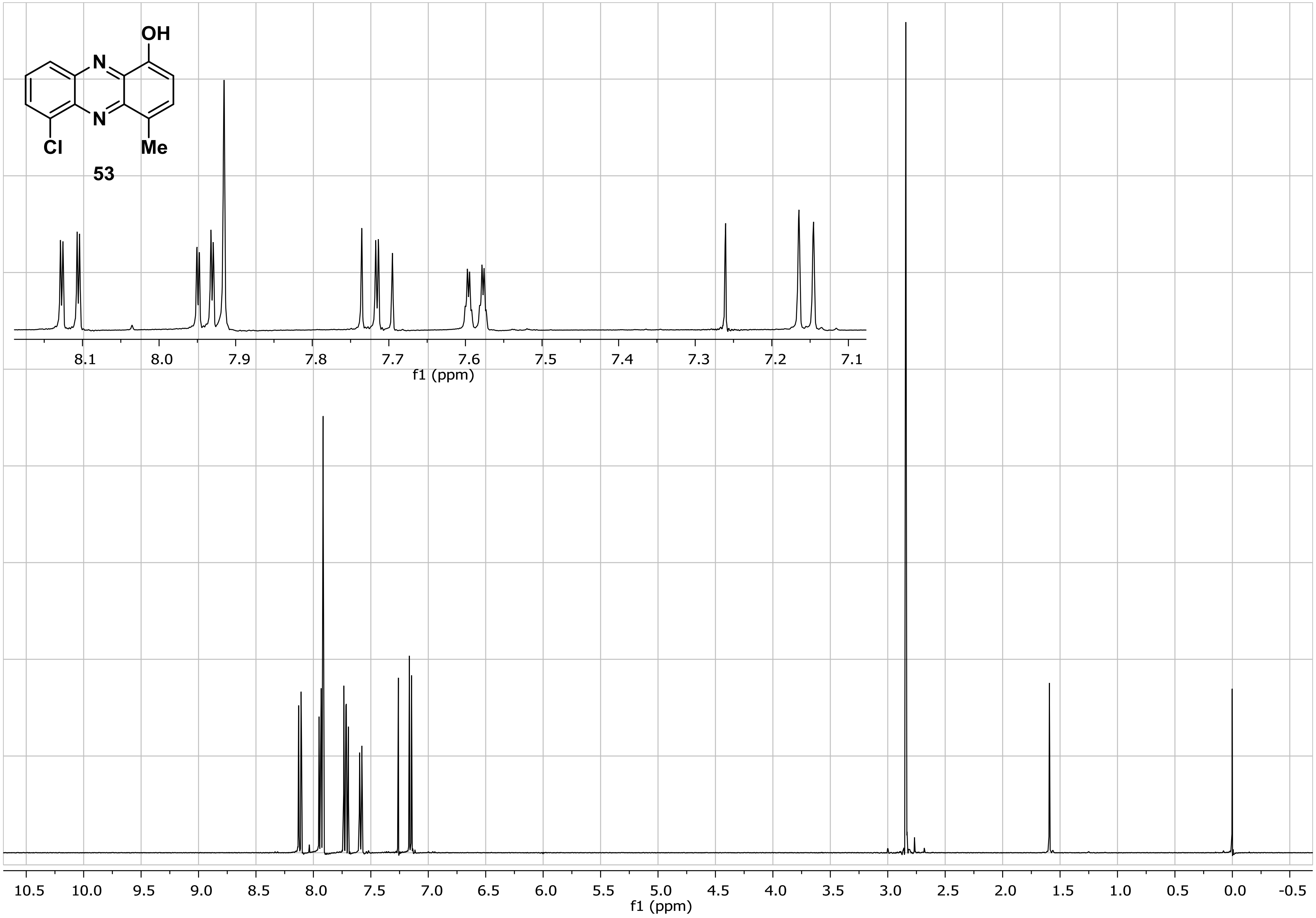
OH
N
N
Cl
Me
53
8.1 8.0 7.9 7.8 7.7 7.6 7.5 7.4 7.3 7.2 7.1
f1 (ppm)
10.5 10.0 9.5 9.0 8.5 8.0 7.5 7.0 6.5 6.0 5.5 5.0 4.5 4.0 3.5 3.0 2.5 2.0 1.5 1.0 0.5 0.0 -0.5
f1 (ppm)

NMR-100

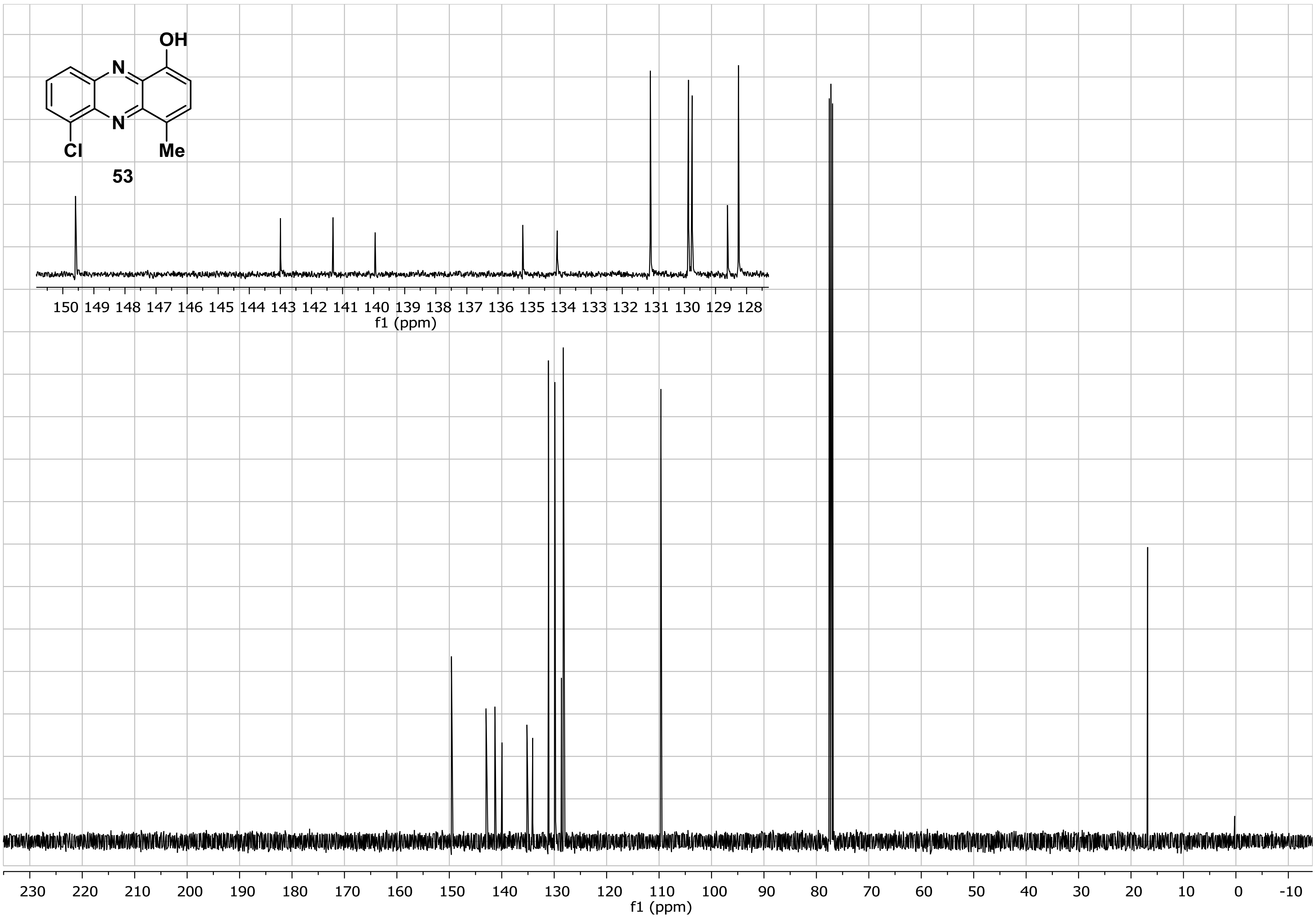
OH
N
N
Cl
Me
53
150 149 148 147 146 145 144 143 142 141 140 139 138 137 136 135 134 133 132 131 130 129 128
f1 (ppm)
230 220 210 200 190 180 170 160 150 140 130 120 110 100 90 80 70 60 50 40 30 20 10 0 -10
f1 (ppm)

NMR-101

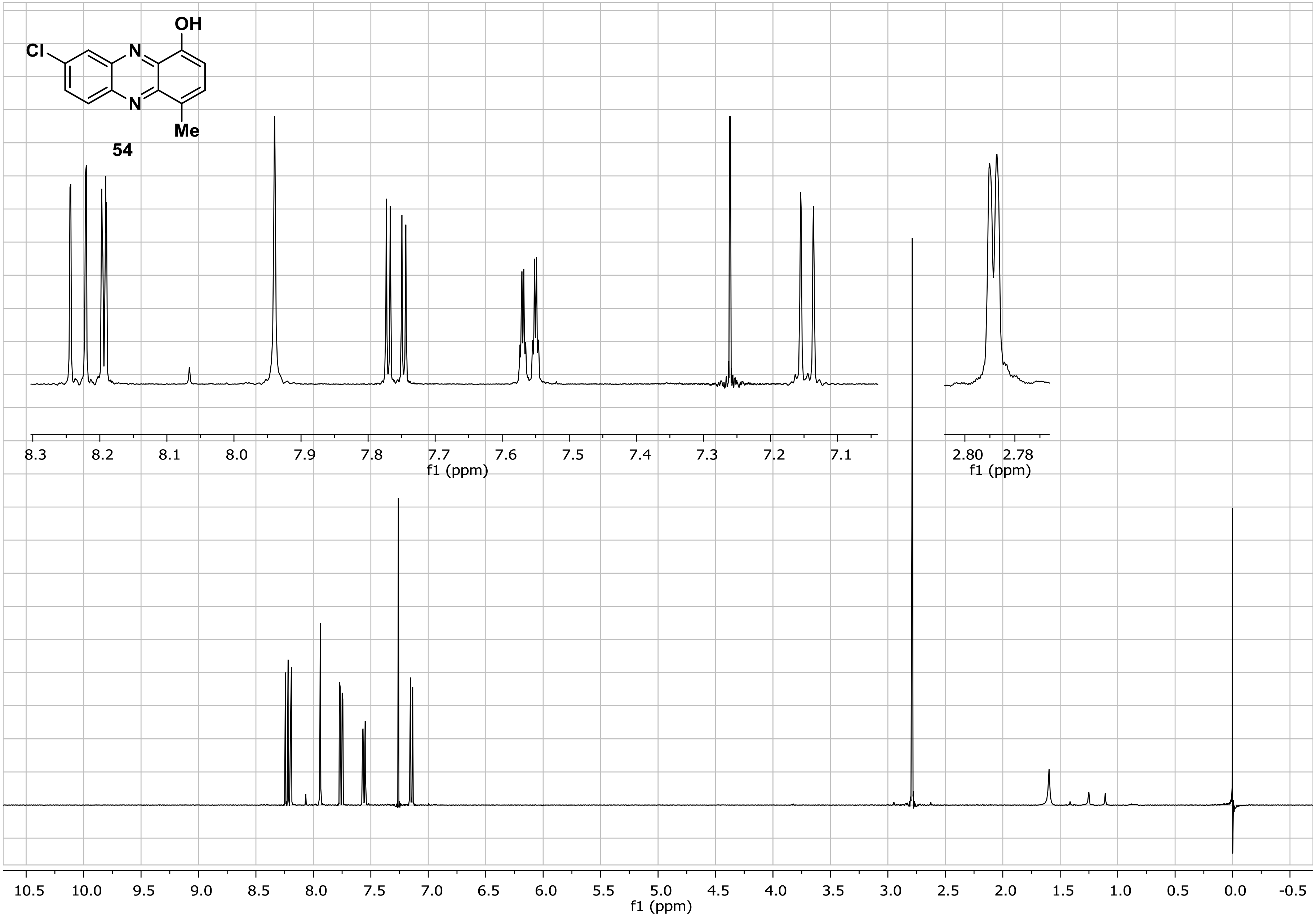
OH
Cl
N
N
Me
54
8.3 8.2 8.1 8.0 7.9 7.8 7.7 7.6 7.5 7.4 7.3 7.2 7.1
f1 (ppm)
2.80 2.78
f1 (ppm)
10.5 10.0 9.5 9.0 8.5 8.0 7.5 7.0 6.5 6.0 5.5 5.0 4.5 4.0 3.5 3.0 2.5 2.0 1.5 1.0 0.5 0.0 -0.5
f1 (ppm)

NMR-102

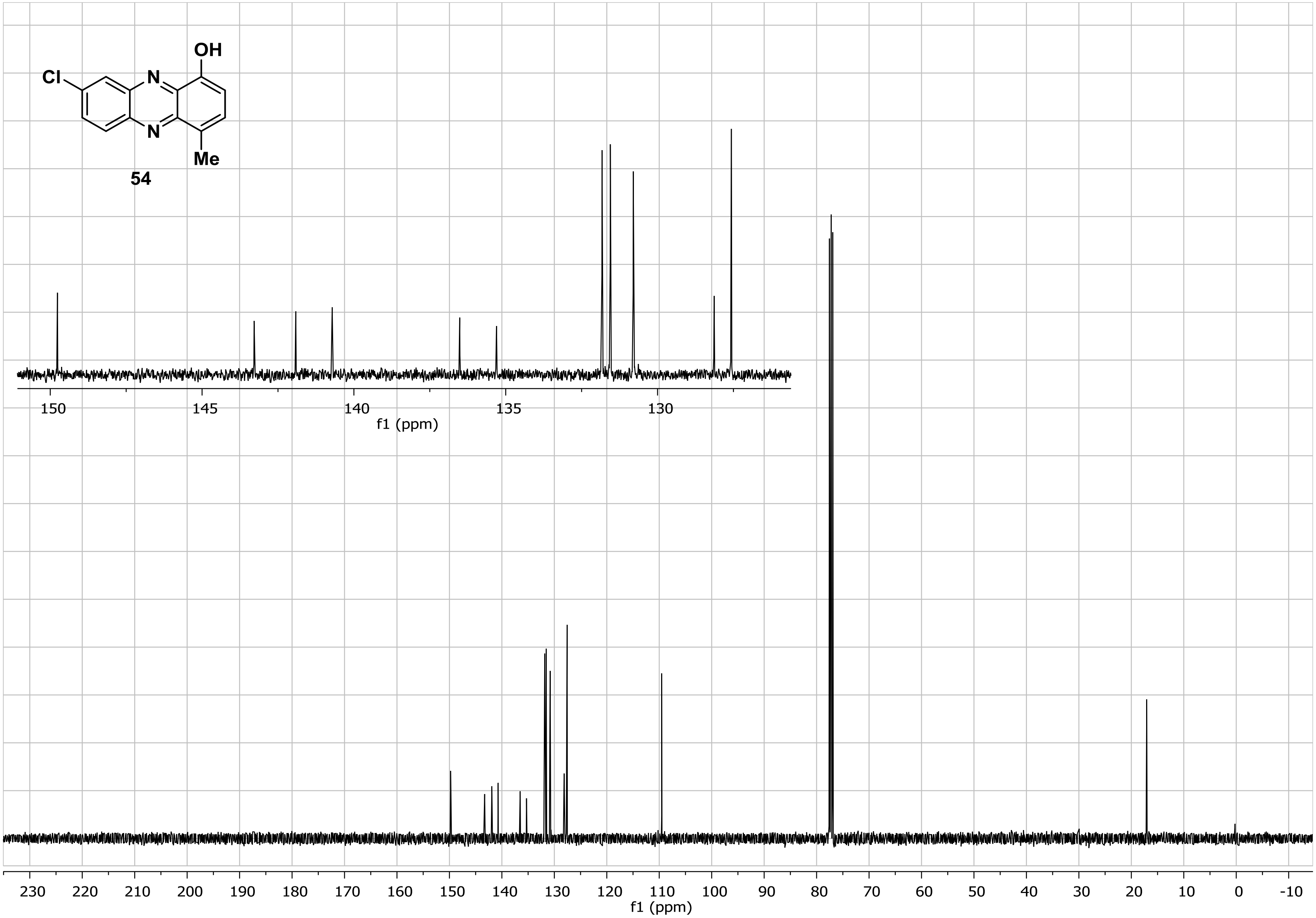

NMR-103

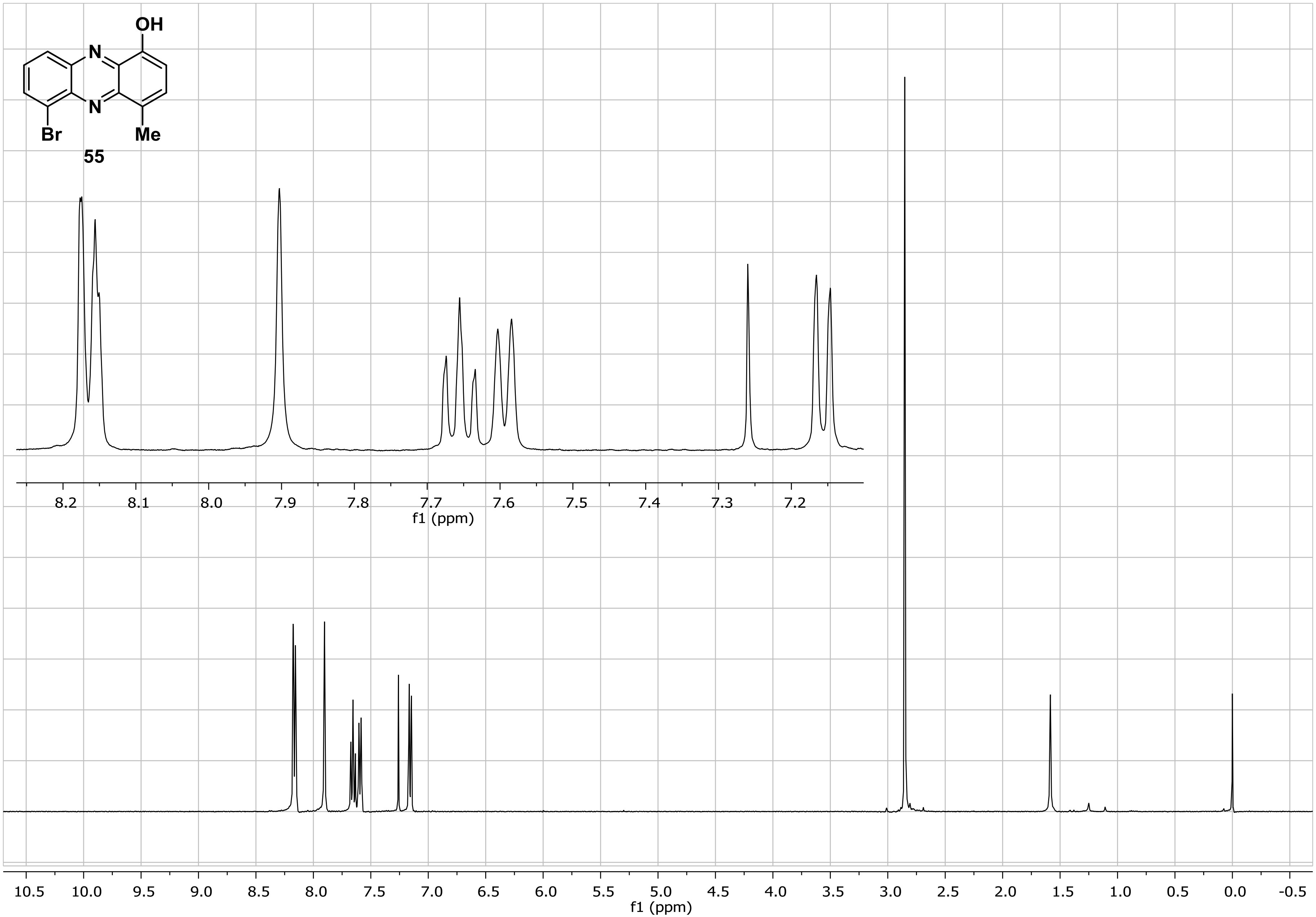

NMR-104

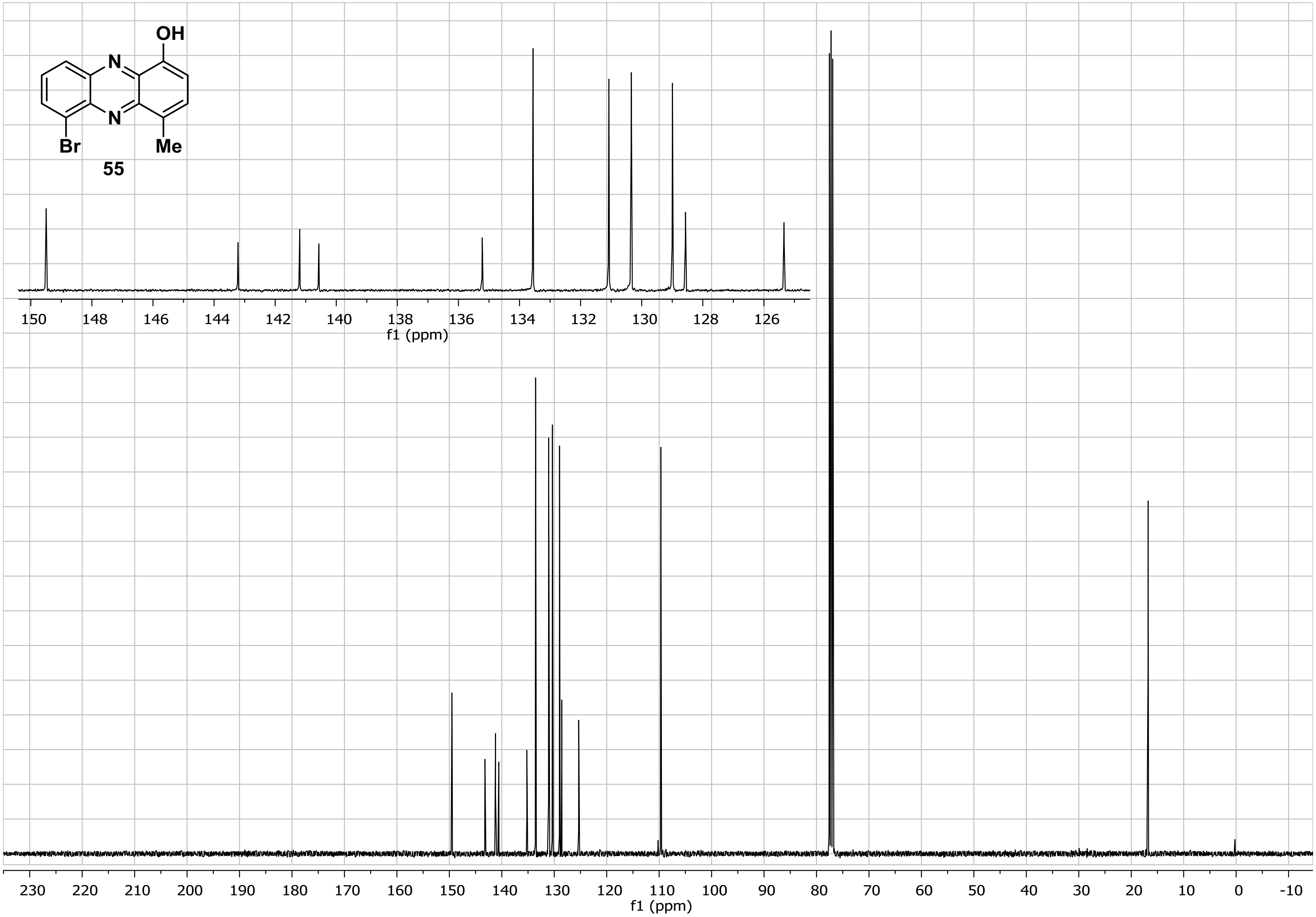
OH
N
N
Br
Me
55
150 148 146 144 142 140 138 136 134 132 130 128 126
f1 (ppm)
230 220 210 200 190 180 170 160 150 140 130 120 110 100 90 80 70 60 50 40 30 20 10 0 -10
f1 (ppm)

NMR-105

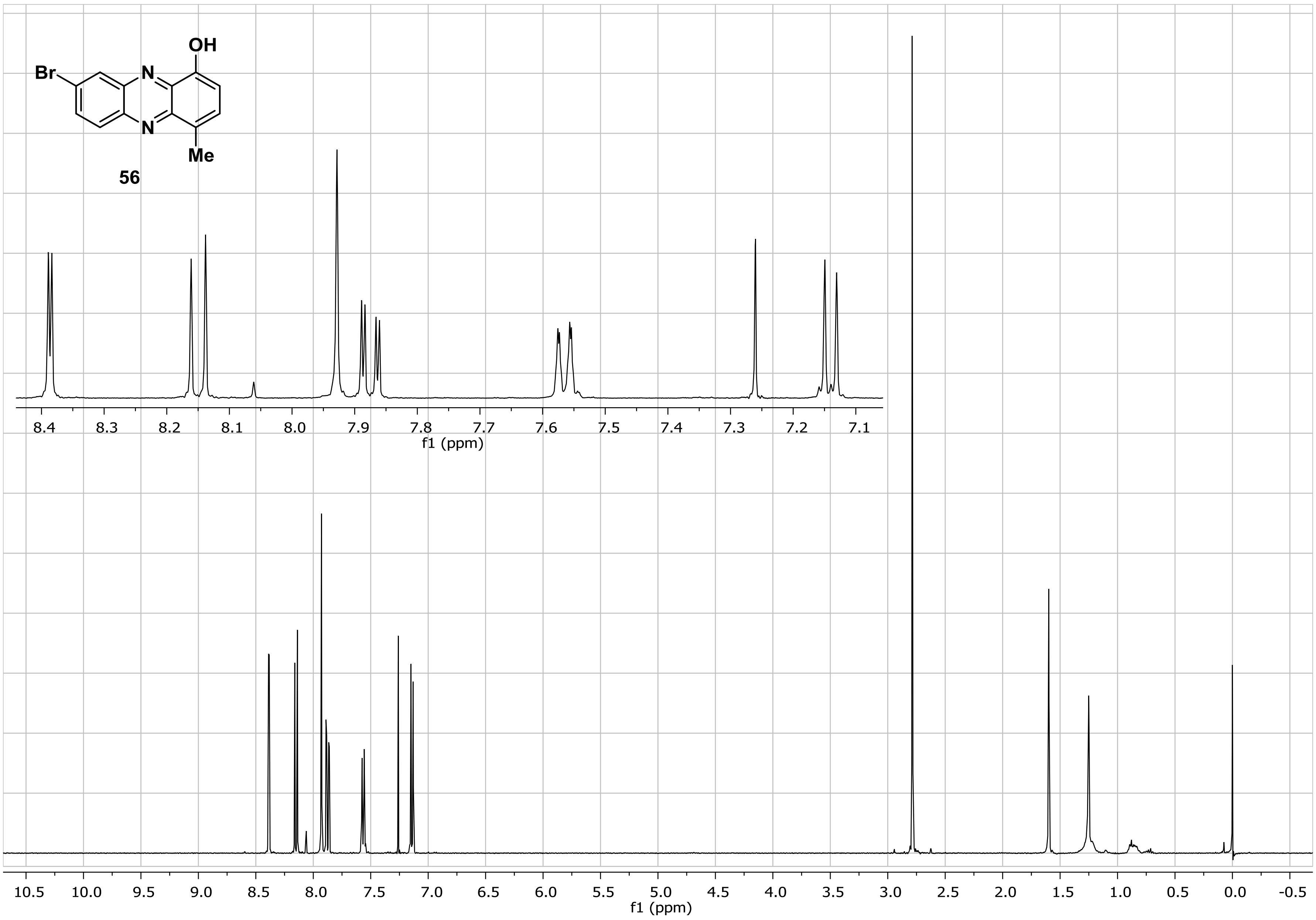

NMR-106

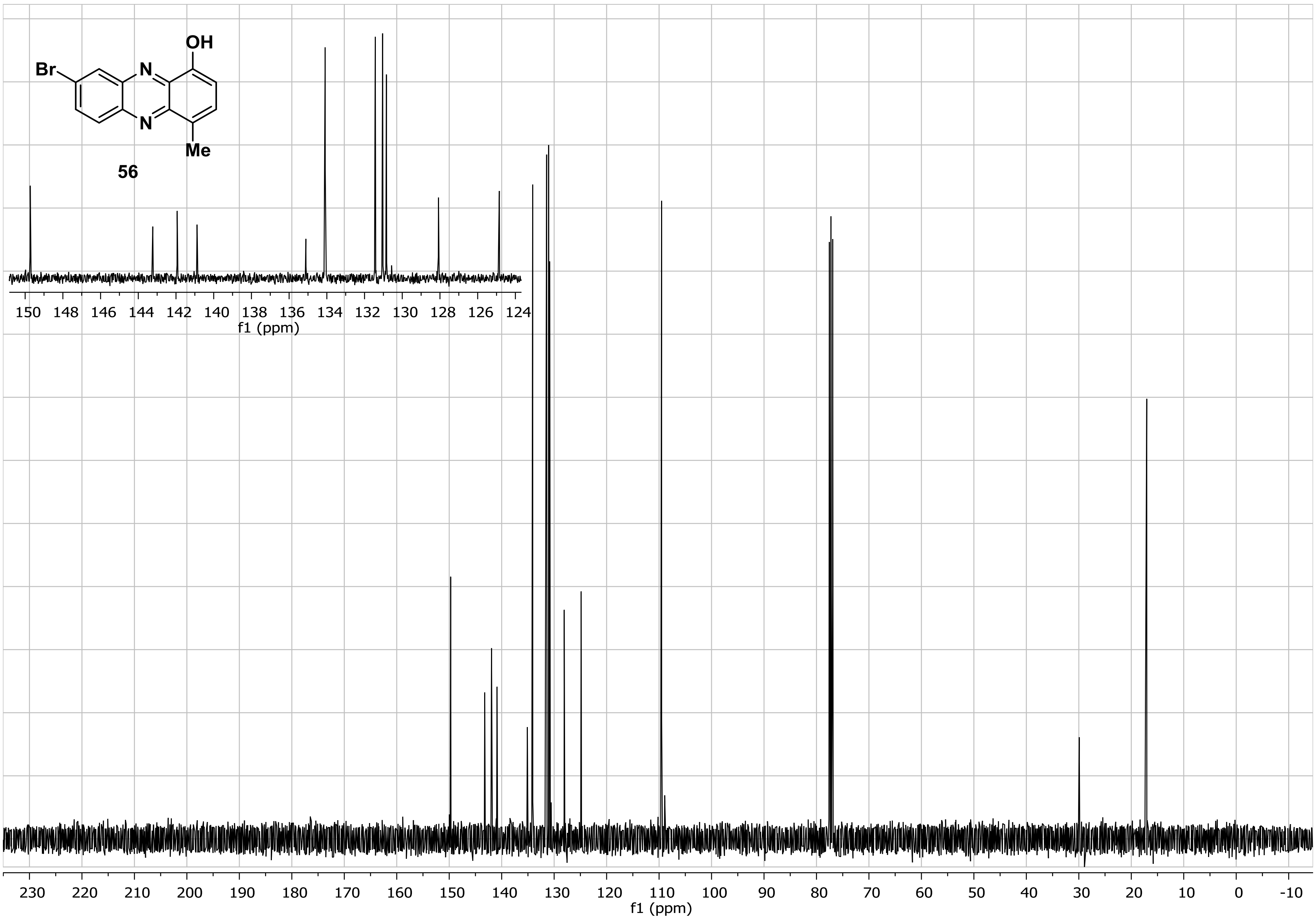

OH
Br
N
N
Me
56
150 148 146 144 142 140 138 136 134 132 130 128 126 124
f1 (ppm)
230 220 210 200 190 180 170 160 150 140 130 120 110 100 90 80 70 60 50 40 30 20 10 0 -10
f1 (ppm)

NMR-107

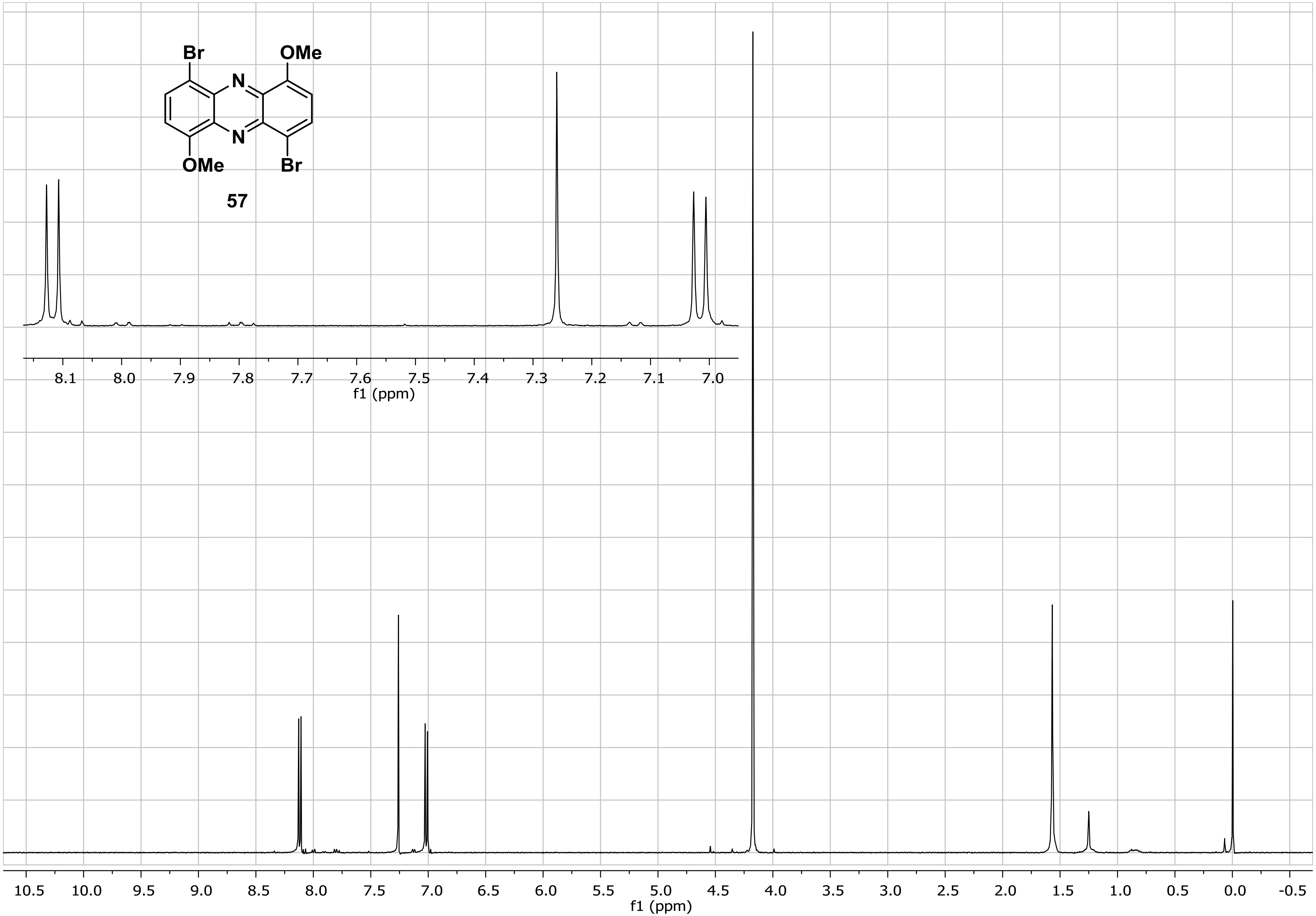

Br
OMe
N
N
OMe
Br
57
8.1
8.0
7.9
7.8
7.7
7.6
7.5
7.4
7.3
7.2
7.1
7.0
f1 (ppm)
10.5
10.0
9.5
9.0
8.5
8.0
7.5
7.0
6.5
6.0
5.5
5.0
4.5
4.0
3.5
3.0
2.5
2.0
1.5
1.0
0.5
0.0
-0.5
f1 (ppm)

NMR-108

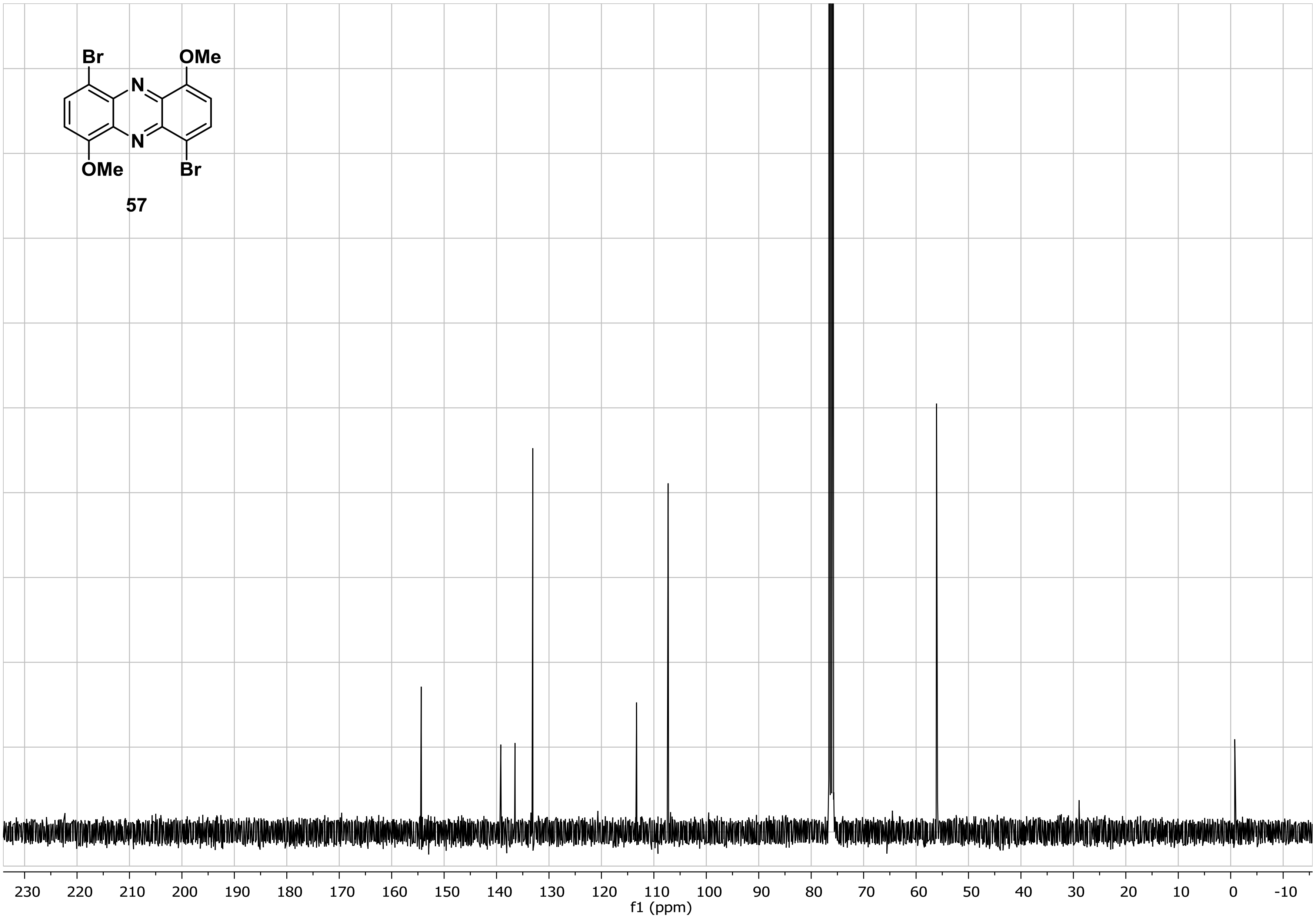
Br
OMe
N
N
OMe
Br
57
230 220 210 200 190 180 170 160 150 140 130 120 110 100 90 80 70 60 50 40 30 20 10 0 -10
f1 (ppm)

NMR-109

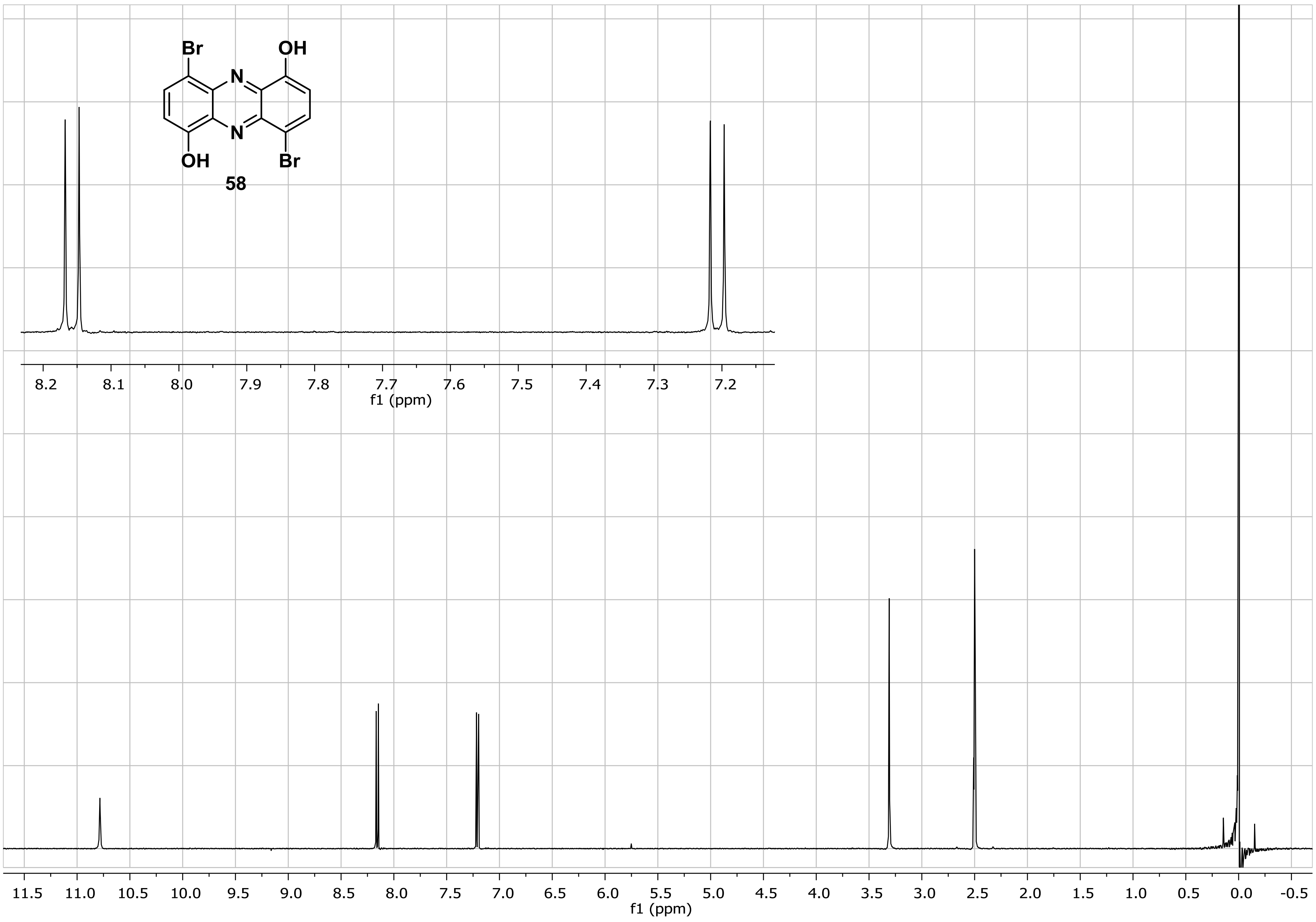

Br
OH
N
N
OH
Br
58
8.2
8.1
8.0
7.9
7.8
7.7
7.6
7.5
7.4
7.3
7.2
f1 (ppm)
11.5
11.0
10.5
10.0
9.5
9.0
8.5
8.0
7.5
7.0
6.5
6.0
5.5
5.0
4.5
4.0
3.5
3.0
2.5
2.0
1.5
1.0
0.5
0.0
-0.5
f1 (ppm)

NMR-110

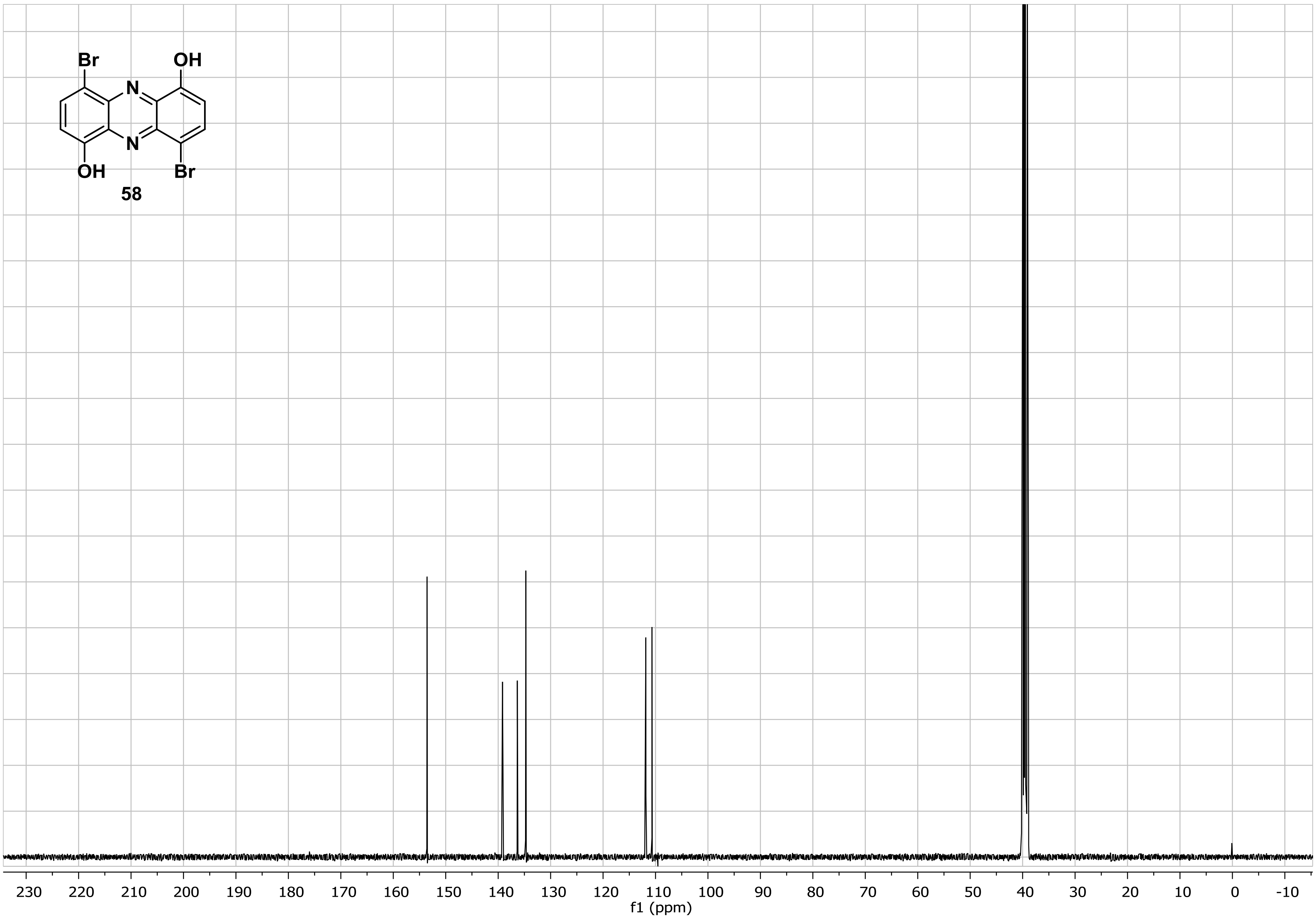
Br
OH
N
N
OH
Br
58
230 220 210 200 190 180 170 160 150 140 130 120 110 100 90 80 70 60 50 40 30 20 10 0 -10
f1 (ppm)

NMR-111

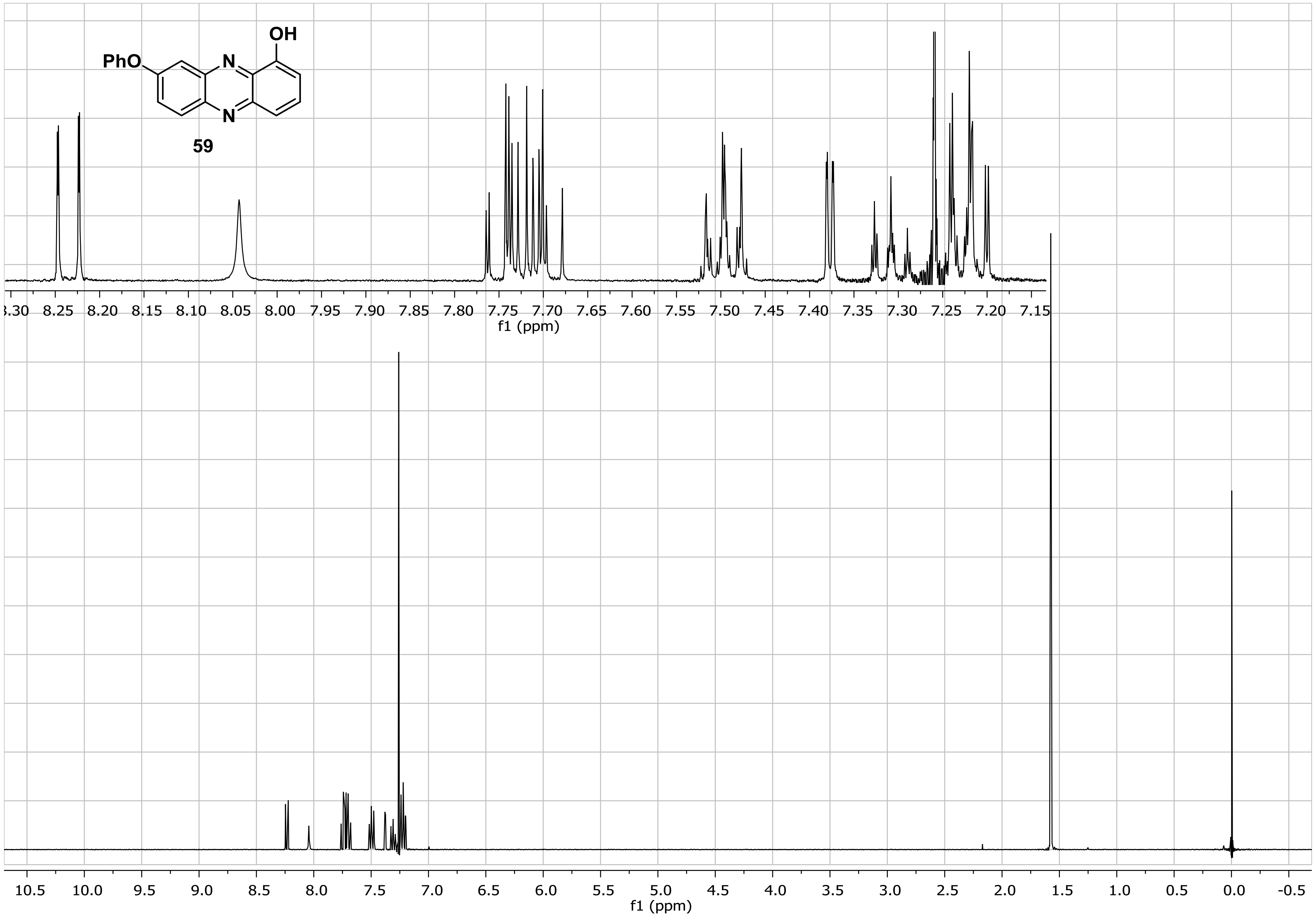

NMR-112

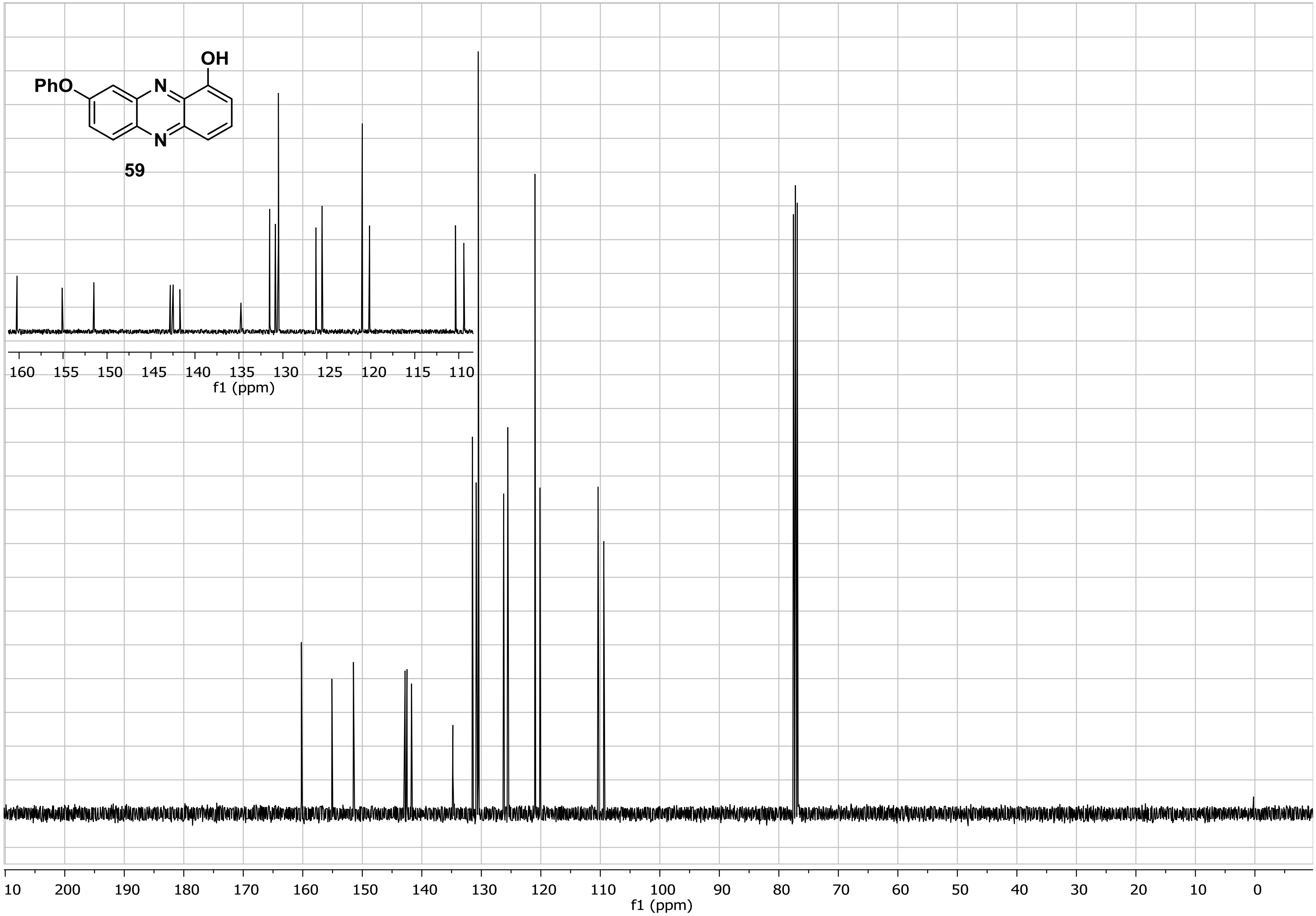
OH
PhO
N
N
59
160 155 150 145 140 135 130 125 120 115 110
f1 (ppm)
10 200 190 180 170 160 150 140 130 120 110 100 90 80 70 60 50 40 30 20 10 0
f1 (ppm)

NMR-113

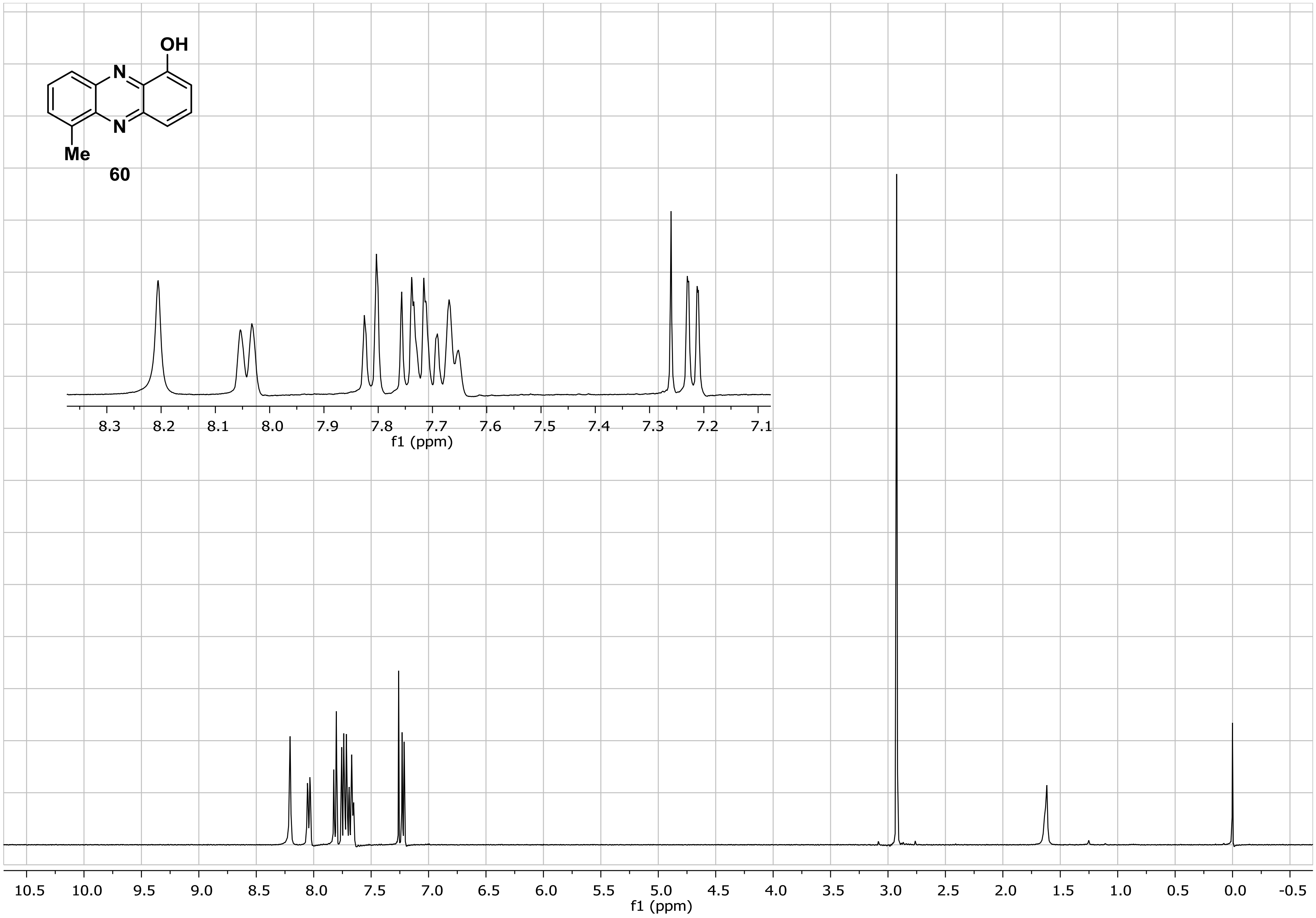
OH
N
N
Me
60
8.3 8.2 8.1 8.0 7.9 7.8 7.7 7.6 7.5 7.4 7.3 7.2 7.1
f1 (ppm)
10.5 10.0 9.5 9.0 8.5 8.0 7.5 7.0 6.5 6.0 5.5 5.0 4.5 4.0 3.5 3.0 2.5 2.0 1.5 1.0 0.5 0.0 -0.5
f1 (ppm)

NMR-114

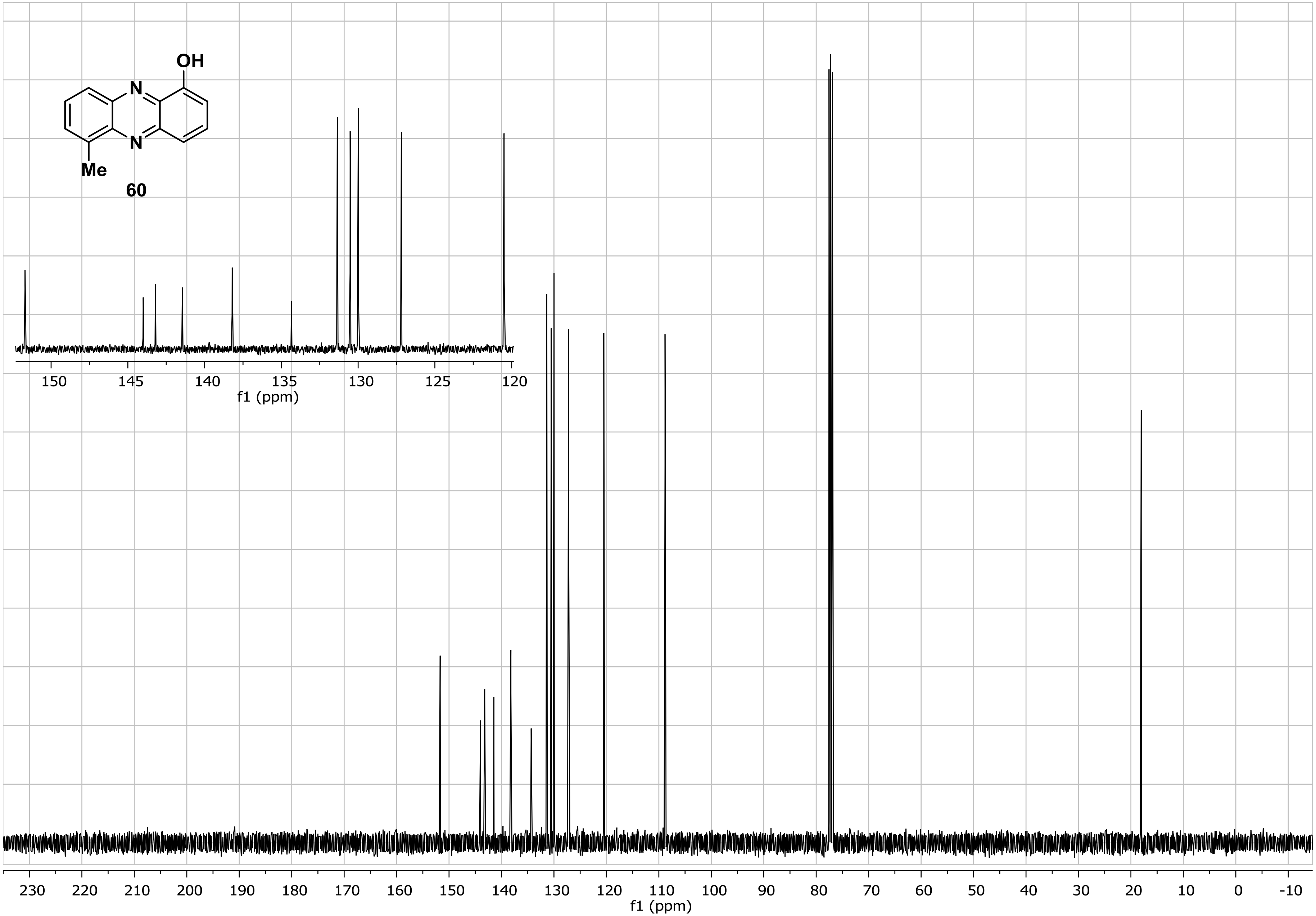
OH
N
N
Me
60
150 145 140 135 130 125 120
f1 (ppm)
230 220 210 200 190 180 170 160 150 140 130 120 110 100 90 80 70 60 50 40 30 20 10 0 -10
f1 (ppm)

NMR-115

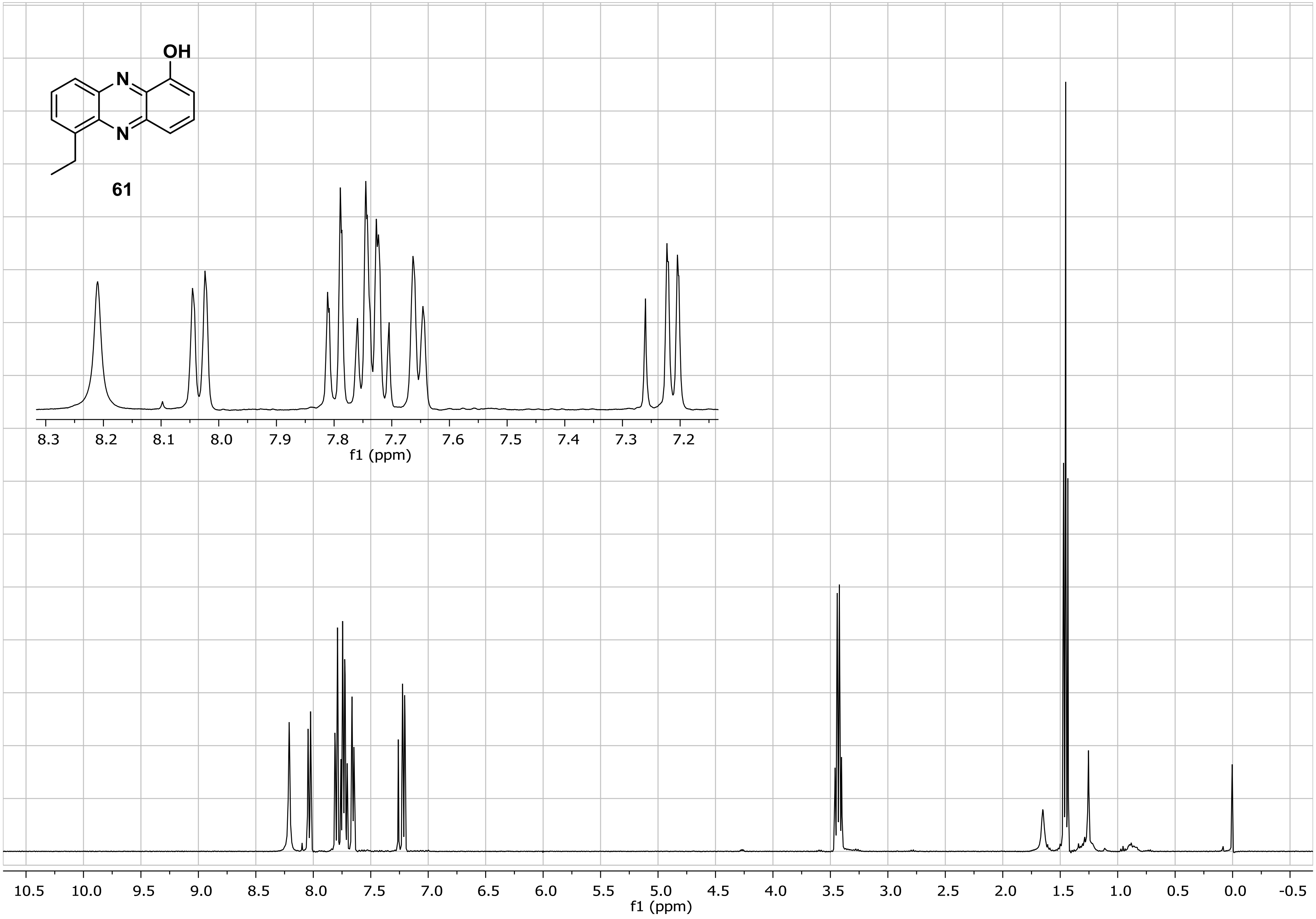
OH
N
N
61
8.3 8.2 8.1 8.0 7.9 7.8 7.7 7.6 7.5 7.4 7.3 7.2
f1 (ppm)
10.5 10.0 9.5 9.0 8.5 8.0 7.5 7.0 6.5 6.0 5.5 5.0 4.5 4.0 3.5 3.0 2.5 2.0 1.5 1.0 0.5 0.0 -0.5
f1 (ppm)

NMR-116

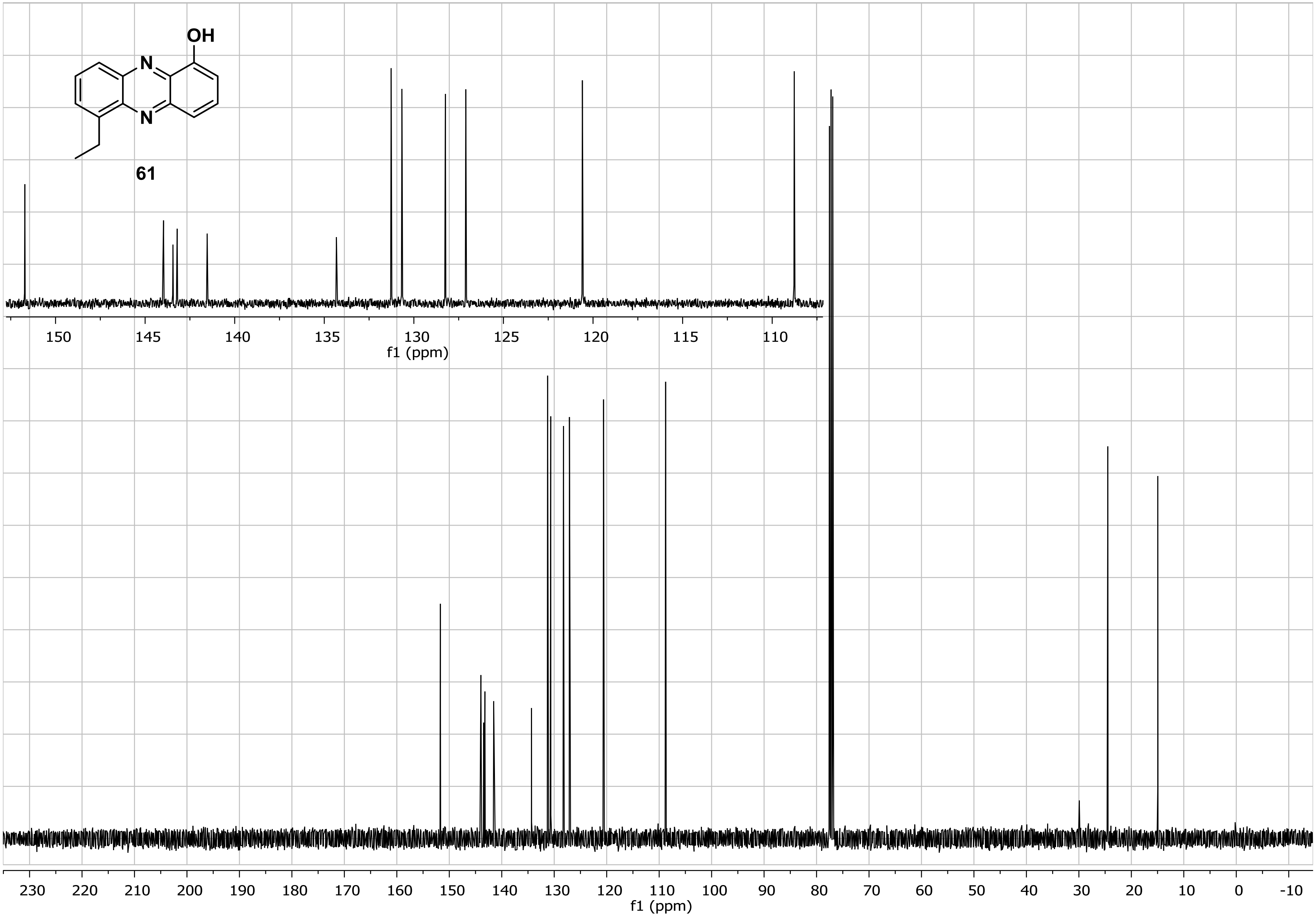

OH
N
N
61
150 145 140 135 130 125 120 115 110
f1 (ppm)
230 220 210 200 190 180 170 160 150 140 130 120 110 100 90 80 70 60 50 40 30 20 10 0 -10
f1 (ppm)

NMR-117

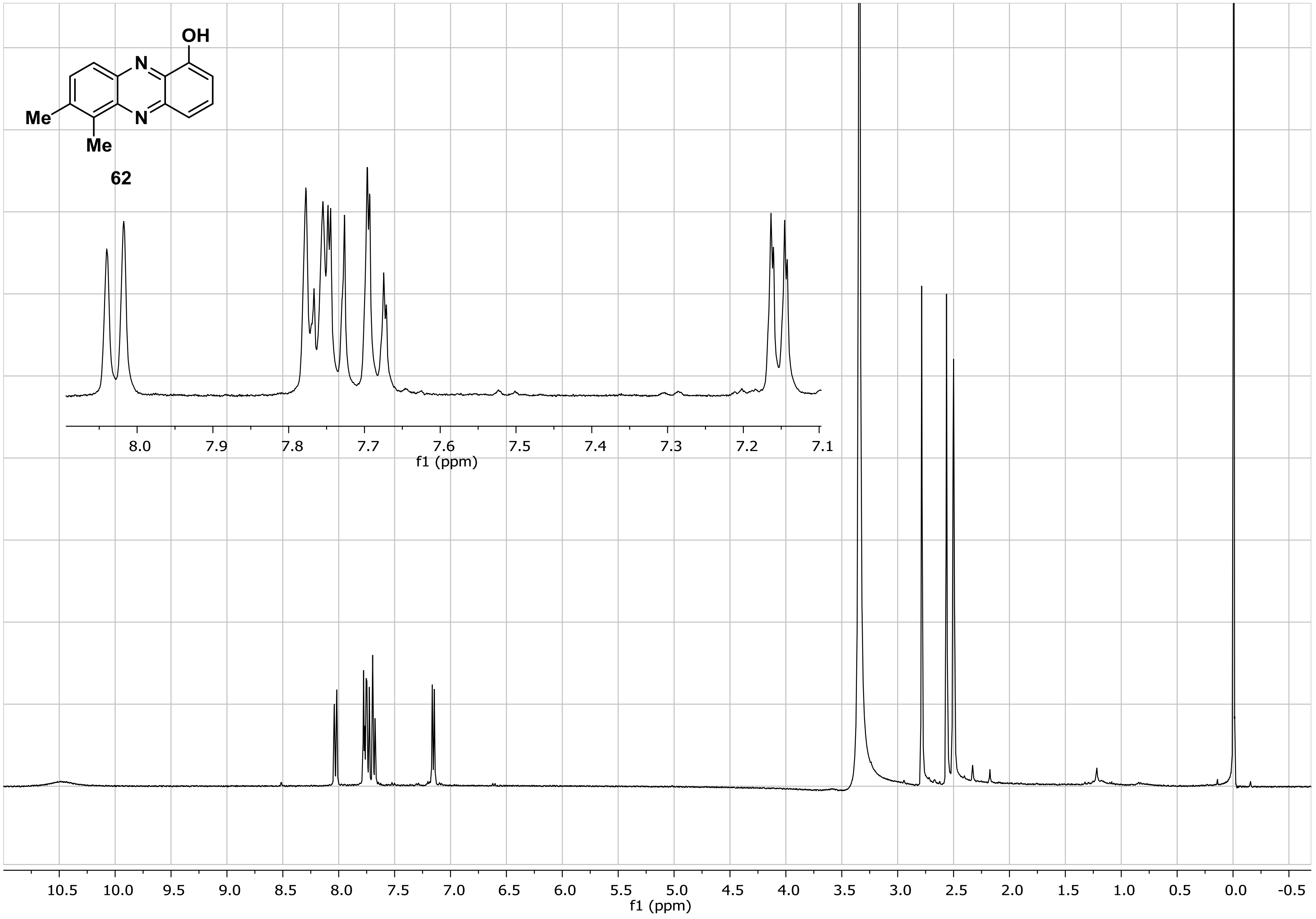
OH
N
N
Me
Me
62
8.0
7.9
7.8
7.7
7.6
7.5
7.4
7.3
7.2
7.1
f1 (ppm)
10.5
10.0
9.5
9.0
8.5
8.0
7.5
7.0
6.5
6.0
5.5
5.0
4.5
4.0
3.5
3.0
2.5
2.0
1.5
1.0
0.5
0.0
-0.5
f1 (ppm)

NMR-118

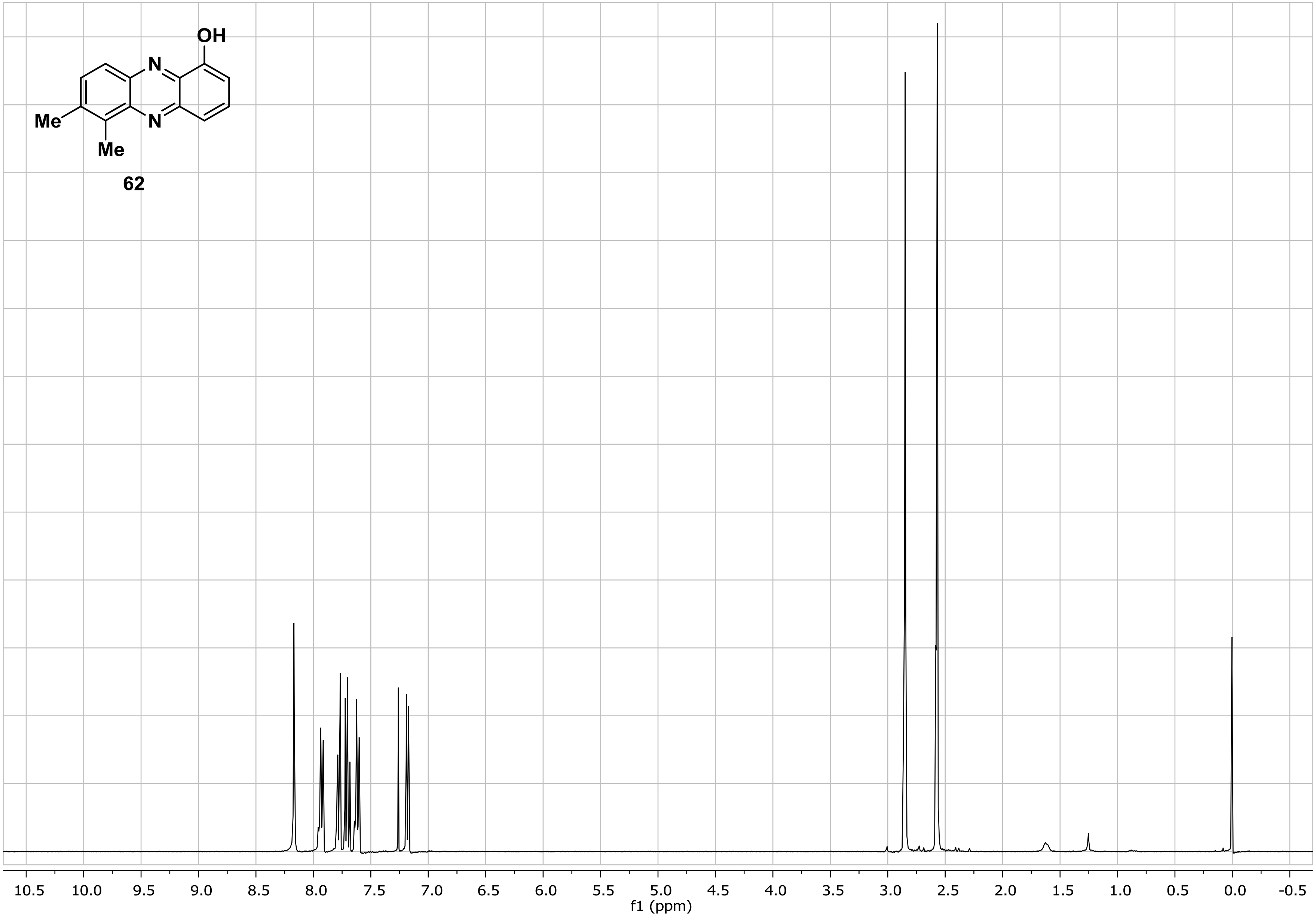
OH
N
N
Me
Me
62
10.5
10.0
9.5
9.0
8.5
8.0
7.5
7.0
6.5
6.0
5.5
5.0
4.5
4.0
3.5
3.0
2.5
2.0
1.5
1.0
0.5
0.0
-0.5
f1 (ppm)

NMR-119

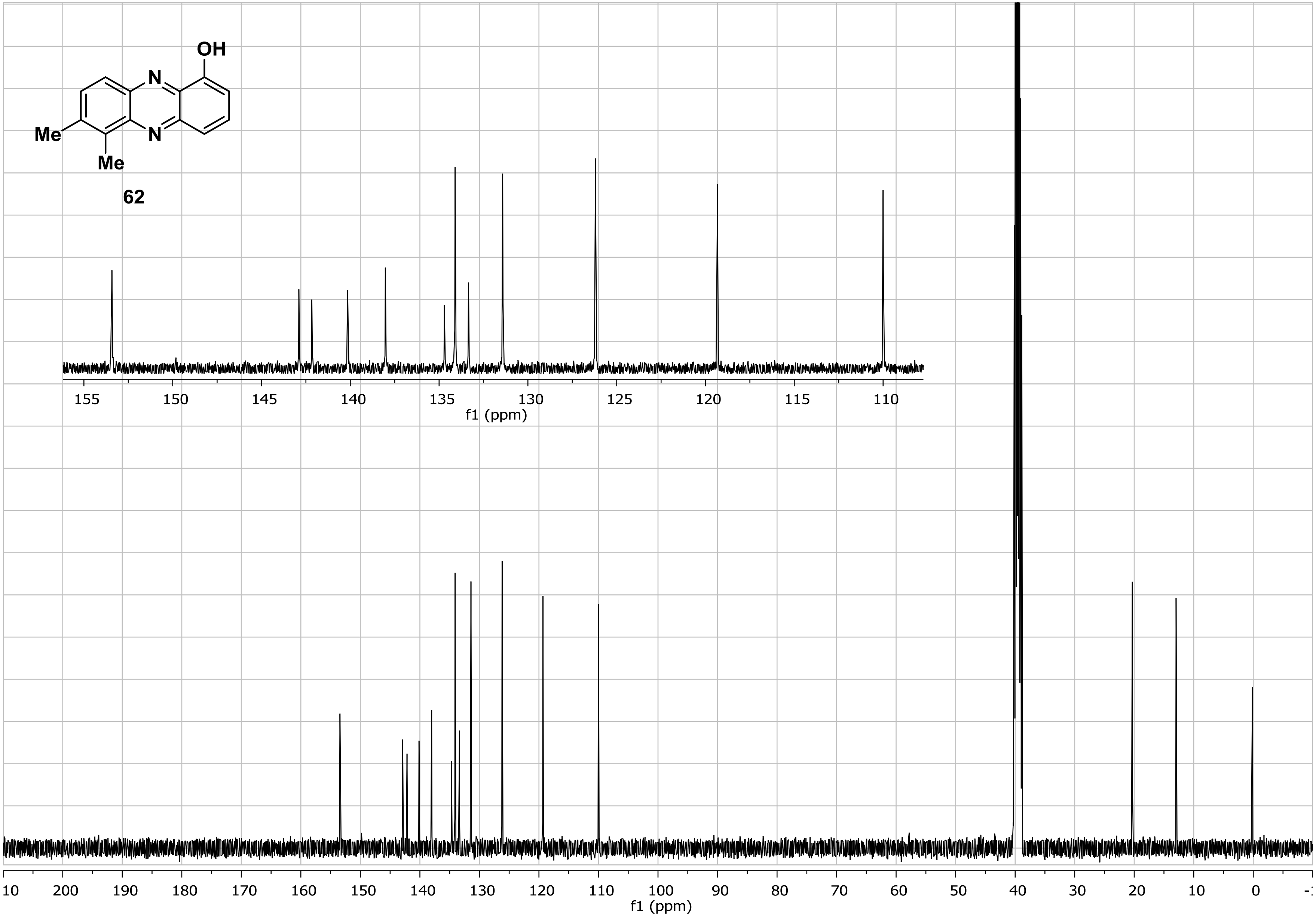
OH
N
N
Me
Me
62
155
150
145
140
135
130
125
120
115
110
f1 (ppm)
10
200
190
180
170
160
150
140
130
120
110
100
90
80
70
60
50
40
30
20
10
0
f1 (ppm)

NMR-120

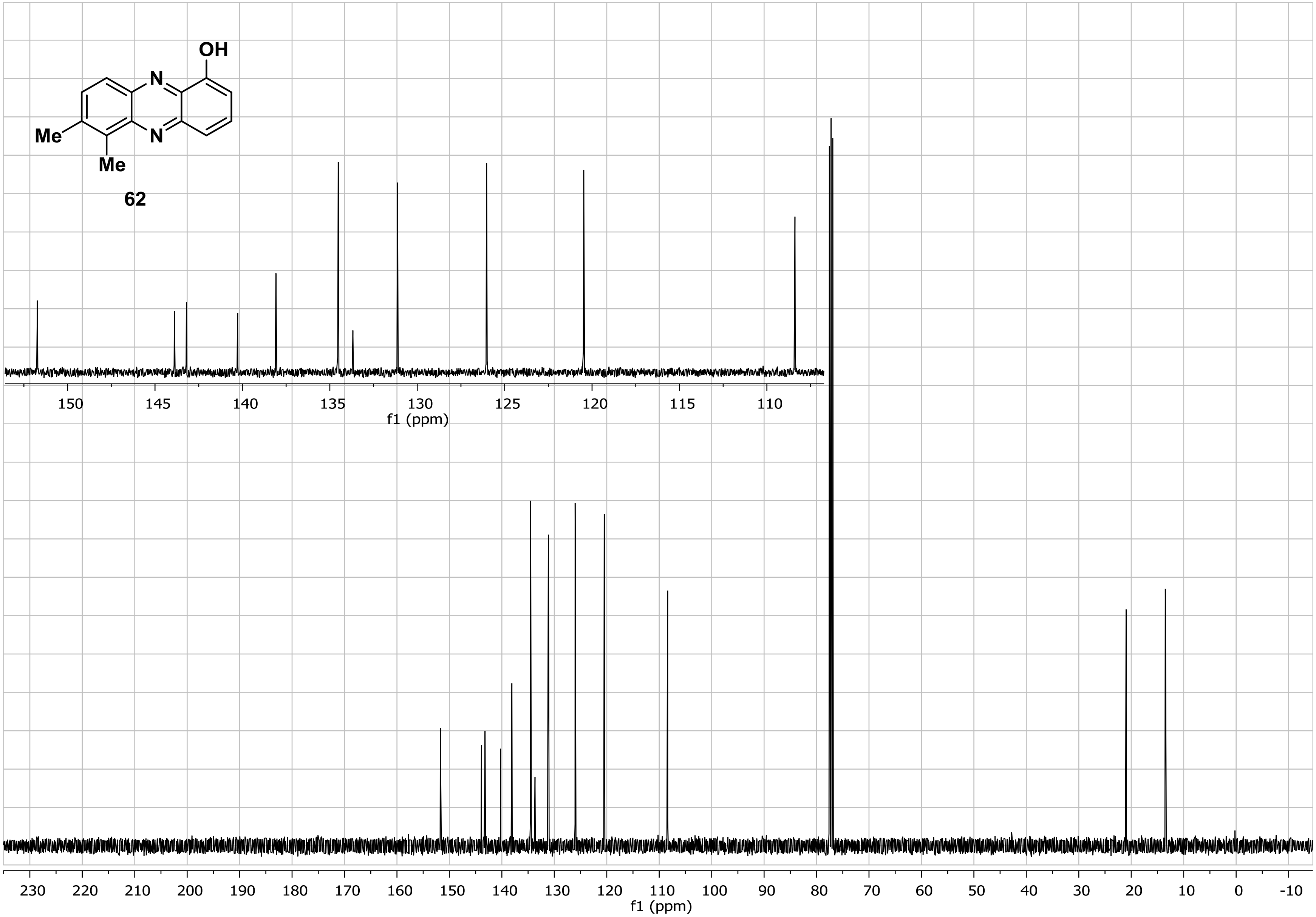
OH
N
N
Me
Me
62
150 145 140 135 130 125 120 115 110
f1 (ppm)
230 220 210 200 190 180 170 160 150 140 130 120 110 100 90 80 70 60 50 40 30 20 10 0 -10
f1 (ppm)

NMR-121

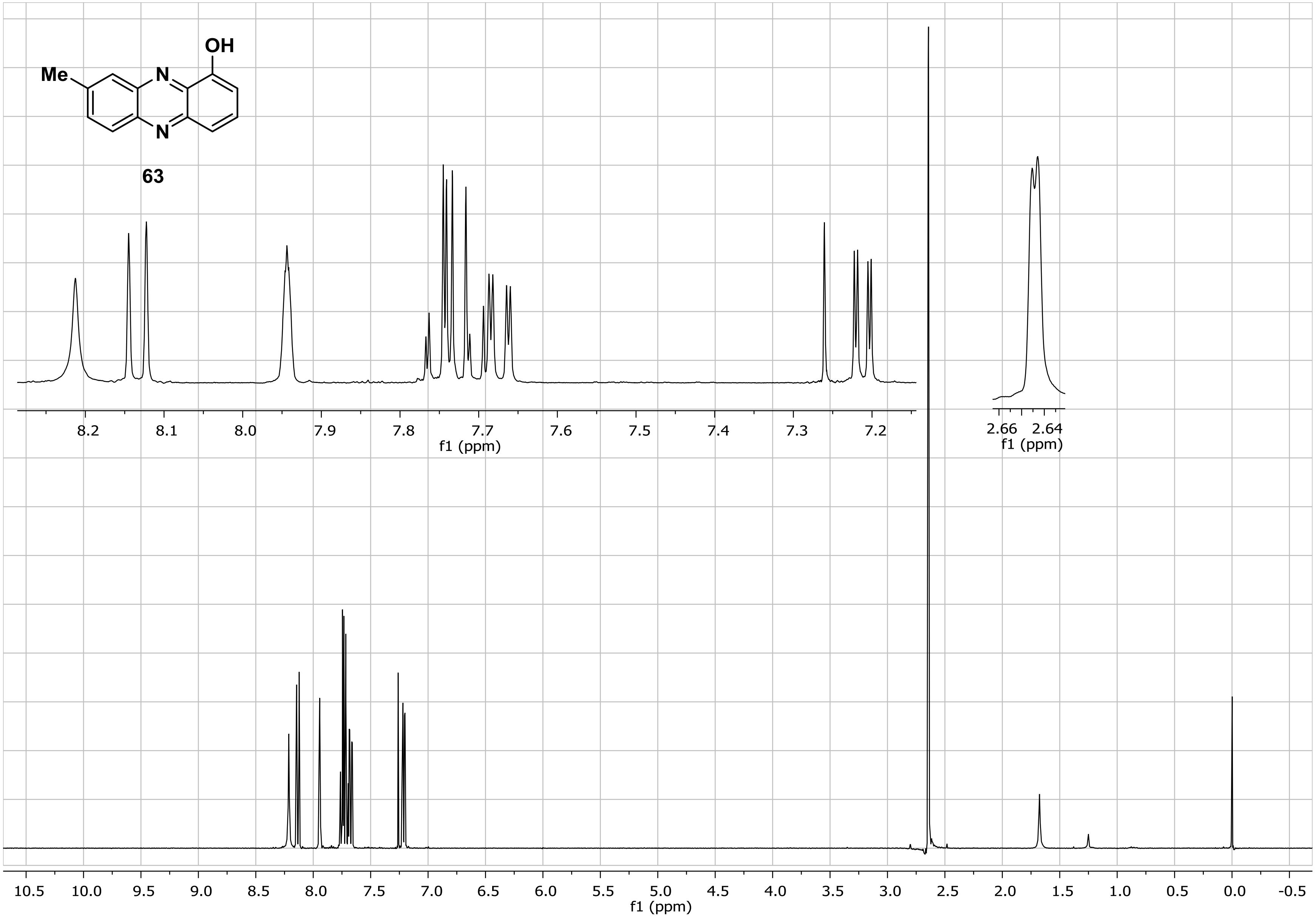

OH
Me
N
N
63
8.2
8.1
8.0
7.9
7.8
7.7
7.6
7.5
7.4
7.3
7.2
f1 (ppm)
2.66
2.64
f1 (ppm)
10.5
10.0
9.5
9.0
8.5
8.0
7.5
7.0
6.5
6.0
5.5
5.0
4.5
4.0
3.5
3.0
2.5
2.0
1.5
1.0
0.5
0.0
-0.5
f1 (ppm)

NMR-122

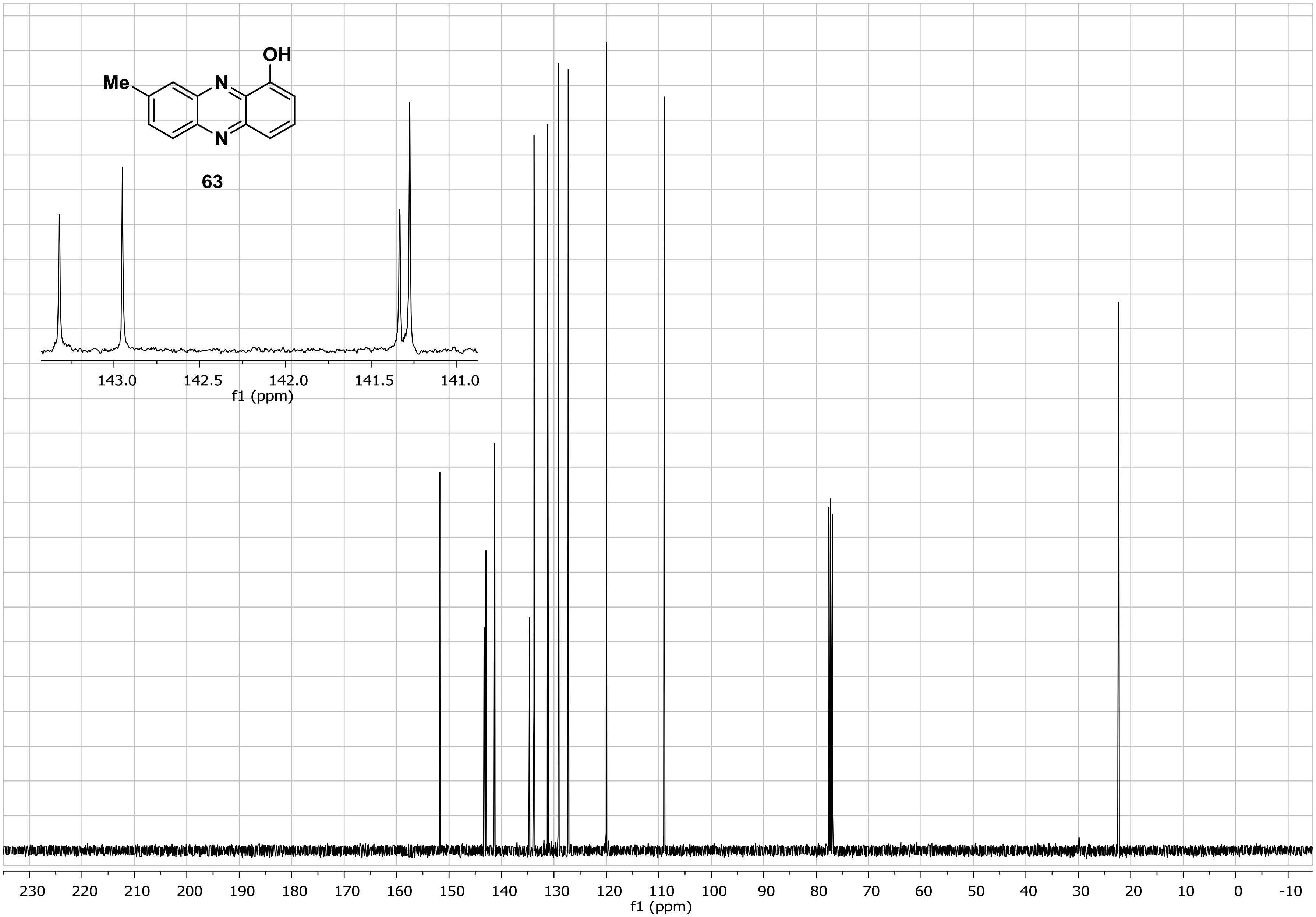
OH
Me
N
N
63
143.0
142.5
142.0
141.5
141.0
f1 (ppm)
230
220
210
200
190
180
170
160
150
140
130
120
110
100
90
80
70
60
50
40
30
20
10
0
-10
f1 (ppm)

NMR-123

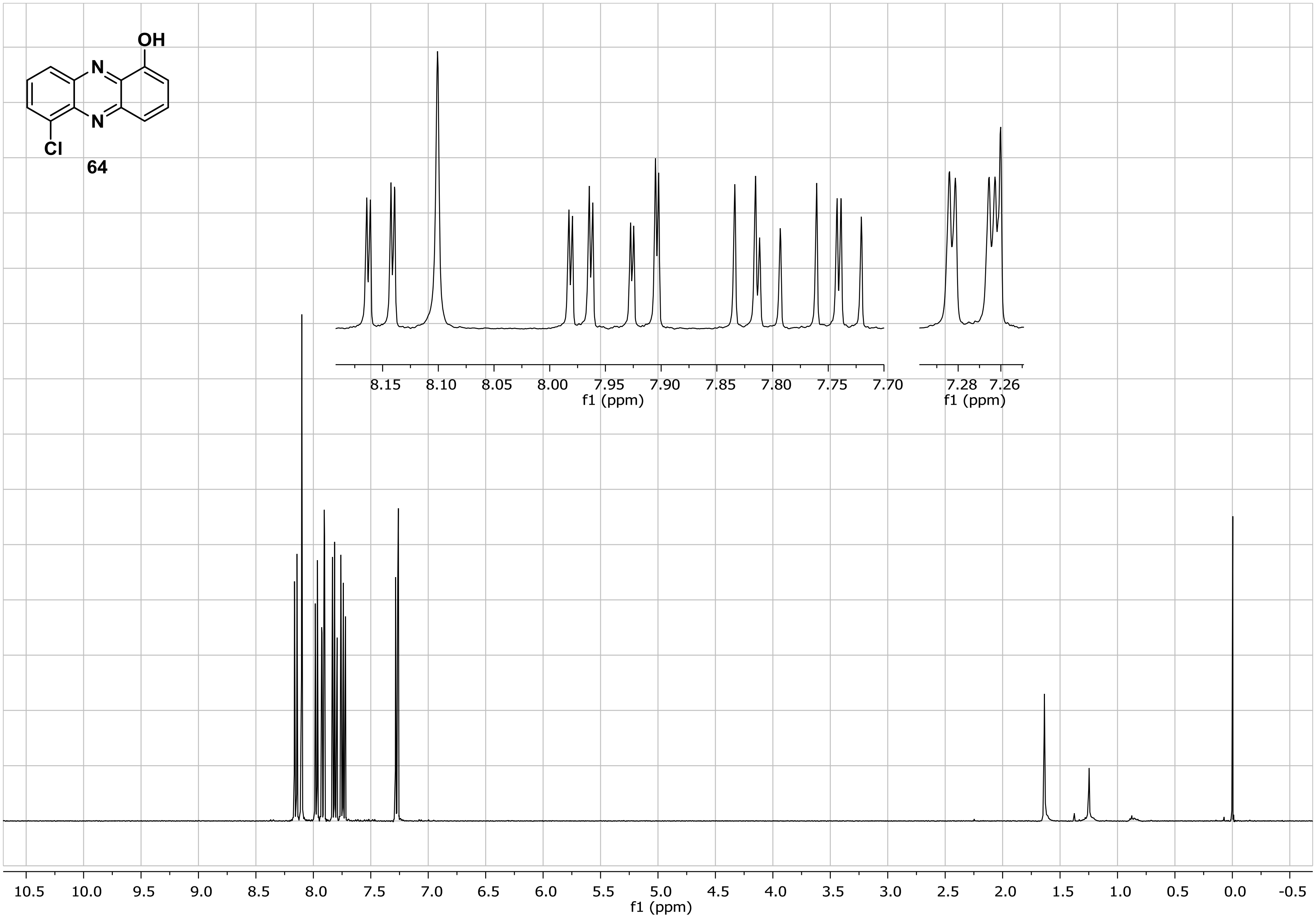
OH
N
N
Cl
64
8.15 8.10 8.05 8.00 7.95 7.90 7.85 7.80 7.75 7.70
f1 (ppm)
7.28 7.26
f1 (ppm)
10.5 10.0 9.5 9.0 8.5 8.0 7.5 7.0 6.5 6.0 5.5 5.0 4.5 4.0 3.5 3.0 2.5 2.0 1.5 1.0 0.5 0.0 -0.5
f1 (ppm)

NMR-124

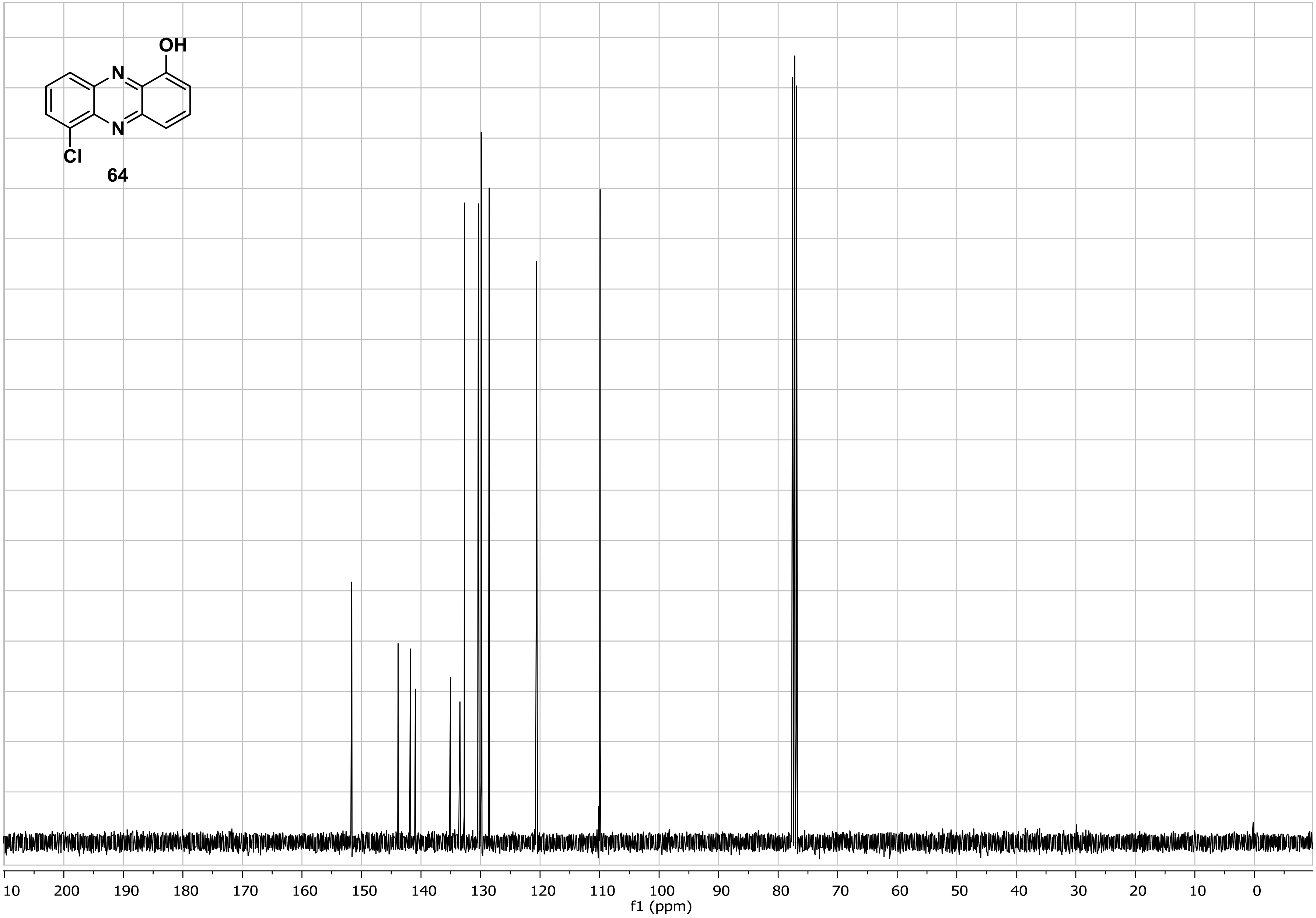

NMR-125

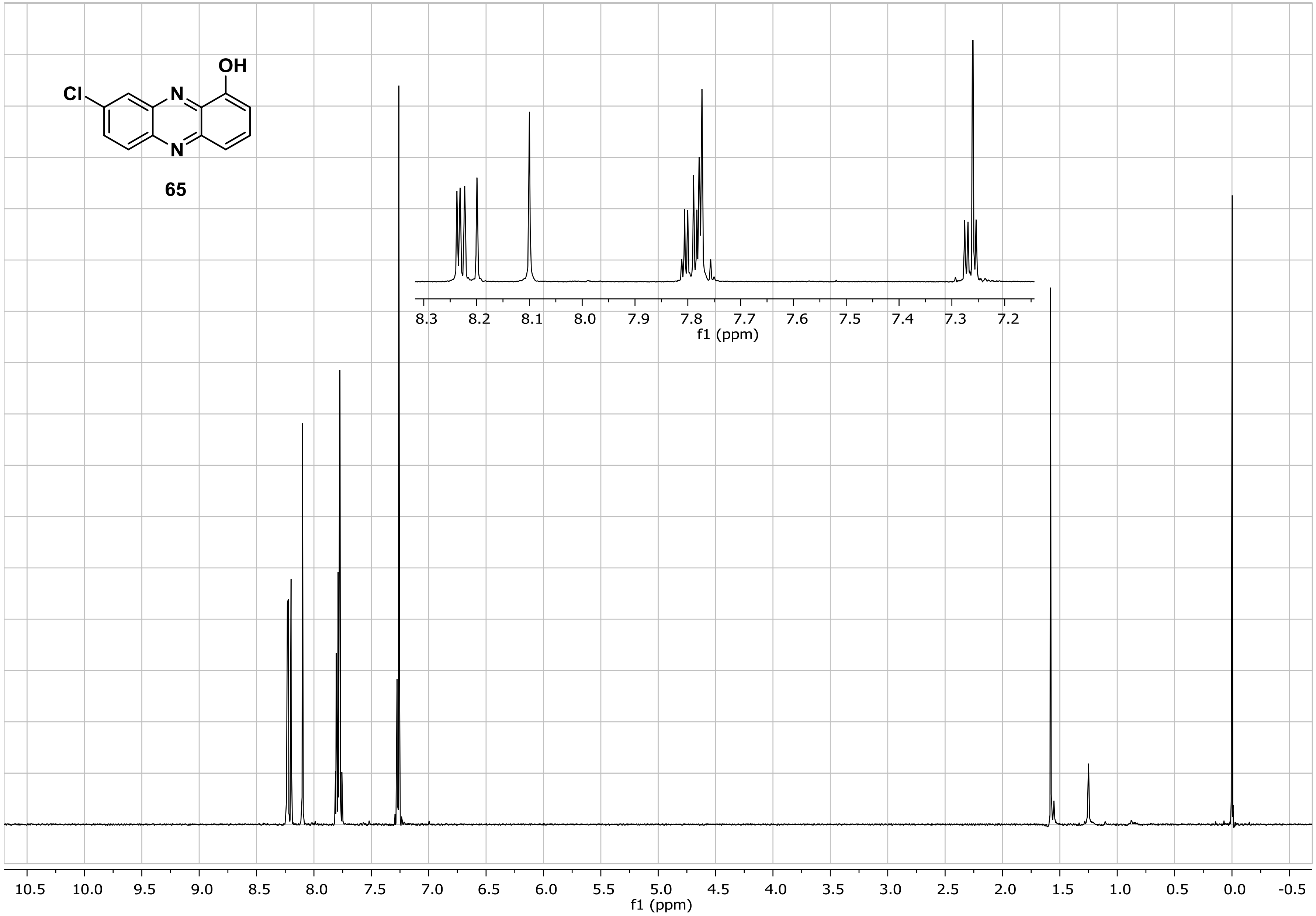

OH
Cl
N
N
65
8.3
8.2
8.1
8.0
7.9
7.8
7.7
7.6
7.5
7.4
7.3
7.2
f1 (ppm)
10.5
10.0
9.5
9.0
8.5
8.0
7.5
7.0
6.5
6.0
5.5
5.0
4.5
4.0
3.5
3.0
2.5
2.0
1.5
1.0
0.5
0.0
-0.5
f1 (ppm)

NMR-126

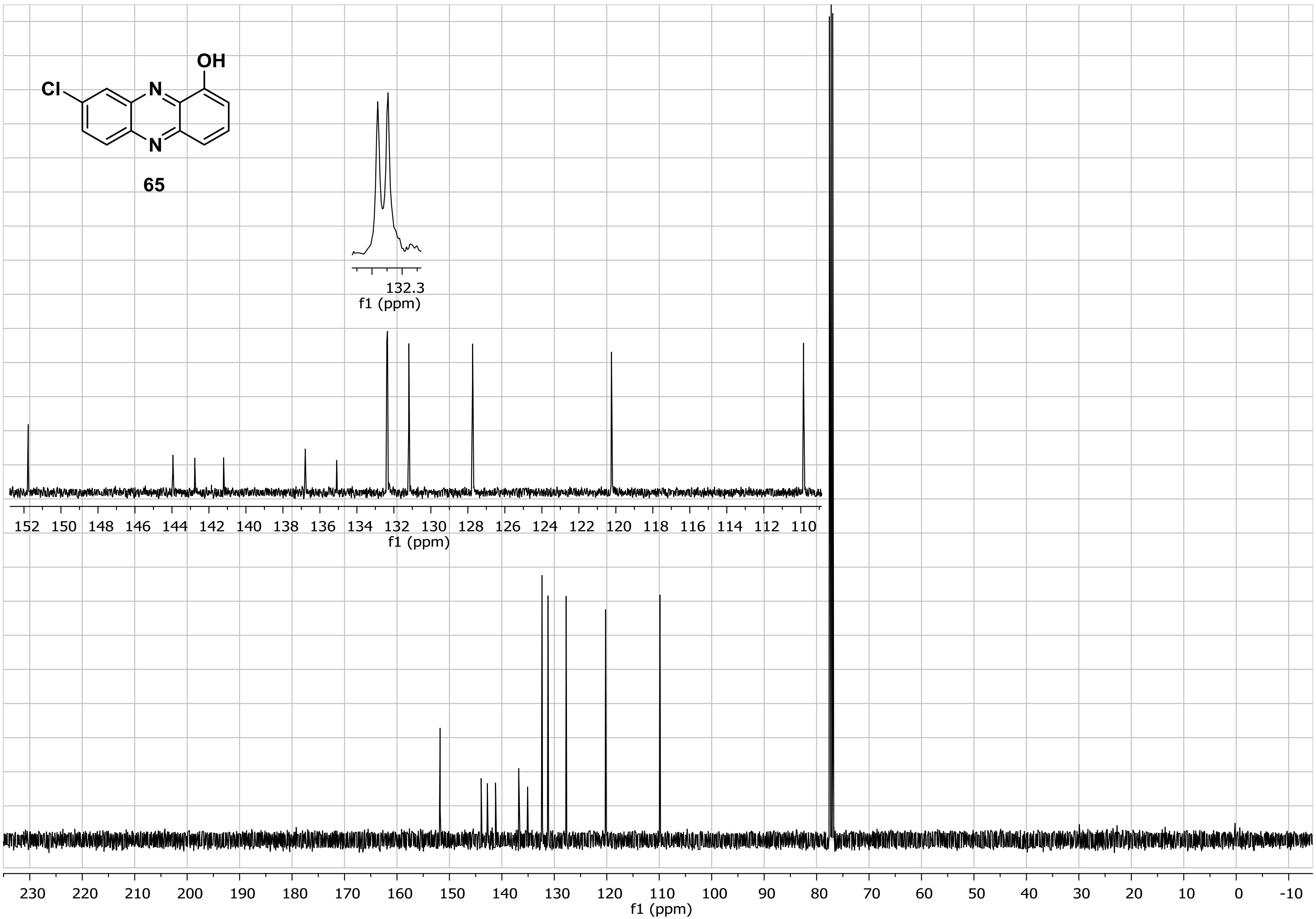
OH
Cl
N
N
65
132.3
f1 (ppm)
152 150 148 146 144 142 140 138 136 134 132 130 128 126 124 122 120 118 116 114 112 110
f1 (ppm)
230 220 210 200 190 180 170 160 150 140 130 120 110 100 90 80 70 60 50 40 30 20 10 0 -10
f1 (ppm)

NMR-127

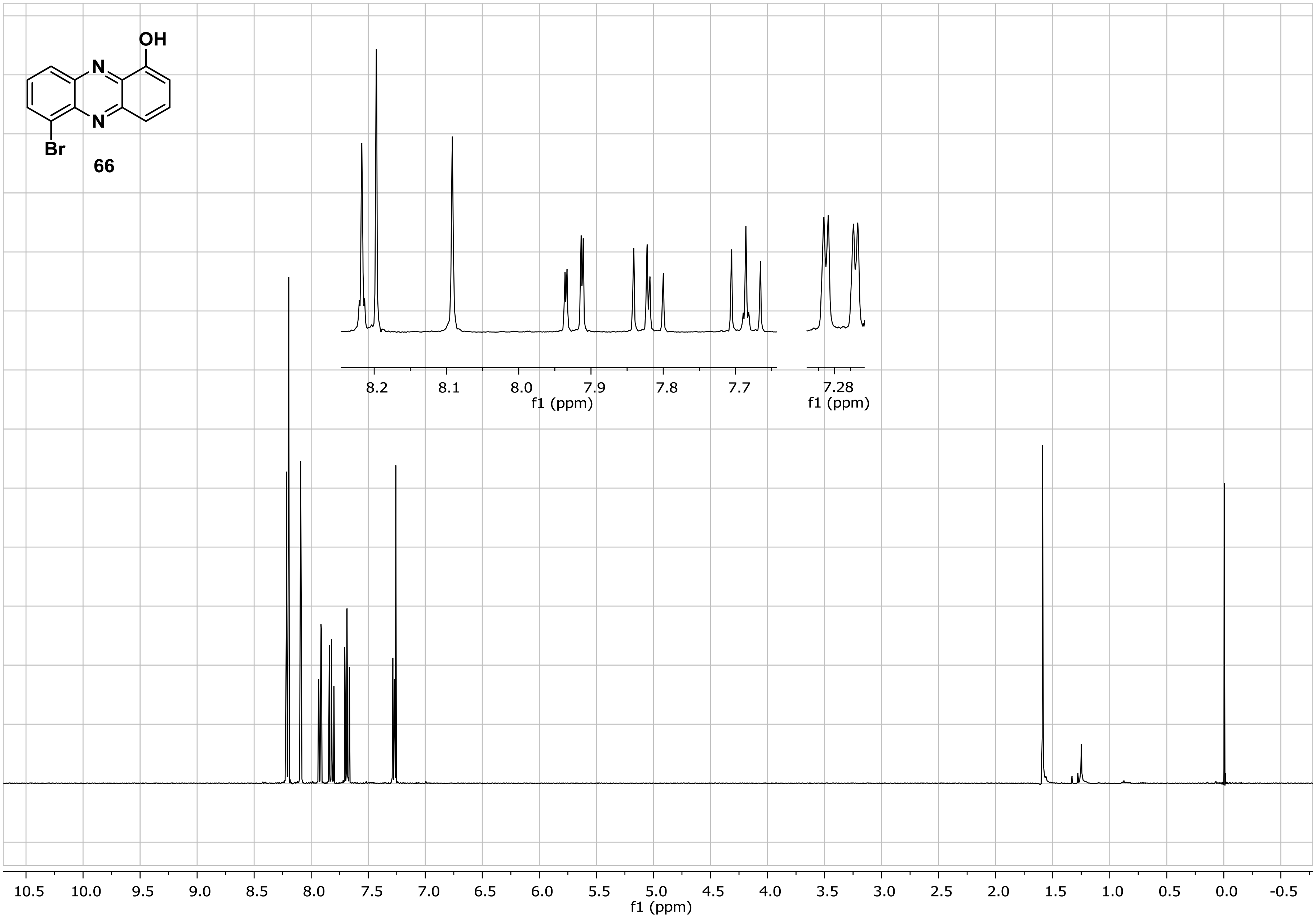

OH
N
N
Br
66
8.2
8.1
8.0
7.9
7.8
7.7
f1 (ppm)
7.28
f1 (ppm)
10.5
10.0
9.5
9.0
8.5
8.0
7.5
7.0
6.5
6.0
5.5
5.0
4.5
4.0
3.5
3.0
2.5
2.0
1.5
1.0
0.5
0.0
-0.5
f1 (ppm)

NMR-128

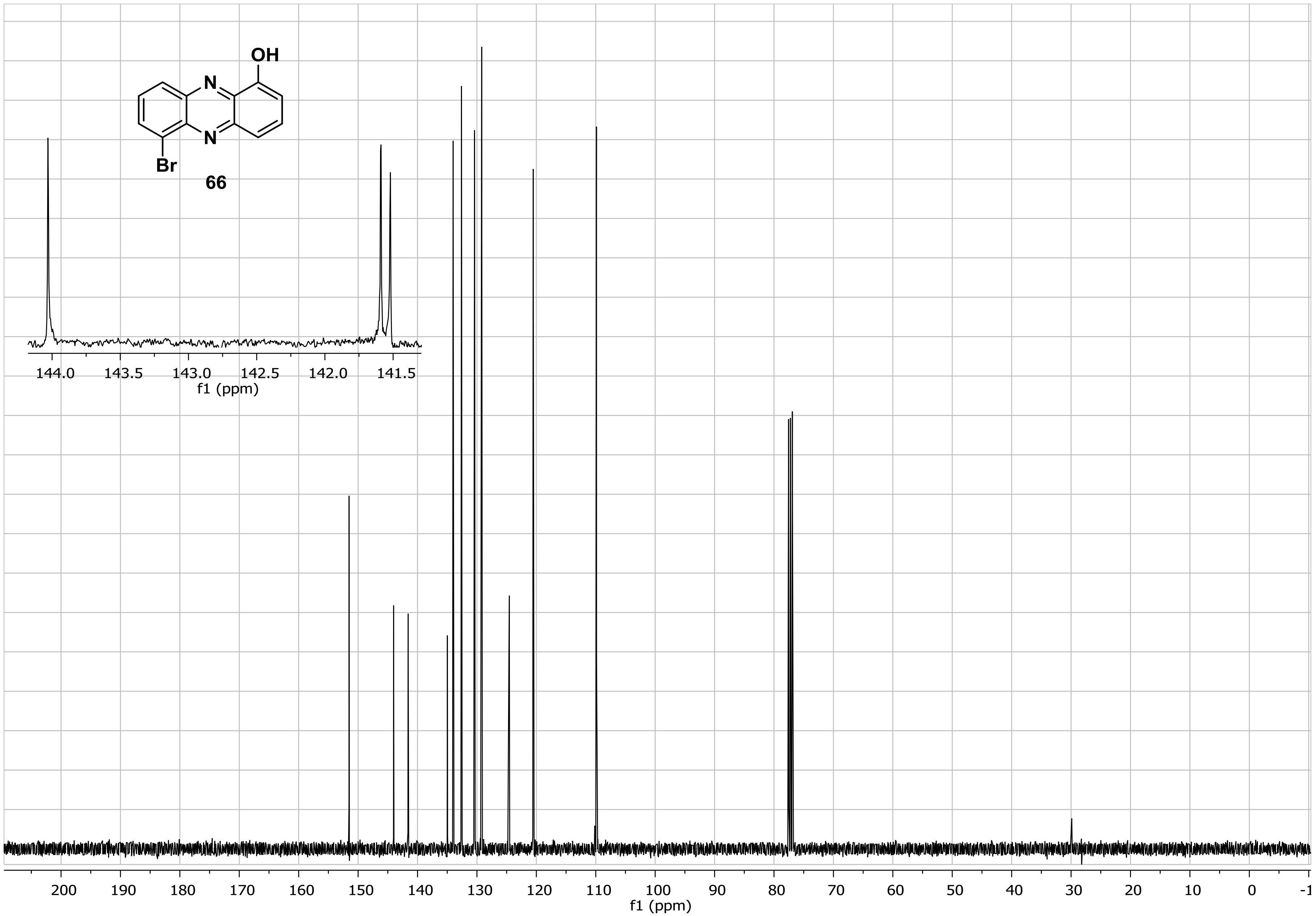

OH
N
N
Br
66
144.0
143.5
143.0
142.5
142.0
141.5
f1 (ppm)
200
190
180
170
160
150
140
130
120
110
100
90
80
70
60
50
40
30
20
10
0
-1
f1 (ppm)

NMR-129
